# Novel insights into the genetics of smoking behaviour, lung function, and chronic obstructive pulmonary disease (UK BiLEVE): a genetic association study in UK Biobank

**DOI:** 10.1016/S2213-2600(15)00283-0

**Published:** 2015-10

**Authors:** Louise V Wain, Nick Shrine, Suzanne Miller, Victoria E Jackson, Ioanna Ntalla, María Soler Artigas, Charlotte K Billington, Abdul Kader Kheirallah, Richard Allen, James P Cook, Kelly Probert, Ma'en Obeidat, Yohan Bossé, Ke Hao, Dirkje S Postma, Peter D Paré, Adaikalavan Ramasamy, UK Brain Expression Consortium (UKBEC), Reedik Mägi, Evelin Mihailov, Eva Reinmaa, Erik Melén, Jared O'Connell, Eleni Frangou, Olivier Delaneau, OxGSK Consortium, Colin Freeman, Desislava Petkova, Mark McCarthy, Ian Sayers, Panos Deloukas, Richard Hubbard, Ian Pavord, Anna L Hansell, Neil C Thomson, Eleftheria Zeggini, Andrew P Morris, Jonathan Marchini, David P Strachan, Martin D Tobin, Ian P Hall

**Affiliations:** a Department of Health Sciences, University of Leicester, Leicester, UK; b Division of Respiratory Medicine, Queen's Medical Centre, University of Nottingham, Nottingham, UK; c Faculty of Medicine and Health Sciences, School of Medicine, University of Nottingham, Nottingham, UK; d University of British Columbia Centre for Heart Lung Innovation, St Paul's Hospital, Vancouver, BC, Canada; e Institut universitaire de cardiologie et de pneumologie de Québec, Department of Molecular Medicine, Laval University, Québec, QC, Canada; f Department of Genetics and Genomic Sciences, Icahn School of Medicine at Mount Sinai, New York, NY, USA; g Icahn Institute of Genomics and Multiscale Biology, Icahn School of Medicine at Mount Sinai, New York, NY, USA; h Department of Respiratory Medicine, Shanghai Tenth People's Hospital, Tongji University, Shanghai, China; i Department of Pulmonary Medicine and Tuberculosis, University of Groningen, University Medical Center Groningen, Groningen, Netherlands; j Department of Molecular Neuroscience, UCL Institute of Neurology, London, UK; k Department of Medical and Molecular Genetics, King's College London, London, UK; l Jenner Institute, University of Oxford, Oxford, UK; m Department of Statistics, University of Oxford, Oxford, UK; n Wellcome Trust Centre for Human Genetics, University of Oxford, Oxford, UK; o Centre for Statistics in Medicine, Nuffield Department of Orthopaedics, Rheumatology and Musculoskeletal Sciences, University of Oxford, Oxford, UK; p Oxford Centre for Diabetes, Endocrinology and Metabolism, University of Oxford, Oxford, UK; q Respiratory Medicine, University of Oxford, Oxford, UK; r Estonian Genome Center, University of Tartu, Tartu, Estonia; s Institute of Environmental Medicine, Karolinska Institutet and Sachs' Children's Hospital, Stockholm, Sweden; t Department of Genetic Medicine and Development, University of Geneva Medical School, Geneva, Switzerland; u William Harvey Research Institute, Barts and The London School of Medicine and Dentistry, Queen Mary University London, London, UK; v Princess Al-Jawhara Al-Brahim Centre of Excellence in Research of Hereditary Disorders (PACER-HD), King Abdulaziz University, Jeddah, Saudi Arabia; w UK Small Area Health Statistics Unit, MRC-PHE Centre for Environment and Health, School of Public Health, Imperial College London, London, UK; x Imperial College Healthcare NHS Trust, St Mary's Hospital, Paddington, London, UK; y Institute of Infection, Immunity and Inflammation, University of Glasgow, Glasgow, UK; z Wellcome Trust Sanger Institute, Hinxton, Cambridgeshire, UK; aa Department of Biostatistics, University of Liverpool, Liverpool, UK; ab Population Health Research Institute, St George's, University of London, London, UK; ac National Institute for Health Research, Leicester Respiratory Biomedical Research Unit, Glenfield Hospital, Leicester, UK

## Abstract

**Background:**

Understanding the genetic basis of airflow obstruction and smoking behaviour is key to determining the pathophysiology of chronic obstructive pulmonary disease (COPD). We used UK Biobank data to study the genetic causes of smoking behaviour and lung health.

**Methods:**

We sampled individuals of European ancestry from UK Biobank, from the middle and extremes of the forced expiratory volume in 1 s (FEV_1_) distribution among heavy smokers (mean 35 pack-years) and never smokers. We developed a custom array for UK Biobank to provide optimum genome-wide coverage of common and low-frequency variants, dense coverage of genomic regions already implicated in lung health and disease, and to assay rare coding variants relevant to the UK population. We investigated whether there were shared genetic causes between different phenotypes defined by extremes of FEV_1_. We also looked for novel variants associated with extremes of FEV_1_ and smoking behaviour and assessed regions of the genome that had already shown evidence for a role in lung health and disease. We set genome-wide significance at p<5 × 10^−8^.

**Findings:**

UK Biobank participants were recruited from March 15, 2006, to July 7, 2010. Sample selection for the UK BiLEVE study started on Nov 22, 2012, and was completed on Dec 20, 2012. We selected 50 008 unique samples: 10 002 individuals with low FEV_1_, 10 000 with average FEV_1_, and 5002 with high FEV_1_ from each of the heavy smoker and never smoker groups. We noted a substantial sharing of genetic causes of low FEV_1_ between heavy smokers and never smokers (p=2·29 × 10^−16^) and between individuals with and without doctor-diagnosed asthma (p=6·06 × 10^−11^). We discovered six novel genome-wide significant signals of association with extremes of FEV_1_, including signals at four novel loci (KANSL1, TSEN54, TET2, and RBM19/TBX5) and independent signals at two previously reported loci (NPNT and HLA-DQB1/HLA-DQA2). These variants also showed association with COPD, including in individuals with no history of smoking. The number of copies of a 150 kb region containing the 5′ end of KANSL1, a gene that is important for epigenetic gene regulation, was associated with extremes of FEV_1_. We also discovered five new genome-wide significant signals for smoking behaviour, including a variant in *NCAM1* (chromosome 11) and a variant on chromosome 2 (between *TEX41* and *PABPC1P2*) that has a *trans* effect on expression of *NCAM1* in brain tissue.

**Interpretation:**

By sampling from the extremes of the lung function distribution in UK Biobank, we identified novel genetic causes of lung function and smoking behaviour. These results provide new insight into the specific mechanisms underlying airflow obstruction, COPD, and tobacco addiction, and show substantial shared genetic architecture underlying airflow obstruction across individuals, irrespective of smoking behaviour and other airway disease.

**Funding:**

Medical Research Council.

## Introduction

Chronic obstructive pulmonary disease (COPD) is a global public health concern and is currently the third leading cause of death worldwide.^1^ Smoking and indoor air pollution are major environmental risk factors for development of COPD, but heritability studies also suggest a strong genetic component in smoking behaviour and in risk of COPD.^1–4^ Spirometry, particularly measurements of forced expiratory volume in 1 s (FEV_1_) and forced vital capacity (FVC), is used to measure airflow obstruction and helps in the diagnosis and grading of severity of COPD. Previous large genome-wide association studies (GWAS) of general population cohorts have identified 32 common genetic variants (minor allele frequency [MAF] >5%) associated with lung function,^5–9^ 12 of which have also shown association with airflow obstruction and risk of COPD.^10–15^ However, these findings only explain a small proportion of the phenotypic variance (∼1·5% for FEV_1_).^8^

**Research in context**

**Evidence before this study**

UK Biobank had completed its recruitment, including its baseline phenotyping and biobanking of samples, before our study began. The DNA had not yet been extracted from the biobanked samples, and the spirometry data quality had not yet been analysed across all UK Biobank participants. We searched for evidence of other large biobanks with spirometry data, including the P3G Catalogue. We did not identify any other biobank with spirometry data and DNA as large as UK Biobank. Evidence regarding the global burden of disease due to smoking or chronic obstructive pulmonary disease (COPD) was obtained from the WHO Global Health Risks Report and a systematic analysis of the Global Burden of Disease Study 2010. Tobacco smoking accounted for about 5·1 million deaths globally in 2004; because of recent increases in smoking prevalence in developing countries, the full global effect of smoking is yet to occur. COPD is the third leading cause of death globally. For previous evidence of genetic associations, we gave the highest ranking to associations reaching genome-wide significance in genome-wide association studies, a lower ranking to associations not reaching genome-wide significance in genome-wide association studies, and the lowest ranking to associations reported in candidate gene studies. To assess evidence of loci associated with lung function, COPD, and smoking behaviour, we queried the Catalog of Published Genome-Wide Association Studies. We used this evidence to report our known findings for genetic variants shown to be associated with forced expiratory volume in 1 s (FEV_1_; eight loci) and smoking behaviour (seven loci). We report candidate gene associations only for variants for which we found genome-wide evidence of association.

**Added value of this study**

We describe, to our knowledge, the first genetic study using the UK Biobank resource and show the quality of the phenotype and genotype data. Additionally, we describe an advance in imputation quality afforded by the use of a newly designed genotyping array used in conjunction with the largest reference panel available so far. A slightly modified version of this array is being used to genotype the remaining samples in UK Biobank. As evidence of the usefulness of these data, we describe novel insights into the genetic architecture of airflow obstruction and smoking. Specifically, we show that there are shared genetic causes of airflow obstruction between smokers and non-smokers, consistent with the limited evidence for gene–smoking interactions described so far. We show that the genetic determinants of low FEV_1_ in individuals without asthma are also informative in individuals with asthma. We report new loci associated with extremes of FEV_1_ and COPD, including evidence that a genomic region of complex structural variation has an effect on lung function and airflow obstruction in the general population. Our novel signals implicate epigenetic mechanisms as contributors to lung health. These findings, taken together with previous findings, will help define pathways underlying predisposition to development of COPD and smoking behaviours. A full understanding of the biological mechanisms underlying these genetic associations will improve our understanding of the pathophysiology of COPD and smoking behaviour, and potentially give rise to novel therapeutic strategies for the management of airway disease and prevention of nicotine addiction.

**Implications of all the available evidence**

This study has improved our understanding of the genetic and molecular basis of smoking behaviour and lung function and provided potential targets for therapeutic intervention. It has also shown the value of sampling from the extremes using a large biobank such as UK Biobank. A similar approach could be adopted for genetic studies of other health-related traits in UK Biobank, using either new genetic assays or the extensive genome-wide data that we and UK Biobank have generated.

Tobacco smoking accounted for about 5·1 million deaths globally in 2004 and for 18% of deaths in high-income countries.^16^ Large GWAS of smoking behaviour^17–19^ have identified up to eight associated loci; the strongest association reported is at the 15q25 locus.^17–19^ Further insight into the genetic factors affecting lung function, smoking behaviour, and COPD could lead to new approaches for smoking cessation and prevention and treatment of COPD.

UK Biobank is the largest European biobank available at present and represents an extensive resource from which to sample phenotypic extremes in the UK population.^20^ UK Biobank contains data from 502 682 individuals (94% of self-reported European ancestry), with extensive health and lifestyle questionnaire data, physical measures (including spirometry), and DNA.

In the UK Biobank Lung Exome Variant Evaluation (UK BiLEVE) study, we undertook nested case-control studies in individuals of European ancestry from UK Biobank to: (1) identify whether there are shared genetic causes underlying low FEV_1_ and high FEV_1_, and a shared genetic cause of low FEV_1_ between never smokers and heavy smokers and between individuals with and without a doctor diagnosis of asthma; (2) identify novel variants associated with extremes of FEV_1_ and smoking behaviour; and (3) provide further insight into regions of the genome that had already shown evidence for a role in lung health and disease.

## Methods

### Study design

We defined case and control groups by selecting individuals from the middle and extremes of the FEV_1_ distribution among both heavy smokers (mean 35 pack-years) and never smokers. We developed a custom array to provide optimum genome-wide coverage of common and low frequency (MAF 1–5%) coding variants and rare (MAF <1%) coding variants relevant to the UK population; this platform also provided dense coverage of genomic regions implicated in lung health and disease. Spirometry data in UK Biobank were obtained using a Vitalograph Pneumotrac 6800 (Buckingham, UK) on at least two occasions. Sampling was undertaken such that equal numbers of males and females were selected in total and the numbers of individuals selected from each age–sex band were proportional to the number of individuals in the band being sampled (appendix pp 3–5). One consequence of this approach is that we enriched our sample for non-smoking individuals with airflow obstruction.

To assess whether the novel regions that we identified as associated with FEV_1_ extremes are also associated with COPD, we defined individuals fulfilling spirometric criteria for the Global Initiative for Chronic Obstructive Lung Disease (GOLD) Stage 2+ COPD (FEV_1_:FVC ratio <0·7 and percent predicted FEV_1_<80%) as COPD cases and we defined individuals with FEV_1_:FVC ratio >0·7 and percent predicted FEV_1_ in excess of 80% from the high FEV_1_ strata as controls. Post-bronchodilator spirometry was not available, although drug treatment was not withheld before spirometry.

To assess the extent of the shared genetic causes of low FEV_1_ between individuals with and without reported or doctor-diagnosed asthma, we identified individuals within our study selection who were also asthma cases as participants who either (1) answered “asthma” to the touch-screen question “Has a doctor ever told you that you have had any of the following conditions?” or (2) reported asthma in a verbal interview at the time of recruitment to UK Biobank.

UK Biobank has received ethics approval from the National Health Service National Research Ethics Service (Ref 11/NW/0382).

### Procedures

We undertook genome-wide genotyping of variants using a new custom Affymetrix Axiom array (UK BiLEVE array; Santa Clara, CA, USA; appendix pp 5–8) that was designed to (1) measure rare coding variation; (2) provide a framework for optimum imputation of non-genotyped variants that are common (MAF >5%) or of low frequency (MAF 1–5%) in the European population, when used in conjunction with a large imputation reference panel of individuals with whole-genome sequence data;^21^ and (3) optimise coverage of genes and genomic regions with established or putative roles in lung health and disease to enable fine mapping. After thorough sample and variant quality control (appendix pp 8–15), we imputed non-genotyped variants using a combined 1000 Genomes Project Phase 1^22^ and UK10K Project^23,24^ reference panel (appendix pp 15–16). The data were used to finalise the design of the UK Biobank array, which is being used for genome-wide genotyping and imputation of the remaining UK Biobank participants.

Using data from previously published studies of whole-genome gene expression and genome-wide genotyping,^25–29^ we assessed whether variants at associated loci (identified as described in the Statistical analysis) regulate levels of mRNA. These expression quantitative trait loci (eQTL) studies included non-tumour lung tissue, blood, and, for variants associated with smoking behaviour, brain. For genes close to peaks of novel signals or genes implicated through eQTL, we assessed differential expression in the lungs of individuals with and without COPD and differential expression in the pseudoglandular and canalicular stages of development of the fetal lung.^30,31^ Additionally, we generated RNA sequencing data to discover novel transcripts of these genes in human bronchial epithelial cells. We tested all genome-wide meta-analysis p values for enrichment in biological pathways defined in publicly available databases. All functional analyses are described in detail in the appendix (pp 21–23).

### Statistical analysis

Case-control comparisons of low FEV_1_ versus high FEV_1_, low FEV_1_ versus average FEV_1_, and high FEV_1_ versus average FEV_1_ were done within each of the heavy and never smokers subsets separately (appendix p 17). To identify whether any individual variants had a significantly different effect on the risk of airflow obstruction in heavy smokers compared with never smokers, we tested for interaction with smoking (appendix p 17). We calculated the proportion of the variance in FEV_1_ explained by genetic variants (appendix p 17). We compared heavy versus never smokers to identify loci associated with smoking behaviour. Association testing was done using a Score test (and Firth test for variants with minor allele count <400)^32^ with imputed marker doses, adjusting for pack-years in smokers and ten principal components. Full genome-wide association results are available via UK Biobank (appendix p 17). For genome-wide association analyses, we set genome-wide significance as p<5 × 10^−8^ and suggestive significance as 5 × 10^−8^<p<5 × 10^−7^. For other analyses, we used a Bonferroni correction for multiple testing. The appendix (p 17) describes quality control after association testing. For the lead single nucleotide polymorphism (SNP) at each of our novel signals of association with FEV_1_ extremes, we tested for association with COPD risk using the aforementioned definition (appendix p 18). We did a meta-analysis across smoking strata using inverse variance weighting. We assessed evidence for polygenic architecture of FEV_1_-defined phenotypes (appendix p 18).^33^ For this analysis, we created discovery and target subpopulations, each of which comprised cases and control groups created by randomly splitting the low FEV_1_ and average FEV_1_ groups (appendix pp 18–20). Variants of MAF of at least 1% associated with low FEV_1_ below given p value thresholds in the discovery population were incorporated into an aggregate score, and the association with the aggregate score was tested in the independent target population. A similar approach (appendix pp 18–20), in each case using independent discovery and target populations, was used to test for a shared polygenic component between high FEV_1_ and low FEV_1_, low FEV_1_ in heavy smokers and in never smokers, and low FEV_1_ in participants who did and those who did not report a history of doctor-diagnosed asthma. To show the reliability of the doctor diagnosis of asthma variable, we showed association with asthma at ten previously reported genome-wide significant loci (appendix pp 21, 29).

### Role of the funding source

The funder of the study had no role in study design, data collection, data analysis, data interpretation, or writing of the report. The corresponding authors had full access to all the data in the UK BiLEVE study and had final responsibility for the decision to submit for publication.

## Results

**Figure 1:**
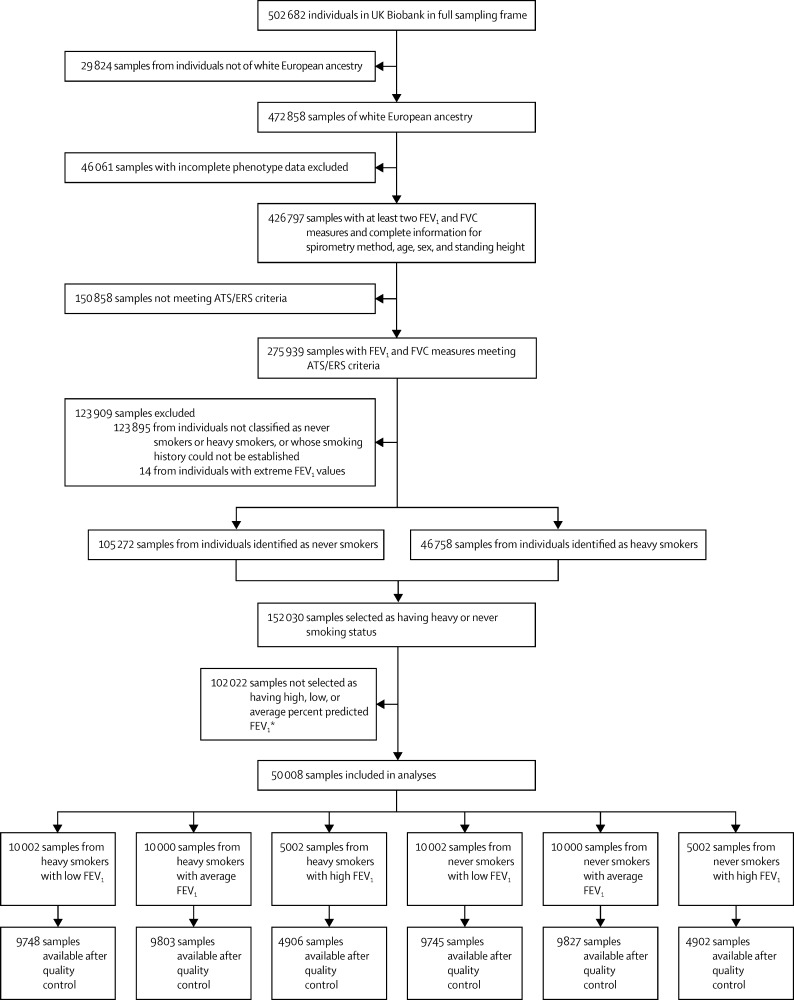
Sample selection strategy ATS=American Thoracic Society. ERS=European Respiratory Society.^34^ FEV_1_=forced expiratory volume in 1 s. FVC= forced vital capacity. *See appendix (pp 3–5) for more details of sample selection.

UK Biobank participants were recruited from March 15, 2006, to July 7, 2010. Sample selection for the UK BiLEVE study started on Nov 22, 2012, and was completed on Dec 20, 2012. We initially selected 50 008 unique samples representing the extremes and middle of the percent predicted FEV_1_ distributions; this comprised 10 002 individuals with low percent predicted FEV_1_, 10 000 individuals with average percent predicted FEV_1_, and 5002 individuals with high percent predicted FEV_1_ from each of the heavy smoker and never smoker groups (figure 1). Within this dataset, 48 931 unrelated individuals passed quality control and were included in subsequent analyses.

We undertook genome-wide genotyping of 807 411 variants. After filtering, genome-wide imputation using the 1000 Genomes Project Phase 1 and UK10K Project reference panel resulted in 42 795 484 variants. Our final dataset for analysis, after excluding variants with information quality less than 0·5 or minor allele count less than three, comprised 28 509 962 imputed or genotyped variants in 48 931 unrelated individuals (table 1 and appendix pp 16 and 95).^23^

**Figure 2:**
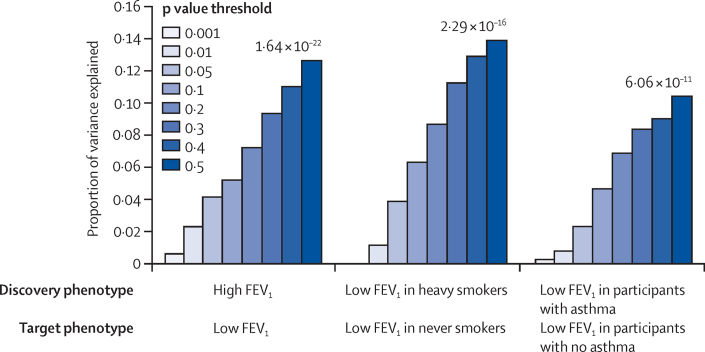
Polygenic component of low forced expiratory volume in 1 s and shared polygenic component of different phenotypes defined by forced expiratory volume in 1 s, smoking, and doctor diagnosis of asthma The p value in the target population shown above the bars is for the p value threshold <0·5. The sample sizes differed between the comparisons; details of these and the assumptions used in the analyses are described in the appendix (pp 18–20). FEV_1_=forced expiratory volume in 1 s.

Using independent discovery and target subpopulations to generate and test risk scores, we found that the association of low FEV_1_ versus average FEV_1_ with the risk score became stronger for increasingly liberal p value thresholds of association in the discovery population (p=6·24 × 10^−16^ for a p value threshold of 0·5). This finding suggests a polygenic component to low FEV_1_, in which many variants of individually small effect size contribute to the risk of low FEV_1_. We found substantial sharing of genetic causes across thousands of genetic variants between low FEV_1_ in heavy smokers and low FEV_1_ in never smokers (p=2·29 × 10^−16^; p value threshold <0·5; figure 2; appendix p 30). Similarly, we found substantially overlapping genetic causes for low FEV_1_ in participants reporting a history of doctor-diagnosed asthma and low FEV_1_ in those without asthma (p=6·06 × 10^−11^; p value threshold <0·5; figure 2; appendix p 31). Finally, overlapping genetic causes were shown for high FEV_1_ and low FEV_1_ (p=1·64 × 10^−22^; p value threshold <0·5; figure 2; appendix p 30).

**Figure 3:**
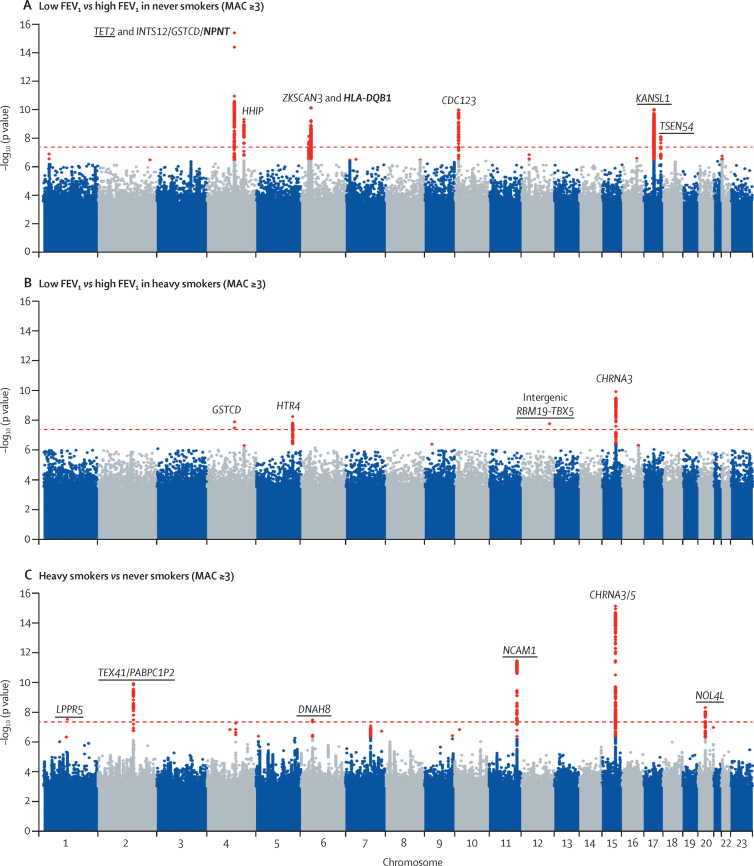
Manhattan plots for low versus high forced expiratory volume in 1 s in never smokers and heavy smokers and for heavy versus never smokers p values are from a Score test and have genomic control applied unless the MAC was less than 400 and Score test p<1·00 × 10^−6^, in which case p values are from a Firth test with no genomic control. Novel loci are underlined and novel signals at previously reported loci are shown in bold. The dashed red line shows the threshold for genome-wide significance (p<5 × 10^−8^). Variants with suggestive evidence of association (p<5 × 10^−7^) are coloured red. Quantile–quantile plots for these analyses are shown in the appendix (pp 113–116). FEV_1_=forced expiratory volume in 1 s. MAC=minor allele count.

In addition to detecting signals of association previously reported by studies of quantitative lung function (appendix p 32–37), in our case-control analysis of FEV_1_ extremes we identified six novel signals of association (p<5 × 10^−8^) with low FEV_1_ versus high FEV_1_ (table 2; figure 3; appendix pp 38–53, 96–98, 102). The sentinel SNPs at five of these six signals, in or near *TET2, NPNT, HLA-DQB1*/*HLA-DQA2, KANSL1,* and *TSEN54*, were common (MAF ≥5%) and showed a stronger association with low FEV_1_ in never smokers than heavy smokers. The sentinel SNP at an intergenic signal between *RBM19* and *TBX5* was a rare variant (MAF=0·13%) that showed strongest association with low FEV_1_ in heavy smokers. The lead SNPs at each of these loci showed association with COPD (table 2; appendix p 55). The 26 previously reported SNPs (associated with FEV_1_, FEV_1_:FVC ratio, or both)^5,7–9^ explained 2·33% of the variance of FEV_1_ in our data; adding in the SNPs representing our six novel signals of association with FEV_1_ extremes, we explained 3·63% of the variance of FEV_1_ (appendix p 17).

Although association with lung function at 4q24 is well established,^5,7^ we report two further independent signals of association at this locus (table 2; appendix pp 56–57, 99–101). The first (rs34712979) was localised to *NPNT* but independent of the previously reported signal, which spanned *INTS12, GSTCD*, and *NPNT* (appendix p 102). The second (rs2047409) was 552 kb from the signals at *INTS12, GSTCD*, and *NPNT* and was localised to *TET2*. The signal of association for rs34712979 was strongest in never smokers (p=9·62 × 10^−16^; table 2) and was weakly correlated (linkage disequilibrium *r*^2^=0·31) with another SNP in *NPNT*—rs6856422—which has also been identified as a novel secondary signal of association at 4q24 by an independent concurrent study of lung function in the general population.^35^ When rs6856422 was included as a covariate in our analysis, the signal for rs34712979 (p=9·62 × 10^−16^) was only slightly attenuated (p=4·66 × 10^−11^). The novel signal at *TET2* (rs2047409) also showed strongest association in never smokers (p=1·31 × 10^−8^). rs2047409 is separated from the previously reported association of rs10516526 with FEV_1_ (GSTCD)^5,7^ by a recombination hotspot and is statistically independent (rs10516526^7^ included as a covariate; rs2047409, p=9·8 × 10^−9^). *TET2* encodes tet methylcytosine dioxygenase 2, which has a role in myelopoiesis, and SNPs in *TET2* have shown association with height.^36^ *TET2* was differentially expressed during fetal lung development (appendix pp 58–59).

We detected a signal of association with FEV_1_ extremes within the HLA region on chromosome 6 that was correlated with a previously reported signal of association with asthma.^37^ The signal we report was strongest in never smokers (rs9274600, p=1·26 × 10^−10^; table 2; appendix p 102). With an imputed proxy (rs17843604) of the asthma-associated SNP rs9273349^37^—included as a covariate in the analysis of rs9274600—the signal for rs9274600 was attenuated (p=5·66 × 10^−4^), confirming that rs9274600 and rs9273359 are correlated. After exclusion of individuals with doctor-diagnosed asthma, the odds ratio for rs9274600 decreased from 1·18 (95% CI 1·11–1·25) to 1·14 (1·08–1·20), but remained significant (p=3·25 × 10^−6^; appendix p 103). This signal is independent of nearby signals reported for lung function,^5,7,8^ including rs7764819,^38^ which is 45 kb from rs9274600 (association for rs9274600 conditioned on rs7764819; p=6·71 × 10^−11^).

We identified a rare SNP that was associated with FEV_1_ extremes in heavy smokers only, after adjusting for pack-years of smoking (p=1·16 × 10^−8^; table 2). This intergenic SNP on chromosome 12 (chr12:114743533, MAF=0·13%) also showed weak evidence of association with smoking behaviour (p=6·12 × 10^−3^; appendix pp 60–62). We noted evidence for change in expression levels with increasing fetal lung age (p=0·04) for one or more probes after adjustment for multiple testing for the nearby gene *TBX5* (appendix pp 58–59).

We noted a broad signal of association (∼1·5 Mb) in an inversion locus at 17q21.31 (rs2532349, near *KANSL1*; appendix p 97). This signal was strongest in never smokers (rs2532349, p=1·66 × 10^−10^), but was also detected in heavy smokers (p=1·47 × 10^−5^). Genes in this locus, which include *MAPT* and *CRHR1*, have previously been associated with pulmonary fibrosis^39,40^ and inhaled corticosteroid response in asthma.^41^ SNP rs2532349 (and SNPs in strong linkage disequilibrium [*r*^2^>0·8]) was associated with mRNA expression levels of at least 15 genes in lung and blood (appendix pp 63–70). We identified differential expression for six genes at 17q21.31 during fetal lung development (and for four genes on different chromosomes regulated by *trans* eQTLs at 17q21.41; appendix pp 58–59). Relatively abundant novel transcripts (ie, compared with other transcripts detected) were identified by RNA sequencing in human bronchial epithelial cells for *WNT3* and *LRRC37A4P*; expression of both genes was associated with rs2532349 in lung and blood (appendix pp 104–109). The SNP rs2532349 (MAF=24%) was in linkage disequilibrium with the inversion (*r*^2^>0.9); the allele associated with low FEV_1_ was positively correlated with the inverted haplotype.^42,43^ The inversion locus contains structural variation resulting from three duplication events (150–300 kb).^42,43^ We imputed the nine common structural haplotypes (appendix pp 23–24)^42^ and found that the number of copies of the 150 kb region containing the 5′ end of *KANSL1* (the entire 150 kb duplication region found only in individuals who carry the inversion and a nested region of the 300 kb duplication region found only in individuals who do not carry the inversion) was associated with extremes of FEV_1_ (p=2·40 × 10^−6^; appendix p 71). The sentinel SNP rs2532349 lies within this region.

A second signal of association with FEV_1_ extremes on chromosome 17 (17q25.1) was within *TSEN54* and occurred only in never smokers (rs7218675; p=1·18 × 10^−8^; table 2). *TSEN54* encodes a subunit of the tRNA splicing endonuclease complex, and rs7218675 was associated with expression of *KIAA0195, TSEN54*, and *GRB2* in blood and expression of *GRB2* in lung tissue (appendix pp 63–66). GRB2 is a ligand of the epidermal growth factor receptor, which links signalling by epidermal growth factor with the MAPK/ERK signalling pathway, triggering cell proliferation. RNA sequencing in human bronchial epithelial cells identified a relatively abundant novel fusion transcript of *TSEN54* and *LLGL2* (appendix p 108).

To corroborate the new signals we identified in *TET2* and *TSEN54*, we present evidence of association with FEV_1_ in a previously reported study^8^ of 48 201 individuals (p=9·9× 10^−5^ and p=0·006, respectively; appendix pp 24, 82).

We identified a further 21 loci with suggestive (5 × 10^−8^<p<5 × 10^−7^) evidence of association with FEV_1_ extremes (appendix pp 72–73), including six rare variants with a minor allele count less than 400. These included signals in *CCDC91* and *RSRC1*, both of which showed genome-wide significant association with lung function in an independent concurrent study of lung function in the general population.^35^

By comparing heavy smokers and never smokers, we identified five novel regions of association with smoking behaviour and confirmed four previously reported loci (15q25, 7p14, *DBH*, and *BDNF*; table 2; appendix pp 32–37).^17–19^ The novel signals included rs4466874, in an intron of *NCAM1* (chromosome 11), and rs10193706, an intergenic SNP on chromosome 2 downstream of *TEX41* and upstream of *PABPC1P2* (table 2; appendix pp 110–112). Uncorrelated (*r*^2^<0·0001 with rs4466874) SNPs in *TTC12* and *ANKK1,* near to *NCAM1,* have also previously shown association with nicotine dependence (appendix pp 74–75).^44^ A proxy of rs10193706 on chromosome 2 (rs953246, *r*^2^=0·48) is a *trans* eQTL for *NCAM1* on chromosome 11 in brain tissue (appendix pp 76–77). Another proxy of rs10193706 on chromosome 2 (rs12622738, *r*^2^=0·86) is a *trans* eQTL in the substantia nigra for *WDR61* on chromosome 15, 300 kb from the established 15q25 smoking locus (appendix pp 76–77).

We also noted novel genome-wide significant signals of association with smoking behaviour in *NOL4L, LPPR5*, and *DNAH8* (table 2, appendix pp 110–112). A SNP in *C20orf203*, near to *NOL4L*, but independent of our sentinel variant, has previously been implicated in nicotine dependence.^45^ We identified secondary independent signals, which did not reach genome-wide significance, at three of the loci associated with smoking behaviour (appendix pp 56–57, 99–101),^46^ including a novel rare (MAF=0·09%) intergenic SNP near *NCAM1*. For novel signals for smoking behaviour, we did a meta-analysis of summary statistics from two previous, less powerful studies^17,19^ and found corroborative evidence for *NCAM1, TEX41/PABPC1P2*, and *NOL4L* (eg, for smoking initiation p=0·0003, p=0·017, and p=0·0006, respectively; appendix pp 24, 83–84). We identified a further eight loci with suggestive (5 × 10^−8^<p<5 × 10^−7^) evidence of association with smoking behaviour (appendix p 72–73), including *CHRNA4* at 20q13.33 (p=1·01 × 10^−7^).^47^

In a genome-wide gene–smoking interaction analysis, although common SNPs on chromosomes 6 and 19 showed suggestive SNP–smoking interactions (p<5 × 10^−7^; appendix p 78), no gene–smoking interactions were detected at genome-wide significance (p<5 × 10^−8^). Three of the six variants associated with FEV_1_ extremes (table 2) showed weak evidence of interaction with smoking (Bonferroni correction for six tests p=0·0083; appendix pp 60–62), including common variants at the *HLA-DQB1/HLA-DQA2* and *TSEN54* loci and the rare variant at the *RBM19/TBX5* locus. In a meta-analysis of the genome-wide association test statistics for low FEV_1_ versus high FEV_1_ across heavy and never smokers, motivated by our finding of shared genetic causes between heavy and never smokers and to increase the sample size, we identified an additional six novel genome-wide significant signals of association with FEV_1_ extremes. These included *CCDC91*, reported as a novel signal of association with lung function in the general population by a concurrent study,^35^ and *SLMAP*, for which there is corroborative evidence of association with lung function (appendix pp 79–80).^8^ Our pathway analysis identified a novel signal of enrichment of the histone subset of the chromatin packaging and remodelling process gene set, which was independently replicated in a concurrent GWAS of lung function in the general population (appendix p 81).^35^ Replication of a previously reported^8^ enrichment of the systemic lupus erythematosus pathway was also noted (appendix p 81).

## Discussion

We describe, to our knowledge, the first genetic association analyses in UK Biobank, targeting the genetic architecture of smoking behaviour and lung function phenotypes. By sampling from the extremes of the FEV_1_ and smoking phenotype distributions, we identified novel associations for FEV_1_ and smoking behaviour. We show genome-wide evidence for shared genetic causes of low FEV_1_ between heavy smokers and never smokers. Furthermore, our analyses suggest that smoking is only likely to interact with a small proportion of the genetic effects we have identified on lung function—that is, smoking and genetic effects generally act separately. We also show shared genetic causes of airflow obstruction between participants who reported doctor-diagnosed asthma and those who did not.

Two of our novel signals of association with smoking behaviour implicate *NCAM1*; one SNP lies within an intron of *NCAM1* and a second variant, located distantly on chromosome 2, is a *trans* eQTL for *NCAM1* in brain tissue (medulla)—ie, it is associated with the level of expression of *NCAM1*. This second SNP is also a *trans* eQTL in substantia nigra tissue for another gene called *WDR61,* which is close to the genes *CHRNA3* and *CHRNA5* at 15q25—a locus strongly associated with smoking behaviour.^17–19^ The substantia nigra plays an important part in reward and addiction,^48^ but little is known about *WDR61* other than that expression can be induced by mechanical strain in mesenchymal stem cells.^49^

We describe six new signals of association with FEV_1_ extremes, all of which were also associated with COPD using our definition based on spirometry. Five of these signals were most strongly associated with extremes of FEV_1_ (low *vs* high) in never smokers. The signal at 17q21.31 for extremes of FEV_1_ suggests a role for structural variation and epigenetic regulation in lung health. We found that the number of copies of the 5′ end of *KANSL1*—a gene disrupted by duplication events—is associated with FEV_1_ extremes. *KANSL1* encodes a protein that is a key component of the NSL1 (histone acetyltransferase) complex.^50^ The disruption of the gene gives rise to a novel truncated transcript,^42^ which encodes a protein missing a domain essential for key interactions with other proteins important for NSL1 function.^51^ Therefore, widespread effects on gene regulation through altered histone acetylation could underlie this association. Reduced expression of *KANSL1* causes a rare multisystem disorder,^52,53^ suggesting an essential role for *KANSL1* in epigenetic regulation. In a genome-wide pathway analysis, we identified the histone gene set, further implicating a role for epigenetic regulation in lung health.

We maximised the power of our study by sampling from the extremes of a large biobank. No other similar resources of a comparable size were available for replication studies. Nevertheless, the novel genome-wide significant signals of association with FEV_1_ extremes in *NPNT* and *KANSL1* in never smokers were also significantly associated in the independent set of heavy smokers. Furthermore, to corroborate the new signals we identified in *TET2* and *TSEN54*, we present evidence of association with FEV_1_ in a previously reported large study of FEV_1_ in the general population.^8^ However, the rare SNP on chromosome 12 for which we found association with extremes of FEV_1_ was exclusive to the recently released UK10K Project component of the imputation panel and has not yet been measured in suitably large studies. Our comparison of smokers and never smokers represents a powerful approach because of the restriction to heavy smokers rather than ever smokers. For novel signals for smoking behaviour, we present additional evidence of association with smoking behaviour for *NCAM1, TEX41/PABPC1P2*, and *NOL4L* in independent populations.^17,19^ Although these independent datasets have limited power, they provide corroboration of key genome-wide significant findings in UK BiLEVE.

One of the strengths of our study design was that the genotyping platform we used allowed for fine mapping of regions already known to contain genetic variants that affect lung function. For example, we were able to identify a novel signal in *NPNT* that was independent of the previously reported signal of association at this locus (spanning *GSTCD, INTS12*, and *NPNT*). The independent *NPNT* signal captured by the genotyped variant rs34712979 was not detected in previous or concurrent studies because it was neither directly genotyped nor imputed with sufficient quality; this finding highlights a further advantage of the UK BiLEVE and UK Biobank array design.

The design of this genotyping array combined the best features of existing genome-wide platforms targeting common SNPs (MAF ≥5%) and putative functional exome chip content, plus additional content to improve imputation of low-frequency variants (MAF 1–5%). In combination with a new large UK-specific imputation reference panel (UK10K Project), these features increase the potential to discover novel signals. In our study, more than 28·5 million variants were imputed; current large meta-analyses combining data from several studies with older arrays and using equivalent quality control filters after imputing to 1000 Genomes Project Phase 1 alone typically measure about 10·6 million variants.^35^ The genome-wide genotype data for these 50 008 individuals have been deposited in UK Biobank to be made available to other approved research projects across many disease areas. The UK BiLEVE array was used as a prototype for the array that is being used in the remaining roughly 450 000 UK Biobank participants. The UK Biobank array shares more than 95% of its content with the UK BiLEVE array. When genotyping of all UK Biobank participants is complete, UK Biobank will provide a unique resource for genome-wide studies of quantitative traits, nested case-control studies, and studies in which longitudinal outcomes can be studied.

Despite the strengths of using a large resource such as UK Biobank, this study has some limitations. In particular, there is a trade-off between obtaining the large sample sizes generally needed for genetic studies and the depth of phenotyping that is practicable in such large populations. Our spirometric definition of COPD was not based on bronchodilator reversibility testing, although we have shown previously that by limiting inclusion to individuals with GOLD stage 2+ spirometry, most of these individuals are likely to have COPD according to more rigorous criteria.^13^ Similarly, our definition of asthma was based on self-reporting of doctor-diagnosed disease. These limitations might have reduced our ability to identify some novel disease associations, although we were able to replicate many known associations using this approach.

In summary, we show the usefulness in sampling from the extremes of UK Biobank data to identify novel genetic signatures underlying phenotypes important in the development of airway disease and smoking behaviour. The ongoing genotyping, and further phenotyping, of the rest of the UK Biobank resource will facilitate further GWAS, which will undoubtedly improve our understanding of the genetic and molecular basis of common disease.

## Figures and Tables

**Table 1:** Absolute and percent predicted forced expiratory volume in 1 s in each subgroup in heavy and never smokers

	**Heavy smokers**			**Never smokers**		
	Number of individuals (n=24 457)	Absolute FEV_1_ (L)	Predicted FEV_1_ (%)	Number of individuals (n=24 474)	Absolute FEV_1_ (L)	Predicted FEV_1_ (%)
Low FEV_1_	9748	1·93 (0·55)	65·6% (11·8)	9745	2·05 (0·54)	69·3% (10·0)
Average FEV_1_	9803	2·68 (0·56)	90·6% (3·9)	9827	2·92 (0·57)	98·7% (1·3)
High FEV_1_	4906	3·49 (0·72)	118·0% (8·1)	4902	3·83 (0·73)	130·3% (8·3)

Data are mean (SD), unless otherwise specified. See appendix (pp 3–5) for details of sample selection. FEV_1_=forced expiratory volume in 1 s.

**Table 2:** Novel genome-wide significant signals of association with extremes of forced expiratory volume in 1 s or smoking behaviour

		**Locus**	**Non-coded/coded allele (minor allele)**	Imputation info score*	**Smoking status**	**MAF (MAC)**†	**OR (95% CI)**	**p value (genomic control corrected)**	**Association with COPD**‡		**Effect on FEV_1_**	
									OR (95% CI)	p value	Beta (SE)§	p value
**Extremes of FEV**_1_**: low *vs* high FEV**_1_												
Genome-wide significant in heavy smokers												
	Chr12:114743533	*RBM19/TBX5*	T/C (T)	0·737	Never	0·002 (60)	0·97 (0·57–1·67)	0·90	1·16 (0·54–2·51)	0·71	0·101 (0·118)	0·39
	..	..	..	..	Heavy	0·001 (39)	11·73 (5·03–27·32)	1·16 × 10^−8^	6·44 (2·89–14·37)	5·40 × 10^−6^	−0·728 (0·151)	1·31 × 10^−6^
Genome-wide significant in never smokers												
	rs34712979 ¶‖, Chr4:106819053	*NPNT*	G/A (A)	1·000	Never	0·268 (7842)	1·27 (1·20–1·34)	9·62 × 10^−16^	1·36 (1·27–1·46)	2·10 × 10^−18^	−0·087 (0·010)	2·27 × 10^−17^
	..	..	..	..	Heavy	0·261 (7636)	1·18 (1·11–1·25)	1·10 × 10^−8^	1·26 (1·18–1·34)	5·43 × 10^−13^	−0·056 (0·010)	4·22 × 10^−8^
	rs9274600¶, Chr6:32635592	*HLA-DQB1/HLA-DQA2*	A/G (G)	0·962	Never	0·472 (13 838)	1·18 (1·13–1·25)	1·26 × 10^−10^	1·24 (1·16–1·32)	1·95 × 10^−11^	−0·057 (0·009)	6·72 × 10^−10^
	..	..	..	..	Heavy	0·468 (13 719)	1·05 (1·00–1·10)	0·096	1·08 (1·02–1·14)	8·58 × 10^−3^	−0·019 (0·009)	0·037
	rs2532349, Chr17:44339473	*KANSL1*	A/G (G)	0·976	Never	0·242 (7088)	1·22 (1·15–1·29)	1·66 × 10^−10^	1·24 (1·16–1·34)	3·97 × 10^−9^	−0·063 (0·011)	3·22 × 10^−9^
	..	..	..	..	Heavy	0·233 (6832)	1·15 (1·08–1·21)	1·47 × 10^−5^	1·14 (1·07–1·22)	9·56 × 10^−5^	−0·050 (0·011)	3·64 × 10^−6^
	rs7218675, Chr17:73513185	*TSEN54*	C/A (C)	0·997	Never	0·291 (8538)	1·18 (1·11–1·25)	1·18 × 10^−8^	1·22 (1·14–1·31)	4·56 × 10^−9^	−0·052 (0·010)	1·94 × 10^−7^
	..	..	..	..	Heavy	0·290 (8503)	1·04 (0·98–1·09)	0·23	1·06 (1·00–1·13)	0·059	−0·017 (0·010)	0·080
	rs2047409, Chr4:106137033	*TET2*	G/A (G)	0·998	Never	0·345 (10 117)	1·17 (1·11–1·23)	1·31 × 10^−8^	1·17 (1·10–1·25)	1·64 × 10^−6^	−0·056 (0·009)	4·19 × 10^−9^
	..	..	..	..	Heavy	0·356 (10 440)	1·07 (1·02–1·13)	8·01 × 10^−3^	1·09 (1·03–1·16)	2·92 × 10^−3^	−0·023 (0·009)	0·014
**Smoking behaviour: heavy *vs* never smokers**												
rs4466874, Chr11:112861434		*NCAM1*	T/C (C)	0·998	NA	0·385 (37 709)	1·10 (1·07–1·13)	3·22 × 10^−12^	NA	NA	NA	NA
rs10193706, Chr2:146316319		*TEX41/ PABPC1P2*	A/C (A)	0·983	NA	0·473 (46 280)	1·09 (1·06–1·12)	1·10 × 10^−10^	NA	NA	NA	NA
rs143125561; rs57342388, Chr20:31162590		*NOL4L*	C/CACGG (CACGG)	0·983	NA	0·233 (22 820)	1·10 (1·07–1·13)	4·65 × 10^−9^	NA	NA	NA	NA
rs61784651‖, Chr1:99445471		*LPPR5*	C/T (T)	1·000	NA	0·170 (16 609)	1·10 (1·07–1·14)	2·89 × 10^−8^	NA	NA	NA	NA
rs10807199, Chr6:38901867		*DNAH8*	C/T (T)	1·000	NA	0·473 (46 286)	1·08 (1·05–1·11)	3·17 × 10^−8^	NA	NA	NA	NA

For variants that showed association with extremes of FEV_1_ in either heavy smokers or never smokers, the results from both the never smokers and heavy smokers are presented. For variants that had genome-wide significant evidence of association for smoking behaviour (and not for extremes of FEV_1_), association with COPD and effect on FEV_1_ were not assessed. Chromosome and position relate to National Center for Biotechnology Information build 37 (hg19). ..=as above. COPD=chronic obstructive pulmonary disease. FEV_1_=forced expiratory volume in 1 s. MAC=minor allele count. MAF=minor allele frequency. NA=not applicable. OR=odds ratio. SE=standard error.

* Indication of certainty of imputation for this variant; an imputation info score of 1 suggests a variant imputed with the highest certainty or a directly genotyped variant.

† In samples included in the comparison.

‡ Analysis of Global Initiative for Chronic Obstructive Lung Disease stage 2+ COPD cases versus controls (heavy smokers: 5803 cases *vs* 4661 controls; never smokers: 3761 cases *vs* 4792 controls).

§ Effect on FEV_1_ beta values are effect-size estimates on an inverse-normal transformed scale after adjustments for age, age^2^, sex, height, and ancestry principal components (appendix p 21).

¶ Novel signals of association within previously reported loci.

‖ Directly genotyped.
