# Corrections

**DOI:** 10.1016/S2213-2600(15)00512-3

**Published:** 2016-01

*Wain LV, Shrine N, Miller S, et al. Novel insights into the genetics of smoking behaviour, lung function, and chronic obstructive pulmonary disease (UK BiLEVE): a genetic association study in UK Biobank.* Lancet Respir Med *2015; **3:** 769–81*—In this Article, in the upper section of Table 2 (Extremes of FEV_1_), the p values and 95% CIs for signals of association are genomic control corrected for the result in the smoking status stratum for which the signal was most highly significant, and uncorrected for the alternate smoking status stratum. This has been clarified in the column header and added to the footnote of table 2, and clarified in the header of Supplementary Table 18 of the appendix. These corrections have been made to the online version as of Dec 30, 2015.

