# Supplementary material for: Novel insights into the genetics of smoking behaviour, lung function, and chronic obstructive pulmonary disease (UK BiLEVE): a genetic association study in UK Biobank
Source: Lancet Respir Med. 2015 Oct;3(10):769–81. doi: 10.1016/S2213-2600(15)00283-0 (PMC4593935; doi:10.1016/S2213-2600(15)00283-0)
Supplement: Supplementary appendix [file mmc1.pdf]

THE LANCET Respiratory Medicine

Supplementary appendix

This appendix formed part of the original submission and has been peer reviewed. We post it as supplied by the authors.

Supplement to: Wain LV, Shrine N, Miller S, et al. Novel insights into the genetics of smoking behaviour, lung function, and chronic obstructive pulmonary disease (UK BiLEVE): a genetic association study in UK Biobank. *Lancet Respir Med* 2015; published online Sept 28. http://dx.doi.org/10.1016/S2213-2600(15)00283-0.

Supplementary Appendix

Novel insights into the genetics of smoking behaviour, lung function, and chronic obstructive pulmonary disease (UK BiLEVE): a genetic association study in UK Biobank

Louise V Wain[1]* PhD, Nick Shrine[1]* PhD, Suzanne Miller[2] PhD, Victoria Jackson[1] MSc, Ioanna Ntalla[1] PhD, María Soler Artigas[1] PhD, Charlotte K Billington[2] PhD, Abdul Kader Kheirallah[2] BSc, Richard Allen[1] MSc, James P Cook[1] PhD, Kelly Probert[2] BSc, Ma'en Obeidat[3] PhD, Yohan Bossé[4] PhD, Ke Hao[5,6,7] ScD, Prof. Dirkje S Postma[8] PhD, Peter D Paré[3] MD, Adaikalavan Ramasamy[9,10,11] DPhil, UK Brain Expression Consortium (UKBEC) [12], Reedik Mägi[13] PhD, Evelin Mihailov[13] MSc, Eva Reinmaa[13] MSc, Erik Melén[14] MD, Jared O'Connell[15,16] DPhil, Eleni Frangou[15,17] MSc(Res), Olivier Delaneau[15,18] PhD, OxGSK Consortium[12], Colin Freeman[16] PhD, Desislava Petkova[16] PhD, Prof. Mark McCarthy[19,16] MD, Ian Sayers[2] PhD, Prof. Panos Deloukas[20,21] PhD, Prof. Richard Hubbard[22] MD, Ian Pavord[23] FMedSci, Anna L Hansell[24,25] MB BChir, Prof. Neil C Thomson[26] MD, Eleftheria Zeggini[27] PhD, Prof Andrew P Morris[28] PhD, Prof. Jonathan Marchini[15,16] DPhil, Prof. David P Strachan[29]* MD, Prof. Martin D Tobin[1, 30]* PhD, Prof. Ian P Hall[2]* MD
*These authors contributed equally
1. Department of Health Sciences, University of Leicester, Leicester LE1 7RH, UK
2. Division of Respiratory Medicine, University of Nottingham, Queen's Medical Centre, Nottingham NG7 2UH, UK
3. University of British Columbia Centre for Heart Lung Innovation, St. Paul's Hospital, Vancouver, BC, Canada
4. Institut universitaire de cardiologie et de pneumologie de Québec, Department of Molecular Medicine, Laval University, Québec, Canada
5. Department of Genetics and Genomic Sciences, Icahn School of Medicine at Mount Sinai, New York, NY, USA
6. Department of Respiratory Medicine, Shanghai Tenth People's Hospital, Tongji University, Shanghai, China
7. Icahn Institute of Genomics and Multiscale Biology, Icahn School of Medicine at Mount Sinai, New York, NY, USA
8. University of Groningen, University Medical Center Groningen, Department Pulmonary Medicine and Tuberculosis, Groningen, The Netherlands
9. Department of Molecular Neuroscience, UCL Institute of Neurology, London WC1N 3BG, UK
10. Department of Medical & Molecular Genetics, King's College London SE1 9RT, UK
11. Jenner Institute, University of Oxford, Oxford OX3 7DQ, UK
12. List of members and affiliations appears at the end of the paper
13. Estonian Genome Center, University of Tartu, Tartu, 51010, Estonia
14. Institute of Environmental Medicine, Karolinska Institutet and Sachs' Children's Hospital, Stockholm, Sweden
15 Department of Statistics, University of Oxford, UK
16. Wellcome Trust Centre for Human Genetics, University of Oxford, Oxford OX3 7BN, UK
17. Centre for Statistics in Medicine Nuffield Department of Orthopaedics, Rheumatology and Musculoskeletal Sciences, University of Oxford, UK
18. Department of Genetic Medicine and Development, University of Geneva Medical School, Geneva, Switzerland
19. Oxford Centre for Diabetes, Endocrinology & Metabolism, University of Oxford, Oxford, UK
20. William Harvey Research Institute, Barts and The London School of Medicine and Dentistry, Queen Mary University London, London, UK
21. Princess Al-Jawhara Al-Brahim Centre of Excellence in Research of Hereditary Disorders (PACER-HD), King Abdulaziz University, Jeddah 21589, Saudi Arabia
22. Faculty of Medicine and Health Sciences, School of Medicine, University of Nottingham, Nottingham, UK
23. Respiratory Medicine, University of Oxford, Oxford, UK
24. UK Small Area Health Statistics Unit, MRC-PHE Centre for Environment and Health, School of Public Health, Imperial College London, UK
25. Imperial College Healthcare NHS Trust, St Mary's Hospital, Paddington, London, UK
26. Institute of Infection, Immunity & Inflammation, University of Glasgow, UK
27. Wellcome Trust Sanger Institute, Hinxton, Cambridgeshire, UK
28. Department of Biostatistics, University of Liverpool, Liverpool, UK
29. Population Health Research Institute, St George's, University of London, London SW17 0RE, UK
30. National Institute for Health Research (NIHR) Leicester Respiratory Biomedical Research Unit, Glenfield Hospital, Leicester, UK

Table of Contents

Supplementary Methods 3

Description of selection of 50,008 samples from UK Biobank (n=502,682) 3
Description of array design and genotyping process 5
Description of post-genotyping quality control (QC) steps undertaken for samples and variants 8
Description of genotype imputation using 1000 Genomes Project and UK10K Project reference panels 15
Description of association testing for autosomal and X, Y and mitochondrial variants 17
Proportion of variance explained 17
Genome-wide analysis of SNP x smoking interaction 17
Association with GOLD Stage 2+ COPD for novel signals of association with extremes of FEV_1 18
Analysis of polygenic architecture of diseases and health-related traits 18
Association with self-reported/doctor diagnosed asthma of loci previously reported for genome-wide significant association with asthma 21
Effect on quantitative FEV_1 for novel signals of association with extremes of FEV_1 21
Analysis of expression data from lung, blood and brain tissues to identify if our novel signals affect gene expression (eQTL) 21
Analysis of differential expression of candidate genes in the lungs of individuals with and without COPD 22
Analysis of differential expression of candidate genes in the developing foetal lung 22
Messenger RNA sequencing in human bronchial epithelial cells (HBECs) to identify novel transcripts of genes at novel loci associated with the extremes of FEV_1 22
Pathway analysis using MAGENTA 23
Stepwise conditional analysis to identify additional independent signals at the novel loci 23
Imputation and association testing of structural variation haplotypes in the inversion locus at chromosome 17q21.31 (*KANSL1*) 23
Corroborative evidence supporting loci with genome-wide significant evidence of association with extremes of FEV_1 24
Corroborative evidence supporting loci with genome-wide significant evidence of association with smoking behaviour (heavy smokers vs never smokers) 24
Power Calculations 25
Analysis to identify whether variants with a high functional score explain the signal. 26
Gene-based analysis of rare and low-frequency variants (MAF < 5%) using SKAT-O 26
Analysis of the effect of geographical location on novel loci 26

Supplementary Tables 29

Supplementary Figures 95

UK Brain Expression Consortium 124

OxGSK Consortium 125

Appendix 1: 127

UK Biobank Unique Identifiers (UDIs) used to select individuals for UK BiLEVE. 127
OxGSK Consortium information 128

References 132

Supplementary Methods

Description of selection of 50,008 samples from UK Biobank (n=502,682)

This section describes how UK Biobank samples were selected for inclusion in this study (UK Biobank Lung Exome Variant Evaluation (UK BiLEVE) consortium study).

Sampling frame

UK Biobank contained information for 502,682 individuals, of which 472,858 were of white European ancestry (based on UK Biobank Unique Data Identifier (UDI) 21000). A total of 426,797 individuals of white European ancestry had at least 2 Forced Expiratory Volume in 1 second (FEV_1) (UDI 3063) and forced volume vital capacity (FVC) (UDI 3062) measures and had complete information for spirometry method used, age, sex and standing height (UDIs 23, 21003, 31 and 50, respectively). Spirometry was undertaken using a Vitalograph Pneumotrac 6800. The participant was asked to record two to three blows (lasting for at least 6 seconds) within a period of about 6 minutes. The reproducibility of the first two blows was compared and, if acceptable (defined as a <5% difference in FVC and FEV_1), a third blow was not required. A total of 275,939 participants had spirometry measures which met ERS/ATS guidelines[1] and these individuals were taken forward as the sampling frame for further selection. Post-bronchodilator spirometry was not available for any participants and medication was not withheld prior to spirometry being undertaken.

Never smokers were defined as individuals who had not smoked tobacco in the past and did not currently smoke tobacco. Ever smokers were defined as individuals who currently smoked cigarettes most days or occasionally, or who had smoked cigarettes in the past on most days or occasionally, or who had tried smoking once or twice. Current cigar/pipe smokers who smoked most days and previously smoked cigarettes were also designated as ever smokers. A pack years variable was defined for all ever smokers as:

$$\left(\frac{number\ of\ cigarettes\ per\ day}{20}\right) \times (age\ stopped\ smoking - age\ started\ smoking)$$

For individuals who gave up smoking for more than 6 months, pack years was defined as:

$$\left(\frac{number\ of\ cigarettes\ per\ day}{20}\right) \times (age\ stopped\ smoking - age\ started\ smoking - 0.5)$$

A percentage of life span smoking variable was defined as:

$$\left(\frac{number\ of\ cigarettes\ per\ day}{20}\right) \times \left(\frac{age\ stopped\ smoking - age\ started\ smoking}{age\ at\ recruitment - 16}\right)$$

For individuals who gave up smoking for more than 6 months, percentage of life span smoking was defined as:

$$\left(\frac{number\ of\ cigarettes\ per\ day}{20}\right) \times \left(\frac{age\ stopped\ smoking - age\ started\ smoking - 0.5}{age\ at\ recruitment - 16}\right)$$

For current smokers, pack years variables were calculated using age at recruitment in place of age stopped smoking. Heavy smokers were defined as individuals with a percentage of life span smoking ≥ 42% (equivalent to a minimum pack years of 10 in the youngest participants). See Appendix 1 for all UDIs for smoking behaviour.

Within the 275,939 European ancestry individuals with 2 or more FEV_1 and FVC measures which met ERS/ATS guidelines and who had non-missing information for spirometry method, age, sex and standing height, 105,281 were never smokers and 46,763 were heavy smokers. After exclusion of 14 individuals who had outlying FEV_1 after adjusting for sex, age, age^2, height and $height^2$, 105,272 never smokers and 46,758 heavy smokers remained.

Healthy never smokers were selected from the never smokers by excluding individuals who indicated that they had experienced wheeze, or reported any of the following respiratory conditions: asthma; chronic obstructive pulmonary disease (COPD); emphysema; chronic bronchitis; bronchiectasis; interstitial lung disease; asbestosis; pulmonary fibrosis; fibrosing/unspecified alveolitis; respiratory failure; pleurisy; spontaneous/recurrent pneumothorax; other respiratory problems (or did not know or declined to answer, according to UDIs 2316, 6152 or 20002). A subset of 81,719 healthy never smokers were used in the calculation of predictive values (below).

Allocation to lung function subgroups

Individuals were grouped into 58 age-sex bands (29 age bands per sex; ages 39, 40 and 41 were grouped into one band and ages 69, 70 and 72 were grouped into one band with ages 42 to 68 each forming a separate band). Predictive values to calculate percent predicted FEV_1 were calculated within each age-sex band in healthy never smokers only by linear regression with FEV_1 as the response variable and standing height as the only covariate with the following equation:

$$\text{predicted FEV}_1 = \beta_{0(\text{age}-\text{sex band})} + \beta_{1(\text{age}-\text{sex band})}\text{height}$$

Percent predicted FEV_1 was then calculated for the ith individual within each age-sex band:

$$\%\ \text{predicted FEV}_{1\ i} = \frac{\text{FEV}_{1i}}{\text{predicted FEV}_{1\ i}} \times 100$$

Our study design specified the selection of 10,000 individuals with low percent predicted FEV_1, 10,000 individuals with average percent predicted FEV_1 and 5,000 individuals with high percent predicted FEV_1 from each of the heavy smoker and never smoker groups (50,000 individuals in total). Sampling was undertaken such that equal numbers of males and females were selected in total and the numbers of individuals selected from each age-sex band were proportional to the number of individuals in the band being sampled from.

For heavy smokers and never smokers separately, individuals were ranked to define each FEV_1 subgroup (high, low and average) within each age-sex band according to their percent predicted FEV_1 such that the 5,000 individuals with the highest percent predicted FEV_1 were selected for the high FEV_1 subgroup and the 10,000 individuals with the lowest percent predicted FEV_1 were selected for the low FEV_1 subgroup. For the average FEV_1 subgroup, the median percent predicted FEV_1 within each age-sex band was calculated and individuals were ranked according to the distance of their percent predicted FEV_1 to the median (the individual with percent predicted FEV_1 closest to the median was therefore ranked 1). 10,000 individuals were then selected for the average FEV_1 subgroup. Where individuals had the same percent predicted FEV_1, they were ranked in a random order.

An extra 5% of individuals were also selected for each FEV_1 subgroup and age-sex band to use as reserves. A total of 50,008 individuals were selected (additional 8 selected to complete plates).

Provision of sample IDs to UK Biobank for DNA extraction

The 50,008 selected individuals were randomised (using a random number generator in R) and sample IDs were provided to UK Biobank who undertook DNA extraction (described below) in the order in which the sample IDs were listed. For samples which failed DNA extraction quality control steps, we selected a replacement from the reserve list for the same lung function subgroup and age-sex band as the failed sample. This list of reserve sample IDs was randomised and provided to UK Biobank such that DNA extraction and genotyping of these replacement samples (after randomisation) was undertaken last.

Sample descriptives

Supplementary Methods Figure 1 shows the distribution of mean % predicted FEV_1 by age for each FEV_1 subgroup and in heavy and never smokers separately.

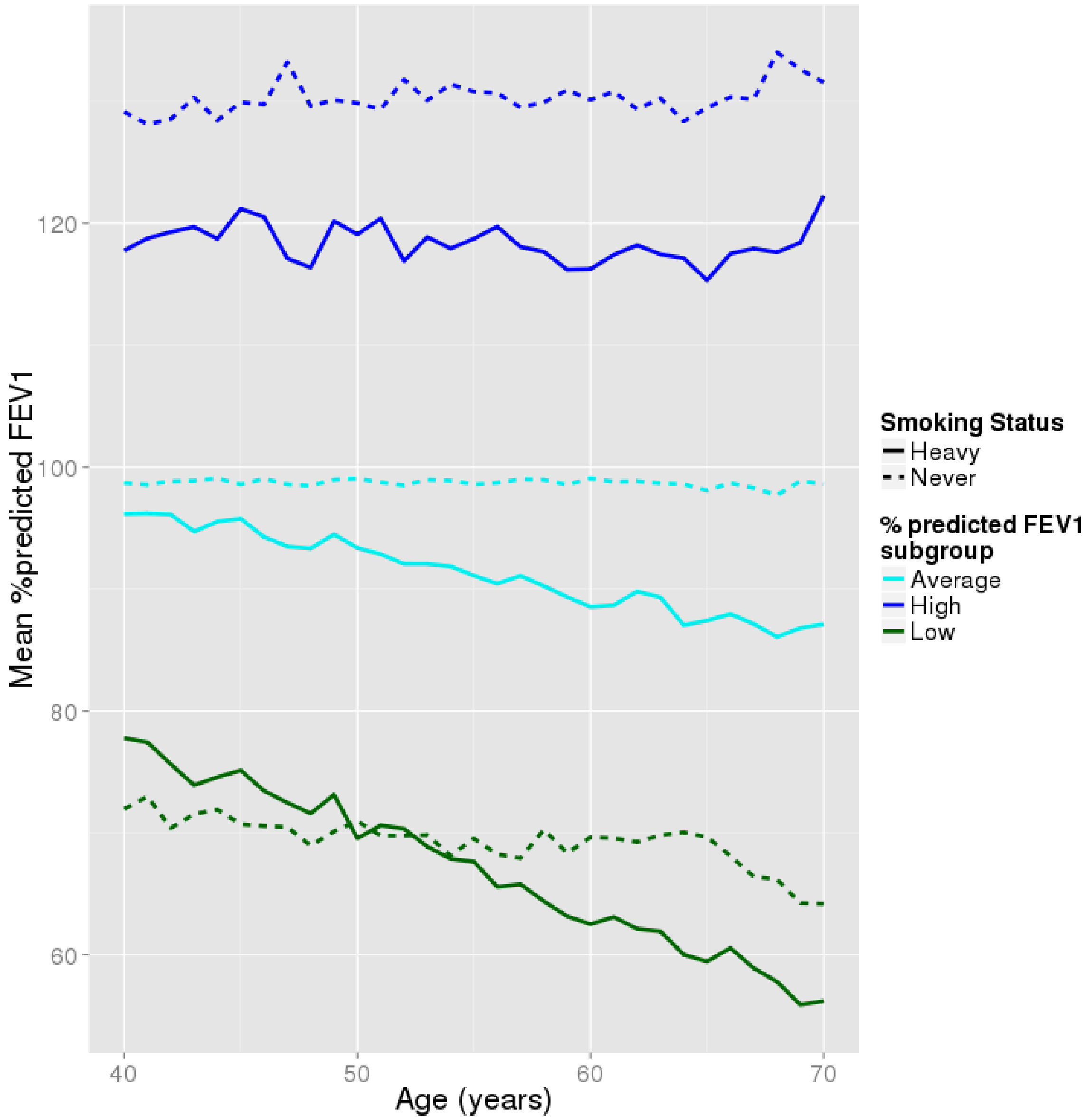

Supplementary Methods Figure 1: Distributions of mean % predicted FEV_1 by age in each FEV_1 group in heavy and never smokers separately.

Description of array design and genotyping process

The Affymetrix Axiom® array used for genotyping the UK BiLEVE samples was an early version of the UK Biobank Axiom® array which has subsequently been made publicly available (and used to genotype the rest of UK Biobank). Details of the final version of the UK Biobank Axiom® array, are available at http://media.affymetrix.com/support/technical/brochures/uk_axiom_biobank_contentsummary_brochure.pdf?cmpid=2014070005.

The UK BiLEVE array was designed to i) measure rare functional variation (akin to the aims of commercially available "exome chip" arrays), ii) provide a framework for optimal imputation of variants that are common (minor allele frequency (MAF) > 5%) or low frequency (MAF 1 to 5%) in the European population, and iii) optimise coverage of genes and genomic regions with established or putative roles in lung health and disease. As the UK BiLEVE array design was to form the basis for the UK Biobank array, additional categories of variants were included which were of potential relevance to a broad range of phenotypes. The UK BiLEVE array and the UK Biobank Axiom arrays have > 95% identical content.

Affymetrix Axiom® technology is based on "features"; a feature is the smallest unit of space on the array. Typical AT or GC variants require 4 features, other variants require 2 features and some previously validated variants require 1 feature.

Array design: Genome-wide coverage for imputation

A key objective for the UK BiLEVE array was to achieve high imputation accuracy in the 1% to 5% MAF range. Variants were selected from Affymetrix databases using a custom algorithm. A total of 246,055 variants in the 5% to 50% were selected from the 1000 Genomes CEU population. This set was boosted to improve imputation in the UK population and in the 1% to 5% MAF range by the addition of 102,514 variants with MAF 5% to 50% from the EUR population (union of CEU, GBR, FIN, IBS and TSI populations) and a further 293,050 variants with MAF 1% to 5% in the EUR population. These booster variants were polymorphic in CEU and GBR populations.

Array design: Rare functional variation

Approximately 130,000 rare coding variants from two sources were included on the array; the exome chip project (http://genome.sph.umich.edu/wiki/Exome_Chip_Design) and the Exome Aggregation Consortium (ExAC) (http://exac.broadinstitute.org/). In brief, the exome chip project developed a design for an array based on exome sequencing data from > 12,000 individuals of multiple ancestries (predominantly European). Allele frequency information collected by the UK Exome chip consortium, and from ExAC European exome sequencing data and UK10K non-Finnish exome sequencing data informed selection of variants expected to be polymorphic in the 500,000 individuals in UK Biobank.

Rare coding variants were selected according to estimated minor allele frequencies (EMAFs) as follows:

- All protein truncating variants (PTV, e.g. premature stop, frameshift, loss of start) with EMAF > 0.0002
- PTV variants with 0.00005 < EMAF < 0.0002 which require 1 or 2 features (see above)
- Additional PTV variants present in the ExAC exomes with EMAF > 0.0002

In addition, 21,000 rare variants in cancer and cardiac disease predisposition genes, as well as other disorders relevant to lung function were selected from HGMD (Human Gene Mutation database).

Array design: Respiratory content

Additional content was added to the design to optimise coverage of variants and genomic regions with known or putative associations with lung function.

Lung function associated variants included in the design were:

- 26 top variants previously reported as being associated with lung function[2-5] plus two tag variants ($r^2 > 0.9$ where possible) each.
- Approximately 390 variants representing potentially interesting regions which showed evidence of nominal significance for association with lung function[4], plus 1 tag variant ($r^2 > 0.9$) where available. In brief, all variants with $P < 10^{-4}$ for either FEV_1 or FEV_1/FVC and which were defined as independent ($r^2 < 0.5$ with other variants with $P < 10^{-4}$) were extracted from the genome-wide meta-analysis results (www.GWAScentral.org, identifier: HGVST946).
- Variant rs9316500 associated with lung function decline (not GW-significant)[6] plus one tag variant.
- 20 exonic variants with $P < 10^{-3}$ for association with resistance to smoking related airflow obstruction[7] plus 2 tags per variant.
- 92 novel putatively functional variants identified in a whole exome sequencing experiment of 100 individuals with resistance to smoking related airflow obstruction[7] (earlier version of the analysis than that published).
- 982 exonic variants identified in a whole exome sequencing experiment of 100 individuals with resistance to smoking related airflow obstruction[7] and which lie within the 26 lung function-associated loci[2-5].
- 58 variants showing nominal evidence of association in an unpublished study of longitudinal lung function.

COPD associated variants included explicitly in the design were:

- Top variants from Wilk et al COPD GWAS[8], a variant in *MMP12* which showed evidence of association with COPD in a candidate gene study[9] and variants with $P < 5 \times 10^{-4}$ evidence for association with lung function in a set of COPD candidate genes (*SERPINA1*, *MACROD2*, *ABCC1*, *CNTN5* and *PDE4D*[3, 10]), plus one tag variant per variant.
- Approximately 390 variants representing potentially interesting regions which showed nominal significance for association with COPD[8]. In brief, all variants with $P < 10^{-4}$ for association with COPD and which were defined as independent ($r^2 < 0.5$ with other variants with $P < 10^{-4}$) were extracted from the full 2.5million publicly available results.
- 16 variants showing suggestive evidence of association with COPD in a pooled case-control analysis of re-sequencing data from the 26 lung function regions, plus 1 tag variant per variant (unpublished).
- 16 variants in *SERPINA1* including the Z and S alleles and all variants that exist in the OMIM database in this gene as being "Clinically associated”.

Asthma associated variants included explicitly in the design were:

- 63 variants listed for asthma phenotypes in the GWAS catalog as downloaded on 23rd January 2013 plus one tag variant per variant.
- 111 variants representing potentially interesting regions which showed evidence of nominal significance for association with severe asthma[11]. In brief, all variants with $P < 10^{-4}$ for association with severe asthma and which were defined as independent ($r^2 < 0.5$ with other variants with $P < 10^{-4}$) were extracted from the full 2.5million imputed database.

Smoking, idiopathic pulmonary fibrosis (IPF) or lung cancer associated variants included explicitly in the design were:

- 21 variants with genome-wide significant ($P < 5 \times 10^{-8}$) evidence of association with cigarettes smoked per day, smoking cessation and smoking initiation[12-14] plus 2 tag variants per variant.
- Variants with genome-wide significant evidence of association with IPF[15, 16] in the *MUC5B* promoter and *TERT*, plus one tag variant per variant.
- Four variants associated with lung cancer[17].

Regions showing robust or putative association with lung function and/or disease were highlighted for inclusion of additional content to boost imputation coverage and quality. These regions were:

- 26 regions associated with lung function[2-5], defined based on P values and linkage disequilibrium (LD) (variants with $-log_{10}(P - value) > 2.5$ and not further from 50kb away from the next variant were selected, including any gene intersecting with the region or the nearest gene, if the region did not include any, ±10kb).
- Chromosome 15q25 region which shows strong association with smoking behaviour [12-14], defined as chr15: 78720518-79113773 (build 37).
- Six additional regions associated with smoking behaviour, defined based on region of association illustrated in published region plots[12-14].
- Three regions ±10kb of three genes associated with IPF (*TERT*, *MUC5B* and *TERC*)[15, 16, 18]

Summary of final array content

Of the 808,370 variants targeted in the design, 802,283 were able to be assayed directly by at least 1 probe on the Axiom® UK BiLEVE genotyping array. A tag variant was assayed for 5,340 variants that could not be directly measured, with 134 tag variants being used for more than 1 target variant and 951 tag variants also being a target variant, giving a total of 806,626 unique variants. An additional 785 variants included by Affymetrix for quality control purposes, gave a total of 807,411 variants assayed by the array. 781,732 variants were targeted by a single probe, with 25,679 targeted by 2 probes to increase the chance of successful genotyping, giving a total of 833,090 probes on the array.

Genotyping

DNA extraction was undertaken at the UK Biobank laboratories (http://www.ukbiobank.ac.uk/wp-content/uploads/2014/04/DNA-Extraction-at-UK-Biobank-October-2014.pdf). 850ul buffy coat from 9ml of whole blood was extracted on a custom TECAN Freedom EVO® 200 platform using Promega Maxwell® 16 Blood DNA Purification Kit (AS1010) (modified to optimise DNA yield from a large volume of buffy coat, including additional lysis and wash buffer and an additional pass through the extraction process). DNA concentration and quality was assessed via 260/280 using a Trinean DropSense® 96. DNA concentration was required to be > 10ng/ul for > 80% of samples on a plate and purity as measured by 260/280 was required to be between 1.8 and 2.2 for > 80% of samples on the plate. Samples were shipped on dry ice for genotyping.
Samples were shipped to Affymetrix, Santa Clara, CA, USA for genotyping. Genotype calling was undertaken using Affymetrix Power Tools v1.15.1 (Axiom® GT1 algorithm) in 11 batches of 4,800 samples comprised of UK BiLEVE samples and Affymetrix control samples (numbers shown in Supplementary Methods Table 1).

	Genotyping batch										
	**b1**	**b2**	**b3**	**b4**	**b5**	**b6**	**b7**	**b8**	**b9**	**b10**	**b11**
**UK BiLEVE samples**	4,598	4,606	4,589	4,603	4,600	4,573	4,596	4,597	4,595	4,600	4,604
**Affymetrix Controls**	202	194	211	197	200	227	204	203	205	200	205
**Total**	4,800	4,800	4,800	4,800	4,800	4,800	4,800	4,800	4,800	4,800	4,809

Supplementary Methods Table 1: Numbers of UK BiLEVE samples and Affymetrix control samples in each genotyping batch

Genotyping was undertaken in the batches which comprised of 50 plates. Variants which had a MAC < 6 in any batch were recalled in each individual plate in that batch as this was shown to improve calling for very rare

variants (unpublished data comparing genotype calls with re-sequencing data from non UK BiLEVE samples, Affymetrix).

Description of post-genotyping quality control (QC) steps undertaken for samples and variants

Variants were excluded prior to sample QC if they failed the basic Affymetrix genotyping quality metrics indicating poor genotype clustering (cluster QC). This included exclusion of variants for which more than three genotype clusters were observed (indicating an off-target measurement), for which the call rate was less than 95% or for which there was failure of one of three cluster quality metrics (Fisher's linear discriminant (FLD), Heterozygous cluster strength offset (HetSO), Homozygote Ratio Offset (HomRO)) defined in the Affymetrix Axiom® Genotyping Solution Data Analysis Guide (http://media.affymetrix.com/support/downloads/manuals/axiom_genotyping_solution_analysis_guide.pdf). Where a variant was assayed by 2 probes the genotypes from the probe with the highest call rate were used.
A total of 50,561 UK BiLEVE samples were genotyped. Samples were excluded sequentially from the analysis according to each of the following criteria (n indicates the number of samples excluded for each step) (Supplementary Methods Table 2):

1. **Poor DNA quality** – Indicated by Affymetrix's dish QC (dQC) metric. Samples were excluded if dQC < 0.82. (n=100)
2. **Call rate** – Samples with call rate < 97% were excluded by Affymetrix in an initial round of genotype clustering. The batches were then re-clustered without these samples. (n=31)
3. **Sex mismatch** – Samples were excluded if the sex inferred from X chromosome genotypes did not match submitted sex (see below for method). (n=125)
4. **Call rate** - Samples with a call rate < 95% after the second round of clustering were excluded. (n=1)
5. **Outlying heterozygosity** (high or low, indicative of a contaminated sample) – Samples with heterozygosity which was three standard deviations (SD) from the mean heterozygosity of all samples were excluded (see below for method). (n=333)
6. **Unintended duplicates** – Samples which share > 98% of alleles identical by descent (IBD) were consistent with either being duplicated samples (with different IDs) or identical twins. Where the duplication could be resolved (e.g. where we could identify which sample of the pair had the correct ID, or they were likely to be twins based on other information) then only 1 sample of the pair was excluded, otherwise both samples were excluded. (n=17)
7. **Intended duplicates** –The sample with the lowest genotyping call rate from each pair of intended duplicates was removed. (n=481)
8. **Principal Components Analysis (PCA) outliers** – Ancestry informative principal components (PCs) were derived from variant genotypes (see detailed methods below). Samples with a score for any of the first 10 principal components that was outside 10 SD from the mean were excluded. (n=104)
9. **Withdrawn consent** – One individual withdrew consent from further study after steps 1 to 8 above had been completed. This sample was excluded from all subsequent steps. (n=1)
10. **Related individuals** (see detailed methods below) – For any pair of samples which shared more than 20% of alleles IBD, the sample with the lowest call rate was excluded. Where more than 2 samples were mutually related, examination of the relationships between the samples was studied to identify which sample(s) were excluded. (n=515)

Details of each step are given below. A total of 48,943 samples remained for subsequent analysis.

	**Removed**	**Remaining**
**No filters**	0	50,561
**DNA quality (dQC)**	10	50,551
**Initial clustering CR<97%**	31	50,520
**Sex mismatch**	125	50,395
**Final clustering CR<95%**	1	50,394
**Heterozygosity outlier**	333	50,061
**Unintended duplicates**	17	50,044
**Intended duplicates**	481	49,563
**PCA outliers**	104	49,459
**Withdrawn participant**	1	49,458
**Related individuals**	515	48,943

Supplementary Methods Table 2: Sample exclusions

Sample QC: Sex mismatches

Two methods were used to identify discrepancies between the sex provided by UK Biobank and the sex inferred from the genotype data. Firstly, a scatterplot of the ratio of the mean X chromosome and Y chromosome probe

intensities (XY ratio) against X chromosome heterozygosity rate (X het rate) was plotted. Secondly, using PLINK v1.07[19], the chromosome X inbreeding (homozygosity) estimate, F, was used to classify samples as male ($F > 0.8$), female ($F < 0.2$) or unknown/ambiguous ($0.2 < F < 0.8$). A total of 82 samples were reported as showing a sex mismatch using both methods and an additional 28 samples were reported using the PLINK approach (Supplementary Methods Figure 2). Seventeen of the samples reported by PLINK only, and one sample reported by both methods were subsequently found to be heterozygosity outliers and were excluded. Thirty-one of the samples detected by both methods had an XY ratio indicative of being male and an X het rate indicative of being female suggesting that these samples had two copies of the X chromosome and a Y chromosome, consistent with Klinefelter syndrome and were excluded from further analysis. Plots of X het rate and XY ratio of the 11 remaining samples reported as showing a sex mismatch by PLINK were re-examined. Three of these samples had a low XY ratio and low X het rate and were likely to be XO (Turner syndrome) or XX/XO mosaics. All 11 samples were subsequently excluded leading to a total exclusion of 110 samples for sex mismatches.

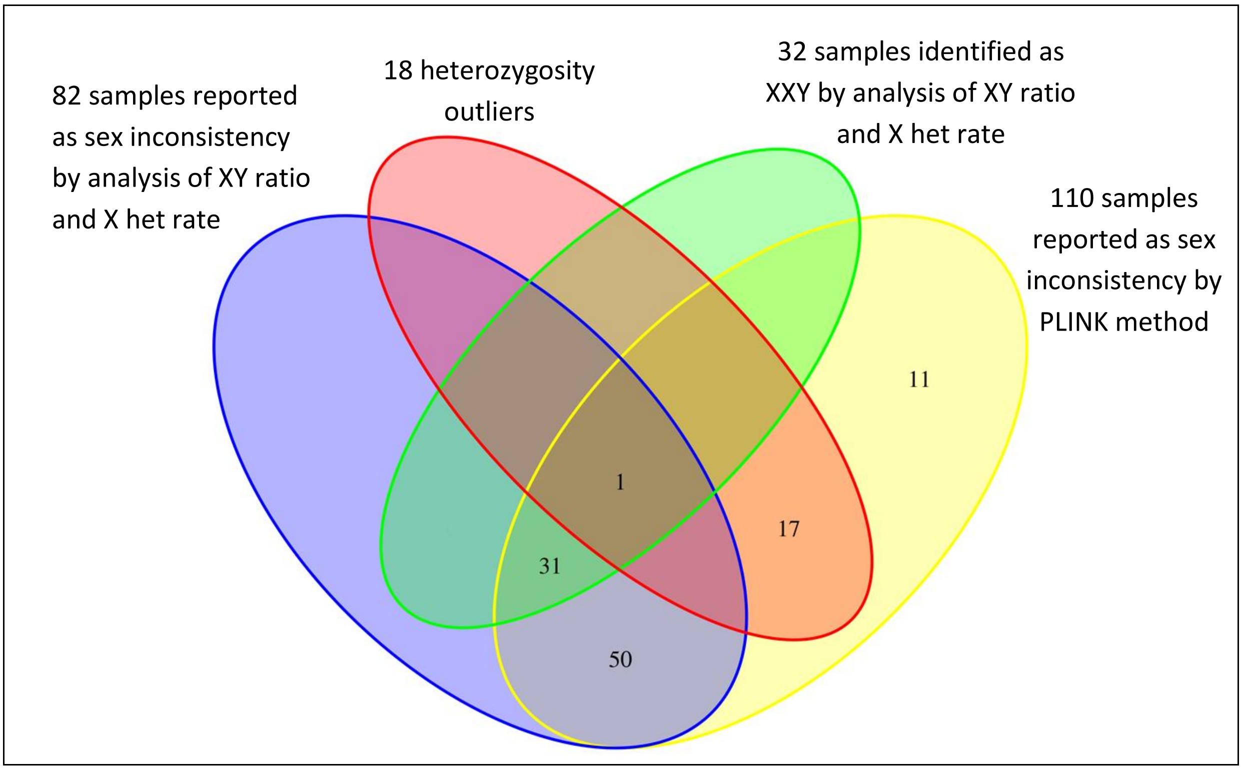

Supplementary Methods Figure 2: Samples reported as having a different sex based on genotype data to that provided by UK Biobank

Sample QC: Heterozygosity

Heterozygosity rate per sample was calculated based on 602,584 autosomal variants with MAF>1%. Supplementary Methods Figure 3 shows a scatter plot of heterozygosity rate against call rate. A total of 333 Samples with a heterozygosity rate greater than 3 SD from the mean were excluded.

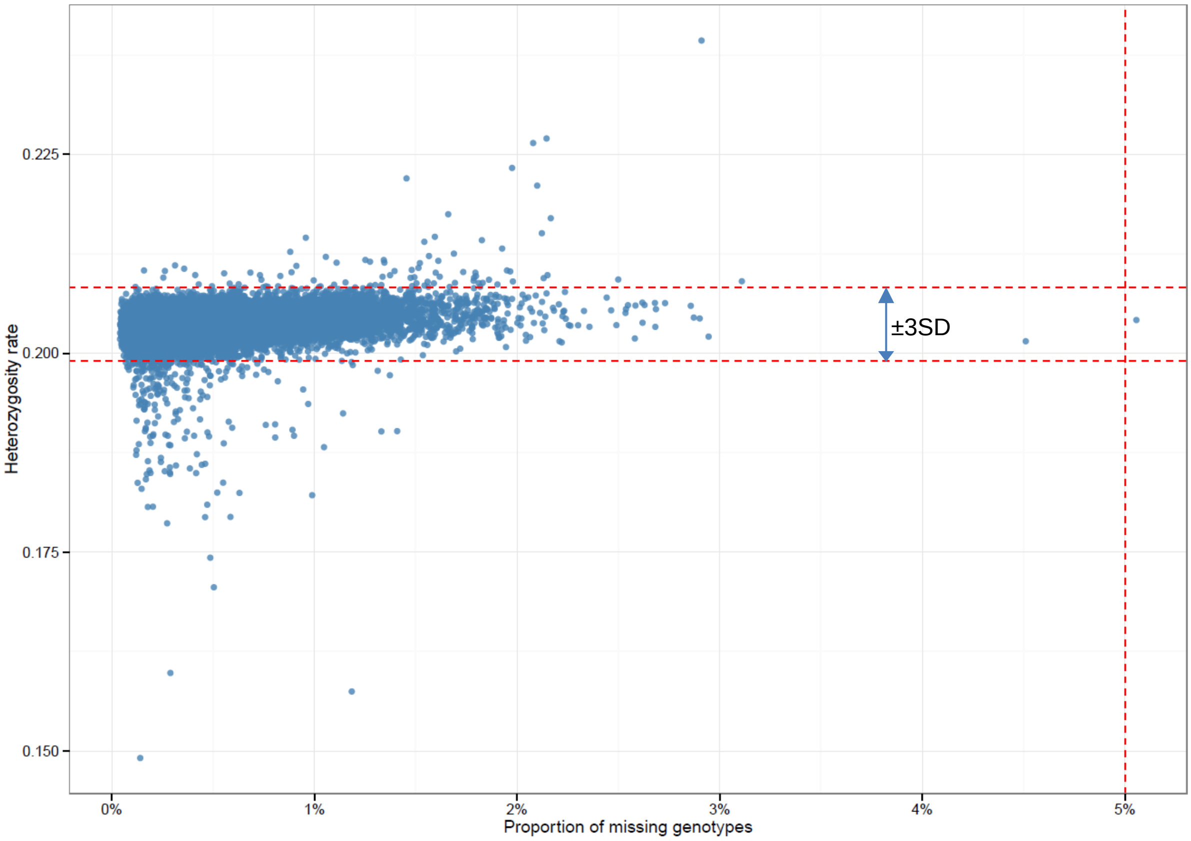

Supplementary Methods Figure 3: Heterozygosity rate vs sample call rate.

Sample QC: Relatedness estimation

The proportion of alleles shared IBD, inferred using PLINK v1.07[19], was used to identify unintended duplicates, confirm intended duplicates and infer relatedness. A subset of autosomal variants was selected based on the following criteria: MAF > 1%, Hardy Weinberg Equilibrium (HWE) ($P > 10^{-6}$), outside regions of strong LD and inversions. These variants were then pruned based on LD ($r^2 > 0.2$ within 50 variant windows) to identify a subset of 244,507 independent variants.

Supplementary Methods Figure 4 shows a scatterplot of the proportion of variants where a pair share 1 allele IBD (Z1) plotted against the proportion sharing 0 alleles IBD (Z0). Hence parents and offspring who share 1 allele IBD at all genotypes (Z1=1, Z0=0) are in the top-left, duplicates and identical twins share 2 alleles IBD across all variants and hence have 0 variants sharing only 1 or 0 alleles IBD (Z1=0, Z0=0) and siblings on average have 50% of variants where they share 1 allele IBD and 25% of variants where they share 0 alleles IBD (Z1=0.5, Z0=0.25). Cousins, half-siblings etc. lie on the line of slope -1, intercept 1, with relatedness decreasing towards Z1=1, Z0=0. A threshold of PI_HAT < 0.2 was used to define unrelated pairs where PI_HAT = Z2 + 0.5 × Z1.

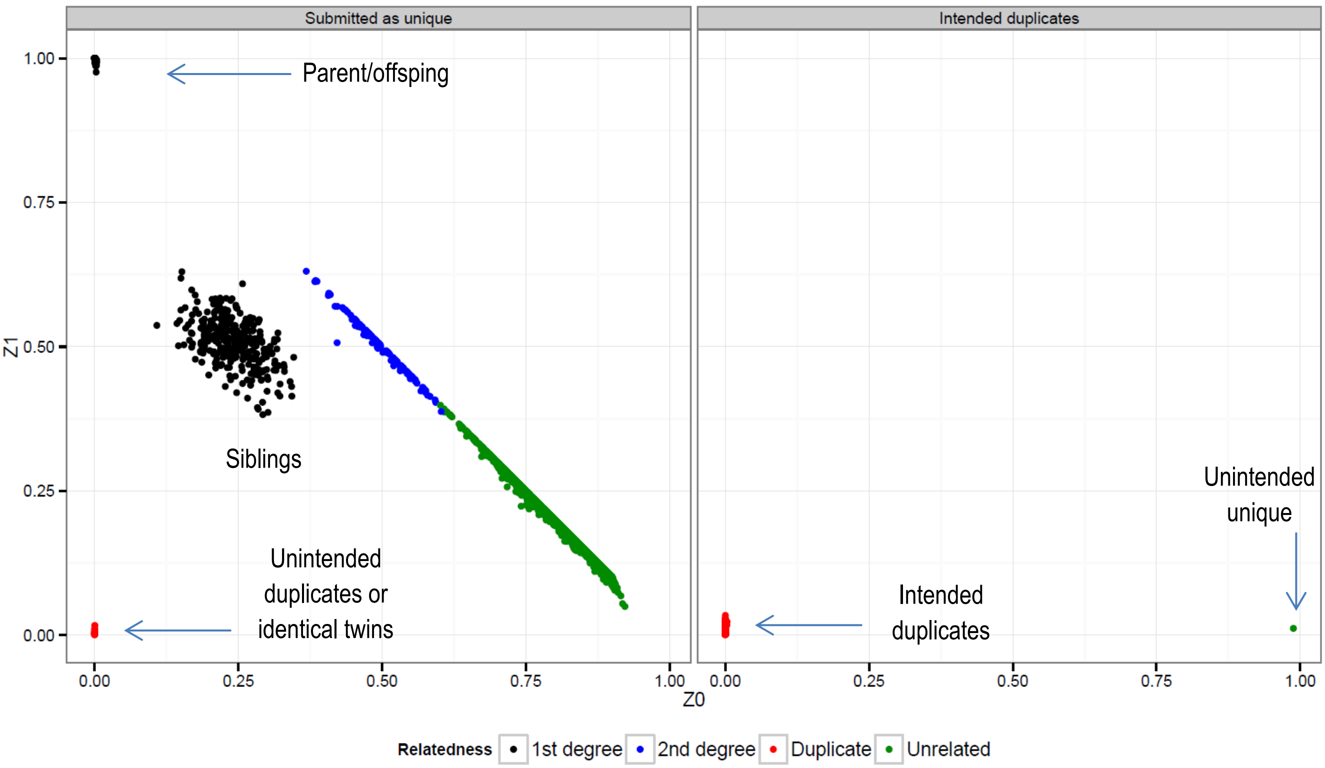

Supplementary Methods Figure 4: Proportion of genotypes where a pair share 1 allele IBD (Z1) plotted against the proportion sharing 0 alleles IBD (Z0) for samples submitted as unique (left panel) and samples submitted as intended duplicates (right panel). Each point represents a pair of samples.

Seven unintended duplicate pairs were identified and were reported back to UK Biobank. Further investigation of these pairs led to exclusion of 6 unique participants corresponding to 17 sets of genotype data.
A total of 481 duplicate pairs which were intended were identified and the sample with the lowest call rate of the pair was removed in each case.

Sample QC: Principal components analysis of ancestry

The intersection of variants used for IBD analysis (described above) and the HapMap3 reference panel were used for PCA of ancestry (43,232 variants). Principal component variant weightings were derived using 987 unrelated HapMap samples and then used to calculate the scores on the principal components of the UK BiLEVE samples using EIGENSOFT 4.2. Supplementary Methods Figure 5 shows that the UK BiLEVE samples' principal component scores lie in the region associated with European ancestry (HapMap CEU and TSI) as expected. Samples which were more than 10 SD outside of the mean score for any of the first 10 principal components were excluded. A total of 104 samples (58 male, 46 female) were excluded, with the following breakdown of outliers excluded by principal component: PC1=19, PC2=56, PC3=22, PC5=7.

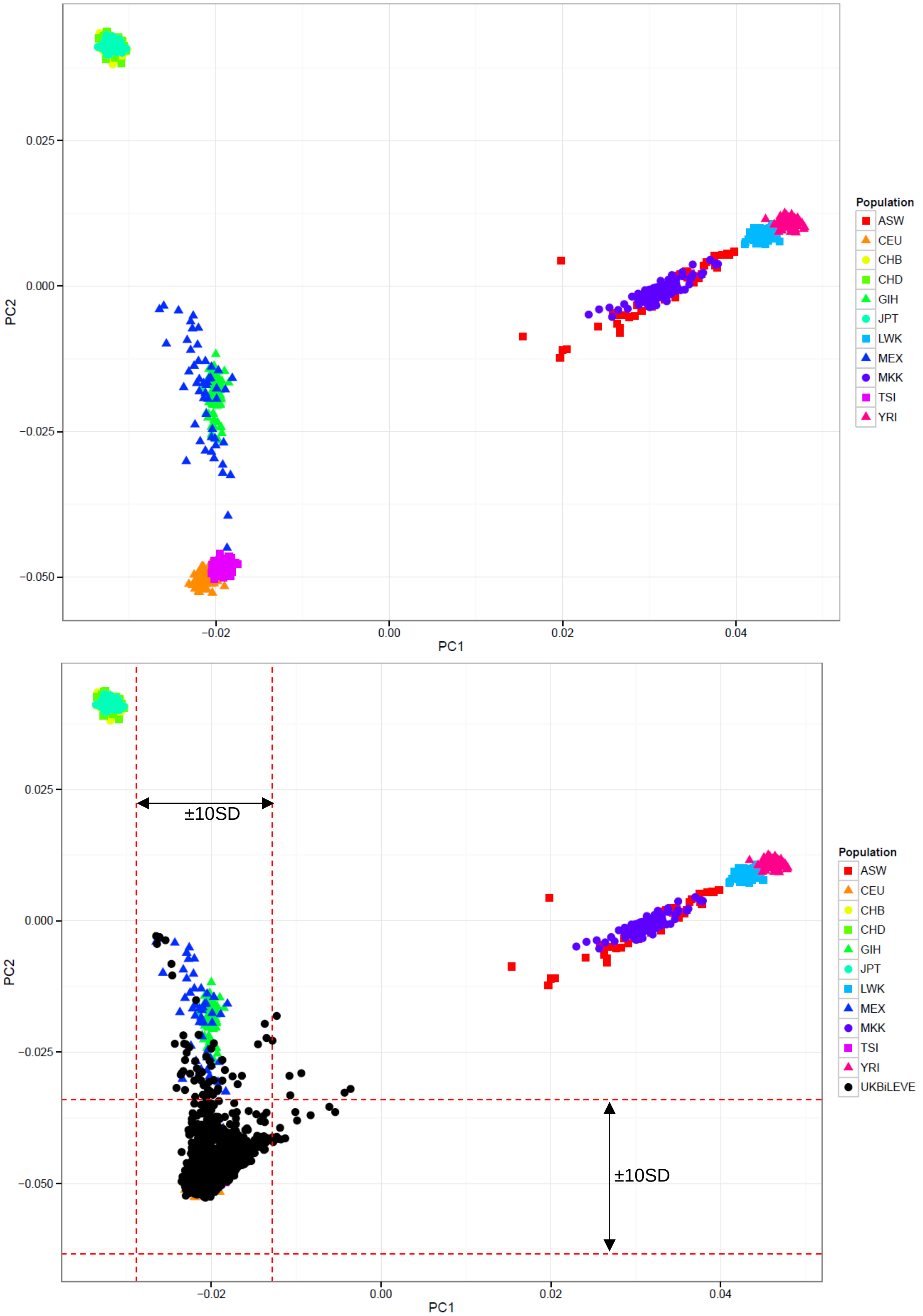

Supplementary Methods Figure 5: First 2 ancestry principal components for HapMap3 populations (top), with UK BiLEVE samples overlaid (bottom).

To test whether there was an association between PCA outlier status and lung function subgroup, we performed a chi-squared test and found no significant evidence of association (P=0.07) (Supplementary Methods Table 3).

	Heavy smokers			Never smokers		
**Outlier**	**Low FEV_1**	**Average FEV_1**	**High FEV_1**	**Low FEV_1**	**Average FEV_1**	**High FEV_1**
**FALSE**	9,883	9,902	4,955	9,855	9,907	4,950
**TRUE**	19(-2)	25(+4)	6(-4)	31(+10)	16(-5)	7(-3)

Supplementary Methods Table 3: Contingency table for association of PCA outlier status with phenotype group. The difference from the expected count under independence is shown in brackets.

Sample QC: Related individuals

Prior to PCA analysis, a total of 526 pairs of samples showed evidence of relatedness by IBD analysis (PI_HAT > 0.2, see above). One of the samples in one of these pairs was subsequently excluded by the PCA analysis leaving 525 pairs of samples showing evidence of relatedness. Although association testing methods that take relatedness into account are well-developed, given the small proportion of related individuals amongst the UK BiLEVE samples (~1%), we excluded related individuals from downstream association testing as follows (NB: related individuals were included in the imputation process but excluded prior to association testing).

Of the 525 pairs of samples showing evidence of relatedness, 1,000 samples were related to only one other sample and for these 500 pairs, the sample with the lowest call rate was excluded. Within the remaining 25 pairs, 30 samples were related to more than one other sample (indicative of more than 2 members of the same family). For these 25 pairs, we grouped the samples into families and assessed family relationships based on ages and sex. In all families, all samples were recruited from the same recruitment centre. We excluded individuals from each family so as to retain as many unrelated individuals as possible. For example, for a mother-father-offspring trio, the offspring was excluded so as to retain the unrelated mother and father. Where only one sample could be retained from a family, the sample with the highest call rate was selected. A total of 515 samples were excluded from association testing.

Variant QC: plate effects

As described above, variants were excluded within each batch if they demonstrated poor clustering. In addition, the presence of plate effects within each genotyping batch was assessed by a chi-squared test of association of allele frequency with plate. Variants with $P < 10^{-6}$ evidence of association were excluded (i.e. set to missing) for that batch. Only plates with at least 24 samples were included in the tests for plate effects. 6,114 variant-plate combinations showed a plate effect i.e. 4,698 variants showed a plate effect in one or more of 384 plates (Supplementary Methods Figure 6).

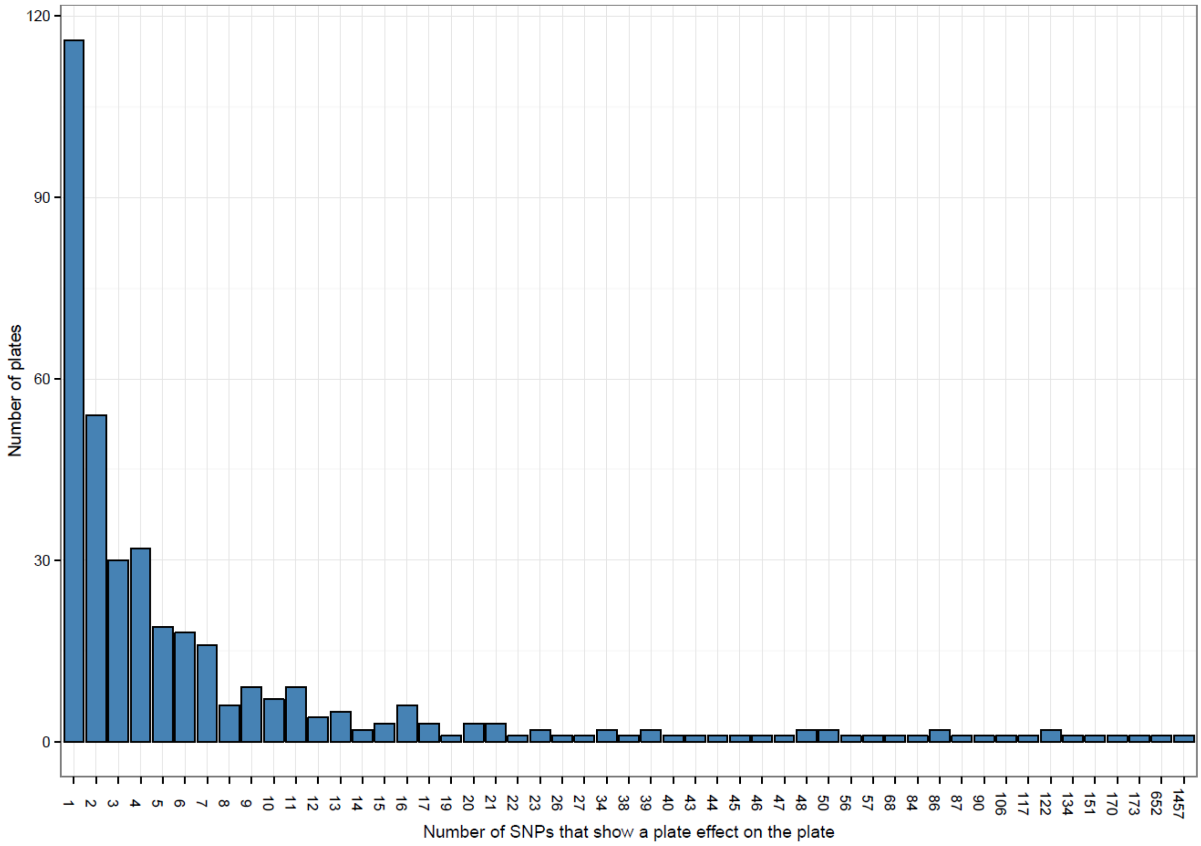

Supplementary Methods Figure 6: Number of plates out of 550 showing a plate effect for N variants. There were 116 plates that showed a plate effect for a single variant. The worst performing plate showed a plate effect for 1,457 variants (0.2% of total of 807,411 variants).

Following merging of variants across all 11 batches, for a given variant, if there was a batch that had a significantly different allele frequency compared to the other batches then that variant was flagged as exhibiting a batch effect. A total of 4,009 variants were flagged as having a batch effect.

Variants which failed cluster QC or plate effect QC in more than 2 batches were considered to have failed overall and were removed from the data set. Variants which failed in 1 or 2 batches had all genotypes set to missing in those 1 or 2 batches but genotypes were retained for other batches. NB: a variant that failed in 1 batch would have had a maximum call rate in the final merged data set of 91% and a variant that failed in 2 batches would have had a maximum call rate in the final merged data set of 82%. A total of 782,260 variants remained after QC.

Supplementary Methods Figure 7 summarises the number of variants which failed cluster QC, exhibited a plate effect or were flagged as exhibiting a batch effect in N batches.

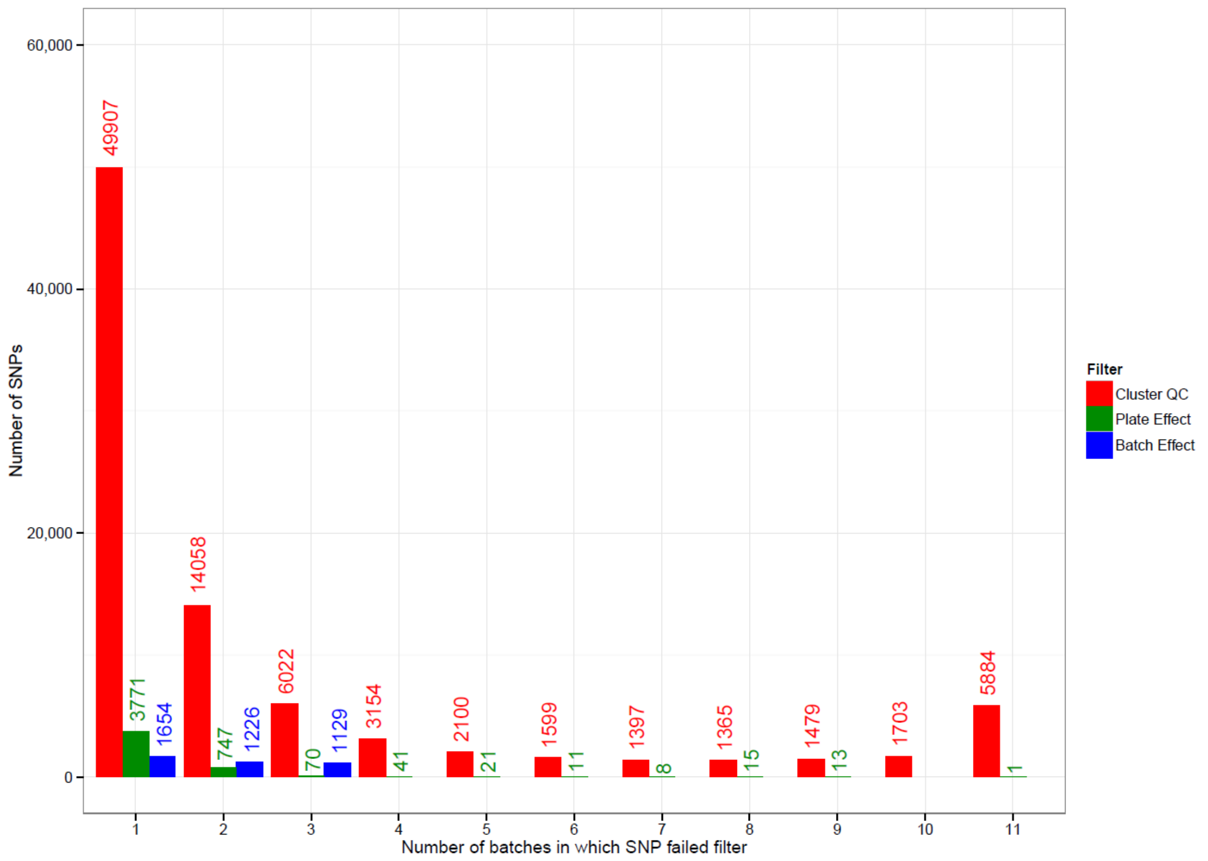

Supplementary Methods Figure 7: Number of variants out of 807,411 failing different genotyping QC filters in N batches. Cluster QC is the clustering quality filters calculated by Affymetrix based on relative genotype cluster positions (FLD, HomRO, HetSO etc.) and also call rate <95%. Note for Batch Effect filter, once a variant had failed in 3 batches no further testing was done.

Description of genotype imputation using 1000 Genomes Project and UK10K Project reference panels

Variants with MAF < 1% (123,306 variants) and HWE $P<10^{-6}$ (39,924 variants) were excluded leaving 619,030 variants for input into imputation. Pre-imputation phasing was performed with SHAPEIT v2.r727 and SHAPEIT v3 across all 49,458 samples, separately by chromosome, using the default parameters and HapMap phase II map of recombination sites. Imputation was undertaken against the 1000 Genomes Project Phase 1[20] and UK10K[21] (EGA study and dataset codes: EGAS00001000713 and EGAD00001000776) reference panels which were combined using IMPUTE2 v2.3.1 (using the –merge-ref-panels option[22] with a buffer of 250 kb and an effective population size of 20,000). 7,053,246 singletons not present in the UK10K panel, 23,932 sites with a SNP and an INDEL at the same position and 443 within large structural deletions were removed from the 1000 Genomes panel before merging. The combined reference panel consisted of 42,792,356 variants across a panel of 4,783 samples. Imputation was undertaken in subsets of 5,000 samples and in 3 Mb genomic chunks with a 250 kb overlap between adjacent chunks. After imputation of all 49,458 UK BiLEVE samples, imputation quality information scores were re-calculated across the 49,458 using QCTOOL v1.4 (-snp-stats option) and used for subsequent filtering. A total of 3,076 variants were genotyped in UK BiLEVE, had MAF > 1% and HWE $P > 10^{-6}$ and were input into the imputation but were not in the combined reference panel. This led to a final imputation output of 42,795,484 variants. Variants with an imputation quality information score (INFO) < 0.5 were excluded. A total of 55,260 directly genotyped variants were of MAF < 1% or HWE $P < 10^{-6}$ and so were excluded from the input for imputation and were not in the combined reference panel. These variants were merged back into the dataset and 28,509,962 variants were taken forward for association testing.

Of our 21.6 M well-imputed and genotyped-only, common autosomal variants (imputation INFO > 0.5, MAC ≥ 20), 6,279 (0.03%) had HWE $P < 10^{-6}$ and 2,359 had HWE $P < 10^{-12}$.

Supplementary Methods Figure 8 gives an overview of the number of variants passing QC which were input into imputation and the final number of variants analysed across all UK BiLEVE samples.

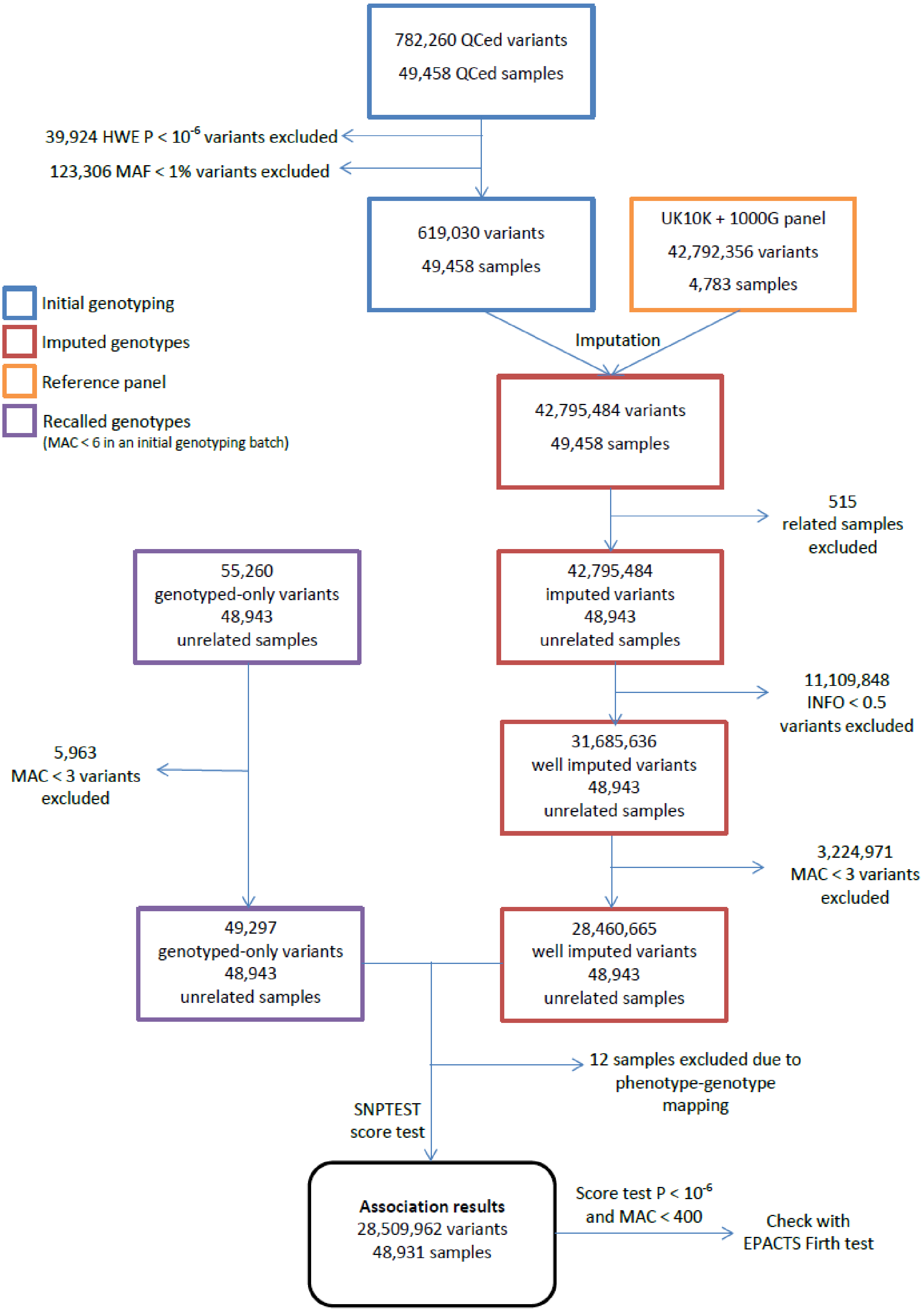

Supplementary Methods Figure 8: Flowchart of QC steps for imputation input variants, variants which were only genotyped (not in imputation panel) and association testing.

Description of association testing for autosomal and X, Y and mitochondrial variants

Genome-wide association testing was carried out for the following nested comparisons

- Heavy smokers with low FEV_1 vs heavy smokers with high FEV_1
- Never smokers with low FEV_1 vs never smokers with high FEV_1
- Heavy smokers with low FEV_1 vs heavy smokers with average FEV_1
- Never smokers with low FEV_1 vs never smokers with average FEV_1
- Heavy smokers with high FEV_1 vs heavy smokers with average FEV_1
- Never smokers with high FEV_1 vs never smokers with average FEV_1
- Heavy smokers vs never smokers

Within each comparison subset of the data, variants with a MAC < 3 were discarded. 515 samples were excluded due to evidence of relatedness, as described above. Association testing of each case-control group was undertaken using SNPTEST v2.5b4[23] (score test) under an additive genetic model of genotype dose (continuous from 0 to 2 reflecting imputation uncertainty), with the first 10 ancestry principal components as covariates and pack years of smoking as an additional covariate in the heavy smoking stratum. The same association model was used for the X chromosome but with male reference allele coded as 0 and alternate allele as 2; likewise for the Y chromosome (female samples removed) and mitochondrial (MT) SNPs (0 to 2 for both male and female) (Supplementary Table 20). For variants with MAC < 400 the association testing was repeated using the Firth test implemented in EPACTS v3.2.4, which is better calibrated for testing low MAC variants than the score test[24]. The genomic control inflation factor lambda was calculated across autosomes for each comparison and used to adjust for population stratification.
For all chromosomes, a P value threshold of $5x10^{-8}$ was used to signify genome-wide significant association. $P < 5 \times 10^{-7}$ was used to signify suggestive association for autosomal chromosomes and chromosome X. Bonferroni-corrected suggestive significance thresholds for signals on the Y and MT chromosomes and in the pseudo-autosomal region were defined as $P < 2 \times 10^{-4}$ (250 variants), $P < 3.6 \times 10^{-4}$ (3.3×10^{-4}, 140 variants) and $P < 3.7 \times 10^{-5}$ (1342 variants), respectively.
Full genome-wide association results are available via UK Biobank (access@ukbiobank.ac.uk).

Selection of signals

"Sentinel" variants representing independent signals of association were identified by iteratively selecting the variant with the lowest P value, assigning that variant as a sentinel and excluding all variants +/-500kb from the sentinel variant before repeating the process. Sentinel variants were annotated using ANNOVAR[25]. For sentinel variants with MAC < 400, we repeated local imputation and association testing following removal of genotyped SNPs with poor clustering (judged by eye); the variant was retained if $P<5x10^{-8}$ following re-analysis.

Calculations of linkage disequilibrium

LD between variants was calculated based on all 49,458 samples using vcftools v0.1.12a (--geno-r2 option i.e. squared correlation coefficient between genotypes encoded 0 to 2).

Proportion of variance explained

The proportion of variance in FEV_1 explained by the previously and newly reported variants was calculated as:

$$\frac{\sum_{i=1}^{n} 2f_i(1-f_i)\beta_i^2}{V}$$

where n is the number of variants f_i and β_i are the effect-allele frequency and effect estimate of the i'th variant, and V is the phenotypic variance. We used the effect estimates from a meta-analysis of quantitative FEV_1 across smokers and non-smokers where FEV_1 is adjusted for age, age^2, sex and height and then rank inverse-normal transformed. As with previously reported proportion of FEV_1 variance explained[4] we assumed a heritability of 40% to estimate the proportion of additive polygenic variance.

Genome-wide analysis of SNP x smoking interaction

The following statistic was used, both comparing the FEV_1 comparison for which the variant was significant in the heavy smokers with that in the never smokers (or vice versa), and also the low FEV_1 vs high FEV_1 comparison in the heavy smokers and in the never smokers:

$$Z = \frac{\beta_{heavy\ smokers} - \beta_{never\ smokers}}{\sqrt{SE_{heavy\ smokers}^2 + SE_{never\ smokers}^2}}$$

where under the null (H_0: $\beta_{heavy\ smokers} = \beta_{never\ smokers}$), $Z \sim N(0,1)$.

A genome-wide scan for smoking interaction was also performed using the above test with the effect estimates and standard errors from the low FEV_1 vs high FEV_1 comparison in the heavy and never smokers. Variants with $P < 5\times10^{-7}$ were followed up with 2 further tests: i) using the same Z statistic as above but with effects and standard errors from a Firth test to control for type I error in low MAC variants; ii) fitting a logistic model, updated from the logistic model used in the main analysis with a variant × smoking interaction term (implemented in R) and using a likelihood ratio test for significance, thereby using the individual level data to estimate the interaction effect.

Association with GOLD Stage 2+ COPD for novel signals of association with extremes of FEV_1

We undertook a case-control analysis for all SNPS in novel regions, which showed genome-wide significant association in at least one of the nested lung function comparisons. We selected 9,564 COPD cases, defined as those samples with GOLD Stage 2+ COPD according to spirometry ($FEV_1/FVC < 0.7$ and % predicted $FEV_1 < 80\%$), and 9,453 controls, selected from the high FEV_1 strata and with $FEV_1/FVC > 0.7$ (all had % predicted FEV_1 in excess of 80%). Post-bronchodilator spirometry was not available for any participants and medication was not withheld prior to spirometry being undertaken. Summaries of these samples are given in Supplementary Methods Table 4.

Analyses were carried out using the score test, implemented in SNPTEST v2.5b4[23] and assuming an additive genetic model of genotype dose. For never smokers, sex, age and the first 10 ancestry principal components were included as covariates. For heavy smokers, pack years were included as an additional covariate. The results for never and heavy smokers were then combined, using inverse variance weighted meta-analysis.

		COPD Cases	Controls	Total
**Heavy smokers**	**n**	5,803	4,661	10,464
	**% predicted FEV_1 mean (SD)**	61.2 (11.8)	118.0 (8.1)	
	**FEV_1/FVC mean (SD)**	0.60 (0.08)	0.78 (0.04)	
**Never smokers**	**n**	3,761	4,792	8,553
	**% predicted FEV_1 mean (SD)**	65.4 (11.4)	130.3 (8.3)	
	**FEV_1/FVC mean (SD)**	0.63 (0.07)	0.79 (0.04)	
	**Total**	9,564	9,453	19,017

Supplementary Methods Table 4: Sample sizes and mean and standard deviation % predicted FEV_1 and FEV_1/FVC of GOLD stage 2+ COPD cases and controls in heavy smokers and never smokers.

Analysis of polygenic architecture of diseases and health-related traits

Risk scores[26] and GCTA[27, 28] were used a) to investigate whether there was evidence for polygenic architecture[29] of FEV_1-defined traits, b) to investigate shared genetic aetiology of FEV_1 between never smokers and heavy smokers, c) to identify whether the genetic variants underlying high FEV_1 also predicted low FEV_1 and d) to explore shared aetiology between individuals with asthma and individuals without asthma. The scores allow the combined influence of many variants with weak effects to be observed by comparing a discovery group and a target group. GCTA was used to estimate the proportion of variance explained in the target population by subsets of variants chosen from the discovery population.

QC of individuals and genotyped variants was undertaken as described above, with additional exclusion of variants based on HWE ($P < 0.001$ excluded) and MAF (MAF < 1% excluded). Only autosomal variants were included in these analyses.

The discovery and target groups for each analysis are described below. For each analysis, a GWAS was performed using PLINK v1.9 (Wald test) with the same covariates and additive genetic model, as described above, for the discovery group. For each variant a value for the log odds ratio and P value were obtained.

Scores for each allele were assigned as equal to the log odds ratio in the discovery group for variants which met a pre-defined P value threshold (scores were set to zero otherwise). P value thresholds of 1.0, 0.5, 0.4, 0.3, 0.2, 0.1, 0.05, 0.01 and 0.001 were investigated. To aid interpretation of the score analysis, log odds ratios were set in the same direction, i.e. the effect allele was chosen as that with log odds ratio > 0.

Risk scores were then calculated for each individual in the target group by summing the score for each allele multiplied by the number of effect alleles across all variants, i.e.:

$$\text{Risk score}_i = \sum_{j=1}^{n} (\text{Score for allele})_j \times (\text{Number of effect alleles})_{i,j}$$

Where i is the individual, j is the variant and n is the number of variants investigated. These scores were then normalised ensuring the scores had a mean of zero and a standard deviation of one. To test if these risk scores

were associated with the phenotype in the target group, logistic regression was performed with the individuals' risk score as the only covariate.

The proportion of variance explained by the subset of variants generated for each target population from each P value threshold was calculated using GCTA[27, 28]. GCTA estimates the genetic relationship between individuals, and then, using REML and adjusting for covariates (in this instance the first 10 principal components and pack years), estimates the proportion of variance explained. Using all variants, for every pair of individuals found to have cryptic relatedness (cut-off value of 0.025) one individual was removed from analyses for each subset of variants. Case-control data is transformed onto a liability scale through an assumed prevalence level[30]. For investigating shared polygenic effects in FEV_1-defined traits, between high FEV_1 and low FEV_1 and between asthma and no asthma; prevalence was set to the proportion of low FEV_1 (21,000) in the whole sampling frame (275,915), i.e. the prevalence was set at 7.611%. We based estimates of prevalence on the known sampling frame from which the UK BiLEVE samples were selected with a known sampling strategy. Thus, when investigating the shared genetic architecture of low FEV1 across the strata defined by smoking status the prevalence was assumed to be the number of never smokers with low FEV_1 (10,500) divided by the number of never smokers in the sampling frame (105,272), i.e. a prevalence of 9.974%.

To first investigate whether there was a polygenic component associated with low $FEV_{1,}$ individuals with low FEV_1 and average FEV_1 were randomly split into discovery and target populations (Supplementary Methods Figure 9). To assess whether the genetic variants underlying high FEV_1 also predicted low FEV_1 (airflow obstruction), the discovery group comprised individuals with high FEV_1 and a random sub-sample of those with average FEV_1. The target sample consisted of those with low FEV_1 and the remaining individuals with average FEV_1 who were not included in the discovery sample (Supplementary Methods Figure 10). To investigate the shared genetic aetiology of low FEV_1 between never smokers and heavy smokers, heavy smokers with average FEV_1 and low FEV_1 were used as the discovery group and never smokers with average and low FEV_1 as the target group (Supplementary Methods Figure 11). Finally, to investigate shared genetic variants between those with and without asthma, the discovery population was selected as those reporting doctor diagnosed asthma with low FEV_1 or average FEV_1 and the target population as those with no doctor diagnosed asthma with low FEV_1 or average FEV_1 (Supplementary Methods Figure 12). Results are presented in Supplementary Table 2. Results were similar if variants with MAF < 5% were excluded.

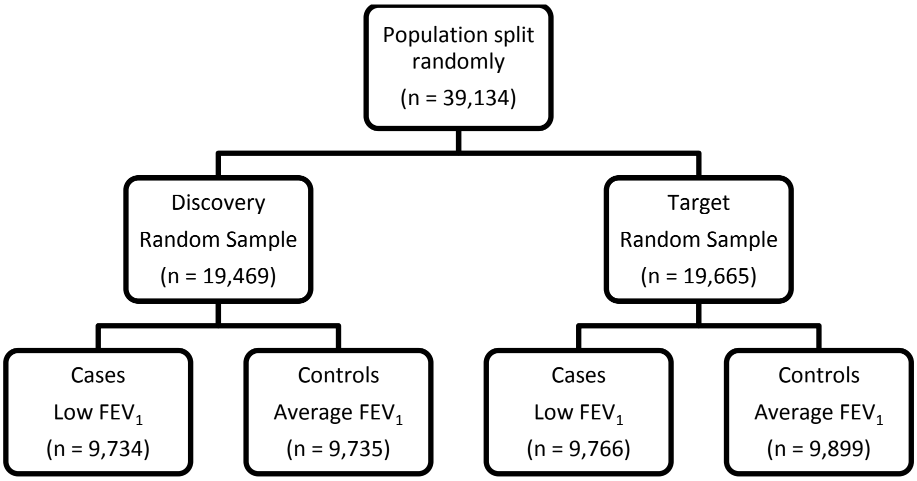

Supplementary Methods Figure 9: Sample sizes for the investigation of the polygenic architecture of FEV_1-defined traits.

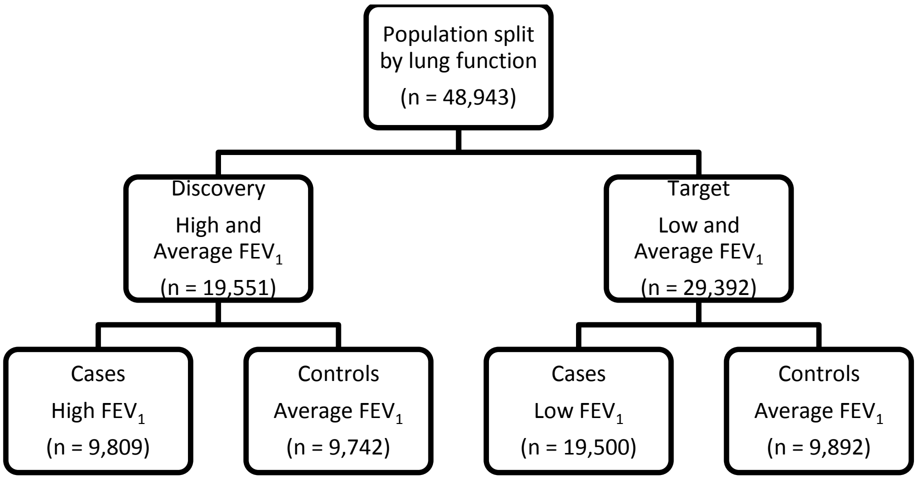

Supplementary Methods Figure 10: Sample sizes for the investigation of the shared genetic aetiology between high FEV_1 and low FEV_1.

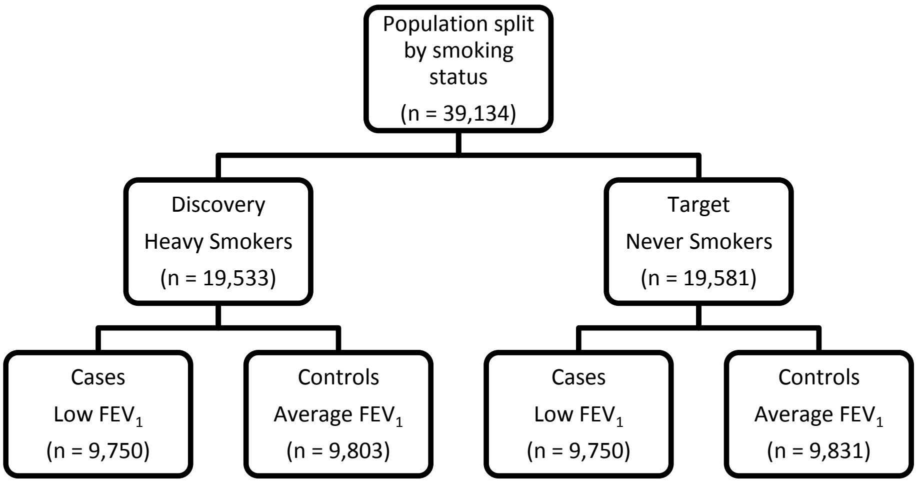

Supplementary Methods Figure 11: Samples sizes for the investigation of the shared genetic aetiology between heavy and never smokers.

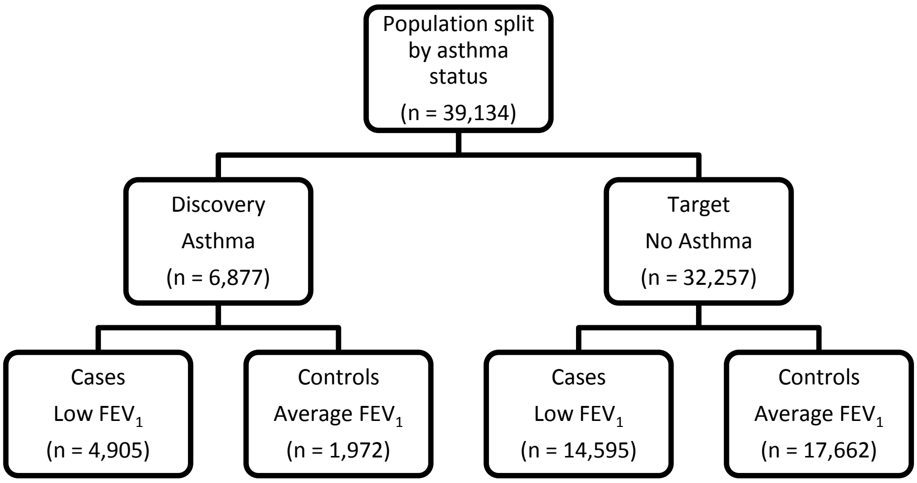

Supplementary Methods Figure 12: Sample sizes for the investigation of the shared genetic aetiology between individuals with doctor diagnosed asthma and individuals with no doctor diagnosed asthma.

Association with self-reported/doctor diagnosed asthma of loci previously reported for genome-wide significant association with asthma

Asthma cases were defined as participants that either (i) answered "asthma" to a touchscreen question *"Has a doctor ever told you that you have had any of the following conditions? (You can select more than one answer) (Blood clot, DVT, bronchitis, emphysema, asthma, rhinitis, eczema, allergy)*", or (ii) reported asthma in verbal interview, as per any of the self-reported, non-cancer illness fields. Using this definition, we identified 7,488 asthma cases and 41,455 controls within the 48,931 unrelated samples passing the QC steps described above. We tested for association with asthma for 17 variants at 12 loci which had previously shown genome-wide significant ($P<5x10^{-8}$) association with asthma[11, 31-34]. Association testing was undertaking using SNPTEST using a logistic model with genotype dose with 10 ancestry principal components and pack years as covariates (0 for never smokers). Results are in Supplementary Table 1.

Effect on quantitative FEV_1 for novel signals of association with extremes of FEV_1

For each of the 6 novel signals of association with extremes of FEV_1, we tested association of FEV_1 as a quantitative trait separately in heavy smokers and never smokers using a linear model with imputed genotype dose and P values from a score test implemented in SNPTEST v2.5. Firstly, residuals from a linear regression of FEV_1 with age, age^2, sex, height and 10 ancestry principal components were obtained, which were then ranked and inverse-normal transformed. These normally distributed z-scores were used as the dependent phenotype in the linear regression. Results are presented in Table 2.

Analysis of expression data from lung, blood and brain tissues to identify if our novel signals affect gene expression (eQTL)

Lung

The descriptions of the lung eQTL dataset and subject demographics have been published previously[35-37]. Briefly, non-tumor lung tissues were collected from patients who underwent lung resection surgery at three participating sites: Laval University (Quebec City, Canada), University of Groningen (Groningen, The Netherlands), and University of British Columbia (Vancouver, Canada). Whole-genome gene expression and genotyping data were obtained from these specimens. Gene expression profiling was performed using an Affymetrix custom array (GPL10379) testing 51,627 non-control probe sets and normalized using RMA[38]. Genotyping was performed using the Illumina Human1M-Duo BeadChip array (using blood or lung samples). Genotype imputation was undertaken using the 1000G reference panel. Following standard microarray and genotyping quality controls, 1,111 patients were available including 409 from Laval, 363 from Groningen, and 339 from UBC. Lung eQTLs were identified to associate with mRNA expression in either cis (within 1 Mb of transcript start site) or in trans (all other eQTLs) and meeting the 10% false discovery rate (FDR) genome-wide significant threshold. Variants which showed evidence of association ($P < 5 \times 10^{-7}$) with extremes of FEV_1 and all proxy variants ($r^2 > 0.3$ with the sentinel variants) were queried. The results for the most significant variant × probeset pair for any genes identified in the look-up and the results for the sentinel variant and/or strongest proxy variants are presented in Supplementary Table 9. There was no significant evidence of association (FDR < 10%) for chr12:114743533, chr11:109843513 and rs34712979 (or proxies) in the data set.

Blood

Evidence for association with gene expression in blood was assessed for all variants which showed evidence of association ($P < 5 \times 10^{-7}$) with extremes of FEV_1 or smoking behaviour, and their proxies ($r^2 > 0.3$). A publicly available resource based on blood expression data from 5,311 individuals, imputed to HapMap 2 was used (resource previously described[39]). Cis and trans eQTL signals meeting the 10% FDR genome-wide significant threshold were identified. The results for the most significant variant × probeset pair for any genes identified in the look-up and the results for the sentinel variant and/or strongest proxy variants are presented in Supplementary Table 10a**.** Data were only available where FDR < 50%.

For loci where it could not be established whether an absence of signals with FDR<10% was due to signals of association with FDR > 10% (only results with FDR < 50% were publicly available) or because there were no data for those variants (either due to absence of a proxy in HapMap or variant QC failure), a 1000 Genomes Project imputed eQTL dataset from the Estonian Genome Project was also queried. These loci were those represented by the following sentinel variants: chr12:114743533, rs2047409, rs34712979, rs4466874, rs10193706, rs61784651 and rs10807199 (Table 2).

The Estonian cohort is from the population-based biobank of the Estonian Genome Project of University of Tartu (EGCUT). The project is conducted according to the Estonian Gene Research Act, and all participants have signed the broad informed consent. The current cohort size is > 51,515, 18 years of age and older, which reflects closely the age distribution in the adult Estonian population. Subjects are recruited by the general practitioners (GPs) and physicians in the hospitals were randomly selected from individuals visiting GP offices or hospitals. Each participant filled out a computer-assisted personal interview during 1-2 hours at a doctor's

office, including personal data (place of birth, place(s) of living, nationality. etc.), genealogical data (three generation family history), educational and occupational history, and lifestyle data (physical activity, dietary habits, smoking, alcohol consumption, women's health, quality of life).

EGCUT data contained 469 male and 490 female samples with average age 38.1. All samples were genotyped using HumanCNV370-DUO BeadChip and then imputed using 1000G phase 1 integrated variant set (Mar 2012), all ancestries reference set.

A total of 712 markers representing 8 loci (sentinel variants plus proxies with $r^2>0.3$) were specified and those with INFO > 0.5 were included in linear regression analysis with gene expression data (N=29,018 probes with full annotation information) with adjustments for sex, age, plate-id and top 46 principal components from the expression values with using SNPTEST2 v.2.5[23] software. Variant × probeset associations with $P < 2.15 \times 10^{-7}$ (Bonferroni-correction for analysis of 8 loci and 29,018 probes) were retained and results are presented in Supplementary Table 10b.

Brain

Evidence for association with gene expression in brain was assessed for all variants which showed evidence of association ($P < 5 \times 10^{-7}$) with smoking behaviour, and their proxies ($r^2 > 0.3$). A publicly available resource of expression data from 10 brain regions in 134 individuals, with variant genotype data imputed to 1000 Genomes Project phase 1 reference panel was used (resource previously described[40]). Cis and trans eQTL signals meeting the 1% FDR genome-wide significant threshold were identified. The results for the most significant variant × probeset pair for any genes identified in the look-up and the results for the sentinel variant and/or strongest proxy variants are presented in Supplementary Table 14.

Analysis of differential expression of candidate genes in the lungs of individuals with and without COPD

Genes were defined as candidate genes for novel signals of association with extremes of FEV_1 if they contained a) the sentinel variant or were the nearest genes, b) a putatively functional variant within the gene, correlated with the sentinel variant, was identified through conditional analysis as explaining the observed association (see Supplementary Table 21) or c) the sentinel variant or a strong proxy variant ($r^2 > 0.8$) was an eQTL for that gene. Publically available microarray data (GSE37147[41]) was mined using GEO2R on the gene expression omnibus website (http://www.ncbi.nlm.nih.gov/geo/info/geo2r.html). Two sample groups were defined. Affymetrix Human ST1.0 array expression data for 87 bronchial brushings in the lungs of individuals with COPD was defined as the first group, whilst the second group had the expression profiles of 151 bronchial brushings from individuals without COPD. There were no significant differences in age, cumulative smoking exposure or smoking status between the individuals with COPD and those without COPD[41]. Differential expression between the 2 groups was identified using the default array statistics. P values were adjusted for multiple testing using the Benjamini & Hochberg method[42]. Results are presented in Supplementary Table 22.

Analysis of differential expression of candidate genes in the developing foetal lung

Genes were defined as candidate genes for novel signals of association with extremes of FEV_1 if they contained a) the sentinel variant or were the nearest genes, b) a putatively functional variant within the gene, correlated with the sentinel variant, was identified through conditional analysis as explaining the observed association (see Supplementary Table 21) or c) the sentinel variant or a strong proxy variant ($r^2 > 0.8$) was an eQTL for that gene. Publically available Affymetrix U133 Plus 2 array data (Gene expression omnibus: GSE14334) of 38 foetal lung samples from the Pseudoglandular (7 - 16 weeks) and Canalicular (17 - 22 weeks) stages of lung development was mined as previously reported[43]. Results are presented in Supplementary Table 7.

Messenger RNA sequencing in human bronchial epithelial cells (HBECs) to identify novel transcripts of genes at novel loci associated with the extremes of FEV_1

We looked for evidence of novel transcripts for genes containing the sentinel SNPs associated with extremes of FEV_1 and for genes which were regulated by nearby (<1Mb) SNPs (eQTLs) using RNA sequencing in HBECs. Passage 3 normal human bronchial epithelial cells (NHBECs) (Lonza, UK), were cultured in growth factor-supplemented medium (BEGM, Lonza as described previously53. Cells were grown under these conditions and four different experimental conditions as part of a related RNA interference (RNAi) project each in three independent biological replicates (12 samples in total). Total RNA was extracted using established methods for RNA isolation (Sigma-Aldrich GenElute Mammalian Total RNA Miniprep Kit) and RNA quality was assessed for degradation on Agilent 2100 Bioanalyzer with all twelve samples having an RNA Integrity Number (RIN) at ~8 or above 8. The sequencing library was prepared with Illumina TruSeq RNA Sample Prep Kit v2. mRNA was poly-A selected by capturing total RNA samples with oligo-dT coated magnetic beads. The mRNA was then fragmented and randomly primed. cDNA was synthesised using random primers. Finally ready-for-sequencing library was prepared by end-repair, phosphorylation, A-tailing, adapter ligation and PCR amplification. Paired-end sequencing was performed on the Illumina HiSeq2000 platform using TruSeq v3 chemistry over 100 cycles yielding approximately 40 million reads per sample. The generated raw reads FastQ

files (100 base pairs; Sanger / Illumina 1.9 encoding) were quality evaluated using FastQC. Mean quality scores across the bases for all reads in all twelve samples were above 28. Un-modified reads were used for subsequent analysis on Ubuntu 12.04 LTS operating system. Un-spliced alignments onto human genome build GRCh37 were performed for each sample individually using Bowtie2 tool utilized by TopHat v2.0.1254. Reads aligning to more than 20 positions were discarded. The subset of reads that were not aligned uniquely were used by TopHat to identify splice junctions. Cufflinks v2.2.155,56 programme was used to assemble transcriptome for each individual sample. Transcriptomes from all the samples were merged using Cuffmerge v1.0.0 feature in order to identify low-expression transcripts requiring deep sequencing coverage. The Cuffmerge generated novel gene transfer format (GTF) annotation file was compared to Ensembl GTF annotation of GRCh37 genome build by using Cuffcompare v2.2.1. All 12 NHBEC samples were used for transcriptome assembly in order to identify reported and novel transcripts. Cuffdiff v2.2.1 generated isoform expressions file was used to determine mRNA variants abundance in untreated NHBEC under basal culture conditions by calculating isoforms' percentage of total transcripts fragments per kilobase of exon per million fragments mapped (FPKM) expression. Splicing graphs depicting novel and known splice transcripts were generated using SpliceGrapher v0.2.457 (Supplementary Figure 6).

Pathway analysis using MAGENTA

We tested whether the results of the meta-analysis of low FEV_1 vs high FEV_1 across heavy smokers and never smokers were enriched for known biological pathways using MAGENTA v2[44]. Briefly, MAGENTA defines a P value for each gene that is the lowest variant P value within 110kb upstream and 40kb downstream of the gene and is corrected for gene size, number of variants per gene and LD within the region. For each gene set, the null hypothesis that there is a random distribution of gene association score ranks within the gene set is tested against the alternative hypothesis that there are more gene association score ranks above a given rank cut-off (75th percentile cut-off is recommended for polygenic traits) compared to random sampling of 10,000 gene sets of identical size. For each gene set, a FDR is calculated as the fraction of all randomly sampled gene sets (10,000 × number of gene sets tested) that have more genes with P value below the cut off (75th percentile) than in the gene set being tested, divided by the fraction of real gene sets that have more genes with P value below the cut off (75th percentile) than in the gene set being tested.

Six databases of biological pathways were tested: including Ingenuity Pathway (June 2008, number of pathways n=92), KEGG (2010, n=186), PANTHER Molecular Function (January 2010, n=276), PANTHER Biological Processes (January 2010, n=254), PANTHER Pathways (January 2010, n=141) and Gene Ontology (April 2010, n=9542). Significance thresholds were Bonferroni corrected for each database.

Variants with MAC less than 400 were excluded. Genes within 500kb of the genome-wide significant associations with FEV_1 reported in this paper, and within 500kb of the 32 variants previously reported as associated with FEV_1, FEV_1/FVC and/or FVC[2-4, 45] were flagged. Results are listed in Supplementary Table 17.

Stepwise conditional analysis to identify additional independent signals at the novel loci

We used a stepwise selection procedure implemented in GCTA[46] to identify independent signals within all the novel regions. This method starts by conditioning all the variants in a region by the most significant variant and then it uses a stepwise procedure to select other variants for which joint P values meet a pre-specified threshold (10^{-3} in this analysis).The software then returns P values for a joint model containing the stepwise-selected independent variants. The joint model P values returned by GCTA were checked by fitting the joint model in R with the glm function. Results are presented in Supplementary Table 6. Variants with a joint conditional $P < 10^{-4}$ were defined as being independent.

Imputation and association testing of structural variation haplotypes in the inversion locus at chromosome 17q21.31 (*KANSL1*)

An imputation reference panel for the nine structural haplotypes observed at 17q21.31 was provided[47]. The structural haplotypes were encoded in the reference panel in the form of bit patterns of 12 surrogate, virtual bi-allelic variants. In this way standard imputation procedures could be used to impute the genotypes of the surrogate markers which could then be decoded into the corresponding structural haplotypes. The reference panel was provided in unphased Beagle 3 format and comprised the 12 surrogate markers and 6,302 flanking variant haplotypes. The reference panel was phased using Beagle 3.3.2[48] then converted to IMPUTE2 format with R. IMPUTE2 v2.3.1 was used for imputation against the reference panel using 185 genotyped variants within the reference panel region, excluding variants within the copy-number variable region. The imputed haplotype frequencies showed acceptable agreement with frequencies for 467 CEU individuals determined by droplet-based digital PCR or sequencing[47] (Supplementary Methods Table 5).

HAPLOTYPE	CEU[47]	imputed
H1.β1.γ1	27.72%	30.02%
H1.β1.γ2	9.90%	10.83%
H1.β1.γ3	15.35%	13.51%
H1.β1.γ4	0.99%	0.18%
H1.β2.γ1	27.23%	22.59%
H1.β3.γ1	1.49%	0.06%
H2.α1.γ2	0.99%	0.73%
H2.α2.γ1	0.99%	0.05%
H2.α2.γ2	15.35%	22.04%

Supplementary Methods Table 5: Imputed haplotype frequencies for 17q21.31 inversion region compared to CEU frequencies provided with imputation reference panel. The haplotypes are defined on the uninverted (H1) or inverted (H2) region with different copy numbers of the regions α, β and γ within the inversion region[47].

We tested association of low FEV_1 versus high FEV_1 with copy number count of the α, β and γ structural polymorphisms using logistic regression across both smoking and non-smoking strata, with 10 ancestry principal components and pack years as covariates (0 pack years for never smokers) (Supplementary Table 11).

Corroborative evidence supporting loci with genome-wide significant evidence of association with extremes of FEV_1

We searched for corroborative evidence of association with FEV_1 for our novel signals of association with extremes of FEV_1 in i) an independent subset of the UK BiLEVE sample and ii) in publicly available association results from a previous large GWAS of FEV_1 in the general population[4] (n=48,201, ever and never smokers first analysed separately and then meta-analysed).

Where the novel signal was identified in never smokers, the results for the same SNP were extracted for the same comparison (i.e. low FEV_1 vs high FEV_1) in heavy smokers, and vice versa, in UK BilEVE. From the previous large GWAS, we extracted the meta-analysis P values for association with FEV_1 for all sentinel SNPs and their proxies (linkage disequilibrium $r^2 > 0.3$). We report both the most significantly associated proxy SNP and the P value for the sentinel or strongest proxy. All results are in Supplementary Table 18.

Corroborative evidence supporting loci with genome-wide significant evidence of association with smoking behaviour (heavy smokers vs never smokers)

To provide corroborative evidence to support our genome-wide significant findings of association with smoking behaviour at 4 loci, regional imputation, association testing and meta-analysis across 15 studies was undertaken. The primary analysis was a comparison of ever smokers vs never smokers (smoking initiation). Secondary analyses of current smokers vs non-current (smoking cessation) and smoking quantity (smoking quantity levels were 0 (defined as 1-10 cigarettes per day (CPD)), 1 (11-20 CPD), 2 (21-30 CPD) and 3 (31 or more CPD)) were also undertaken. Supplementary Methods Table 6 describes the sample sizes available for each study. SHAPEIT2[49] was used to phase a region 500Kb either side of each site with 200 conditioning states in the phasing run. Imputation was carried out using IMPUTE2[50] with the 1000 Genomes Phase 1 dataset as a reference panel. SNPTEST was used to carry out association testing.

Age and sex were included as covariates within each cohort. Some of the cohorts were analysed using other covariates, such as principal components and case-control status (see Supplementary Material of Liu et al.[12]). META[12] was used to apply meta-analysis across studies. The meta-analysis was carried out by combining study-specific β estimates using a fixed effects model, which used the inverse of the variance of the study-specific β estimates to give weight to the contribution of each study. The variance of each cohort's β estimate was multiplied by the genomic control λ estimate to correct for observed inflation. The genomic control λ estimates for each study were taken from Liu et al. (2010)[12]. At each variant only those studies which had INFO ≥ 0.5 were included in the meta-analysis. Results are given in Supplementary Table 19.

Cohort	Never smokers	Ever smokers	Non-current smokers	Current smokers	Smoking quantity
GSK_BIPOLAR	546	657	344	313	600
GSK_EPIC	1589	1927	1574	353	0
GSK_KORA	831	811	1425	217	251
GSK_LOLIPOP	635	653	395	258	648
GSK_UNIPOLAR	856	935	432	503	897
GSK_COPD	0	0	905	725	1630
GSK_GEMS	793	910	642	268	860
GSK_LAUSANNE	2275	3357	1872	1485	3130
GSK_MEDSTAR	469	853	553	300	818
GSK_POPGEN	494	608	0	0	571
GSK_PENNCATH	0	0	612	464	0
WTCCC_HT	0	0	649	1198	796
WTCCC_RA	431	739	497	240	0
WTCCC_CHD	461	1457	1218	239	1235
WTCCC_IBD	678	511	678	403	0
TOTAL	10058	13418	11796	6966	11436

Supplementary Methods Table 6: Sample sizes for smoking traits per cohort.

In addition, we undertook a look-up of our novel genome-wide significant signals of association with smoking behaviour in the publicly available GWAS data from the Tobacco and Genetics (TAG) [14]consortium. Results from this look-up, and meta-analysis with the results described above, are presented in Supplementary Table 19.

Power Calculations

We undertook power calculations prior to the start of the project based on use of an exome array, as shown in Supplementary Methods Table 7.

Genotyping	Case:control ratio	N of cases (e.g. low FEV_1 group) assayed	Power for OR 2 MAF 1%†	Power for OR 3 MAF 0.3%†	Power for OR 3.5 MAF 0.2%†	Power for OR 4.5 MAF 0.1%†
Exome array	1:1	10,000	>99%	98%	96%	82%

Supplementary Methods Table 7: Power estimates for rare variants with case:control ratio of 1:1. †Calculations assume an additive genetic model (that is the odds ratios of disease are expressed per copy of the risk variant) and a 5% baseline prevalence of disease. OR= odds ratio.

Due to advances in genotyping arrays and reduced costs it became possible to include a genome-wide imputation grid to the custom array in addition to exome array content and other categories of content. Illustrative power calculations for common and low frequency variants are shown in Supplementary Methods Table 8.

Genotyping	Case:control ratio	N of cases assayed	Power for OR 1.6 MAF 2%†	Power for OR 1.3 MAF 5%†	Power for OR 1.25 MAF 10%†	Power for OR 1.15 MAF 40%†
Custom array	1:1	10,000	96%	71%	93%	92%

Supplementary Methods Table 8: Power estimates for low frequency and common variants with case:control ratio of 1:1. †Calculations assume an additive genetic model (that is the odds ratios of disease are expressed per copy of the risk variant) and a 5% baseline prevalence of disease.

Corresponding illustrative power calculations for common and low frequency variants are shown in Supplementary Methods Table 9 for a case-control ratio of 2:1, relevant to comparison of groups from the two extremes of the % predicted FEV_1 distribution.

Genotyping	Case:control ratio	N of cases assayed	Power for OR 1.7 MAF 2%†	Power for OR 1.4 MAF 5%†	Power for OR 1.3 MAF 10%†	Power for OR 1.2 MAF 40%†
Custom array	2:1	10,000	88%	88%	89%	97%

Supplementary Methods Table 9: Power estimates for low frequency and rare variants with case:control ratio 2:1. †Calculations assume an additive genetic model (that is the odds ratios of disease are expressed per copy of the risk variant) and a 5% baseline prevalence of disease.

Analysis to identify whether variants with a high functional score explain the signal.

In order to identify if there were suggestively functional variants which explain the novel and previously reported association signals, association testing was repeated for each novel and previously reported sentinel variant with each nearby functional variant included in the logistic model in turn. To do this, variants within 1 Mb of the sentinel variant and which were in LD with the sentinel variant ($r^2 > 0.3$) and/or had nominal evidence of association ($P < 5 \times 10^{-4}$) were annotated with their functional effect. Variants were annotated using ENSEMBL's Variant Effect Predictor (VEP)[51] and functional effects were predicted with SIFT[52], PolyPhen-2[53], CADD[54], and GWAVA[55] databases. If a variant was annotated as 'deleterious' by SIFT, 'probably damaging' or 'potentially damaging' by PolyPhen-2, had a CADD scaled score ≥ 20 (CADD_PHRED ≥ 20), or had a GWAVA score > 0.5, it was defined as a functional variant.

CADD (Combined Annotation-Dependent Depletion) is a method for integrating many diverse annotations, namely conservation metrics, functional genomic data, transcript information, and protein level scores into a single score for each coding and noncoding variant. Scaled CADD score (CADD_PHRED) ranks each variant relative to all possible substitutions of the human genome (~8.6 billion SNVs of the GRCh37/hg19 reference genome). A scaled CADD score of greater or equal to 20 indicates the 1% most deleterious variants in the human genome.

GWAVA (genome-wide annotation of variants) is a tool that combines information from a wide range of annotations to predict the functional impact of noncoding variants. We used a GWAVA score threshold of 0.5, as proposed by the authors[55], above which noncoding variants were considered as 'deleterious'.

Annotation results were filtered with VEP's --pick flag, which selects only one consequence per variant based on the canonical, biotype status and length of the transcript as well as the ranking of the consequence type. For variants with multiple annotations, we selected the most deleterious annotation (i.e. if a variant was annotated as frameshift variant and intronic variant, the variant was considered to be frameshift).

The association of the sentinel variant was identified as being explained by a functional variant if the P value for the sentinel variant was > 0.01 in the joint association test. Results are in Supplementary Table 21.

Gene-based analysis of rare and low-frequency variants (MAF < 5%) using SKAT-O

ENSEMBL's Variant Effect Predictor (VEP) was also used to annotate genotyped variants based on the ENSEMBL version transcript set[51]. Annotation results were also filtered with VEP's --pick flag, and for variants with multiple annotations, we selected the most deleterious annotation. In total we identified 115,444 variants in the protein coding regions of genes (exonic variants), of which 104,673 variants were annotated as loss of function (LoF) or missense variants.

Gene-based analysis was performed using the optimal unified kernel-based test (SKAT-O)[56], which maximizes power by selecting the best combination of the burden test and the non-burden sequence kernel association test (SKAT). For each FEV_1 comparison and heavy smokers vs never smokers, we ran two SKAT-O tests including two classes of variants: (1) loss of function (LoF) and missense variants with MAF <5%, and (2) LoF and missense variants with MAF <5%, which were predicted by SIFT[52] to be 'deleterious' or by PolyPhen-2[53] to be 'probably damaging' or 'possibly damaging' or variants with CADD scaled (CADD_PHRED) score ≥ 20[54]. Allele frequencies used for the inclusion threshold were estimated based on all 48,943 unrelated UK BiLEVE samples.

For each gene we selected the minimal P value between the two gene-based tests. In total for each comparison we tested, 9,427 genes in the analysis with LoF and missense variants, and 3,393 genes in the analysis with deleterious LoF and missense variants. Genes with less than 3 variants meeting the criteria for inclusion were excluded.

We defined a statistical significance threshold of $P < 3.9 \times 10^{-6}$ (Bonferroni-corrected for 12,820 genes). All analyses included the first ten principal components and pack years (for heavy smokers) as covariates. Missing genotypes of variants were imputed with the average allele frequency of the genotyped individuals. All genes with SKAT-O $P < 10^{-4}$ are reported in Supplementary Table 23.

For each gene with $P < 10^{-4}$, SKAT-O analyses were re-run excluding each variant in turn to identify whether the SKAT-O signal was driven by a single variant (Supplementary Figure 9).

Analysis of the effect of geographical location on novel loci

Rounded East and North home location coordinates were used to assign each individual to a postcode area (the one or two letter sequence at the start of a UK postcode) using the Ordnance Survey tool Code Point Open (http://www.ordnancesurvey.co.uk/business-and-government/products/code-point-open.html). Supplementary Methods Table 10 shows which region each postcode area corresponds to, and the number of unrelated individuals in each region.

Region	Postcodes	UK BiLEVE Individuals
Northern	CA DH DL HG NE SR TS YO	6,018
East & West Ridings	BD DN HD HU HX LS S WF	7,368
North Midland	DE LE LN NG NN	3,361
Eastern	AL CB CM CO EN IG IP LU MK NR PE RM SG SS WD	3
Southeast	BN CT DA GU ME RH TN	431
Southern	BH DT HP OX PO RG SL SO SP	3,418
Southwest	BA BS EX GL GY PL SN TA TQ TR	4,102
Wales	CF LD LL NP SA	2,189
Midlands	B CV DY HR ST SY TF WR WS WV	3,775
Scotland	AB DD DG EH FK G HS IV KA KW KY ML PA PH TD ZE	3,660
London	BR CR E EC HA KT N NW SE SM SW TW UB W WC	5,317
Northwest	BB BL CH CW FY IM L LA M OL PR SK WA WN	8,797

Supplementary Methods Table 10: Allocation of participants to postcode area.

Out of 48,943 individuals, 504 had missing home location coordinates were not mapped. Those in the “Eastern” region were reassigned to their next closest region (two samples were re-assigned to London and one sample was reassigned to the Southeast). The geographical distribution of participants included in UK BiLEVE is shown in Supplementary Methods Figure 13.

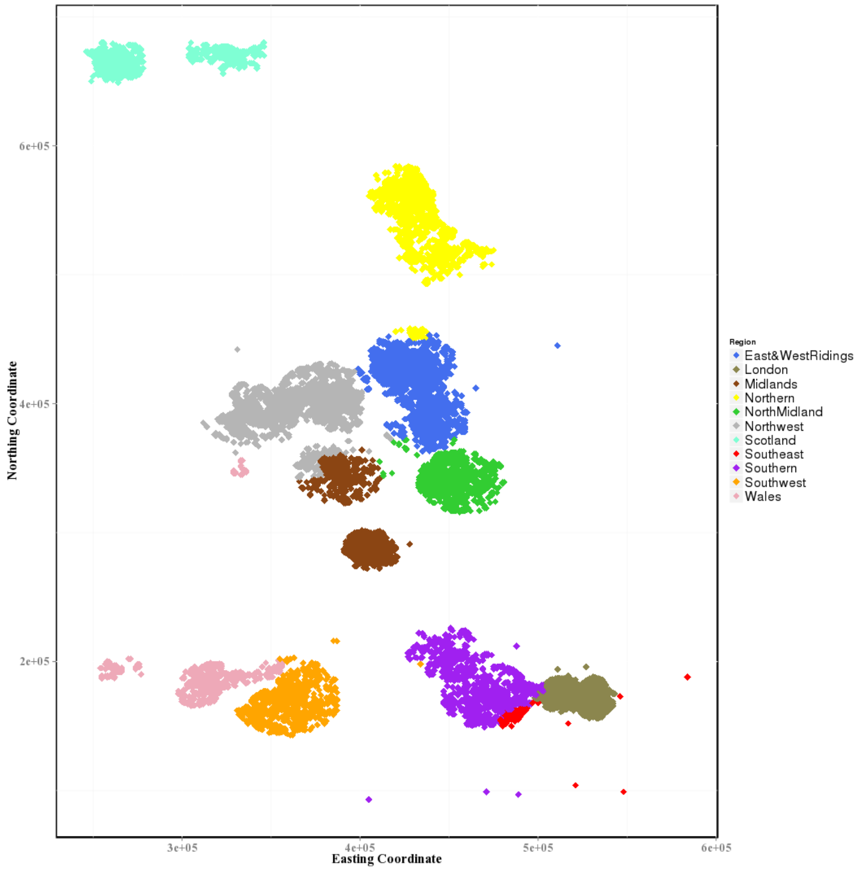

Supplementary Methods Figure 13: Plot of Northing coordinate against Easting coordinate for all participants and coloured according to region as designated by postcode area.

To assess within-UK population structure (manifest as allele frequency variation by geographical region of residence) for selected genetic variants we performed a likelihood ratio test (implemented using the Deducer

package, http://www.deducer.org, in R) which has better statistical properties than a Chi-squared test at low minor allele counts. The likelihood ratio test was performed for the sentinel variants in novel signals of association with extremes of FEV_1 and smoking. As a positive control, we also tested rs9378805 in *IRF4* for association as this SNP showed the strongest evidence of association with geographical location in a previous report[57]. Results are presented in Supplementary Methods Table 11.

Variant	Locus	Chr	Position	Likelihood Ratio Test P value
rs9378805	*IRF4*	*6*	*417727*	*1.78E-15*
rs61784651	*LPPR5*	1	99445471	0.398
rs10193706	*TEX41/PABPC1P2*	2	146316319	0.360
rs2047409	*TET2*	4	106137033	0.011
rs34712979	*NPNT*	4	106819053	0.556
rs9274600	*HLA-DQB1/HLA-DQA2*	6	32635592	0.001
chr11:109843513	*C11orf187/ZC3H12C*	11	109843513	0.190
rs4466874	*NCAM1*	11	112861434	0.165
chr12:114743533	*RBM19/TBX5*	12	114743533	0.335
rs2532349	*KANSL1*	17	44339473	0.401
rs7218675	*TSEN54*	17	73513185	0.340
rs143125561;rs57342388	*C20orf112*	20	31162590	0.039
Affx-89025677		MT	5633	0.001
Affx-89025698		MT	15812	5.06E-06
rs148708877		PAR	2676085	1.12E-09
rs2857319		PAR	2697154	0.020

Supplementary Methods Table 11: Geographical variation of novel loci. Likelihood Ratio Test P values for association of each of the novel loci (for extremes of FEV_1 or smoking behaviour) with geographical region defined by 11 postcode areas. The SNP rs9378805 in *IRF4* was included as a positive control having been previously reported as being strongly associated with geographical location.

Supplementary Tables

Supplementary Table 1: Association with doctor-diagnosed asthma (cases vs controls) for loci previously reported as having genome-wide significant association ($P < 5 \times 10^{-8}$) with asthma in European adults. Consistent: whether detection of effect in this study is consistent with that of previous study (na: direction of effect in previous study could not be determined from the literature).

Reported Gene(s)	chr	position	SNP	P	Consistent
IL6R	1	154426264	rs4129267	6.47E-03	yes
IL1RL1	2	102953617	rs3771180	8.25E-07	na
IL1RL1, IL18R1	2	102955082	rs13408661	2.47E-06	yes
IL18R1	2	102986222	rs3771166	8.38E-05	yes
PDE4D	5	59369794	rs1588265	4.67E-01	no
TSLP	5	110401872	rs1837253	2.24E-10	yes
BTNL2, HLA-DRA	6	32379489	rs9268516	1.78E-08	yes
HLA-DQ	6	32625869	rs9273349	4.88E-16	yes
IL33	9	6190076	rs1342326	9.39E-10	yes
IL33	9	6193455	rs2381416	1.58E-10	yes
LRRC32	11	76270683	rs7130588	1.56E-06	yes
SMAD3	15	67446785	rs744910	3.71E-07	yes
GSDMB	17	38064405	rs11078927	6.67E-11	yes
ORMDL3	17	38069949	rs7216389	2.04E-11	yes
ORMDL3	17	38089344	rs4794820	6.58E-10	na
GSDMA	17	38121993	rs3894194	1.22E-06	yes
IL2RB	22	37534034	rs2284033	4.26E-02	yes

Supplementary Table 2: The genetic architecture of FEV_1-defined traits. Investigations into a) the polygenic architecture of low FEV_1, b) the shared genetic aetiology between high FEV_1 and low FEV_1, c) the shared genetic aetiology of FEV_1 in heavy smokers and never smokers and d) the shared genetic aetiology of FEV_1 in individuals with and without asthma.

a) Investigation of the polygenic architecture of FEV_1-defined traits

	Risk Score			Proportion of variance
**P threshold**	**OR**	**CI**	**P**	**explained**
1	1.125	[1.094, 1.158]	2.19×10^{-16}	0.1425
0.5	1.123	[1.092, 1.155]	6.24×10^{-16}	0.1140
0.4	1.122	[1.091, 1.154]	1.06×10^{-15}	0.1043
0.3	1.120	[1.089, 1.152]	3.72×10^{-15}	0.0950
0.2	1.116	[1.085, 1.148]	1.77×10^{-14}	0.0745
0.1	1.110	[1.079, 1.142]	3.65×10^{-13}	0.0496
0.05	1.105	[1.074, 1.136]	4.04×10^{-12}	0.0313
0.01	1.090	[1.059, 1.121]	2.30×10^{-9}	0.0111
0.001	1.061	[1.032, 1.091]	3.69×10^{-5}	0.0008

b) Investigation of the shared genetic aetiology between high FEV_1 and low FEV_1

	Risk Score			Proportion of variance
**P threshold**	**OR**	**CI**	**P**	**explained**
1	0.883	[0.861, 0.905]	5.34×10^{-23}	0.1480
0.5	0.884	[0.862, 0.906]	1.64×10^{-22}	0.1260
0.4	0.885	[0.864, 0.908]	4.98×10^{-22}	0.1099
0.3	0.886	[0.864, 0.908]	6.84×10^{-22}	0.0934
0.2	0.889	[0.867, 0.911]	1.28×10^{-20}	0.0716
0.1	0.895	[0.873, 0.917]	1.76×10^{-18}	0.0519
0.05	0.901	[0.879, 0.923]	1.28×10^{-16}	0.0411
0.01	0.911	[0.889, 0.934]	1.41×10^{-13}	0.0229
0.001	0.918	[0.896, 0.941]	1.36×10^{-11}	0.0060

c) Investigation of the shared genetic aetiology of FEV1 between heavy and never smokers

	Risk Score			Proportion of variance
**P threshold**	**OR**	**CI**	**P**	**explained**
1	1.128	[1.096, 1.160]	8.42×10^{-17}	0.1787
0.5	1.126	[1.094, 1.158]	2.29×10^{-16}	0.1389
0.4	1.126	[1.095, 1.158]	1.84×10^{-16}	0.1286
0.3	1.123	[1.092, 1.155]	8.12×10^{-16}	0.1124
0.2	1.122	[1.091, 1.154]	1.58×10^{-15}	0.0866
0.1	1.118	[1.087, 1.150]	8.84×10^{-15}	0.0631
0.05	1.107	[1.076, 1.138]	1.82×10^{-12}	0.0387
0.01	1.075	[1.046, 1.106]	4.23×10^{-7}	0.0109
0.001	1.056	[1.027, 1.086]	1.46×10^{-4}	0.0003

d) Investigation of the shared genetic aetiology between individuals with doctor diagnosed asthma and individuals with no doctor diagnosed asthma

	Risk Score			Proportion of variance
P threshold	OR	CI	P	explained
1	1.077	[1.054, 1.101]	3.47×10^{-11}	0.1235
0.5	1.076	[1.053, 1.100]	6.06×10^{-11}	0.1042
0.4	1.077	[1.053, 1.101]	3.80×10^{-11}	0.0903
0.3	1.076	[1.053, 1.100]	5.79×10^{-11}	0.0843
0.2	1.073	[1.049, 1.097]	4.50×10^{-10}	0.0686
0.1	1.073	[1.050, 1.097]	3.49×10^{-10}	0.0466
0.05	1.072	[1.049, 1.096]	5.97×10^{-10}	0.0230
0.01	1.057	[1.034, 1.080]	8.20×10^{-7}	0.0080
0.001	1.042	[1.018, 1.066]	2.16×10^{-4}	0.0026

Supplementary Table 3: Evidence of association with extremes of FEV_1 or smoking behaviour in UK BiLEVE for previously reported lung function (A) and smoking (B) loci. Look up of association results for variants which have been previously reported as showing association with lung function or smoking behaviour, and most strongly associated variant in this study (UK BiLEVE). Where a correlated variant shows stronger association in UK BiLEVE than the previously reported variant, this variant is also included in the table. Strongest previously reported SNP for FEV_1 and/or FEV_1/FVC is from Soler Artigas et al[4]. Strongest previously reported SNPs for smoking behaviour are from three previous reports[12-14]. cor: correlation of coded allele of previously reported SNP and coded allele of UK BiLEVE variant, where applicable. Z score and P for smoking interaction based on comparisons of high FEV_1 vs low FEV_1 in heavy smokers and never smokers is presented. Coded allele, effect estimate and standard error and P value for the previously reported association with FEV_1 (for lung function loci) or smoking behaviour are given. Consistency of direction of effect between UK BiLEVE and the previous report is indicated. se: standard error. *Minor allele. Minor allele count (MAC) in first column is total MAC in all samples; MAC column is MAC for subset of samples included in association of each trait. Full genome-wide association results are available via UK Biobank (access@ukbiobank.ac.uk).

(A) Lung function loci		Lambda	MAF (MAC)	Imputation INFO	beta	se (GC corrected)	OR (95% C.I)	P	Interaction Z score	Interaction P	Previously reported coded allele	MAF (HapMap)	Previously reported FEV_1 effect	Previously reported FEV_1 se	Previously reported P FEV_1	Consistent direction
TNS1	**previously reported FEV_1 rs2571445 (A*/G) MAC: 39561**	Low FEV_1 vs High FEV_1 in heavy smokers														
		1.066	0.402 (11776)	1.000	-0.109	0.026 (0.027)	0.896 (0.850,0.945)	**4.64E-05**	-0.005	0.996	G	0.619	0.047	0.007	9.83E-11	consistent
		Low FEV_1 vs High FEV_1 in never smokers														
		1.097	0.401 (11754)	1.000	-0.109	0.025 (0.027)	0.897 (0.851,0.945)	**4.22E-05**								consistent
		Heavy smokers vs Never smokers														
		1.101	0.400 (39140)	1.000	-0.004	0.013 (0.014)	0.996 (0.969,1.023)	7.47E-01								
	**UK BiLEVE rs918949 (C*/T) MAC: 39776 cor: 0.964**	Low FEV_1 vs High FEV_1 in never smokers														
		1.097	0.402 (11784)	1.000	-0.112	0.025 (0.027)	0.894 (0.848,0.942)	**2.43E-05**								consistent
MECOM	**previously reported FEV_1 rs1344555 (C/T*) MAC: 20116**	Low FEV_1 vs High FEV_1 in heavy smokers														
		1.066	0.206 (6026)	1.000	0.015	0.031 (0.033)	1.015 (0.952,1.082)	6.46E-01	-1.566	0.117	T	0.167	-0.034	0.006	2.65E-08	consistent
		Low FEV_1 vs High FEV_1 in never smokers														
		1.097	0.201 (5900)	1.000	0.087	0.031 (0.033)	1.091 (1.023,1.163)	7.54E-03								consistent
		Heavy smokers vs Never smokers														
		1.101	0.203 (19887)	1.000	0.017	0.016 (0.017)	1.017 (0.984,1.051)	3.21E-01								
GSTCD	**previously reported FEV_1 rs10516526 (A/G*) MAC: 6221**	Low FEV_1 vs High FEV_1 in heavy smokers														
		1.066	0.062 (1831)	1.000	-0.125	0.052 (0.054)	0.883 (0.794,0.981)	2.10E-02	2.863	0.004	G	0.075	0.108	0.014	4.75E-14	consistent
		Low FEV_1 vs High FEV_1 in never smokers														
		1.097	0.064 (1889)	1.000	-0.341	0.050 (0.053)	0.711 (0.641,0.789)	**1.14E-10**								consistent
		Heavy smokers vs Never smokers														
		1.101	0.063 (6147)	1.000	-0.002	0.026 (0.028)	0.998 (0.946,1.054)	9.54E-01								
	**UK BiLEVE rs10516528 (G/T*) MAC: 6118 cor: 0.992**	Low FEV_1 vs High FEV_1 in never smokers														
		1.097	0.063 (1850)	0.992	-0.358	0.051 (0.054)	0.699 (0.629.0.776)	2.14E-11								consistent

(A) Lung function loci			Lambda	MAF (MAC)	Imputation INFO	beta	se (GC corrected)	OR (95% C.I)	P	Interaction Z score	Interaction P	Previously reported coded allele	MAF (HapMap)	Previously reported FEV_1 effect	Previously reported FEV_1 se	Previously reported P FEV_1	Consistent direction
HHIP	**previously reported BOTH rs1032296 (T*/C) MAC: 42539**	Low FEV_1 vs High FEV_1 in heavy smokers	1.066	0.430 (12606)	0.989	-0.134	0.026 (0.027)	0.875 (0.830,0.922)	**6.69E-07**	0.074	0.941	T	0.271	-0.047	0.007	8.74E-11	consistent
		Low FEV_1 vs High FEV_1 in never smokers	1.097	0.432 (12667)	0.989	-0.137	0.025 (0.026)	0.872 (0.829,0.918)	**2.05E-07**								consistent
		Heavy smokers vs Never smokers	1.101	0.430 (42058)	0.989	0.005	0.013 (0.014)	1.005 (0.979,1.033)	6.97E-01								
	**UK BiLEVE rs13107665 (A*/G) MAC: 45630 cor: 0.730**	Low FEV_1 vs High FEV_1 in never smokers	1.097	0.464 (13581)	0.996	-0.159	0.025 (0.026)	0.853 (0.810,0.897)	**8.31E-10**								consistent
HTR4	**previously reported BOTH rs1985524 (G/C*) MAC: 43999**	Low FEV_1 vs High FEV_1 in heavy smokers	1.066	0.443 (12992)	1.000	-0.146	0.026 (0.027)	0.864 (0.820,0.911)	4.57E-08	-1.859	0.063	G	0.608	-0.048	0.007	3.06E-11	consistent
		Low FEV_1 vs High FEV_1 in never smokers	1.097	0.445 (13037)	1.000	-0.077	0.025 (0.026)	0.926 (0.880,0.975)	3.21E-03								consistent
		Heavy smokers vs Never smokers	1.101	0.445 (43550)	1.000	-0.007	0.013 (0.014)	0.993 (0.967,1.019)	5.85E-01								
	**UK BiLEVE rs12374521 (C/T*) MAC: 44705 cor: -0.982**	Low FEV_1 vs High FEV_1 in heavy smokers	1.066	0.451 (13212)	0.994	0.156	0.026 (0.027)	1.169 (1.109,1.232)	5.35E-09								consistent
ZKSCAN3	**previously reported FEV_1 rs6903823 (A/G*) MAC: 24882**	Low FEV_1 vs High FEV_1 in heavy smokers	1.066	0.249 (7310)	1.000	0.117	0.029 (0.030)	1.124 (1.059,1.193)	1.15E-04	-0.182	0.855	G	0.186	-0.037	0.006	2.18E-10	consistent
		Low FEV_1 vs High FEV_1 in never smokers	1.097	0.257 (7530)	1.000	0.125	0.028 (0.030)	1.133 (1.069,1.201)	2.36E-05								consistent
		Heavy smokers vs Never smokers	1.101	0.252 (24621)	1.000	-0.042	0.015 (0.015)	0.959 (0.930,0.988)	6.64E-03								
	**UK BiLEVE rs6904596 (G/A*) MAC: 12798 cor: 0.635**	Low FEV_1 vs High FEV_1 in never smokers	1.097	0.133 (3904)	1.000	0.219	0.037 (0.038)	1.244 (1.154,1.342)	1.17E-08								consistent

(A) Lung function loci		Lambda	MAF (MAC)	Imputation INFO	beta	se (GC corrected)	OR (95% C.I)	P	Interaction Z score	Interaction P	Previously reported coded allele	MAF (HapMap)	Previously reported FEV_1 effect	Previously reported FEV_1 se	Previously reported P FEV_1	Consistent direction
CDC123	**previously reported BOTH rs7068966 (C*/T) MAC: 48191**	Low FEV_1 vs High FEV_1 in heavy smokers														
		1.066	0.487 (14262)	1.000	-0.108	0.025 (0.026)	0.897 (0.853,0.945)	3.41E-05	1.120	0.263	T	0.425	0.029	0.004	2.82E-12	consistent
		Low FEV_1 vs High FEV_1 in never smokers														
		1.097	0.487 (14265)	1.000	-0.150	0.025 0.026)	0.861 (0.818,0.906)	9.56E-09								consistent
		Heavy smokers vs Never smokers														
		1.101	0.487 (47685)	1.000	0.005	0.013 (0.013)	1.005 (0.979.1.032)	6.89E-01								
	**UK BiLEVE rs78420228; rs67863175 (CA*/C) MAC: 43921 cor: 0.902**	Low FEV_1 vs High FEV_1 in never smokers														
		1.097	0.445 (13027)	0.986	-0.169	0.025 (0.026)	0.845 (0.802,0.890)	1.85E-10								consistent
C10orf11	**previously reported FEV_1 rs11001819 (G/A*) MAC: 49040**	Low FEV_1 vs High FEV_1 in heavy smokers														
		1.066	0.495 (14499)	1.000	-0.102	0.025 (0.026)	0.903 (0.858,0.950)	9.44E-05	0.425	0.671	G	0.500	-0.029	0.004	2.98E-12	consistent
		Low FEV_1 vs High FEV_1 in never smokers														
		1.097	0.494 (14459)	1.000	-0.118	0.025 (0.026)	0.889 (0.845,0.935)	6.03E-06								consistent
		Heavy smokers vs Never smokers														
		1.101	0.496 (48534)	1.000	-0.007	0.013 (0.013)	0.993 (0.967,1.020)	6.07E-01								

(B) Smoking loci		Lambda	MAF (MAC)	Imputation INFO	beta	se (GC corrected)	OR (95% C.I)	P	Previously reported coded allele	MAF (HapMap)	Previously reported CPD effect	Previously reported CPD se	Previously reported CPD P
CHRNA3	**Previously reported, cigarettes per day rs1051730 (G/A*) MAC: 34111**	1.101	0.345 (33752)	1.000	0.111	0.014 (0.014)	1.118 (1.087,1.149)	4.43E-15	G	0.660	-0.079	0.005	1.71E-66
	**UK BiLEVE rs71448806 (CGCGGGC/C*) MAC: 40436 cor: 0.857**	1.101	0.409 (40023)	0.994	0.111	0.013 (0.014)	1.118 (1.088,1.148)	6.38E-16					
**7p14**	**Previously reported, cigarettes per day rs215605 (G*/T) MAC: 37443**	1.101	0.379 (37045)	1.000	-0.043	0.013 (0.014)	0.958 (0.932,0.984)	**1.97E-03**	G	0.357	0.260	0.040	5.40E-09
	**UK BiLEVE rs215600 (G*/A) MAC: 35841 cor: 0.930**	1.101	0.362 (35458)	0.998	-0.052	0.013 (0.014)	0.949 (0.923,0.975)	**1.92E-04**					
**8p11**	**Previously reported, cigarettes per day rs13280604 (G*/A) MAC: 21840**	1.101	0.221 (21602)	1.000	0.028	0.015 (0.016)	1.028 (0.996,1.061)	8.71E-02	A	0.784	0.310	0.050	1.30E-08
LOC100188947	**Previously reported, cigarettes per day rs1329650 (G/T*) MAC: 26431**	1.101	0.267 (26139)	1.000	-0.019	0.014 (0.015)	0.981 (0.953,1.011)	2.16E-01	T	0.280	-0.367	0.059	5.67E-10
EGLN2	**Previously reported, cigarettes per day rs3733829 (A/G*) MAC: 35642**	1.101	0.360 (35244)	1.000	0.016	0.013 (0.014)	1.016 (0.989,1.045)	2.46E-01	G	0.360	0.333	0.058	1.04E-08
**19q13**	**Previously reported, cigarettes per day rs7937 (C*/T) MAC: 44394**	1.101	0.449 (43944)	1.000	0.007	0.013 (0.014)	1.007 (0.981,1.034)	5.94E-01	T	0.560	0.240	0.040	2.40E-09

(B) Smoking loci		Lambda	MAF (MAC)	Imputation INFO	beta	se (GC corrected)	OR (95% C.I)	P	Previously reported coded allele	MAF (HapMap)	Previously reported CPD effect	Previously reported CPD se	Previously reported CPD P
DBH	**Previously reported, cigarettes per day rs3025343 (G/A*) MAC: 11809**	1.101	0.119 (11686)	1.000	0.091	0.020 (0.021)	1.095 (1.052,1.141)	1.15E-05	G	0.840	0.121	0.022	3.56E-08
	**UK BiLEVE rs111280114 (A/G*) MAC: 10425 cor: 0.892**	1.101	0.105 (10321)	1.000	0.099	0.021 (0.022)	1.104 (1.058,1.152)	5.98E-06					
BDNF	**Previously reported, cigarettes per day rs6265 (G*/T) MAC: 18402**	1.101	0.186 (18203)	1.000	-0.064	0.017 (0.017)	0.938 (0.907,0.971)	2.36E-04	T	0.210	-0.061	0.011	1.84E-08
	**UK BiLEVE rs2049045 (G*/A) MAC: 18210 cor: 0.799**	1.101	0.184 (18013)	1.000	-0.066	0.017 (0.017)	0.936 (0.905,0.969)	1.55E-04					

Supplementary Table 4: Candidate genes for novel loci associated with extremes of FEV_1 or smoking behaviour. Evidence: indication as to how gene is selected as a candidate gene for this locus including positional evidence (intronic, nearest gene or flanking gene), functional variation explaining the signal (missense signal, functional signal) or eQTL evidence (blood, lung and/or brain cis and trans eQTL evidence for the sentinel variant or proxy variant with $r^2 > 0.8$, indicated by "proxy" in Supplementary Tables 8, 9 and 13). GWAS catalog by variant: any variants with $r^2 \geq 0.8$ with the sentinel variant which have a record in GWAS catalog with $P < 5 \times 10^{-8}$. GWAS catalog by gene: any signals of association ($P < 5 \times 10^{-8}$) in GWAS catalog with any trait. Human Protein Atlas: summary of immunohistochemistry staining of genes in relevant cell types and tissues.

a) low vs high FEV_1 - heavy smokers: chr12:114743533

rsid	name	chr	position	gene	gene name	evidence	Gene function	Known effects on phenotype/ disease	Human Protein Atlas	Knock-out mouse model	GWAS catalog by variant	GWAS catalog by gene
**chr12:114743533**	***RBM19/ TBX5***	12	114743533	*TBX5*	T-box transcription factor 5	nearest gene	Member of the T-Box family contained a common DNA binding domain. Encodes a transcription factor involved in developmental processes.	Involved in development of the heart, lung and trachea[58, 59]. Associated diseases include Holt-Oram syndrome and lung agenesis (failure of organ to develop)[60].	Variable staining in tissues. Moderate cytoplasmic, membranous and nuclear staining in bronchial epithelium. Cytoplasmic and membranous staining in macrophages of the lung. Negative in pneumocytes.	IMPC KO mouse = mouse production planned. TBX5 plays an essential role in lung development of mice[59, 61].	none	Electrocardiographic traits[62], PR interval[63]
				RBM19	probable RNA-binding protein 19	flanking gene	Encodes a nucleolar protein that contains six RNA-binding motifs[64]. May be involved in regulating ribosome biogenesis[65].	Plays a role in embryo pre-implantation development in the mouse[66]. Associated with Holt-Oram and Ulnar-Mammary syndromes[67].	Moderate staining in most tissues. Moderate cytoplasmic, membranous and nuclear staining in bronchial epithelium and macrophages. Strong nuclear staining in pneumocytes.	IMPC KO mouse = ES cells produced and mouse production planned.	none	none

b) **low vs high FEV_1 - never smokers: rs2532349**

rsid	name	chr	position	gene	gene name	evidence	Gene function	Known effects on phenotype/ disease	Human Protein Atlas	Knock-out mouse model	GWAS catalog by variant	GWAS catalog by gene
**rs2532349**	***KANSL1***	17	44339473	*LRRC37A*	Leucine Rich Repeat Containing 37A	nearest gene, lung and blood eQTL regulated gene	Protein coding gene.	Linked to Parkinson's disease risk[68] and dyslexia[69].	Strong expression in testes. Strong staining in cilia/ciliated cells of Bronchus. Negative in pneumocytes and macrophages of lung.	IMPC KO mouse = ES cells produced and mouse production planned.	none	none
				KANSL1 (KIAA1267)	KAT8 Regulatory NSL Complex Subunit 1	flanking gene, lung and blood eQTL regulated gene	Encodes a nuclear protein that is a subunit of two protein complexes involved with histone acetylation, the MLL1 complex and the NSL1 complex. May be involved in the regulation of transcription[70, 71].	Associated diseases include Koolen De Vries syndrome[72], and Kansl1-related intellectual disability syndrome/ADHD[73].	Low to moderate expression in most tissues. Strong membranous, cytoplasmic and moderate nuclear staining in bronchus. Moderate staining in pneumocytes and macrophages of lung	IMPC KO mouse = ES cells produced and mouse produced. No phenotype identified from mouse phenotyping work.	Intracranial volume[74]	Intracranial volume[74]

rsid	name	chr	position	gene	gene name	evidence	Gene function	Known effects on phenotype/ disease	Human Protein Atlas	Knock-out mouse model	GWAS catalog by variant	GWAS catalog by gene
				MAPT	Microtubule-Associated Protein Tau	lung eQTL regulated gene	Transcript undergoes complex, regulated alternative splicing, giving rise to several mRNA species. Transcripts are differentially expressed in the nervous system, depending on stage of neuronal maturation and neuron type. Promotes microtubule assembly and stability, and might be involved in the establishment and maintenance of neuronal polarity[75].	Mutations have been associated with several neurodegenerative disorders such as Alzheimer's disease[76], Pick's disease, frontotemporal dementia[77], cortico-basal degeneration and progressive supranuclear palsy[78].	Strong expression in male reproductive system and Central Nervous System. Negative in pneumocytes and macrophages of the lung.	IMPC KO mouse = ES cells produced and mouse produced. No phenotype identified from mouse phenotyping work.	Parkinson's disease[79-82], interstitial lung disease[83], Progressive supranuclear palsy[84], Idiopathic pulmonary fibrosis[85]	Parkinson's disease[79-82], interstitial lung disease[83], Progressive supranuclear palsy[84], Idiopathic pulmonary fibrosis[85]
				CRHR1	Corticotropin Releasing Hormone Receptor 1	blood eQTL regulated gene	G-protein coupled receptor that binds neuropeptides of the corticotropin releasing hormone family. Major regulators of the hypothalamic-pituitary-adrenal pathway. Essential for the activation of signal transduction pathways that regulate diverse physiological processes including stress, reproduction, immune response and obesity[86].	Required for normal embryonic development of the adrenal gland[87] and for normal hormonal responses to stress[88]. Linked to lung disease and therapy response[89-92].	Not present	IMPC KO mouse = ES cells produced and mouse production in progress.	none	none

rsid	name	chr	position	gene	gene name	evidence	Gene function	Known effects on phenotype/ disease	Human Protein Atlas	Knock-out mouse model	GWAS catalog by variant	GWAS catalog by gene
				LRRC37A4P	Leucine Rich Repeat Containing 37, Member A4, Pseudogene	lung and blood eQTL regulated gene	No gene information.	No gene information.	Not present	No information	none	none
				PLEKHM1	Pleckstrin Homology Domain Containing, Family M (with RUN domain) member 1	lung eQTL regulated gene	Encoded protein is essential for bone resorption, and may play a critical role in vesicular transport in the osteoclast[93].	Mutations in this gene are associated with autosomal recessive osteopetrosis type 6 (OPTB6). Candidate ovarian cancer susceptibilty gene[94].	Moderate to strong expression in most tissues. Strong membranous, and cytoplasmic staining in Bronchus. Moderate staining in pneumocytes and macrophages of lung.	IMPC KO mouse = Mouse produced. Phenotypes identified. Long tibia, Increased bone mineral, increased body fat, increased startle reflex. No lung phenotype stated.	none	Ovarian cancer in BRCA1 mutation carriers[95]
				WNT3	Wingless-Type MMTV Integration Site Family, Member 3	lung eQTL regulated gene	The WNT gene family consists of structurally related genes which encode secreted signaling proteins.<br>Ligand for members of the frizzled family of seven transmembrane receptors.<br>WNT proteins have been implicated in oncogenesis and in several developmental processes, including regulation of cell fate and patterning during embryogenesis.	Studies of the gene expression suggest that this gene may play a key role in some cases of human breast, rectal, lung, and gastric cancer through activation of the WNT-beta-catenin-TCF signaling pathway[96-99]. Diseases associated with WNT3 include tetra-amelia syndrome (viable human but no limbs develop)[100].	Not present	IMPC KO mouse = Mouse produced. Phenotypes identified. Homozygotes lethal following birth. Heterozygotes have decreased body weight, increased energy expenditure, increased oxygen consumption and increased CO2 production. Mouse has limbs. No lung phenotype stated.	Parkinson's disease[101]	Parkinson's disease[101]

rsid	name	chr	position	gene	gene name	evidence	Gene function	Known effects on phenotype/ disease	Human Protein Atlas	Knock-out mouse model	GWAS catalog by variant	GWAS catalog by gene
				ARL17A	ADP-Ribosylation Factor-Like 17A	lung eQTL regulated gene	GTP-binding protein that functions as an allosteric activator of the cholera toxin catalytic subunit, an ADP-ribosyltransferase. Involved in protein trafficking; may modulate vesicle budding and uncoating within the Golgi apparatus.	Copy number varaition linked to dyslexia[69].	Variable staining in tissues. Moderate membranous and cytoplasmic staining in Bronchus. Moderate to low staining in pneumocytes of lung. Macrophages negative.	No information	none	none
				BRWD1 (chr21)	Bromodomain And WD Repeat Domain Containing 1	lung trans eQTL regulated gene	WD repeats are minimally conserved regions of approximately 40 amino acids typically bracketed by gly-his and trp-asp (GH-WD) residues which may facilitate formation of heterotrimeric or multiprotein complexes. Gene is located within the Down syndrome region-2 on chromosome 21.	Mutations in BRWD1 leads to defective spermiogenesis in mice[102].	Moderate/strong staining in most tissues. Strong membranous and cytoplasmic staining in Bronchus. Moderate to strong staining in pneumocytes and macrophages of lung.	IMPC KO mouse = ES cells produced and mouse production in progress.	none	none
				TXNRD1 (chr12)	Thioredoxin Reductase 1	lung trans eQTL regulated gene	Encodes a member of the family of pyridine nucleotide oxidoreductases. Reduces thioredoxins as well as other substrates, plays a role in selenium metabolism, protection against oxidative stress, cell proliferation and transformation.	May be involved in colon[103] and colorectal cancer[104].	Moderate expression in most tissues. Strong in Adrenal gland. Strong cytoplasmic and membranous staining in Bronchus and Macrophages. Low in Pneumocytes.	IMPC KO mouse = ES cells produced and mouse production planned.	none	none

rsid	name	chr	position	gene	gene name	evidence	Gene function	Known effects on phenotype/ disease	Human Protein Atlas	Knock-out mouse model	GWAS catalog by variant	GWAS catalog by gene
				SH3D20 (ARHGAP27)	Rho GTPase Activating Protein 27	blood eQTL regulated gene	Rho GTPase-activating protein which may be involved in clathrin-mediated endocytosis.	Candidate ovarian cancer susceptibilty gene[94].	Most tissues negative. Strong membranous and cytoplasmic staining in goblet cells of Bronchus. Negative in pneumocytes and macrophages of lung.	IMPC KO mouse = ES cells produced and mouse production planned.	none	none
				EPB41L5 (chr2)	Erythrocyte Membrane Protein Band 4.1 Like 5	blood trans eQTL regulated gene	May contribute to the correct positioning of tight junctions during the establishment of polarity in epithelial cells[105].	No known phenotype/human disease.	Variable tissue staining. Strong membranous and cytoplasmic staining bronchus and macrophages of the lung. Pneumocytes negative.	IMPC KO mouse = Mice produced. Many phenotypes identified. Diseases include intussusception (invagination of intestines into another section of intestines) and Diamond Blackfan Anemia. None lung specific.	none	none
				NUDT1 (chr7)	Nudix (Nucleoside Diphosphate Linked Moiety X)-Type Motif 1	blood trans eQTL regulated gene	Oxidative damage repair gene. Antimutagenic. Acts as a sanitizing enzyme for oxidized nucleotide pools, thus suppressing cell dysfunction and death induced by oxidative stress.	Associated diseases include familial adenomatous polyposis[106].	Moderate to strong staining in most tissues. Moderate/Strong membranous and cytoplasmic staining in bronchus, pneumocytes and macrophages.	IMPC KO mouse = ES cells produced and mouse production planned.	none	none
				Other							NSF: Parkinson's disease[81]	na

c) **low vs high FEV_1 - never smokers: rs7218675**

rsid	name	chr	position	gene	gene name	evidence	Gene function	Known effects on phenotype/ disease	Human Protein Atlas	Knock-out mouse model	GWAS catalog by variant	GWAS catalog by gene
**rs7218675**	***TSEN54***	17	73513185	*TSEN54*	TSEN54 TRNA Splicing Endonuclease Subunit	intronic SNP, blood eQTL regulated gene	Encodes a subunit of the tRNA splicing endonuclease complex, which catalyzes the removal of introns from precursor tRNAs. Complex is also implicated in pre-mRNA 3-prime end processing.	Mutations in this gene result in pontocerebellar hypoplasia[107-109].	Staining in most tissues. Moderate membranous and cytoplasmic staining in bronchus. Negative in pneumocytes and macrophages.	IMPC KO mouse = ES cells produced and mouse production planned.	none	none
				GRB2	Growth Factor Receptor-Bound Protein 2	blood eQTL regulated gene, weak lung eQTL regulated gene	Encoded protein binds the epidermal growth factor receptor. Adapter protein that provides a critical link between cell surface growth factor receptors and the Ras signaling pathway[110].	No known phenotype/human disease.	Staining in most tissues. Low membranous , cytoplasmic and nuclear staining in bronchus. Low nuclear staining in pneumocytes. Moderate membranous, cytoplasmic and nuclear staining in macrophages.	IMPC KO mouse = ES cells and mice produced. No phenotyping data available.	none	none

rsid	name	chr	position	gene	gene name	evidence	Gene function	Known effects on phenotype/ disease	Human Protein Atlas	Knock-out mouse model	GWAS catalog by variant	GWAS catalog by gene
				KIAA0195		blood eQTL regulated gene	Protein coding gene.	No known phenotype/human disease.	Variable staining in most tissues. Strong membranous and cytoplasmic staining in bronchus and macrophages. Pneumocytes negative.	IMPC KO mouse = Mice produced. Many phenotypes identified, none lung specific.	none	none

d) **low vs high FEV_1 - never smokers: rs2047409**

rsid	name	chr	position	gene	gene name	evidence	Gene function	Known effects on phenotype/ disease	Human Protein Atlas	Knock-out mouse model	GWAS catalog by variant	GWAS catalog by gene
**rs2047409**	***TET2***	4	106137033	*TET2*	Tet Methylcytosine Dioxygenase 2	intronic SNP, functional signal	Encoded protein plays a key role in active DNA demethylation which is important in transcriptional regulation. Protein is a methylcytosine dioxygenase that catalyzes the conversion of methylcytosine to 5-hydroxymethylcytosine[111].	Involved in myelopoiesis, and gene defects associated with several myeloproliferative disorders, as well as blastic plasmacytoid dendritic cell, and refractory anemia with excess blasts[112]. rs2047409 is weakly correlated with rs6855629, r2=0.35; effect on height is positively correlated with effect on FEV_1[113]	Expressed in every tissue. Strong/Moderate nuclear staining in bronchus, pneumocytes and macrophages.	IMPC KO mouse = ES cells produced and mouse production planned.	(intergenic) Prostate cancer[114]	Height[113, 115], breast cancer[116]
				PPA2	Pyrophosphatase (Inorganic) 2	weak lung eQTL regulated gene	Encoded protein is localized to the mitochondrion. PPases catalyze the hydrolysis of pyrophosphate to inorganic phosphate, which is important for the phosphate metabolism of cells[117].	SNPs in PPA2 associated with response to antipsychotics in Schizophrenia[118].	Moderate staining in most tissues. Moderate membranous and cytoplasmic staining in pneumocytes. Strong staining in bronchus and macrophages.	No information	none	none

e) **heavy vs never smokers: rs4466874**

rsid	name	chr	position	gene	gene name	evidence	Gene function	Known effects on phenotype/ disease	Human Protein Atlas	Knock-out mouse model	GWAS catalog by variant	GWAS catalog by gene
**rs4466874**	***NCAM1***	11	112861434	*NCAM1*	neural cell adhesion molecule 1	intronic SNP, functional signal	Encodes a cell adhesion protein which is a member of the immunoglobulin superfamily. Involved in cell-to-cell interactions as well as cell-matrix interactions during nervous system development and T cell and dendritic cell differentiation[119].	Associated diseases include subcutaneous panniculitis-like t-cell lymphoma and small cell carcinoma[120].	Variable staining in tissues. Strong staining in neuropil of cortex and cells of the granular and molecular layers of the cerebellum. Low cytoplasmic staining in bronchial epithelium. Negative in pneumocytes and macrophages of the lung.	IMPC KO mouse = ES cells produced and mouse production planned.	none	Cardiac muscle measurement[121]
				COL20A1	Collagen, Type XX, Alpha 1	brain trans eQTL regulated gene (all)	Protein coding gene that potentially encodes a collagen protein.	Gene in the breast cancer risk predictive model within the Chinese Han population[122].	Strong expression in Spleen, Bone Marrow and Lymph nodes. Strong nuclear expression in Bronchus and strong nuclear in <50% of Pneumocytes and macrophages. Moderate in neuronal and glial cells of brain cortex	IMPC – ES cells produced and KO mouse in production.	none	none

f) heavy vs never smokers: rs10193706

rsid	name	chr	position	gene	gene name	evidence	Gene function	Known effects on phenotype/ disease	Human Protein Atlas	Knock-out mouse model	GWAS catalog by variant	GWAS catalog by gene
**rs10193706**	***TEX41/ PABPC1P2***	2	146316319	*TEX41*	Testis Expressed 41	nearest gene	Affiliated with the IncRNA class.	No known phenotype/human disease.	Not present	No information	none	none
				PABPC1P2	poly(A) binding protein, cytoplasmic 1 pseudogene 2	flanking gene	Pseudogene.	Associated with Oculopharyngeal Muscular Dystrophy[123].	Not present.	No information	none	none
				DLAT (chr11)	Dihydrolipoamide S-Acetyltransferase	brain trans eQTL regulated gene (substantia nigra)	Encodes component E2 of the multi-enzyme pyruvate dehydrogenase complex (PDC). PDC resides in the inner mitochondrial membrane and catalyzes the conversion of pyruvate to acetyl coenzyme A.	Associated with autoimmune liver disease primary biliary cirrhosis[124]. Mutations also cause pyruvate dehydrogenase E2 deficiency which causes primary lactic acidosis in infancy and early childhood[125].	Moderate to Strong protein expression in most brain cells. Strong cytoplasmic and membranous staining in Bronchus and Pneumocytes. Moderate in Macrophages.	IMPC – ES cells produced, mouse production planned.	none	none

rsid	name	chr	position	gene	gene name	evidence	Gene function	Known effects on phenotype/ disease	Human Protein Atlas	Knock-out mouse model	GWAS catalog by variant	GWAS catalog by gene
				PLUNC (BPIFA1) (chr20)	BPI Fold Containing Family A, Member 1	brain trans eQTL regulated gene (substantia nigra)	Suggested to be involved in inflammatory responses to irritants in the upper airways as expressed in the upper airways and nasopharyngeal regions[126]. Reduces the surface tension in secretions from airway epithelia and inhibits the formation of biofilm by pathogenic Gram-negative bacteria, such as P.aeruginosa[127] and K.pneumoniae[128].	Potentially a molecular marker for detection of micrometastasis in non-small-cell lung cancer[129].	BPIFA1 is negative in every tissue apart from low in epidermal skin cells, low in lung macrophages and moderate in nasopharynx.	IMPC – ES cells produced, mouse production in progress.	none	none
				WDR61 (chr15)	WD Repeat Domain 61	brain trans eQTL regulated gene (substantia nigra)	Subunit of the human PAF and SKI complexes, which function in transcriptional regulation and are involved in events downstream of RNA synthesis, such as RNA surveillance[130, 131].	No known phenotype/human disease.	Low/Moderate protein expression in most tissues. Low in Pneumocytes, moderate cytoplasmic/membranous staining in macrophages and bronchus. Variable staining in brain.	IMPC – ES cells produced, mouse production planned	none	none

rsid	name	chr	position	gene	gene name	evidence	Gene function	Known effects on phenotype/ disease	Human Protein Atlas	Knock-out mouse model	GWAS catalog by variant	GWAS catalog by gene
				PRIC285 (HELZ2) (chr20)	Helicase with Zinc Finger 2, Transcriptional Coactivator	brain trans eQTL regulated gene (substantia nigra)	Encoded protein is a nuclear transcriptional co-activator for peroxisome proliferator activated receptor alpha (PPARalpha)[132].	Possible implicated in the signalling of autoimmune pathogenesis[133].	Low expression in neuropil and glial cells of the brain. Not expressed in lung.	IMPC – ES cells produced, mouse in production	none	none
				ZW10 (chr11)	Zw10 Kinetochore Protein	brain trans eQTL regulated gene (substantia nigra)	Encodes a protein that is one of many involved in mechanisms to ensure proper chromosome segregation during cell division [134]. Essential component of the mitotic checkpoint, which prevents cells from prematurely exiting mitosis. Involved in regulation of membrane traffic between the Golgi and the endoplasmic reticulum[135].	Associated diseases include Roberts syndrome (extremely rare genetic disorder that is characterized by mild to severe prenatal retardation or disruption of cell division, leading to malformation of the bones in the skull, face, arms, and legs)[136].	Variable staining in tissues including brain. Strong cytoplasmic and membranous staining in epithelium of bronchus, Moderate in Pneumocytes and macrophages.	IMPC – ES cells produced, mouse production planned	none	none

g) heavy vs never smokers: rs143125561; rs57342388

rsid	name	chr	position	gene	gene name	evidence	Gene function	Known effects on phenotype/ disease	Human Protein Atlas	Knock-out mouse model	GWAS catalog by variant	GWAS catalog by gene
**rs143125561; rs57342388**	***C20orf112***	20	31162590	*C20orf112 (NOL4L)*	Chromosome 20 Open Reading Frame 112	intronic SNP	Protein coding gene.	Involved in oncogenesis of Leukemia[137].	Variable expression in tissues. Strong in digestive organs. Moderate nuclear and cytoplasmic staining in macrophages. Low in pneumocytes. Moderate in Bronchus.	No information	none	none
				ASXL1	Additional Sex Combs Like 1 (Drosophila)	blood eQTL regulated gene	Encodes a chromatin-binding protein required for normal determination of segment identity in the developing embryo. Protein thought to disrupt chromatin in localized areas, enhancing transcription of certain genes while repressing the transcription of other genes.	Mutations associated with myelodysplastic syndromes and chronic myelomonocytic leukemia[138].	Not present	IMPC KO mouse = Mouse produced. Many phenotypes identified. Altered blood, bone, vision and neurology. No main lung phenotype. Pubmed literature search found ASLX1 (-/-) mice have ventricular septal defects (heart) and have a failure in lung maturation[139].	none	none
				PLAGL2	Pleiomorphic Adenoma Gene-Like 2	brain eQTL regulated gene (cerebellum and all)	Zinc-finger protein that recognizes DNA and/or RNA A surfactant protein C (SP-C) transactivator, in type II cells[140]. Regulates actin cytoskeletal architecture and cell migration[141].	Associated with the development of lung adenocarcinoma and emphysema[142, 143] and colorectal cancer[144]. Regulates Wnt signaling to impede differentiation in neural stem cells and gliomas[145].	Expressed in a variety of tissues, moderate in all brain cells. Moderate cytoplasmic, membranous and nuclear staining in bronchial epithelium. Low cytoplasmic and membranous	IMPC – ES cells produced, mouse production planned.	none	none

rsid	name	chr	position	gene	gene name	evidence	Gene function	Known effects on phenotype/ disease	Human Protein Atlas	Knock-out mouse model	GWAS catalog by variant	GWAS catalog by gene
									staining in Pneumocytes. Strong staining in macrophages.			
				LOC729911		brain trans eQTL regulated gene (all)	RNA gene affiliated with the antisense RNA class.	No known phenotype/human disease.	Not present	No information	none	none

h) **heavy vs never smokers: rs61784651**

rsid	name	chr	position	gene	gene name	evidence	Gene function	Known effects on phenotype/ disease	Human Protein Atlas	Knock-out mouse model	GWAS catalog by variant	GWAS catalog by gene
**rs61784651**	***LPPR5***	1	99445471	*LPPR5 (previously known as PAP2)*	lipid phosphate phosphatase-related protein type 5	intronic SNP	The protein encoded by this gene is a type 2 member of the phosphatidic acid phosphatase (PAP) family. PAPs convert phosphatidic acid to diacylglycerol, and function in de novo synthesis of lycerolipids as well as in receptor-activated signal transduction mediated by phospholipase D[146]. Modulates inflammatory responses in macrophages[147].	No known phenotype/human disease.	Variable staining in tissues. Moderate cytoplasmic and membranous staining in bronchial epithelium, low in macrophages and negative in pneumocytes.	No information	none	none

i) heavy vs never smokers: rs10807199

rsid	name	chr	position	gene	gene name	evidence	Gene function	Known effects on phenotype/ disease	Human Protein Atlas	Knock-out mouse model	GWAS catalog by variant	GWAS catalog by gene
**rs10807199**	***DNAH8***	6	38901867	*DNAH8*	dynein, axonemal, heavy chain 8	intronic SNP	The protein encoded by this gene is a heavy chain of an axonemal dynein involved in sperm and respiratory cilia motility[148]. Axonemal dyneins generate force through hydrolysis of ATP and binding to microtubules.	Potentially involved in myosin storage myopathy and reducing body myopathy.	Expressed in all tissues. Moderate cytoplasmic and membranous staining in bronchus, pneumocytes and macrophages.	No information	none	none

Supplementary Table 5: Analysis of GOLD Stage 2+ COPD. Results of analysis of COPD cases (GOLD Stage 2+ COPD according to spirometry: $FEV_1/FVC < 0.7$ and %predicted $FEV_1 < 80\%$) vs controls from the high FEV_1 strata with $FEV_1/FVC > 0.7$, carried out for SNPS in novel regions identified in the nested lung function comparisons. Minor allele counts (MAC) and frequencies (MAF), odds ratios (OR) and P values (P) are shown for never smokers and heavy smokers separately. Overall odds ratios and P values for never smokers and heavy smokers combined are also shown, calculated using inverse variance weighted meta-analysis.

			**Never smokers (3,761 COPD cases; 4,795 controls)**			**Heavy smokers (5,803 COPD cases; 4,661 controls)**			**Meta-Analysis of never smokers and heavy smokers**	
**Variant ID<br>Locus<br>Position (b37)**	**Noncoded/coded allele<br>*minor allele**	**Imputation INFO**	**MAF (MAC)**	**OR (95% CI)**	**P**	**MAF (MAC)**	**OR (95% CI)**	**P**	**OR (95% CI)**	**P**
chr12:114743533<br>*RBM19/TBX5*<br>Chr12:114743533	T*/C	0.737	0.0020 (33)	1.16 (0.54, 2.51)	0.705	0.0016 (34)	6.44 (2.89, 14.37)	5.40×10^{-06}	2.64 (1.51, 4.60)	6.22×10^{-04}
rs34712979<br>*NPNT*<br>Chr4:106819053	G/A*	1.000	0.2624 (4490)	1.36 (1.27, 1.46)	2.10×10^{-18}	0.2593 (5427)	1.26 (1.18, 1.34)	5.43×10^{-13}	1.31 (1.25, 1.37)	3.04×10^{-29}
rs9274600<br>*HLA-DQB1/HLA-DQA2*<br>Chr6:32635592	A/G*	0.962	0.4679 (8006)	1.24 (1.16, 1.32)	1.95×10^{-11}	0.4711 (9859)	1.08 (1.02, 1.14)	8.58×10^{-03}	1.15 (1.10, 1.20)	9.64×10^{-11}
rs2532349<br>*KANSL1*<br>Chr17:44339473	A/G*	0.976	0.2347 (4017)	1.24 (1.16, 1.34)	3.97×10^{-09}	0.2289 (4791)	1.14 (1.07, 1.22)	9.56×10^{-05}	1.19 (1.13, 1.25)	7.09×10^{-12}
rs7218675<br>*TSEN54*<br>Chr17:73513185	C*/A	0.997	0.2961 (5067)	1.22 (1.14, 1.31)	4.56×10^{-09}	0.2890 (6048)	1.06 (1.00, 1.13)	0.059	1.13 (1.08, 1.18)	8.67×10^{-08}
rs2047409<br>*TET2*<br>Chr4:106137033	G*/A	0.998	0.3528 (6037)	1.17 (1.10, 1.25)	1.64×10^{-06}	0.3562 (7455)	1.09 (1.03, 1.16)	2.92×10^{-03}	1.13 (1.08, 1.18)	5.71×10^{-08}

Supplementary Table 6: Results of stepwise conditional analysis for novel genome-wide significant signals of association with extremes of FEV_1 and smoking behaviour. Beta, se (standard error) and P are the unconditional association results. Beta_j, se_j and P_j are the association results after fitting all variants in a joint model. Beta_glm, se_glm and P_glm are the association results when the joint model was fitted in R with the glm function. The LD (r^2) with the sentinel variant for each stepwise selected variant is shown. Novel independent signals with $P<10^{-4}$ for P_j and P_glm are highlighted in orange. The 3 independent signals at the chromosome 4 *TET2/GSTCD/NPNT* (rs2047409 and rs34712979) locus are highlighted in green. rs10516528 is highly correlated (r^2=0.85) with rs10516526. Analyses were not run for the rare variant signal of association due to unreliability of the method for very low MAFs.

**sentinel**	**Stepwise selected**			**Unconditional**				**Joint conditional**				**Joint R glm**				
**variant**	**SNP**	**bp**	**refA**	**freq**	**beta**	**se**	**P**	**freq_geno**	**beta_j**	**se_j**	**P_j**	**beta_glm**	**se_glm**	**P_glm**	**P/P_j ratio**	**r2_sentinel**
rs7218675	rs7218675	73513185	A	0.291	0.164	0.028	2.40E-09	0.709	0.167	0.029	1.23E-08				0.1950	.
	rs192276566	73771528	C	0.000	-2.562	0.733	4.70E-04	0.000	-2.643	0.754	4.52E-04				1.0396	0.00018
	chr17:74405765	74405765	C	0.001	1.794	0.503	3.62E-04	0.999	1.828	0.533	6.06E-04				0.5985	0.00010
rs2047409	rs34712979	106819053	A	0.268	0.236	0.028	4.30E-17	0.268	0.204	0.030	**5.16E-12**				0.0000	0.00247
	rs10516528	106739593	T	0.063	-0.358	0.051	2.33E-12	0.063	-0.300	0.054	***2.53E-08***				0.0001	0.00129
	rs2047409	106137033	A	0.345	0.155	0.026	2.70E-09	0.655	0.140	0.027	**2.64E-07**				0.0102	.
	chr4:106791232	106791232	T	0.000	2.727	0.754	2.96E-04	1.000	2.715	0.780	5.03E-04				0.5879	0.00002
	chr4:106028230	106028230	G	0.001	1.979	0.520	1.42E-04	0.999	1.914	0.552	5.28E-04				0.2690	0.00063
rs4466874	rs4466874	112861434	C	0.385	0.096	0.013	2.65E-13	0.385	0.098	0.014	**1.81E-12**	0.098	0.013	**8.63E-14**	0.1461	.
	chr11:113786129	113786129	T	0.001	-0.906	0.211	1.85E-05	0.999	-0.929	0.223	**3.11E-05**	-0.997	0.236	**2.39E-05**	0.5959	0.00023
	chr11:112691261	112691261	C	0.000	1.198	0.331	2.99E-04	1.000	1.248	0.368	6.98E-04	1.428	0.404	4.04E-04	0.4282	0.00045
rs10193706	rs10193706	146316319	C	0.473	0.087	0.013	1.28E-11	0.527	0.088	0.014	**2.59E-10**	0.088	0.013	**1.35E-11**	0.0494	.
	rs10928224	145465576	A	0.336	-0.070	0.014	2.16E-07	0.664	-0.068	0.015	**3.09E-06**	-0.068	0.014	**7.92E-07**	0.0699	0.00788
	rs180720480	146135537	A	0.001	0.736	0.195	1.63E-04	0.001	0.733	0.218	7.56E-04	0.771	0.209	2.19E-04	0.2162	0.00004
	rs67969609	145760353	G	0.068	0.049	0.026	5.52E-02	0.067	0.091	0.028	9.77E-04	0.091	0.026	4.36E-04	56.5056	0.02198
rs61784651	rs61784651	99445471	T	0.170	0.099	0.017	5.83E-09	0.170	0.097	0.018	**4.66E-08**	0.098	0.017	**1.12E-08**	0.1253	.
	rs12060706	99256762	T	0.281	-0.059	0.014	3.56E-05	0.281	-0.058	0.015	**9.37E-05**	-0.058	0.014	**4.52E-05**	0.3794	0.00252
	chr1:99617312	99617312	G	0.002	-0.569	0.157	2.79E-04	0.998	-0.621	0.171	2.85E-04	-0.642	0.163	8.50E-05	0.9781	0.00020
rs34712979	rs34712979	106819053	A	0.268	0.236	0.028	4.30E-17	0.268	0.204	0.030	**5.16E-12**				0.0000	.
	rs10516528	106739593	T	0.063	-0.358	0.051	2.33E-12	0.063	-0.300	0.054	**2.53E-08**				0.0001	0.01865
	rs2047409	106137033	A	0.345	0.155	0.026	2.70E-09	0.655	0.140	0.027	**2.64E-07**				0.0102	0.00247
	chr4:106791232	106791232	T	0.000	2.727	0.754	2.96E-04	1.000	2.715	0.780	5.03E-04				0.5879	0.00007
	chr4:106028230	106028230	G	0.001	1.979	0.520	1.42E-04	0.999	1.914	0.552	5.28E-04				0.2690	0.00010

sentinel variant	Stepwise selected SNP	bp	refA	Unconditional freq	beta	se	P	Joint conditional freq_geno	beta_j	se_j	P_j	Joint R glm beta_glm	se_glm	P_glm	P/P_j ratio	r2_sentinel
rs9274600	rs187940467	32631390	C	0.022	-0.082	0.125	5.15E-01	0.004	5.399	0.639	**2.80E-17**	-0.204	0.168	2.25E-01	1.8388E+16	0.00377
	rs144780116	32628903	A	0.004	-0.722	0.205	4.29E-04	0.003	-4.563	0.656	**3.63E-12**	0.435	0.649	5.03E-01	1.1830E+08	0.00414
	rs9274600	32635592	G	0.472	0.169	0.025	1.69E-11	0.473	0.134	0.027	**4.57E-07**	0.144	0.027	**5.74E-08**	0.0000	.
	rs7746553	31895973	G	0.150	-0.161	0.035	3.16E-06	0.150	-0.146	0.035	**2.61E-05**	-0.147	0.034	**1.82E-05**	0.1207	0.00399
	rs76846904	32499917	T	0.004	-0.819	0.213	1.16E-04	0.003	-1.458	0.398	2.52E-04	-0.992	0.648	1.26E-01	0.4598	0.00343
	rs9275601	32682664	T	0.442	0.121	0.025	1.26E-06	0.442	0.091	0.026	4.55E-04	0.101	0.027	1.53E-04	0.0028	0.05115

Supplementary Table 7: Identification of differential gene expression of genes within novel loci associated with extremes of FEV_1 in 38 foetal lungs. Probe ID: Affymetrix Probe ID, Ave expr: average expression for probe during the entire time period, Adj.P.Val: adjusted P value (B-H method) for differential expression over time, beta: mean change in gene expression per day during the studied period (7-22 weeks of gestational age). Based on analysis of the complete dataset, probes with average expression were defined as having a value of between 4.1 and 7.9, low expression was classed as 0-4 and highly expressed probes had an average expression of 8 – 14.

Gene Name	Probe ID	Ave expr	Adj.P.Val	beta	Comment
**RBM19/TBX5**					
RBM19	205115_s_at	5.4781	0.6541	0.0011	Expressed, no change with age
RBM19	206019_at	6.1945	0.7642	-0.0007	Expressed, no change with age
TBX5	1563018_at	5.4798	0.0409	0.0138	Increased expression with increasing fetal lung age
TBX5	207155_at	5.8043	0.0949	0.0062	Expressed, no change with age
TBX5	240715_at	10.0677	0.3837	0.0017	High expression. No change with age
TBX5	211886_s_at	6.6775	0.7701	-0.0013	Expressed, no change with age
**KANSL1**					
ARL17A	232987_at	4.7466	0.0052	0.0055	Increased expression with increasing fetal lung age
ARL17A	210718_s_at	5.1641	0.0610	0.0120	Expressed, no change with age
ARL17A	1554245_x_at	6.3856	0.1773	0.0040	Expressed, no change with age
ARL17A	210435_at	4.0587	0.2412	-0.0033	Expressed, no change with age
ARL17A	1555794_at	3.2139	0.3228	-0.0012	Low expression, no change with age
ARL17A	1555144_at	4.0393	0.4239	0.0016	Expressed, no change with age
ARL17A	1555964_at	4.7879	0.4341	-0.0020	Expressed, no change with age
ARL17A	243899_at	7.1329	0.5856	0.0022	Expressed, no change with age
ARL17A	229028_s_at	6.0402	0.7747	0.0016	Expressed, no change with age
BRWD1	219280_at	5.4423	0.0279	0.0051	Increased expression with increasing fetal lung age
BRWD1	231860_at	5.7359	0.0394	0.0116	Increased expression with increasing fetal lung age
BRWD1	238890_at	5.5859	0.2617	0.0030	Expressed, no change with age
BRWD1	231960_at	5.4966	0.5297	0.0019	Expressed, no change with age
BRWD1	244622_at	5.2654	0.5549	0.0017	Expressed, no change with age
BRWD1	1553227_s_at	5.4699	0.5844	0.0012	Expressed, no change with age
BRWD1	214820_at	6.7721	0.7932	0.0011	Expressed, no change with age
BRWD1	225446_at	6.9943	0.9299	0.0003	High expression. No change with age
CRHR1	211897_s_at	3.9606	0.2496	-0.0024	Low expression, no change with age
CRHR1	214619_at	4.7340	0.6893	0.0006	Expressed, no change with age
CRHR1	208593_x_at	4.1227	0.7354	-0.0008	Expressed, no change with age
EPB41L5	230951_at	5.1632	0.0348	0.0083	Increased expression with increasing fetal lung age
EPB41L5	229292_at	6.9410	0.2008	0.0048	Expressed, no change with age
EPB41L5	220977_x_at	5.2246	0.3537	-0.0024	Expressed, no change with age
EPB41L5	225855_at	8.1580	0.4984	-0.0019	High expression. No change with age
KANSL1 (KIAA1267)	215046_at	7.5284	0.0641	0.0042	Expressed, no change with age
KANSL1 (KIAA1267)	225117_at	9.7108	0.2088	0.0021	High expression. No change with age
KANSL1 (KIAA1267)	1554791_a_at	5.5482	0.3837	0.0018	Expressed, no change with age
KANSL1 (KIAA1267)	231252_at	7.9157	0.6594	0.0024	Expressed, no change with age
KANSL1 (KIAA1267)	230561_s_at	6.4178	0.7479	0.0012	Expressed, no change with age
KANSL1 (KIAA1267)	243589_at	7.2129	0.9961	0.0000	Expressed, no change with age
LRRC37A	220219_s_at	6.3042	0.0004	-0.0091	Decreased expression with increasing fetal lung age
LRRC37A4	220220_at	4.3522	0.0658	-0.0073	Expressed, no change with age

Gene Name	Probe ID	Ave expr	Adj.P.Val	beta	Comment
MAPT	225379_at	6.1239	0.0114	0.0047	Increased expression with increasing fetal lung age
MAPT	233117_at	3.6818	0.0136	0.0029	Increased expression with increasing fetal lung age
MAPT	203928_x_at	5.8884	0.0977	0.0030	Expressed, no change with age
MAPT	206401_s_at	5.2464	0.1315	0.0037	Expressed, no change with age
MAPT	203929_s_at	5.2448	0.4020	-0.0028	Expressed, no change with age
MAPT	203930_s_at	3.9347	0.8177	-0.0004	Low expression, no change with age
NUDT1	204766_s_at	6.6900	0.0000	-0.0183	Decreased expression with increasing fetal lung age
PLEKHM1	216200_at	3.7814	0.0475	0.0039	Increased expression with increasing fetal lung age
PLEKHM1	212700_x_at	4.5515	0.6104	0.0010	Expressed, no change with age
PLEKHM1	212717_at	7.7685	0.9839	0.0000	Expressed, no change with age
SH3D20 (ARHGAP27)	225618_at	6.6592	0.0048	0.0051	Increased expression with increasing fetal lung age
SH3D20 (ARHGAP27)	243536_x_at	4.1868	0.1136	-0.0023	Expressed, no change with age
SH3D20 (ARHGAP27)	1554594_at	3.4714	0.1388	0.0019	Low expression, no change with age
SH3D20 (ARHGAP27)	229424_s_at	5.4093	0.4612	0.0022	Expressed, no change with age
SH3D20 (ARHGAP27)	227057_at	4.9406	0.6682	0.0014	Expressed, no change with age
TXNRD1	201266_at	8.5030	0.0134	-0.0057	Decreased expression with increasing fetal lung age
TXNRD1	1561080_at	3.1134	0.5542	0.0006	Low expression, no change with age
WNT3	229103_at	4.1887	0.0062	-0.0070	Decreased expression with increasing fetal lung age
WNT3	231743_at	5.2558	0.7922	-0.0008	Expressed, no change with age
WNT3	221455_s_at	4.0483	0.8569	-0.0004	Expressed, no change with age
**TET2**					
PPA2	1556285_s_at	8.1827	0.0733	-0.0069	High expression. No change with age
PPA2	1554499_s_at	4.2460	0.0871	-0.0030	Expressed, no change with age
PPA2	220741_s_at	8.7777	0.0909	-0.0038	High expression. No change with age
PPA2	228366_at	5.5152	0.1134	0.0038	Expressed, no change with age
PPA2	1556284_at	3.1119	0.3456	0.0011	Low expression, no change with age
PPA2	1559496_at	8.1297	0.7911	-0.0015	High expression. No change with age
TET2	227624_at	8.4478	0.0066	0.0045	Increased expression with increasing fetal lung age
TET2	235461_at	5.8939	0.5865	0.0038	Expressed, no change with age
TET2	1569385_s_at	5.3304	0.6077	0.0023	Expressed, no change with age
**TSEN54**					
GRB2	223049_at	8.3290	0.7984	-0.0005	High expression. No change with age
GRB2	215075_s_at	7.5928	0.8491	0.0005	Expressed, no change with age
KIAA0195	202650_s_at	6.8187	0.7795	0.0007	Expressed, no change with age
KIAA0195	222210_at	4.7468	0.9630	0.0001	Expressed, no change with age
TSEN54	225879_at	8.1830	0.1662	-0.0023	High expression. No change with age
TSEN54	1558304_s_at	7.3573	0.3183	-0.0018	Expressed, no change with age
TSEN54	241402_at	5.9426	0.4565	-0.0013	Expressed, no change with age

Supplementary Table 8: Associations of all novel genome-wide significant results for all extremes of FEV_1 comparisons and heavy smokers vs never smokers comparison, ordered by significance. Effect allele for all comparisons corresponds to those given in Table 2. MAC: minor allele count. se: standard error. gc: genomic control. OR: odds ratio. Full genome-wide association results are available via UK Biobank (access@ukbiobank.ac.uk).

trait	MAC	lambda	beta	se (gc corrected)	OR	OR lower	OR upper	P (gc corrected)	Z stat smoking interaction	P smoking interaction
**chr12:114743533**										
Low FEV_1 vs High FEV_1 - heavy smokers	39	1.066	2.462	0.431	11.727	5.034	27.319	1.16E-08	4.621	3.83E-06
High FEV_1 vs Average FEV_1 - heavy smokers	49	1.050	-1.353	0.366	0.258	0.126	0.529	2.16E-04		
Heavy vs Never smokers	151	1.101	0.547	0.199	1.728	1.169	2.554	6.12E-03		
Low FEV_1 vs Average FEV_1 - heavy smokers	33	1.033	0.971	0.450	2.642	1.093	6.386	3.10E-02		
Low FEV_1 vs Average FEV_1 - never smokers	71	1.039	-0.341	0.278	0.711	0.412	1.226	2.20E-01		
High FEV_1 vs Average FEV_1 - never smokers	51	1.060	-0.332	0.356	0.718	0.357	1.443	3.52E-01		
Low FEV_1 vs High FEV_1 - never smokers	60	1.095	-0.039	0.326	0.962	0.507	1.824	9.05E-01		
**rs2532349**										
Low FEV_1 vs High FEV_1 - never smokers	7088	1.095	0.196	0.031	1.216	1.145	1.291	1.66E-10	1.453	1.46E-01
Low FEV_1 vs High FEV_1 - heavy smokers	6832	1.066	0.132	0.031	1.141	1.073	1.213	2.71E-05		
High FEV_1 vs Average FEV_1 - never smokers	6873	1.060	-0.127	0.030	0.881	0.830	0.935	3.21E-05		
Heavy vs Never smokers	23158	1.101	-0.053	0.016	0.949	0.919	0.979	9.27E-04		
Low FEV_1 vs Average FEV_1 - never smokers	9676	1.039	0.069	0.024	1.071	1.022	1.123	4.18E-03		
High FEV_1 vs Average FEV_1 - heavy smokers	6639	1.050	-0.081	0.031	0.922	0.868	0.980	9.22E-03		
Low FEV_1 vs Average FEV_1 - heavy smokers	9210	1.033	0.061	0.025	1.062	1.012	1.115	1.43E-02		
**rs7218675**										
Low FEV_1 vs High FEV_1 - never smokers	8538	1.095	0.164	0.029	1.179	1.114	1.247	1.18E-08	3.188	1.43E-03
High FEV_1 vs Average FEV_1 - never smokers	8664	1.060	-0.141	0.028	0.869	0.822	0.918	5.03E-07		
High FEV_1 vs Average FEV_1 - heavy smokers	8536	1.050	-0.043	0.028	0.958	0.907	1.012	1.25E-01		
Low FEV_1 vs High FEV_1 - heavy smokers	8503	1.066	0.034	0.029	1.035	0.978	1.095	2.40E-01		
Low FEV_1 vs Average FEV_1 - never smokers	11055	1.039	0.021	0.023	1.022	0.977	1.069	3.50E-01		
Heavy vs Never smokers	28271	1.101	-0.003	0.015	0.997	0.968	1.026	8.39E-01		
Low FEV_1 vs Average FEV_1 - heavy smokers	11247	1.033	0.003	0.023	1.003	0.959	1.049	8.89E-01		
**rs2047409**										
Low FEV_1 vs High FEV_1 - never smokers	10117	1.095	0.155	0.027	1.168	1.107	1.232	1.31E-08	2.164	3.04E-02
Low FEV_1 vs Average FEV_1 - never smokers	13545	1.039	0.109	0.022	1.115	1.069	1.164	4.65E-07		
Low FEV_1 vs High FEV_1 - heavy smokers	10440	1.066	0.071	0.028	1.074	1.017	1.133	1.02E-02		

trait	MAC	lambda	beta	se (gc corrected)	OR	OR lower	OR upper	P (gc corrected)	Z stat smoking interaction	P smoking interaction
High FEV_1 vs Average FEV_1 - heavy smokers	10528	1.050	-0.054	0.027	0.948	0.899	0.999	4.40E-02		
High FEV_1 vs Average FEV_1 - never smokers	10652	1.060	-0.045	0.027	0.956	0.908	1.007	9.09E-02		
Heavy vs Never smokers	34529	1.101	-0.021	0.014	0.979	0.953	1.007	1.37E-01		
Low FEV_1 vs Average FEV_1 - heavy smokers	13775	1.033	0.010	0.022	1.010	0.968	1.054	6.58E-01		
**rs4466874**										
Heavy vs Never smokers	37709	1.101	0.096	0.014	1.101	1.072	1.131	3.22E-12		
Low FEV_1 vs Average FEV_1 - heavy smokers	15530	1.033	0.045	0.021	1.046	1.004	1.091	3.29E-02		
Low FEV_1 vs High FEV_1 - heavy smokers	11722	1.066	0.034	0.027	1.034	0.981	1.090	2.08E-01	0.354	7.23E-01
Low FEV_1 vs High FEV_1 - never smokers	10968	1.095	0.020	0.027	1.021	0.968	1.076	4.49E-01		
Low FEV_1 vs Average FEV_1 - never smokers	14663	1.039	0.012	0.021	1.012	0.970	1.055	5.86E-01		
High FEV_1 vs Average FEV_1 - heavy smokers	11560	1.050	0.010	0.026	1.010	0.960	1.064	6.89E-01		
High FEV_1 vs Average FEV_1 - never smokers	10977	1.060	-0.009	0.027	0.991	0.940	1.044	7.26E-01		
**rs10193706**										
Heavy vs Never smokers	46280	1.101	0.087	0.013	1.091	1.062	1.120	1.10E-10		
Low FEV_1 vs High FEV_1 - heavy smokers	13575	1.066	0.047	0.026	1.048	0.995	1.104	7.43E-02	1.484	1.38E-01
High FEV_1 vs Average FEV_1 - heavy smokers	13647	1.050	-0.043	0.026	0.958	0.911	1.007	9.44E-02		
High FEV_1 vs Average FEV_1 - never smokers	14258	1.060	0.017	0.026	1.017	0.967	1.069	5.13E-01		
Low FEV_1 vs Average FEV_1 - never smokers	18959	1.039	0.009	0.021	1.009	0.969	1.050	6.78E-01		
Low FEV_1 vs Average FEV_1 - heavy smokers	17984	1.033	0.008	0.021	1.008	0.967	1.050	7.18E-01		
Low FEV_1 vs High FEV_1 - never smokers	14137	1.095	-0.008	0.026	0.992	0.943	1.044	7.59E-01		
**rs143125561;rs57342388**										
Heavy vs Never smokers	22821	1.101	0.094	0.016	1.099	1.065	1.134	4.65E-09		
High FEV_1 vs Average FEV_1 - never smokers	6664	1.060	0.072	0.031	1.075	1.012	1.142	1.93E-02		
Low FEV_1 vs High FEV_1 - never smokers	6640	1.095	-0.068	0.031	0.935	0.879	0.994	3.15E-02	-1.222	2.22E-01
Low FEV_1 vs Average FEV_1 - heavy smokers	9449	1.033	-0.023	0.025	0.977	0.931	1.025	3.44E-01		
Low FEV_1 vs High FEV_1 - heavy smokers	7050	1.066	-0.013	0.031	0.987	0.928	1.049	6.67E-01		
Low FEV_1 vs Average FEV_1 - never smokers	8708	1.039	0.005	0.025	1.005	0.956	1.056	8.47E-01		
High FEV_1 vs Average FEV_1 - heavy smokers	7130	1.050	-0.005	0.030	0.995	0.938	1.056	8.80E-01		
**rs61784651**										
Heavy vs Never smokers	16609	1.101	0.099	0.018	1.104	1.066	1.144	2.89E-08		
High FEV_1 vs Average FEV_1 - never smokers	4789	1.060	0.071	0.035	1.073	1.003	1.148	4.12E-02		
Low FEV_1 vs High FEV_1 - never smokers	4831	1.095	-0.046	0.035	0.955	0.892	1.023	1.89E-01	-0.603	5.46E-01

trait	MAC	lambda	beta	se (gc corrected)	OR	OR lower	OR upper	P (gc corrected)	Z stat smoking interaction	P smoking interaction
Low FEV_1 vs Average FEV_1 - never smokers	6304	1.039	0.026	0.028	1.027	0.972	1.085	3.46E-01		
Low FEV_1 vs High FEV_1 - heavy smokers	5194	1.066	-0.016	0.035	0.984	0.920	1.053	6.41E-01		
High FEV_1 vs Average FEV_1 - heavy smokers	5200	1.050	0.011	0.033	1.011	0.947	1.080	7.37E-01		
Low FEV_1 vs Average FEV_1 - heavy smokers	6900	1.033	0.005	0.027	1.005	0.953	1.060	8.46E-01		
**rs10807199**										
Heavy vs Never smokers	46287	1.101	0.074	0.013	1.077	1.049	1.106	3.17E-08		
Low FEV_1 vs High FEV_1 - heavy smokers	14118	1.066	0.031	0.026	1.032	0.980	1.086	2.34E-01	0.961	3.36E-01
High FEV_1 vs Average FEV_1 - heavy smokers	14149	1.050	-0.024	0.025	0.977	0.929	1.027	3.55E-01		
High FEV_1 vs Average FEV_1 - never smokers	13640	1.060	0.016	0.026	1.016	0.966	1.068	5.41E-01		
Low FEV_1 vs Average FEV_1 - never smokers	18132	1.039	0.012	0.021	1.012	0.971	1.054	5.76E-01		
Low FEV_1 vs Average FEV_1 - heavy smokers	18912	1.033	0.007	0.021	1.007	0.967	1.049	7.40E-01		
Low FEV_1 vs High FEV_1 - never smokers	13623	1.095	-0.004	0.026	0.996	0.946	1.048	8.68E-01		
**rs34712979**										
Low FEV_1 vs High FEV_1 - never smokers	7842	1.095	0.236	0.029	1.266	1.195	1.341	9.62E-16	1.670	9.49E-02
Low FEV_1 vs Average FEV_1 - never smokers	10583	1.039	0.129	0.023	1.138	1.087	1.191	2.90E-08		
Low FEV_1 vs High FEV_1 - heavy smokers	7636	1.066	0.166	0.030	1.180	1.113	1.252	3.11E-08		
High FEV_1 vs Average FEV_1 - heavy smokers	7450	1.050	-0.111	0.029	0.895	0.845	0.948	1.51E-04		
High FEV_1 vs Average FEV_1 - never smokers	7395	1.060	-0.110	0.029	0.896	0.846	0.949	1.96E-04		
Low FEV_1 vs Average FEV_1 - heavy smokers	10380	1.033	0.059	0.023	1.060	1.013	1.110	1.26E-02		
Heavy vs Never smokers	25643	1.101	-0.018	0.015	0.982	0.953	1.012	2.39E-01		
**rs9274600**										
Low FEV_1 vs High FEV_1 - never smokers	13838	1.095	0.169	0.026	1.184	1.125	1.247	1.26E-10	3.346	8.21E-04
High FEV_1 vs Average FEV_1 - never smokers	13571	1.060	-0.099	0.026	0.906	0.860	0.953	1.53E-04		
Low FEV_1 vs Average FEV_1 - never smokers	18680	1.039	0.073	0.021	1.076	1.033	1.121	4.62E-04		
Low FEV_1 vs High FEV_1 - heavy smokers	13719	1.066	0.043	0.027	1.044	0.990	1.101	1.09E-01		
High FEV_1 vs Average FEV_1 - heavy smokers	13669	1.050	-0.027	0.026	0.973	0.925	1.024	2.99E-01		
Heavy vs Never smokers	45913	1.101	-0.014	0.014	0.986	0.960	1.013	3.09E-01		
Low FEV_1 vs Average FEV_1 - heavy smokers	18348	1.033	0.021	0.021	1.021	0.980	1.064	3.23E-01		

Supplementary Table 9: Evidence for the role of novel variants associated with extremes of FEV_1 as eQTLs in lung. Cis and trans results for the most significant variant × probeset pair for any genes (*gene*) identified in the look-up and the results for the sentinel variant and/or strongest proxy variants (*proxy*) are presented. Z.laval, Z.Groningen and Z.UBC are the per-study estimates which were then meta-analysed. *Number of significant SNP x probe pairs for that gene.

cis/trans-gene/proxy	SNP	Alleles	Position (b37)	r^2 with sentinel	Z.Laval	Z.Groningen	Z.UBC	Meta-analysis P	Gene	# significant gene x snp pairs*
**rs2047409 (chr4)**										
cis-gene	rs2647262	T A	106267237	0.307	-5.019	-4.879	-2.611	1.51E-13	PPA2	76
cis-proxy	rs12639764	T C	106216205	0.351	-3.520	-5.319	-1.853	4.00E-10	PPA2	
	rs12639764	T C	106216205	0.351	-3.296	-5.293	-1.555	2.86E-09	PPA2	
**rs2532349 (chr17)**										
cis-gene	rs365825	A G	43705601	0.966	-27.166	-22.177	-21.285	<2.23E-308	KANSL1	31394
	rs365825	A G	43705601	0.966	30.428	20.086	24.790	<2.23E-308	LRRC37A4P	6740
	rs7210219	T C	44018519	0.939	-18.334	-8.181	-10.534	1.10E-113	MAPT	13477
	rs62065436	G A	43556652	0.745	-8.235	-5.380	-9.174	1.15E-40	PLEKHM1	3367
	rs199526	G C	44847707	0.807	-8.788	-5.141	-5.291	2.17E-30	WNT3	3326
	rs79234974	A G	44202467	0.490	-3.429	-3.210	-1.834	8.17E-07	LRRC37A4P	2936
	rs2532349	A G	44339473	1.000	1.975	3.119	3.350	3.32E-06	ARL17A	32
cis-proxy	rs2532349	A G	44339473	1.000	-28.636	-22.764	-22.049	<2.23E-308	KANSL1	
	rs2532349	A G	44339473	1.000	29.915	19.378	23.672	<2.23E-308	LRRC37A4P	
	rs2532349	A G	44339473	1.000	-29.027	-22.855	-21.898	<2.23E-308	KANSL1	
	rs2532349	A G	44339473	1.000	23.490	17.362	20.069	2.15E-266	LRRC37A4P	
	rs2532349	A G	44339473	1.000	-20.351	-17.182	-17.205	3.91E-212	KANSL1	
	rs2532349	A G	44339473	1.000	-19.298	-13.696	-15.431	2.05E-170	KANSL1	
	rs2532349	A G	44339473	1.000	-16.838	-9.067	-11.242	3.70E-108	MAPT	
	rs2532349	A G	44339473	1.000	-17.485	-7.920	-10.393	3.06E-105	MAPT	
	rs2532349	A G	44339473	1.000	-16.214	-8.300	-11.524	1.54E-102	MAPT	
	rs2532349	A G	44339473	1.000	-7.998	-4.287	-8.820	1.91E-36	PLEKHM1	
	rs2532349	A G	44339473	1.000	-6.612	-3.724	-6.957	8.82E-25	MAPT	
	rs2532349	A G	44339473	1.000	-7.866	-4.403	-3.875	5.32E-22	WNT3	
	rs2532349	A G	44339473	1.000	4.292	5.579	4.050	1.24E-14	KANSL1	
	rs2532349	A G	44339473	1.000	1.975	3.119	3.350	3.32E-06	ARL17A	

cis/trans-gene/proxy	SNP	Alleles	Position (b37)	r^2 with sentinel	Z.Laval	Z.Groningen	Z.UBC	Meta-analysis P	Gene	# significant gene x snp pairs*
	rs2532349	A G	44339473	1.000	-3.210	-3.262	-1.739	4.31E-06	LRRC37A	
trans-gene	rs146749482	G A	44773783	0.872	-5.420	-3.449	-2.616	7.96E-12	BRWD1 (chr21)	21
	rs111250307	C T	44357304	0.918	-23.720	-19.632	-24.100	9.88E-324	TXNRD1 (chr12)	10227
trans-proxy	rs2532349	A G	44339473	1.000	-23.510	-19.275	-21.164	4.18E-289	TXNRD1 (chr12)	
**rs7218675 (chr17)**										
cis-gene	rs35584364	C T	73425899	0.328	-7.274	-4.766	-3.654	1.22E-20	GRB2	78
	rs35584364	C T	73425899	0.328	2.050	3.402	3.730	1.96E-07	NT5C	3
	rs35584364	C T	73425899	0.328	2.150	2.831	2.698	1.19E-05	NUP85	1
	rs59541498	A G	73538143	0.473	3.277	2.254	1.905	1.41E-05	SLC25A19	1
cis-proxy	rs9913780	G C	73525684	0.988	4.108	2.346	1.695	1.39E-06	GRB2	
	rs9913780	G C	73525684	0.988	3.670	2.695	1.699	1.95E-06	GRB2	
**rs9274600 (chr6)**										
cis-gene	rs3828791	G A	32635813	0.602	21.021	13.696	13.740	9.68E-173	HLA-DQA2	590
	rs9271376	A G	32587113	0.300	-18.717	-11.311	-12.228	1.37E-126	HLA-DRB5	605
	rs9271376	A G	32587113	0.300	-12.704	-14.080	-13.020	2.43E-110	HLA-DQB1	1638
	rs182983566	A G	32569297	0.562	-16.679	-8.215	-9.058	3.18E-88	HLA-DRB6	562
	rs111831085	G C	32501534	0.332	-11.346	-10.464	-11.557	1.42E-82	HLA-DRB1	675
	rs4947344	C T	32677846	0.359	-12.372	-8.244	-7.760	9.84E-62	HLA-DQB2	482
	rs3129758	G A	32584625	0.508	-6.251	-7.509	-7.492	2.09E-34	AGPAT1	1218
	rs115958783	A G	32585967	0.326	4.596	3.599	5.172	1.91E-14	TAP2	128
	rs78468647	T C	32635197	0.416	5.172	3.885	3.797	1.44E-13	HLA-DPA1	38
	rs9273542	C T	32628812	0.417	-4.077	-4.814	-2.726	1.64E-11	RNF5	100
	rs113742126	A G	32605800	0.385	3.958	2.788	5.031	2.60E-11	APOM	71
	rs2073045	G A	32339548	0.445	-3.871	-3.912	-3.635	4.57E-11	MICA	256
	rs9274497	G A	32633928	0.417	-3.853	-4.275	-1.919	8.75E-09	HLA-DPB1	39
	rs9273529	C T	32628698	0.417	3.023	2.182	3.113	2.15E-06	NOTCH4	9
	rs1980496	C T	32340070	0.380	1.371	2.011	4.705	3.41E-06	BAT2	2
cis-proxy	rs9274600	NA	32635592	1.000	NA	8.321	NA	8.69E-17	HLA-DQB2	
	rs9274600	NA	32635592	1.000	NA	6.278	NA	3.44E-10	AGPAT1	
	rs9274600	NA	32635592	1.000	NA	6.021	NA	1.73E-09	HLA-DQB1	

cis/trans-gene/proxy	SNP	Alleles	Position (b37)	r^2 with sentinel	Z.Laval	Z.Groningen	Z.UBC	Meta-analysis P	Gene	# significant gene x snp pairs*
	rs9274600	NA	32635592	1.000	NA	5.380	NA	7.46E-08	HLA-DRB5	
	rs9274600	NA	32635592	1.000	NA	4.589	NA	4.45E-06	AGPAT1	
	rs9274600	NA	32635592	1.000	NA	-4.498	NA	6.88E-06	HLA-DQA2	
	rs9274600	NA	32635592	1.000	NA	4.454	NA	8.43E-06	HLA-DQB2	
trans-gene	rs113637589	A G	32514026	0.326	23.345	23.671	21.651	<2.23E-308	HLA-DRB3	609
	rs9273241	C T	32614025	0.600	NA	NA	-28.561	2.06E-179	HLA-DRB4	561
	rs78310104	G A	32603742	0.564	-7.031	-2.215	-7.119	4.29E-21	C19orf6 (chr19)	30
	rs9272462	G A	32605620	0.385	5.354	4.653	5.556	6.40E-19	HLA-C	36
	rs9273539	G T	32628779	0.416	NA	-7.706	-4.322	5.98E-18	ZFP57	41
	rs9273539	G T	32628779	0.416	NA	7.299	3.820	1.06E-15	HLA-A	50
	rs75335976	T A	32588416	0.374	-3.715	-4.824	-4.707	2.88E-14	CDSN	278
	rs113637589	A G	32514026	0.326	4.370	3.787	4.702	1.48E-13	ZNF764 (chr16)	1
	rs9273480	C T	32628103	0.413	2.079	4.762	4.683	3.56E-12	BTN3A2	31
	rs9273529	C T	32628698	0.417	3.804	2.867	5.027	1.80E-11	APOM	7
	rs9273539	G T	32628779	0.416	NA	-5.482	-3.855	2.86E-11	HCG4P6	18
trans-proxy	rs9273507	A G	32628432	0.944	NA	-10.343	-9.646	2.10E-45	HLA-DRB3	
	rs9273527	T C	32628621	0.943	NA	-10.305	-9.588	6.19E-45	HLA-DRB3	
	rs9273490	G A	32628193	0.944	NA	-10.211	-9.512	3.00E-44	HLA-DRB3	
	rs9273481	G C	32628122	0.942	NA	-10.139	-9.414	1.63E-43	HLA-DRB3	
	rs9274522	C T	32634373	0.944	-1.027	-7.272	-4.598	3.10E-15	HLA-DRB3	
	rs9274620	G T	32635965	0.945	NA	-5.301	-5.462	9.23E-14	HLA-DRB3	
	rs9274622	T C	32635990	0.945	NA	-5.298	-5.461	9.43E-14	HLA-DRB3	
	rs9274632	G C	32636093	0.945	NA	-5.277	-5.469	1.00E-13	HLA-DRB3	
	rs9274652	C T	32636235	0.944	NA	-5.279	-5.455	1.03E-13	HLA-DRB3	
	rs9274624	G A	32636021	0.945	NA	-5.272	-5.468	1.06E-13	HLA-DRB3	
	rs9274645	G A	32636190	0.945	NA	-5.272	-5.447	1.11E-13	HLA-DRB3	
	rs9274653	T C	32636254	0.944	NA	-5.271	-5.447	1.11E-13	HLA-DRB3	
	rs9274538	A G	32634661	0.942	NA	-6.232	-3.673	3.02E-12	HLA-DRB3	
	rs9273507	A G	32628432	0.944	NA	6.467	2.900	1.18E-11	HLA-A	
	rs9273527	T C	32628621	0.943	NA	6.427	2.869	2.31E-11	HLA-A	

cis/trans-gene/proxy	SNP	Alleles	Position (b37)	r^2 with sentinel	Z.Laval	Z.Groningen	Z.UBC	Meta-analysis P	Gene	# significant gene x snp pairs*
	rs9273507	A G	32628432	0.944	NA	-6.225	-3.016	3.11E-11	ZFP57	
	rs9273490	G A	32628193	0.944	NA	6.287	2.768	5.47E-11	HLA-A	
	rs9273527	T C	32628621	0.943	NA	-6.168	-2.974	6.72E-11	ZFP57	
	rs9273481	G C	32628122	0.942	NA	6.180	2.675	1.51E-10	HLA-A	
	rs9273507	A G	32628432	0.944	NA	4.708	4.341	1.52E-10	BTN3A2	
	rs9273527	T C	32628621	0.943	NA	4.691	4.332	1.72E-10	BTN3A2	
	rs9273490	G A	32628193	0.944	NA	-5.969	-2.840	2.46E-10	ZFP57	

Supplementary Table 10: Evidence for the role of novel variants associated with extremes of FEV_1 or smoking behaviour as eQTLs in blood. a) HapMap imputed resource previously described by Westra et al[39]. Cis and trans results for the most significant variant × probeset pair for any genes (*gene*) identified in the look-up and the results for the sentinel variant and/or strongest proxy variants (*proxy*) are presented. b) 1000 Genomes Project imputed resource from Estonian Genome Project. All variant × probe signals with $P<2.15x10^{-7}$ for 8 loci (see **Supplementary Methods**) are presented. Results for each datasets are presented in the following order: EGCUT;SHIP_TREND;Groningen-HT12;Groningen-H8v2;Rotterdam;DILGOM;INCHIANTI;HVH-v3;HVH-v4 ("-" indicates dataset failed QC). Due to the high-level of correlation of SNPs at 17q21.31 (*KANSL1*), only the strongest eQTL signal for each gene for a proxy of rs2532349 is shown.

A)

gene/ proxy	SNP	position (b37)	r^2 with sentinel	Alleles	Z Score per dataset	Overall Z Score	Overall P	Gene	# significant gene × SNP pairs*
**rs2532349 (chr17)**									
cis-gene	rs10445335	43934896	0.964	T/A	14.82,19.55,23.60,-,14.78,9.81,16.58,-,-	41.387	9.81E-198	MGC57346	223
	rs17426064	43828698	0.964	C/T	8.96,9.38,12.41,7.68,2.35,6.79,9.33,2.41,-	21.758	5.75E-105	CRHR1-IT1	228
	rs2696425	43666906	0.971	G/C	-3.65,-7.06,-8.27,-3.26,-3.49,-2.92,-6.19,-1.95,-	-13.758	4.57E-43	SH3D20	53
	rs4630591	44192568	0.907	C/T	0.15,-6.52,-5.45,-2.89,-6.00,-5.84,-6.06,-,-	-12.202	3.03E-34	KANSL1	397
	rs183211	44788310	0.759	G/A	4.38,3.04,5.31,2.36,4.07,3.46,2.13,1.37,-	9.660	4.45E-22	NSF	44
	rs1635298	43744344	0.852	A/T	-1.52,-2.95,-2.96,-,-3.35,-1.49,-2.37,-1.91,-	-6.188	6.10E-10	LRRC37A4PP,AC091132.16-2,AC091132.16-1	151
	rs10221243	44212310	0.954	G/A	2.66,1.52,1.72,2.35,3.46,-,2.65,-,-	5.598	2.16E-08	LRRC37A,ARL17A	163
	rs199497	44866602	0.330	T/C	-2.15,-0.50,-2.47,0.15,-1.32,-1.08,-1.61,-1.36,-	-3.776	1.59E-04	GOSR2	10
	rs199500	44863413	0.631	C/T	4.04,1.49,2.78,-,-0.11,0.54,-0.37,0.08,-	3.746	1.80E-04	WNT3	1
cis-proxy	rs10445335	43934896	0.964	T/A	14.82,19.55,23.60,-,14.78,9.81,16.58,-,-	41.387	9.81E-198	MGC57346	
	rs17426064	43828698	0.971	C/T	8.96,9.38,12.41,7.68,2.35,6.79,9.33,2.41,-	21.758	5.75E-105	CRHR1-IT1	
	rs2696425	43666906	0.971	G/C	-3.65,-7.06,-8.27,-3.26,-3.49,-2.92,-6.19,-1.95,-	-13.758	4.57E-43	SH3D20	
	rs4630591	44192568	0.907	C/T	0.15,-6.52,-5.45,-2.89,-6.00,-5.84,-6.06,-,-	-12.202	3.03E-34	KANSL1	
	rs17687667	43754099	0.971	G/A	-1.58,-2.98,-2.68,-,-3.10,-1.33,-2.45,-1.87,-	-5.960	2.52E-09	LRRC37A4P,AC091132.16-2,AC091132.16-1	
	rs10221243	44212310	0.954	G/A	2.66,1.52,1.72,2.35,3.46,-,2.65,-,-	5.598	2.16E-08	LRRC37A,ARL17A	
trans-gene	rs393152	43719143	0.964	A/G	2.30,4.61,2.01,-,1.13,0.70,1.88,-1.44,0.97	5.247	1.55E-07	NUDT1	4
	rs415430	44859144	0.818	T/C	-2.78,-2.30,-2.50,-,-1.63,-1.24,-1.31,0.47,-0.94	-4.936	7.99E-07	EPB41L5	1

gene/ proxy	SNP	position (b37)	r^2 with sentinel	Alleles	Z Score per dataset	Overall Z Score	Overall P	Gene	# significant gene × SNP pairs*
trans-proxy	rs393152	43719143	0.964	A/G	2.30,4.61,2.01,-,1.13,0.70,1.88,-1.44,0.97	5.247	1.55E-07	NUDT1	
	rs2942168	43714850	0.971	G/A	2.30,4.61,2.01,-,1.10,0.68,1.88,-1.42,0.98	5.231	1.69E-07	NUDT1	
	rs8070723	44081064	0.970	A/G	2.30,4.58,2.01,-,1.05,0.90,1.75,-1.44,0.97	5.222	1.77E-07	NUDT1	
	rs12185268	43923683	0.971	A/G	2.30,4.52,1.90,-,1.02,0.90,1.88,-1.44,0.97	5.175	2.28E-07	NUDT1	
**rs7218675 (chr17)**									
cis-gene	rs7212620	73461930	0.460	A/T	7.32,6.33,2.71,-,3.77,5.36,4.27,-0.13,-	11.860	1.92E-32	KIAA0195	9
	rs7212620	73461930	0.460	A/T	-4.50,0.03,-5.06,-4.00,-,-4.13,-5.26,-0.20,-	-8.905	5.33E-19	GRB2	10
	rs4789206	73510012	0.555	T/A	4.25,2.23,3.10,2.15,3.43,4.27,1.85,-0.13,-	7.923	2.31E-15	TSEN54	8
	rs7212620	73461930	0.460	A/T	-3.23,-2.76,-3.60,-2.73,-3.01,-1.62,-4.17,0.66,-	-7.851	4.13E-15	MRPS7	4
	rs7212620	73461930	0.460	A/T	-0.36,-1.12,-2.25,-3.37,-,-1.19,-2.87,0.13,-	-4.072	4.67E-05	MIF4GD	1
cis-proxy	rs7218675	73513185	1.000	A/C	5.29,3.80,1.20,-,2.76,3.33,1.58,1.78,-	7.337	2.18E-13	KIAA0195	
	rs7218675	73513185	1.000	A/C	4.56,0.99,1.76,2.00,2.76,3.72,1.68,-0.47,-	6.315	2.69E-10	TSEN54	
	rs7218675	73513185	1.000	A/C	-3.26,0.74,-4.24,-2.44,-,-1.96,-3.40,-0.11,-	-5.811	6.22E-09	GRB2	
**rs9274600 (chr6)**									
cis-gene	rs9272723	32609427	0.605	T/C	9.15,13.72,12.72,2.61,10.36,9.37,4.96,1.15,-	25.039	2.29E-138	HLA-DRB5	5
	rs9272535	32606756	0.315	G/A	12.18,11.68,5.74,-,8.08,1.60,5.19,-,-	18.647	1.33E-77	HLA-DRB6	4
	rs9272535	32606756	0.315	G/A	5.81,8.40,4.09,-,7.15,8.59,1.70,2.11,-	14.468	1.93E-47	HLA-DRA	7
	rs9272723	32609427	0.605	T/C	3.68,1.77,5.20,3.17,5.09,2.94,5.03,0.70,-	10.097	5.70E-24	TAP2	13
	rs9272723	32609427	0.605	T/C	-1.58,-0.43,-4.84,-1.80,-3.49,-3.19,-3.67,-1.01,-	-7.232	4.77E-13	HLA-DOB	1
	rs9272535	32606756	0.315	G/A	1.22,5.66,2.85,-,-,1.55,2.25,3.42,-	6.524	6.86E-11	PSMB9	5
	rs3104405	32682308	0.340	C/A	2.12,5.50,2.71,0.30,0.63,1.53,2.80,0.41,-	6.319	2.63E-10	HLA-DMA	1
	rs7744001	32626086	0.398	G/A	-0.86,-2.64,-1.69,-0.18,-0.83,-1.19,-5.60,1.77,-	-4.782	1.74E-06	PSMB9,TAP1	4
	rs9272535	32606756	0.315	G/A	1.49,2.08,1.58,2.27,0.08,1.08,1.56,1.01,-	3.740	1.84E-04	HLA-DQA2	1
	rs9272346	32604372	0.597	G/A	1.97,0.37,2.29,-,-,1.51,2.05,-,-	3.631	2.83E-04	AL662789.11	1

gene/ proxy	SNP	position (b37)	r^2 with sentinel	Alleles	Z Score per dataset	Overall Z Score	Overall P	Gene	# significant gene × SNP pairs*
cis-proxy	rs6906021	32626311	0.818	T/C	3.41,3.17,4.24,2.55,3.37,2.32,0.81,2.04,-	7.826	5.05E-15	TAP2	
	rs6906021	32626311	0.818	T/C	2.12,2.36,1.62,1.42,4.52,3.01,0.81,-,-	5.929	3.06E-09	HLA-DRA	
	rs6906021	32626311	0.818	T/C	-1.49,-1.43,-1.48,-2.10,-2.15,-0.36,-3.97,1.50,-	-4.535	5.77E-06	TAP2	
	rs6906021	32626311	0.818	T/C	3.23,0.96,1.41,2.22,2.62,1.51,-0.32,2.04,-	4.436	9.17E-06	PSMB9	
trans-gene	rs9272346	32604372	0.597	G/A	-4.86,-8.56,-7.62,-3.24,-5.28,-4.71,-1.98,-2.27,-0.88	-14.420	3.87E-47	LIMS1	3
	rs9272346	32604372	0.597	G/A	-5.59,-4.30,-5.38,-,-,-4.11,-1.78,-,-	-9.648	4.99E-22	U66060.1-23	1
	rs9272346	32604372	0.597	G/A	2.21,0.00,5.56,2.59,4.75,3.05,2.40,0.55,-0.97	7.630	2.36E-14	AOAH	3
	rs9272346	32604372	0.597	G/A	3.89,3.92,2.64,-,-0.14,3.58,4.35,-,-	7.305	2.76E-13	U66061.1-11	1
	rs9272535	32606756	0.315	G/A	-5.68,-1.77,-0.53,-1.93,-2.79,-1.08,-0.05,0.37,-0.80	-5.201	1.99E-07	TRIM56	2
trans-proxy	rs9272346	32604372	0.597	G/A	-4.86,-8.56,-7.62,-3.24,-5.28,-4.71,-1.98,-2.27,-0.88	-14.420	3.87E-47	LIMS1	
	rs9272346	32604372	0.597	G/A	-5.59,-4.30,-5.38,-,-,-4.11,-1.78,-,-	-9.648	4.99E-22	U66060.1-23	
	rs9272346	32604372	0.597	G/A	2.21,0.00,5.56,2.59,4.75,3.05,2.40,0.55,-0.97	7.630	2.36E-14	AOAH	
	rs9272346	32604372	0.597	G/A	3.89,3.92,2.64,-,-0.14,3.58,4.35,-,-	7.305	2.76E-13	U66061.1-11	
**rs143125561;rs57342388 (chr20)**									
cis-gene	rs3746612	31035936	0.353	C/G	4.44,2.48,5.81,2.44,2.21,0.32,3.70,1.76,-	8.584	9.13E-18	ASXL1	69
	rs14353	30922398	0.341	T/C	-0.66,-1.36,-2.85,-1.42,-1.85,-1.08,-2.72,-0.57,-	-4.560	5.12E-06	HCK	26
	rs3787371	30791178	0.315	T/C	2.66,1.21,0.04,2.16,2.29,1.94,2.08,-,-	4.285	1.83E-05	hsa-mir-1825,POFUT1	15
	rs1028563	30918532	0.340	T/C	21.11,14.23,18.43,-,9.60,11.68,10.84,-,-	35.685	9.81E-198	TM9SF4	30
cis-proxy	rs6119904	31161755	0.926	T/C	3.41,1.36,3.10,1.55,0.52,0.25,3.10,0.08,-	5.161	2.45E-07	ASXL1	
	rs4911241	31140165	0.967	T/C	3.41,1.15,3.17,1.78,0.39,0.09,3.07,0.21,-	5.052	4.37E-07	ASXL1	
	rs6141752	31157912	0.989	T/C	3.35,1.21,3.17,1.71,0.55,0.07,3.00,-0.17,-	5.035	4.79E-07	ASXL1	
	rs7268588	31154273	0.987	G/A	3.35,1.18,3.17,1.72,0.55,0.07,3.00,-0.06,-	5.034	4.81E-07	ASXL1	
	rs911527	31165105	0.979	T/C	3.35,1.21,3.17,1.68,0.55,0.05,3.02,-0.29,-	5.019	5.20E-07	ASXL1	
	rs6119897	31145415	0.978	G/A	3.35,1.15,3.14,1.74,0.47,0.07,3.05,0.18,-	5.014	5.32E-07	ASXL1	

gene/ proxy	SNP	position (b37)	r^2 with sentinel	Alleles	Z Score per dataset	Overall Z Score	Overall P	Gene	# significant gene × SNP pairs*
**rs10807199 (chr6)**									
cis-gene	rs9296266	38990614	0.312	A/G	2.60,1.64,2.92,2.00,3.21,3.17,-0.49,1.68,-	5.806	6.41E-09	C6orf64	23
cis-proxy	rs2073037	39014050	0.773	A/G	-2.45,-1.24,-1.44,-0.71,-3.04,-2.05,-0.07,-1.08,-	-4.310	1.63E-05	C6orf64	

B)

gene/ proxy	SNP	position (b37)	r^2 with sentinel	Alleles	Z Score per dataset	beta	P	Gene	Gene chromosome
**rs4466874 (chr11)**									
trans-proxy	rs4937870	112826709	0.428	A/G	na	-0.151	5.10E-08	OR2T33	CHR1

Supplementary Table 11: Imputation of structural haplotypes at 17q21.31 (*KANSL1*) and association with extremes of FEV_1. Genomic regions α, β, and γ are those comprising the structural haplotypes in the 17q21.31 inversion region[47] with their start and end positions. The columns beta, se, OR and P show respectively the fitted effect estimate, its standard error, odds ratio and P value of association for a logistic regression of low FEV_1 versus high FEV_1 with copy number of each genomic region for both heavy and never smokers with 10 ancestry principal components and pack years smoked as covariates (0 for never smokers).

Genomic region	start (hg19)	end (hg19)	beta	se	OR	P
**α**	44212781	44366715	0.157	0.033	1.17	2.40E-06
**β**	44165260	44433878	-0.108	0.044	0.897	0.014
**γ**	44366715	44566776	1.78E-04	0.02	1	0.993

Supplementary Table 12: Loci showing suggestive evidence of association ($P<5x10^{-7}$) with extremes of FEV_1 or smoking behaviour. MAC: Minor Allele Count, MAF: Minor Allele Frequency, INFO: imputation quality score, se: standard error, gc: genomic control, OR: odds ratio, CI: confidence interval. Low minor allele count (MAC < 400) variants are indicated in yellow and the Firth test P value is shown.

Variant	Location	Chr:position (b37)	noncoded/ coded allele (*minor allele)	MAF(MAC)	INFO	beta	se (gc corrected)	OR	OR lower 95% CI	OR upper 95% CI	P (gc corrected)	Firth test P
**Low vs high FEV_1 in heavy smokers**												
rs185224597	MTAP	9:21860063	C/T*	0.004(113.2)	0.710	-1.298	0.250	0.273	0.167	0.446	2.32E-07	3.98E-07
**Low vs High FEV_1 in never smokers**												
rs5772996	WNT4(109kb),ZBTB40(199kb)	1:22578575	T*/TC	0.376(11006.6)	0.996	0.140	0.027	1.150	1.091	1.212	1.72E-07	
rs200840970;rs10709087	CCDC91	12:28597782	A*/AT	0.453(13274.8)	0.993	-0.135	0.026	0.874	0.830	0.919	1.93E-07	
rs11704827	MICAL3	22:18450287	A/T*	0.231(6779.2)	0.981	-0.158	0.031	0.854	0.804	0.907	2.39E-07	
rs56117028	WWP2	16:69884929	T/A*	0.251(7343.1)	0.885	0.163	0.032	1.177	1.106	1.253	3.4E-07	
rs6462481	BBS9	7:33510616	C/T*	0.228(6690.7)	0.988	0.157	0.031	1.170	1.101	1.244	4.04E-07	
rs28540589	LINC00824/LINC00977	8:130116850	A/T*	0.088(2586.3)	0.984	-0.231	0.046	0.794	0.726	0.868	4.38E-07	
rs200179115	DIRC3	2:218196785	AG/A*	0.009(251.3)	0.946	-0.732	0.145	0.481	0.362	0.639	4.4E-07	2.73E-07
rs1635183	THSD7A	7:11683379	C*/G	0.243(7117.9)	0.980	-0.153	0.030	0.858	0.808	0.911	4.47E-07	
**High vs average FEV_1 in heavy smokers**												
chr4:127454100	MIR2054(1026kb),INTU(1100kb)	4:127454100	G*/A	0.001(36.5)	0.751	-2.468	0.446	0.085	0.035	0.203	3.14E-08	3.12E-07
chr13:97824414	OXGR1(178kb),MBNL2(50kb)	13:97824414	A*/C	0.000(11.0)	0.673	-4.587	0.873	0.010	0.002	0.056	1.46E-07	4.71E-07
rs75936762	RSRC1	3:157898693	A/G*	0.301(8850.7)	0.998	-0.144	0.028	0.866	0.820	0.914	2.34E-07	
**High vs average FEV_1 in never smokers**												
chr3:56337829	ERC2	3:56337829	A*/C	0.004(107.5)	0.629	-1.466	0.272	0.231	0.136	0.394	7.1E-08	1.65E-07
rs76993656	LOC340073	5:134571736	G/A*	0.027(794.0)	1.000	0.421	0.078	1.523	1.306	1.776	7.82E-08	
rs199640474	EYS	6:65005227	GA/G*	0.327(9632.0)	0.860	0.154	0.029	1.166	1.102	1.235	1.3E-07	
rs186464237	KCTD16(320kb),PRELID2(962kb)	5:144176946	C/T*	0.008(224.5)	0.975	0.779	0.149	2.180	1.627	2.919	1.71E-07	1.76E-07

Variant	Location	Chr:position (b37)	noncoded/ coded allele (*minor allele)	MAF(MAC)	INFO	beta	se (gc corrected)	OR	OR lower 95% CI	OR upper 95% CI	P (gc corrected)	Firth test P
rs143031547	MIR181A1HG	1: 198840934	G/T*	0.233(23102)	0.983	-0.156	0.030	0.856	0.806	0.908	3.12E-07	
rs168493	ZCCHC6/GAS1	9:89107564	C/T*	0.242(7128.9)	0.981	-0.153	0.030	0.858	0.809	0.910	3.25E-07	
rs273230	PTGFR/IFI44L	1:79071336	A/G*	0.283(8331.1)	0.973	-0.147	0.029	0.864	0.816	0.914	3.35E-07	
**Low vs average FEV_1 in never smokers**												
rs138400467	CSGALNACT1	8:19268195	C/T*	0.015(574.6)	0.893	0.462	0.091	1.587	1.327	1.897	4.14E-07	
rs115559990	HCG4B	6:29894410	C/T*	0.362(14187.6)	0.949	-0.111	0.022	0.895	0.857	0.934	4.55E-07	
**Heavy smokers vs never smokers**												
rs11729080	PITX2(941kb),C4orf32(563kb)	4:112503872	G/A*	0.169(16540.0)	1.000	-0.097	0.018	0.907	0.876	0.940	5.25E-08	
rs13438223	ZNF394	7:99094765	G/A*	0.141(13841.8)	0.986	-0.104	0.019	0.901	0.867	0.936	7.98E-08	
rs11697662	CHRNA4	20:61992005	C*/T	0.195(19101.2)	0.976	-0.091	0.017	0.913	0.882	0.944	1.01E-07	
chr10:15715866	ITGA8	10:15715866	C*/T	0.008(817.2)	0.836	-0.426	0.081	0.653	0.557	0.765	1.4E-07	
rs117238688	TPK1(1261kb),CNTNAP2(19kb)	7:145793961	A/G*	0.026(2576.6)	0.898	0.231	0.044	1.260	1.155	1.374	1.79E-07	
rs12346096	FIBCD1/LAMC3	9:133848437	A/G*	0.158(15442.6)	0.949	0.096	0.019	1.101	1.061	1.143	3.64E-07	
rs114980514	ADCY2	5:7413311	G/A*	0.010(975.3)	0.975	0.347	0.068	1.414	1.237	1.617	3.92E-07	
rs191015772	F3/LINC01057	1:95048850	T/C*	0.004(414.3)	0.779	0.592	0.117	1.808	1.437	2.275	4.47E-07	

Supplementary Table 13: Conditional association results. Association results for variants when conditioned on another variant in order to determine independence of association signal between variants by comparing the unconditional and conditional association results. LD: linkage disequilibrium, MAC: Minor Allele Count, MAF: Minor Allele Frequency, INFO: imputation quality score, se: standard error, gc: genomic control, OR: odds ratio, CI: confidence interval.

For the low FEV_1 vs high FEV_1 – never smokers section, the sentinel variants are conditioned on concurrently or previously reported lung function signals in the same region. For the heavy smokers vs never smokers section the first 3 sentinel variants are secondary novel signals within regions containing a genome-wide significant variant, on which they are conditioned. The remaining variants are conditioned on previously reported smoking behaviour variants within the region.

**Sentinel variant**	**Variant to condition on**								**Conditional association results**				
	**variant**	**reason**	**LD with sentinel (r^2)**	**Position (b37)**	**Distance to sentinel**	**noncoded/ coded allele (*minor allele)**	**MAF (MAC)**	**INFO**	**variant**	**OR**	**OR lower**	**OR upper**	**P**
**Low FEV_1 vs High FEV_1 - never smokers**													
rs34712979	rs6856422	Concurrently reported in 1000G paper	0.305	106,841,962	22,909	G/T*	0.454 (13293)	0.954	rs34712979	1.252	1.171	1.339	4.66E-11
rs2047409	rs10516526	previous lung function signal (Repapi et al.)	0.001	106,688,904	551,871	A/G*	0.064 (1889)	1.000	rs2047409	1.161	1.103	1.222	9.81E-09
rs9274600	rs17843604	proxy for Gabriel SNP rs9273349	0.648	32,620,283	15,309	C*/T	0.406 (11896)	0.998	rs9274600	1.154	1.064	1.252	5.66E-04
rs9274600	rs7764819	previous lung function signal (Hancock et al.)	0.070	32,680,576	44,984	T/G*	0.118 (3453)	0.999	rs9274600	1.186	1.127	1.248	6.71E-11
rs9274600	rs2857595	*NCR3* lung function signal (SpiroMeta-CHARGE)	0.060	31,568,469	1,067,123	G/A*	0.176 (5156)	1.000	rs9274600	1.176	1.118	1.237	3.09E-10
rs9274600	rs2070600	*AGER* lung function signal (SpiroMeta-CHARGE)	0.047	32,151,443	484,149	C/T*	0.064 (1888)	1.000	rs9274600	1.194	1.135	1.255	6.31E-12
rs9274600	rs6903823	*ZKSCAN3* lung function signal (SpiroMeta-CHARGE)	0.031	28,322,296	4,313,296	A/G*	0.257 (7530)	1.000	rs9274600	1.166	1.109	1.226	1.73E-09
**Heavy vs never smokers**													
chr11:113786129	rs4466874	Genome-wide significant signal in region	2.41E-04	112,861,434	924,695	T/C*	0.385 (37710)	0.998	chr11:113786129	0.392	0.259	0.594	9.62E-06
rs10928224	rs10193706	Genome-wide significant signal in region	0.008	146,316,319	850,743	A*/C	0.473 (46280)	0.983	rs10928224	0.939	0.914	0.964	3.95E-06
rs12060706	rs61784651	Genome-wide significant signal in region	0.003	99,445,471	188,709	C/T*	0.170 (16609)	1.000	rs12060706	0.946	0.920	0.973	1.06E-04
rs4466874	rs2303380	*TTC12* nicotine dependence (Gelernter et al.)	1.43E-05	113,200,709	339,275	G*/A	0.373 (36484)	0.989	rs4466874	1.101	1.073	1.130	2.13E-13
rs4466874	rs4938012	*ANKK1* nicotine dependence (Gelernter et al.)	1.44E-04	113,259,654	398,220	A*/G	0.326 (31896)	0.980	rs4466874	1.101	1.073	1.130	2.19E-13

Sentinel variant	Variant to condition on								Conditional association results				
	variant	reason	LD with sentinel (r^2)	Position (b37)	Distance to sentinel	noncoded/ coded allele (*minor allele)	MAF (MAC)	INFO	variant	OR	OR lower	OR upper	P
rs11697662	rs1044394	*CHRNA4* nicotine dependence (Han et al.)	0.018	61,982,085	9,920	A*/G	0.063 (6181)	0.978	rs11697662	0.911	0.882	0.941	1.66E-08
rs11697662	rs2236196	*CHRNA4* tobacco dependence (Hutchison et al. & Li et al.)	0.426	61,977,556	14,449	G*/A	0.250 (24475)	0.979	rs11697662	0.911	0.873	0.950	1.31E-05

Supplementary Table 14: Evidence for the role of novel variants associated with smoking behaviour as eQTLs in brain. Cis and trans results for the most significant variant × probeset pair for any genes (*gene*) identified in the look-up and the results for the sentinel variant and/or strongest proxy variants (*proxy*) are presented. Tissue types: MEDU: medulla (inferior olivary nucleus), THAL: Thalamus, PUTM: Putamen, SNIG: Substantia nigra, FCTX: Frontal cortex, CRBL: Cerebellar cortex, WHMT: Intralobular white matter, TCTX: Temporal cortex, aveALL: average signal across 10 brain regions.

gene/proxy	SNP	position (b37)	r^2 with sentinel	Tissue	Z score	P	Gene	# significant gene × SNP pairs*
**rs4466874 (chr11)**								
cis-gene	rs4937870	112826709	0.428	MEDU	4.775	4.71E-06	USP28	1
cis-proxy	rs4937870	112826709	0.428	MEDU	4.775	4.71E-06	USP28	
trans-gene	rs1245094	112932573	0.505	THAL	-4.966	2.08E-06	C7orf51	32
	rs7937151	112835024	0.982	aveALL	-4.918	2.55E-06	COL20A1	96
	rs4547132	112832813	0.561	PUTM	-4.749	5.26E-06	GIGYF1	1
	rs1447481	112788472	0.394	MEDU	4.714	6.09E-06	GNB2	1
	rs56331084	112957783	0.333	MEDU	-4.649	7.99E-06	RP11-410N8.4	3
	rs3943739	112948688	0.428	MEDU	4.626	8.79E-06	C20orf160	6
trans-proxy	rs4466874	112861434	1.000	aveALL	-4.895	2.82E-06	COL20A1	
**rs10193706 (chr2)**								
trans-gene	rs13030994	146143090	0.565	SNIG	-5.442	2.48E-07	RASGRF1	76
	rs2890772	146175106	0.631	SNIG	5.183	8.00E-07	DLAT	4
	rs10187072	146101224	0.335	SNIG	-5.132	1.00E-06	GMEB2	1
	rs7603916	146372665	0.369	SNIG	5.132	1.00E-06	MEPCE	15
	rs13400519	146249507	0.420	WHMT	5.098	1.17E-06	ZCWPW1	1
	rs13030994	146143090	0.565	SNIG	-4.913	2.61E-06	TM9SF4	35
	rs2890772	146175106	0.631	SNIG	4.860	3.27E-06	WDR61	4
	rs2381726	146133554	0.498	aveALL	-4.766	4.89E-06	KIAA1024	20
	rs1474011	146118069	0.490	aveALL	4.759	5.03E-06	BASE	20
	rs953246	146335486	0.480	MEDU	-4.690	6.72E-06	NCAM1	1
	rs12622738	146258263	0.856	SNIG	-4.679	7.06E-06	PLUNC	1
	rs13410636	146370032	0.369	MEDU	4.634	8.50E-06	TPD52L2	1
	rs12622738	146258263	0.856	SNIG	-4.617	9.12E-06	PRIC285	1
	rs12622738	146258263	0.856	SNIG	4.616	9.18E-06	ZW10	1
trans-proxy	rs12622738	146258263	0.856	SNIG	5.014	1.69E-06	DLAT	
	rs12622738	146258263	0.856	SNIG	-4.679	7.06E-06	PLUNC	
	rs12622738	146258263	0.856	SNIG	4.658	7.68E-06	WDR61	
	rs12622738	146258263	0.856	SNIG	-4.617	9.12E-06	PRIC285	
	rs12622738	146258263	0.856	SNIG	4.616	9.18E-06	ZW10	
**rs143125561;rs57342388 (chr20)**								
cis-gene	rs1555133	31048382	0.460	aveALL	-6.107	1.06E-08	PLAGL2	134
	rs2889678	31189993	0.353	FCTX	4.736	5.54E-06	C20orf160	1
cis-proxy	rs3746615	31123592	0.963	aveALL	-5.685	8.01E-08	PLAGL2	
	rs1108445	31127647	0.964	aveALL	-5.676	8.36E-08	PLAGL2	
	rs4911241	31140165	0.967	aveALL	-5.566	1.40E-07	PLAGL2	
	rs7268588	31154273	0.987	aveALL	-5.531	1.65E-07	PLAGL2	

gene/proxy	SNP	position (b37)	r^2 with sentinel	Tissue	Z score	P	Gene	# significant gene × SNP pairs*
	rs911529	31146939	0.982	aveALL	-5.518	1.75E-07	PLAGL2	
	rs6141752	31157912	0.989	aveALL	-5.511	1.81E-07	PLAGL2	
	rs6141753	31158635	0.988	aveALL	-5.510	1.81E-07	PLAGL2	
	rs6119897	31145415	0.978	aveALL	-5.506	1.85E-07	PLAGL2	
	rs1535374	31162125	0.989	aveALL	-5.498	1.91E-07	PLAGL2	
	rs79943462	31163048	0.992	aveALL	-5.494	1.95E-07	PLAGL2	
	rs201742802	31163047	0.963	aveALL	-5.494	1.95E-07	PLAGL2	
	rs6141755	31163565	0.971	aveALL	-5.417	2.78E-07	PLAGL2	
	rs10875486	31163573	0.980	aveALL	-5.416	2.79E-07	PLAGL2	
	rs911527	31165105	0.979	aveALL	-5.382	3.26E-07	PLAGL2	
	rs34627022	31170277	0.977	aveALL	-5.325	4.22E-07	PLAGL2	
	rs6141759	31177292	0.977	aveALL	-5.225	6.63E-07	PLAGL2	
	rs56827178	31178577	0.946	aveALL	-5.201	7.36E-07	PLAGL2	
	rs6119904	31161755	0.926	aveALL	-5.094	1.19E-06	PLAGL2	
	rs4911102	31179500	0.928	aveALL	-5.071	1.31E-06	PLAGL2	
	rs56827178	31178577	0.946	CRBL	-4.621	8.99E-06	PLAGL2	
	rs6141759	31177292	0.977	CRBL	-4.603	9.66E-06	PLAGL2	
	rs34627022	31170277	0.977	CRBL	-4.603	9.67E-06	PLAGL2	
trans-gene	rs6057602	31181808	0.611	aveALL	-5.399	3.01E-07	LOC729911	5
	rs293566	31097877	0.528	WHMT	-5.026	1.60E-06	AP4M1	2
	rs6087399	31185542	0.431	THAL	-5.002	1.77E-06	ARFRP1	4
	rs2029086	31189411	0.436	aveALL	-4.968	2.05E-06	KCNQ2	5
	rs4578930	31173490	0.626	THAL	4.846	3.47E-06	ZAN	15
	rs4911100	31166129	0.630	TCTX	4.718	5.98E-06	ZFAND3	1
	rs293561	31091206	0.338	THAL	4.640	8.29E-06	DAAM2, LOC100131657	1
trans-proxy	rs3746615	31123592	0.963	aveALL	-4.607	9.52E-06	LOC729911	
**rs10807199 (chr6)**								
trans-gene	rs9394548	38848517	0.459	THAL	4.622	8.93E-06	AP4M1	1
	rs34576374	38739532	0.347	CRBL	-4.802	4.19E-06	C7orf43	2
	rs9394548	38848517	0.459	aveALL	-4.647	8.07E-06	CBFA2T2	1
	rs9394548	38848517	0.459	FCTX	4.896	2.81E-06	COX4I2	1
	rs6940724	38896631	0.443	PUTM	-4.667	7.41E-06	HMG20A	7
	rs7756407	38727453	0.371	THAL	-5.01	1.72E-06	LOC387810	2
	rs9394554	38893153	0.603	TCTX	-4.929	2.44E-06	LPPR4	8
	rs9394548	38848517	0.459	TCTX	-4.673	7.24E-06	TIMM8B	1
	rs862431	38850470	0.313	TCTX	5.094	1.18E-06	TM9SF4	7
	rs9394548	38848517	0.459	TCTX	-4.954	2.19E-06	ZNF394	3
trans-proxy	rs10947762	38888413	0.604	TCTX	-4.907	2.67E-06	LPPR4	

Supplementary Table 15: Genome-wide smoking interaction results. Smoking interaction test P values for variants with P.simple < 5×10^{-7}. The minor allele count (MAC), effect estimates (beta) and P values are shown from the separate never smokers and heavy smokers low FEV_1 vs high FEV_1 association testing. The smoking interaction effect P values are given for the 3 smoking interaction tests used (Supplementary methods): using the Z statistic (P.simple), the Z statistic with Firth test effect estimates and standard errors (P.Firth) and using the likelihood ratio test with a logistic model with a smoking interaction term (P.LRT).

SNP	chr	position	Never smokers			Heavy smokers			Smoking interaction		
			**MAC**	**beta**	**P**	**MAC**	**beta**	**P**	**P.simple**	**P.Firth**	**P.LRT**
chr2:150165243	2	150,165,243	15.061	4.245	4.99E-06	20.315	-1.352	4.55E-02	**3.99E-07**	2.99E-04	3.04E-06
rs7648566	3	185,940,339	10.252	1.599	4.40E-02	6.347	-8.243	5.11E-06	**2.49E-07**	5.15E-03	4.16E-05
rs201609026	4	32,981,056	3.929	1.700	1.70E-01	3.634	-14.007	2.17E-06	**4.02E-07**	3.15E-02	2.64E-04
rs145334889	4	187,285,087	14.383	1.488	3.40E-02	11.708	-5.430	6.82E-06	**2.84E-07**	2.60E-03	5.43E-06
chr5:117057860	5	117,057,860	4.176	12.067	1.39E-06	6.780	-1.776	1.12E-01	**1.32E-07**	1.17E-02	1.72E-04
rs7766366	6	51,712,759	4.256	1.580	1.86E-01	3.020	-16.042	1.16E-06	**2.06E-07**	1.36E-02	1.47E-04
chr6:169329463	6	169,329,463	9457.740	0.130	3.88E-06	9500.730	-0.075	7.74E-03	**8.96E-08**	**8.83E-08**	**1.39E-07**
chr7:152229070	7	152,229,070	3.428	13.620	1.01E-06	5.542	-1.377	2.90E-01	**3.58E-07**	3.29E-02	5.52E-04
rs141001422	11	109,855,881	6.424	-8.910	1.06E-06	9.299	1.267	1.89E-01	**2.71E-07**	4.25E-03	3.31E-04
rs79606326	12	88,384,764	5.966	1.558	1.35E-01	3.102	-13.787	2.85E-06	**3.79E-07**	7.39E-03	1.35E-04
chr16:5616478	16	5,616,478	6.741	7.355	2.40E-06	7.380	-1.319	1.37E-01	**4.62E-07**	4.42E-03	6.36E-05
rs117920382	19	23,709,912	4.983	1.606	1.35E-01	3.103	-12.572	3.15E-06	**4.37E-07**	4.26E-02	3.66E-05
rs2526714	19	43,530,004	12310.600	0.101	1.88E-04	12222.100	-0.087	1.33E-03	**3.34E-07**	2.42E-06	**3.98E-07**
chr21:22306937	21	22,306,937	9.737	-1.282	1.52E-01	6.851	14.773	1.74E-06	**2.49E-07**	9.39E-03	4.40E-03

Supplementary Table 16: Meta-analysis of association results in heavy smokers and never smokers for the comparison of low FEV_1 vs high FEV_1. Previously reported signals (including those reported in the primary analyses in this study) are shaded grey. Genomic control inflation factor (lambda) = 0.9. A look up of results in a previously published GWAS of lung function in the general population[4] is also shown. INFO = imputation quality information. *Reported in Soler Artigas et al (2015)[149].

Sentinel rsid noncoded/coded allele chr:position (b37)	gene	Results for heavy smokers and never smokers separately			Meta-analysis of heavy and never smokers results		Proxy with most signifcant P in SpiroMeta-CHARGE analysis of FEV_1		P of best proxy SNP in SpiroMeta-CHARGE analysis of FEV_1	
		Smoking status	Beta (se)	MAC	Beta (se gc corrected)	P (gc corrected)	Proxy (r^2 with sentinel)	P	Proxy (r^2 with sentinel)	P
rs34712979 G/A	NPNT (intron)	Heavy	0.166(0.031)	7636	0.201(0.022)	2.68E-20				
4:106819053	INFO=1.000	Never	0.236(0.031)	7842						
rs67760252 A/AT	KANSL1 (intron) (17q21.31)	Heavy	0.150(0.034)	6236	0.175(0.024)	1.56E-13				
17:44192592	INFO=0.959	Never	0.199(0.033)	6499						
rs6828982 T/C	GYPA(408.7kb),HHIP-AS1(93.5kb)	Heavy	-0.125(0.027)	13462	-0.142(0.019)	2.02E-13				
4:145470604	INFO=0.997	Never	-0.158(0.027)	13535						
rs4372354 T/C	CDC123 (intron)	Heavy	-0.119(0.028)	14597	-0.143(0.020)	5.21E-13				
10:12288990	INFO=0.934	Never	-0.167(0.028)	14536						
rs13212093 C/T	ZNF184(d165.8kb),LOC100507173(55.1kb)	Heavy	0.173(0.042)	3467	0.192(0.029)	5.35E-11				
6:27606716	INFO=0.999	Never	0.212(0.041)	3621						
rs7715901 A/G	HTR4 (intron)	Heavy	-0.147(0.028)	11575	-0.127(0.020)	8.74E-11				
5:147856392	INFO=0.999	Never	-0.108(0.028)	11566						
rs11704827 A/T	MICAL3 (intron)	Heavy	-0.117(0.033)	6709	-0.138(0.023)	1.75E-09	rs2083882 (0.64)	4.42E-02	rs1076543 (1.00)	2.20E-01
22:18450287	INFO=0.981	Never	-0.158(0.032)	6779						
rs200154334 CAT/C	SPAG17(134.2kb),TBX15(563.6kb)	Heavy	-0.134(0.032)	7260	-0.138(0.023)	1.86E-09	rs2474946 (0.65)	1.46E-02	rs17038164 (0.94)	1.32E-01
1:118862070	INFO=0.948	Never	-0.141(0.032)	7240						
rs9267653 T/C	SLC44A4 (intron)	Heavy	0.111(0.030)	8497	0.124(0.021)	4.36E-09				
6:31840415	INFO=0.990	Never	0.138(0.030)	8323						
rs35337335 GC/G	DARS(78.7kb),CXCR4(50.0kb)	Heavy	0.134(0.032)	7112	0.132(0.022)	4.39E-09	rs2236783 (0.51)	7.79E-04	rs11693502 (0.90)	7.18E-02
2:136821878	INFO=0.984	Never	0.130(0.032)	7264						
rs201043192 AGG/A	HLA-DQB1 (intron)	Heavy	0.059(0.027)	13099	0.114(0.019)	4.56E-09				
6:32628537	INFO=0.981	Never	0.168(0.027)	12973						
rs11001819 G/A	C10orf11 (intron)	Heavy	-0.102(0.027)	14499	-0.110(0.019)	9.59E-09				
10:78315224	INFO=1.000	Never	-0.118(0.027)	14459						
rs139887111 AT/A	TET2 (intron)	Heavy	0.084(0.029)	9580	0.117(0.021)	1.27E-08				
4:106116214	INFO=0.995	Never	0.151(0.029)	9362						
rs200840970 A/AT	CCDC91* (intron)	Heavy	-0.079(0.027)	13424	-0.107(0.019)	2.53E-08	rs7969946 (0.60)	2.02E-05	rs1949978 (0.94)	4.21E-04
12:28597782	INFO=0.993	Never	-0.135(0.027)	13275						
rs7652294 G/T	SLMAP (intron)	Heavy	-0.122(0.032)	7146	-0.124(0.022)	2.58E-08	rs4568105 (0.75)	9.72E-05	rs9848092 (0.96)	1.10E-04
3:57869990	INFO=0.996	Never	-0.127(0.031)	7145						

Sentinel rsid noncoded/coded allele chr:position (b37)	gene	Results for heavy smokers and never smokers separately			Meta-analysis of heavy and never smokers results		Proxy with most signifcant P in SpiroMeta-CHARGE analysis of FEV_1		P of best proxy SNP in SpiroMeta-CHARGE analysis of FEV_1	
		**Smoking status**	**Beta (se)**	**MAC**	**Beta (se gc corrected)**	**P (gc corrected)**	**Proxy (r^2 with sentinel)**	**P**	**Proxy (r^2 with sentinel)**	**P**
rs2571445 A/G	TNS1 (exon)	Heavy	-0.109(0.028)	11776	-0.109(0.020)	2.65E-08				
2:218683154	INFO=1.000	Never	-0.109(0.028)	11754						
rs979012 T/C	FERMT1(519.2kb),BMP2(125.4kb)	Heavy	-0.090(0.028)	10710	-0.109(0.020)	4.62E-08	rs967417 (0.46)	1.49E-02	rs979012 (1.00)	2.00E-02
20:6623374	INFO=0.998	Never	-0.127(0.028)	10762						

Supplementary Table 17: MAGENTA pathway analysis. Results of gene set enrichment analysis (MAGENTA) for genome-wide results from a meta-analysis of low FEV_1 vs high FEV_1 in heavy smokers and never smokers. Only gene sets with false discovery rate > 0.05 are presented. Analyses were run before and after excluding variants within the HLA region. Genes within 500kb of novel (i.e. reported in this paper) and previously reported genome-wide significant signals of association with lung function are flagged. Original gene set size: original number of genes per gene set in publicly available dataset. Effective gene set size: effective number of genes per gene set analysed after removing genes that were not assigned a gene score (e.g. no variants in their region), or after adjusting for physical clustering of genes in a given gene set (removing all but one gene from a subset of genes assigned the same best variant, retaining the gene with the most significant gene score). Gene set enrichment P value: see Supplementary methods for significance threshold for each database. FDR: estimated false discovery rate. The result for the systemic lupus erythematosus pathway following exclusion of HLA genes is shown in italics/grey.

**database**	**gene set**	**original gene set size**	**effective gene set size**	**gene set enrichment P**	**FDR**	**flagged genes**
**<u>all genes</u>**						
PANTHER_MOLECULAR_FUNCTION	Histone	86	35	**6.00E-06**	9.00E-04	HIST1H1B, H3F3B, HIST1H2AL, HIST1H2AM, HIST2H2AC, HIST1H3I, HIST1H3J, HIST1H4L, HIST2H2AB, HIST2H3D
KEGG	KEGG_SYSTEMIC_LUPUS_ERYTHEMATOSUS	140	68	**7.00E-06**	1.30E-03	C2, FCGR1A, GRIN2A, H3F3B, HLA-DMA, HLA-DMB, HLA-DOA, HLA-DOB, HLA-DPA1, HLA-DPB1, HLA-DQA1, HLA-DQA2, HLA-DQB1, HLA-DRA, HLA-DRB1, HLA-DRB5, TNF, HIST1H2AL, HIST1H2AM, HIST2H2AC, HIST1H2BO, HIST2H2BE, HIST1H3I, HIST1H3J, HIST1H4L, HIST2H2AB, HIST2H3D
**<u>HLA genes excluded</u>**						
PANTHER_MOLECULAR_FUNCTION	Histone	86	35	**1.30E-05**	1.10E-03	HIST1H1B, H3F3B, HIST1H2AL, HIST1H2AM, HIST2H2AC, HIST1H3I, HIST1H3J, HIST1H4L, HIST2H2AB, HIST2H3D
GOTERM	positive regulation of MAPKKK cascade	30	28	1.10E-05	9.90E-03	ADRB2
KEGG	KEGG_SYSTEMIC_LUPUS_ERYTHEMATOSUS	140	62	**1.40E-04**	2.13E-02	FCGR1A, GRIN2A, H3F3B, HIST1H2AL, HIST1H2AM, HIST2H2AC, HIST1H2BO, HIST2H2BE, HIST1H3I, HIST1H3J, HIST1H4L, HIST2H2AB, HIST2H3D

Supplementary Table 18: Corroborative evidence in support of the novel signals of association with low FEV_1 vs high FEV_1. Where the novel signal was identified in never smokers (Discovery result), the results for the same SNP are presented for the same comparison (trait) in heavy smokers, and vice versa, in UK BiLEVE. The previous large GWAS comprised data from 48,201 individuals from the general population with ever and never smokers first analysed separately and then meta-analysed: meta-analysis P values for association with FEV_1 are presented for the most significant proxy SNP and the sentinel SNP or best proxy. Minor allele counts (MAC) and frequencies (MAF), odds ratios (OR) and P values (P) are shown.

	UK BiLEVE Discovery results			UK BiLEVE Results from independent subset (i.e. other smoking group)			SpiroMeta-CHARGE (Soler Artigas et al 2011) - Most significant proxy			SpiroMeta-CHARGE (Soler Artigas et al 2011) - Sentinel/best proxy		
**Variant ID**	**Smoking status**	**OR (95% CI)**	**P (gc-corrected)**	**Smoking status**	**OR (95% CI)**	**P (gc-corrected)**	**SNP**	**r^2 with sentinel**	**FEV_1 P**	**SNP**	**r^2 with sentinel**	**FEV_1 P**
rs34712979 (*NPNT*)	Never	1.27 (1.20, 1.34)	9.62×10^{-16}	Heavy	1.18 (1.11, 1.25)	1.10×10^{-8}	none available			none available		
rs9274600 (*HlA-DQB1/ HlA-DQA2*)	Never	1.18 (1.13, 1.25)	1.26×10^{-10}	Heavy	1.05 (1.00, 1.10)	0.096	rs3104405	0.34	3.86×10^{-4}	rs9272723	0.61	5.7×10^{-3}
rs2532349 (*KANSL1*)	Never	1.22 (1.15, 1.29)	1.66×10^{-10}	Heavy	1.15 (1.08, 1.21)	1.47×10^{-05}	rs1358071	0.77	1.42×10^{-5}	rs1358438	0.98	1.82×10^{-4}
rs7218675 (*TSEN54*)	Never	1.18 (1.11, 1.25)	1.18×10^{-08}	Heavy	1.04 (0.98, 1.09)	0.225	rs7212620	0.44	3.89×10^{-4}	rs7218675	1.00	6.13×10^{-3}
rs2047409 (*TET2*)	Never	1.17 (1.11, 1.23)	1.31×10^{-08}	Heavy	1.07 (1.02, 1.13)	8.01×10^{-03}	rs12639764	0.35	9.7×10^{-5}	rs2047409	1.00	9.85×10^{-5}
chr12:114743533 (*RBM19/TBX5*)	Heavy	11.73 (5.03, 27.32)	1.16×10^{-08}	Never	0.97 (0.57, 1.67)	0.901	none available			none available		

Supplementary Table 19: Association with 3 smoking behaviour traits in Oxford-GlaxoSmithKline and Tobacco and Genetics consortia of the 5 novel signals of association with smoking behaviour in UK BiLEVE. N effective: sum of the per-study products of imputation quality and sample size. % N effective is the N effective percentage based on total sample size for each analysis. EAF is effect/coded allele frequency. Results for 4 of the 5 SNPs were not available in the Tobacco and Genetics Consortium, in which case the best tag SNP available was used; effect alleles were aligned to be the positively correlated alleles in the 2 consortia. Inverse-variance weighted meta-analysis results across the 2 consortia are also shown. P < 0.05 are highlighted in bold. Dir = direction of effect in OxGSK and TAG.

		**Oxford-GlaxoSmithkline (OxGSK)**					**Tobacco and Genetics Consortium (TAG)**							**Meta-analysis**			
**Sentinel rsid**	**effect allele**	**N effective (%)**	**EAF**	**beta**	**se**	**P**	**rsid**	**noncoded/ coded allele**	**r^2 with sentinel SNP**	**EAF**	**beta**	**se**	**P**	**beta**	**se**	**P**	**Dir**
**Smoking Initiation**																	
**ever smokers n=13418 vs never smokers n=10058**							**n = 143023**							**n=166499**			
rs4466874	C	23466.34 (99.9)	0.397	0.046	0.020	**0.0229**	rs1940718	C/T	0.997	0.411	0.035	0.012	**0.0036**	0.037	0.010	**0.0003**	+ +
rs10193706	C	21621.43 (92.1)	0.527	0.046	0.020	**0.0235**	rs10193706	A/C	Same SNP	0.518	0.020	0.016	0.2020	0.029	0.012	**0.0170**	+ +
rs143125561	CACGG	22086.39 (94.1)	0.220	0.047	0.024	0.0554	rs6141752	C/T	0.989	0.259	0.040	0.014	**0.0044**	0.041	0.012	**0.0006**	+ +
rs61784651	C	19886.39 (84.7)	0.163	0.029	0.029	0.3136	rs11584061	C/T	0.624	0.862	-0.036	0.018	**0.0483**	-0.017	0.015	0.2542	+ -
rs10807199	T	22189.81 (94.5)	0.426	0.014	0.020	0.4916	rs9462467	A/T	0.998	0.492	0.006	0.012	0.6086	0.008	0.010	0.4299	+ +
**Smoking Cessation**																	
**current smokers n=6966 vs non-current smokers n=11796**							**n = 64924**							**n=83686**			
rs4466874	C	18753.95 (99.6)	0.398	0.059	0.024	**0.0137**	rs1940718	C/T	0.997	0.402	-0.017	0.016	0.2884	0.007	0.013	0.6167	+ -
rs10193706	C	17424.31 (92.9)	0.531	-0.005	0.024	0.8391	rs10193706	A/C	Same SNP	0.513	-0.067	0.020	**0.0006**	-0.042	0.015	**0.0053**	- -
rs143125561	CACGG	17640.32 (94.0)	0.227	-0.053	0.029	0.0646	rs6141752	C/T	0.989	0.240	-0.032	0.019	0.0834	-0.039	0.016	**0.0138**	- -
rs61784651	C	16146.83 (86.1)	0.163	-0.011	0.034	0.7358	rs11584061	C/T	0.624	0.887	-0.015	0.025	0.5365	-0.014	0.020	0.4843	- -
rs10807199	T	16218.21 (86.4)	0.442	-0.021	0.024	0.3696	rs9462467	A/T	0.998	0.489	-0.002	0.016	0.8873	-0.008	0.013	0.5318	- -
**Smoking Quantity**																	
**n=11436**							**n = 73853**							**n=85289**			
rs4466874	C	11432.41 (99.9)	0.403	-0.007	0.012	0.5539	rs1940718	C/T	0.997	0.404	0.103	0.084	0.2185	-0.005	0.012	0.6804	- +
rs10193706	C	10705.08 (93.6)	0.538	-0.010	0.012	0.4220	rs10193706	A/C	Same SNP	0.515	-0.018	0.105	0.8633	-0.010	0.012	0.4137	- -
rs143125561	CACGG	10791.50 (94.4)	0.230	0.009	0.014	0.5169	rs6141752	C/T	0.989	0.241	0.075	0.097	0.4371	0.011	0.014	0.4510	+ +

rs61784651	C	10018.48 (87.6)	0.166	0.009	0.017	0.6110	rs11584061	C/T	0.624	0.887	0.135	0.127	0.2900	0.011	0.017	0.5206	+ +
rs10807199	T	11248.33 (98.4)	0.466	0.002	0.012	0.8380	rs9462467	A/T	0.998	0.490	0.035	0.084	0.6755	0.003	0.012	0.7946	+ +

Supplementary Table 20: Significant associations for mitochondrial (MT) and pseudoautosomal region (PAR) variants with extremes of FEV_1. Significance was defined based on Bonferroni correction for the number of variants analysed on the same chromosome. Chromosome lambda: genomic control lambda for the chromosome with and without exclusion of variants with MAC < 20. Threshold: Bonferroni corrected P value threshold for significance. MAF: minor allele frequency. MAC: minor allele count. se: standard error. OR: odds ratio. 95% CI: 95% confidence interval. P: P value. No chrY variants reached a threshold of 2.02x10-4 (best P value was 2.9x10-2).

**Comparison**	**SNP**	**position**	**noncoded/ coded allele (*minor allele)**	**MAF (MAC)**	**beta**	**se**	**OR (95% CI)**	**P**	**chr lambda (no MAC filter)**	**chr lambda (MAC<20 filtered out)**	**significance threshold**
**Chr MT**											
Low FEV_1 vs High FEV_1 in heavy smokers	Affx-89025677	5633	T*/C	0.008 (224)	0.397	0.104	1.487 (1.213-1.823)	1.37E-04	0.850	0.905	3.57E-04
Low FEV_1 vs High FEV_1 in heavy smokers	Affx-89025698	15812	A*/G	0.008 (244)	0.402	0.099	1.495 (1.231-1.815)	5.07E-05	0.850	0.905	3.57E-04
**Chr PAR**											
Low FEV_1 vs High FEV_1 in heavy smokers	rs148708877	2676085	G*/C	0.305 (8732)	0.152	0.030	1.164 (1.098-1.233)	2.99E-07	0.959	0.957	3.77E-05
Low FEV_1 vs High FEV_1 in heavy smokers	rs2857319	2697154	C*/A	0.273 (7945)	0.156	0.034	1.169 (1.093-1.250)	5.07E-06	0.959	0.957	3.77E-05
Low FEV_1 vs Average FEV_1 in heavy smokers	rs148708877	2676085	G*/C	0.307 (11717)	0.108	0.024	1.114 (1.063-1.167)	5.36E-06	0.921	0.903	3.77E-05

Supplementary Table 21: Putatively functional variants in novel and previously reported regions of association with extremes of FEV_1, lung function or smoking behaviour. Functional variants were defined as variants within 1Mb of the sentinel variant, which were annotated as ‘deleterious’ by SIFT, ‘probably damaging’ or ‘potentially damaging’ by PolyPhen-2, had a CADD scaled score = 20, or had a GWAVA score > 0.5, and were in linkage disequilibrium (LD) with the sentinel variant (r^2 > 0.3) and/or had nominal evidence of association (P < 5×10-4). Regions for which we did not identify any functional variants are not listed in the table. For each region the total number of functional variants identified and the total number of variants explaining the association signal of the sentinel variant is provided. Only functional variants that explained the association of the sentinel variant (sentinel variant P > 0.01 in the conditional analysis) are listed. MAF: minor allele frequency. se: standard error. gc: genomic control. #missense variant classified as “tolerated” (0.1) by SIFT and “probably_damaging” (0.987) by Polyphen. Imputation Panel: + and – indicate the presence or abscence of the variant in each of the HapMap, 1000G and UK10K imputation reference panels. Missense variants are highlighted in red.

A) Novel genome-wide significant variants ($P < 5*10^{-8}$)

										**Conditional Analysis**			
**Sentinel variant**		**Unconditional analysis**		**N functional variants**	**Functional variant**					**Sentinel variant**		**Functional variant**	
**Locus**<br>**rsid**<br>**chr:position (b37)**<br>**noncoded/coded allele**<br>**(*minor allele)**	**MAF**	**beta**<br>**(se)**	**P (not gc-corrected)**	**All identified**<br>**(explain**<br>**the signal)**	**rsid**<br>**noncoded/coded allele**<br>**(*minor allele)**	**LD (r^2)**	**MAF**	**Imputation panel**	**Annotation:**<br>**Consequence**<br>**SYMBOL**<br>**GWAVA score**<br>**CADD scaled score**	**beta**<br>**(se)**	**P**	**beta**<br>**(se)**	**P**
Low FEV_1 vs High FEV_1 in heavy smokers													
RBM19-TBX5<br>chr12:114743533<br>12:114743533<br>C/T*	0.001	2.462<br>(0.418)	3.8E-09	1 (0)	-	-	-	-	-	-	-	-	-
Low FEV_1 vs Average FEV_1 in never smokers													
NPNT<br>rs34712979<br>4:106819053<br>A*/G	0.268	0.236<br>(0.028)	4.3E-17	4 (0)	-	-	-	-	-	-	-	-	-
HLA-DQB1<br>rs9274600<br>6:32635592<br>G*/A	0.472	0.169<br>(0.025)	1.7E-11	137 (0)	-	-	-	-	-	-	-	-	-
KANSL1<br>rs2532349<br>17:44339473<br>G*/A	0.242	0.196<br>(0.029)	2.3E-11	95 (4)	rs117671932<br>G*/T	0.97	0.229	"-+-"	upstream<br>*RP11-259G18.3*<br>0.51<br>1.19	0.356<br>(0.189)	0.060	-0.158<br>(0.189)	0.402
					rs2696692<br>T*/C	0.96	0.234	"-++"	upstream<br>*RP11-259G18.1*<br>0.52<br>6.18	0.294<br>(0.154)	0.056	-0.096<br>(0.154)	0.532

										Conditional Analysis			
**Sentinel variant**		**Unconditional analysis**		**N functional variants**	**Functional variant**					**Sentinel variant**		**Functional variant**	
**Locus<br>rsid<br>chr:position (b37)<br>noncoded/coded allele<br>(*minor allele)**	**MAF**	**beta<br>(se)**	**P (not gc-corrected)**	**All identified<br>(explain<br>the signal)**	**rsid<br>noncoded/coded allele<br>(*minor allele)**	**LD (r^2)**	**MAF**	**Imputation panel**	**Annotation:<br>Consequence<br>SYMBOL<br>GWAVA score<br>CADD scaled score**	**beta<br>(se)**	**P**	**beta<br>(se)**	**P**
					rs17663792<br>T*/C	0.96	0.230	"+++"	upstream<br>*KANSL1*<br>0.58<br>8.11	0.318<br>(0.153)	0.038	-0.120<br>(0.153)	0.430
					rs17763515<br>A*/G	0.95	0.229	"-++"	upstream<br>*SPPL2C*<br>0.55<br>2.17	0.334<br>(0.130)	0.010	-0.137<br>(0.128)	0.287
TSEN54<br>rs7218675<br>17:73513185<br>A/C*	0.291	0.164<br>(0.028)	2.4E-09	6 (0)	-	-	-	-	-		-		-
TET2<br>rs2047409<br>4:106137033<br>A/G*	0.345	0.155<br>(0.026)	2.7E-09	9 (1)	rs10007915<br>G*/C	0.87	0.371	"+++"	upstream<br>*TET2*<br>0.66<br>9.05	0.133<br>(0.071)	0.061	-0.023<br>(0.070)	0.747
<u>Heavy smokers vs Never smokers</u>													
NCAM1<br>rs4466874<br>11:112861434<br>C*/T	0.385	0.096<br>(0.013)	2.6E-13	7 (2)	rs7945073<br>A/G	1.00	0.385	"-++"	intronic<br>*NCAM1*<br>0.52<br>10.85	0.363<br>(0.208)	0.081	-0.267<br>(0.208)	0.199
					rs10789929<br>T/C	0.97	0.383	"-++"	upstream<br>*NCAM1*<br>0.55<br>2.64	0.145<br>(0.079)	0.067	-0.050<br>(0.080)	0.532
TEX41/PABPC1P2<br>rs10193706<br>2:146316319<br>C/A*	0.473	0.087<br>(0.013)	1.3E-11	22 (0)	-	-	-	-	-		-		-
C20orf112<br>rs143125561;rs57342388<br>20:31162590<br>CACGG*/C	0.233	0.094<br>(0.015)	7.9E-10	12 (0)	-	-	-	-	-		-		-
DNAH8<br>rs10807199<br>6:38901867<br>T*/C	0.473	0.074<br>(0.013)	6.5E-09	6 (0)	-	-	-	-	-		-		-

B) Previously reported regions (P < 10^{-3})

										Conditional Analysis			
**Sentinel variant**		**Unconditional analysis**		**N functional variants**	**Functional variant**					**Sentinel variant**		**Functional variant**	
**Locus<br>rsid<br>chr:position (b37)<br>noncoded/coded allele<br>(*minor allele)**	**MAF**	**beta<br>(se)**	**P (not gc-corrected)**	**All identified<br>(explain<br>the signal)**	**rsid<br>noncoded/coded allele<br>(*minor allele)**	**LD (r^2)**	**MAF**	**Imputation panel**	**Annotation:<br>Consequence<br>SYMBOL<br>GWAVA score<br>CADD scaled<br>score**	**beta<br>(se)**	**P**	**beta<br>(se)**	**P**
Low FEV_1 vs High FEV_1 in never smokers													
GSTCD<br>rs10516528<br>4:106739593<br>T*/G	0.063	-0.358<br>(0.051)	2.3E-12	9 (1)	rs77988914<br>C*/T	0.99	0.064	"-++"	intronic<br>*GSTCD*<br>0.55<br>2.39	-0.807<br>(0.389)	0.038	0.463<br>(0.385)	0.229
HHIP<br>rs13107665<br>4:145472644<br>G/A*	0.464	-0.159<br>(0.025)	1.3E-10	8 (0)	-	-	-	-	-		-		-
Low FEV_1 vs High FEV_1 in heavy smokers													
HTR4<br>rs12374521<br>5:147836880<br>T/C*	0.451	0.156<br>(0.026)	1.7E-09	1 (1)	rs6580550<br>C*/T	0.85	0.445	"+++"	downstream<br>*HTR4*<br>0.52<br>4.59	0.126<br>(0.066)	0.056	-0.033<br>(0.065)	0.611
Low FEV_1 vs High FEV_1 in never smokers													
ADAM19<br>rs10476073<br>5:156954363<br>C*/T	0.409	0.111<br>(0.025)	1.2E-05	4 (0)	-	-	-	-	-		-		-
ZKSCAN3<br>rs6904596<br>6:27491299<br>A*/G	0.133	0.219<br>(0.037)	2.3E-09	31 (5)	rs66785117<br>G*/T	0.90	0.120	"-++"	intergenic<br>NA<br>0.56<br>8.05	0.248<br>(0.122)	0.041	-0.025<br>(0.124)	0.838
					rs17750747<br>C*/T	0.89	0.120	"+++"	regulatory<br>NA<br>0.56<br>1.19	0.291<br>(0.117)	0.013	-0.072<br>(0.120)	0.550
					rs67101035<br>G*/C	0.89	0.120	"-++"	downstream<br>*HIST1H4K*<br>0.53<br>4.38	0.289<br>(0.114)	0.011	-0.070<br>(0.117)	0.549
					rs72847313<br>T*/C	0.89	0.119	"-++"	intergenic<br>NA<br>0.58<br>4.29	0.296<br>(0.116)	0.011	-0.077<br>(0.120)	0.519

										Conditional Analysis			
Sentinel variant		Unconditional analysis		N functional variants	Functional variant					Sentinel variant		Functional variant	
**Locus rsid chr:position (b37) noncoded/coded allele (*minor allele)**	**MAF**	**beta (se)**	**P (not gc-corrected)**	**All identified (explain the signal)**	**rsid noncoded/coded allele (*minor allele)**	**LD (r^2)**	**MAF**	**Imputation panel**	**Annotation: Consequence SYMBOL GWAVA score CADD scaled score**	**beta (se)**	**P**	**beta (se)**	**P**
					rs17751184<br>T*/C	0.89	0.120	"+++"	upstream<br>*HIST1H2AI*<br>0.64<br>4.80	0.293<br>(0.114)	0.010	-0.074<br>(0.116)	0.525
Low FEV_1 vs High FEV_1 in heavy smokers													
NCR3<br>rs36057735<br>6:31319923<br>G*/C	0.201	0.156<br>(0.032)	8.5E-07	39 (0)	-	-	-	-	-		-		-
Low FEV_1 vs High FEV_1 in never smokers													
ARMC2<br>rs2848598<br>6:109158405<br>T*/C	0.093	0.173<br>(0.043)	5.3E-05	2 (1)	rs2848600<br>A*/G	0.35	0.234	"-++"	regulatory<br>NA<br>0.58<br>3.99	0.138<br>(0.054)	0.010	0.046<br>(0.037)	0.207
Low FEV_1 vs Average FEV_1 in never smokers													
GPR126<br>rs4896582<br>6:142703877<br>A*/G	0.296	-0.109<br>(0.022)	1.1E-06	3 (1)	rs9389984<br>T*/C	0.85	0.266	"-++"	intronic<br>*GPR126*<br>0.59<br>2.54	-0.112<br>(0.057)	0.049	0.003<br>(0.059)	0.964
Low FEV_1 vs High FEV_1 in never smokers													
CDC123<br>rs78420228;rs67863175<br>10:12299623<br>C/CA	0.445	-0.169<br>(0.025)	2.5E-11	3 (1)	rs11593567<br>G/A*	0.95	0.458	"+++"	downstream<br>*RN7SL232P*<br>0.54<br>NA	-0.135<br>(0.109)	0.213	-0.035<br>(0.108)	0.743
C10orf11<br>rs11001819<br>10:78315224<br>A*/G	0.494	-0.118<br>(0.025)	2.1E-06	21 (5)	rs2579773<br>C*/A	0.75	0.462	"+++"	intergenic<br>NA<br>0.49<br>24.70	-0.086<br>(0.050)	0.084	0.037<br>(0.050)	0.459
					rs7894799<br>C*/T	0.75	0.462	"+++"	regulatory<br>NA<br>0.57<br>10.00	-0.087<br>(0.050)	0.080	0.036<br>(0.050)	0.477
					rs2579762<br>C*/A	0.86	0.477	"+++"	downstream<br>*C10orf11*<br>0.74<br>15.81	-0.123<br>(0.069)	0.073	-0.006<br>(0.069)	0.936

										Conditional Analysis			
**Sentinel variant**		**Unconditional analysis**		**N functional variants**	**Functional variant**					**Sentinel variant**		**Functional variant**	
**Locus<br>rsid<br>chr:position (b37)<br>noncoded/coded allele<br>(*minor allele)**	**MAF**	**beta<br>(se)**	**P (not gc-corrected)**	**All identified (explain the signal)**	**rsid<br>noncoded/coded allele<br>(*minor allele)**	**LD (r^2)**	**MAF**	**Imputation panel**	**Annotation:<br>Consequence<br>SYMBOL<br>GWAVA score<br>CADD scaled score**	**beta<br>(se)**	**P**	**beta<br>(se)**	**P**
					rs846575<br>A*/G	0.66	0.440	"+++"	intergenic<br>NA<br>0.55<br>16.51	-0.088<br>(0.042)	0.038	0.038<br>(0.043)	0.379
					rs846626<br>G*/T	0.65	0.439	"-++"	intergenic<br>NA<br>0.64<br>8.83	-0.092<br>(0.042)	0.028	0.032<br>(0.043)	0.448
<u>Low FEV_1 vs Average FEV_1 in never smokers</u>													
THSD4<br>rs4337253<br>15:71609306<br>C*/G	0.337	0.077<br>(0.021)	3.5E-04	2 (2)	rs11853359<br>A*/G	0.97	0.336	"+++"	intronic<br>*THSD4*<br>0.57<br>10.93	0.088<br>(0.118)	0.457	-0.011<br>(0.118)	0.923
					rs11856837<br>C*/T	0.41	0.174	"+++"	intronic<br>*THSD4*<br>0.56<br>12.57	0.059<br>(0.028)	0.033	0.034<br>(0.035)	0.326
<u>Low FEV_1 vs High FEV_1 in heavy smokers</u>													
CFDP1<br>rs8047983<br>16:75380305<br>C*/T	0.263	-0.150<br>(0.029)	2.0E-07	5 (1)	rs7186825<br>C*/T	0.68	0.198	"+++"	intronic<br>*RP11-77K12.1*<br>0.59<br>10.10	-0.120<br>(0.051)	0.017	-0.040<br>(0.056)	0.475
<u>Low FEV_1 vs Average FEV_1 in heavy smokers</u>													
KCNE2<br>rs56217903<br>21:35667824<br>T/A*	0.373	-0.072<br>(0.021)	7.0E-04	1 (0)	-	-	-	-	-		-		-
<u>Heavy smokers vs Never smokers</u>													
CHRNA3<br>rs71448806<br>15:78913353<br>C*/CGCGGGCGG	0.409	0.111<br>(0.013)	2.2E-17	11 (0)	-	-	-	-	-		-		-
DBH<br>rs111280114<br>9:136459454<br>G*/A	0.105	0.099<br>(0.021)	2.0E-06	8 (2)	rs739447<br>T*/C	0.80	0.119	"+++"	noncoding exonic<br>*LL09NC01-254D11.1*<br>0.53	0.060<br>(0.046)	0.196	0.042<br>(0.044)	0.339

										Conditional Analysis			
**Sentinel variant**		**Unconditional analysis**		**N functional variants**	**Functional variant**					**Sentinel variant**		**Functional variant**	
**Locus<br>rsid<br>chr:position (b37)<br>noncoded/coded allele<br>(*minor allele)**	**MAF**	**beta<br>(se)**	**P (not gc-corrected)**	**All identified (explain the signal)**	**rsid<br>noncoded/coded allele<br>(*minor allele)**	**LD (r^2)**	**MAF**	**Imputation panel**	**Annotation:<br>Consequence<br>SYMBOL<br>GWAVA score<br>CADD scaled score**	**beta<br>(se)**	**P**	**beta<br>(se)**	**P**
									7.75				
					rs148428140<br>T*/G	0.79	0.118	"-++"	downstream<br>*LL09NC01-254D11.1*<br>0.59<br>6.48	0.064<br>(0.045)	0.156	0.037<br>(0.043)	0.391
BDNF-AS<br>rs2049045<br>11:27694241<br>C*/G	0.184	-0.066<br>(0.017)	7.2E-05	9 (1 (missense))	rs6265<br>T*/C	0.98	0.186	"+++"	missense[#]<br>*BDNF*<br>-<br>13.00	-0.148<br>(0.132)	0.263	0.083<br>(0.132)	0.528
HSD17B12<br>rs11037504<br>11:43631737<br>A*/G	0.445	-0.047<br>(0.013)	3.0E-04	4 (2)	rs12275384<br>T*/C	0.85	0.421	"+++"	intronic<br>*HSD17B12*<br>0.61<br>4.86	-0.034<br>(0.032)	0.288	-0.014<br>(0.032)	0.655
					rs4755717<br>G*/C	0.87	0.413	"-++"	upstream<br>*MIR129-2*<br>0.58<br>10.70	-0.039<br>(0.034)	0.253	-0.009<br>(0.034)	0.799
PRDM11<br>rs11604310<br>11:45351420<br>T*/C	0.159	0.060<br>(0.018)	7.1E-04	10 (8)	rs7484258<br>T/G*	0.98	0.162	"-++"	regulatory<br>NA<br>0.53<br>NA	0.046<br>(0.112)	0.682	-0.013<br>(0.111)	0.906
					rs7952613<br>C*/G	0.98	0.162	"+++"	regulatory<br>NA<br>0.54<br>0.27	0.077<br>(0.111)	0.488	-0.018<br>(0.110)	0.869
					rs56408918<br>G/A*	0.32	0.295	"-++"	downstream<br>*PRDM11*<br>0.59<br>NA	0.041<br>(0.021)	0.054	-0.025<br>(0.017)	0.138
					rs61882568<br>T*/C	0.81	0.160	"-++"	upstream<br>*RP11-430H10.2*<br>0.56<br>9.41	0.077<br>(0.040)	0.053	-0.019<br>(0.039)	0.621
					rs7943277<br>G*/C	0.81	0.160	"+++"	upstream<br>*RP11-430H10.2*<br>0.55<br>6.24	0.078<br>(0.040)	0.050	-0.021<br>(0.039)	0.601

										**Conditional Analysis**			
**Sentinel variant**		**Unconditional analysis**		**N functional variants**	**Functional variant**					**Sentinel variant**		**Functional variant**	
**Locus**<br>**rsid**<br>**chr:position (b37)**<br>**noncoded/coded allele**<br>**(*minor allele)**	**MAF**	**beta**<br>**(se)**	**P (not gc-corrected)**	**All identified**<br>**(explain**<br>**the signal)**	**rsid**<br>**noncoded/coded allele**<br>**(*minor allele)**	**LD (r^2)**	**MAF**	**Imputation panel**	**Annotation:**<br>**Consequence**<br>**SYMBOL**<br>**GWAVA score**<br>**CADD scaled score**	**beta**<br>**(se)**	**P**	**beta**<br>**(se)**	**P**
					rs7943376<br>G*/C	0.81	0.160	"+++"	upstream<br>*RP11-430H10.2*<br>0.64<br>9.09	0.078<br>(0.040)	0.050	-0.021<br>(0.039)	0.601
					rs10769130<br>A/C*	0.33	0.296	"-++"	intronic<br>*CTD-2560E9.3*<br>0.52<br>NA	0.042<br>(0.021)	0.049	-0.024<br>(0.017)	0.163
					rs11607009<br>C*/T	0.53	0.224	"-++"	noncoding exonic<br>*RP11-430H10.1*<br>0.56<br>7.12	0.063<br>(0.026)	0.014	-0.005<br>(0.022)	0.836

Supplementary Table 22: Identification of differential gene expression of genes within novel loci associated with extremes of FEV_1 in the lungs of individuals with and without COPD. LogFC: Log2-fold change between the gene expression in bronchial brushings of COPD and non-COPD samples. Adjusted P: P value after adjustment for multiple testing.

**Locus**	**Gene**	**ST 1.0 array probe**	**logFC**	**Adjusted P**
RBM19/TBX5	*TBX5*	6910_at	3.46E-02	**4.47E-01**
	RBM19	9904_at	4.12E-03	**9.34E-01**
KANSL1	*LRRC37A*	387646_at	2.67E-02	**8.15E-01**
	KANSL1 (KIAA1267)	284058_at	5.97E-04	**9.88E-01**
	MAPT	4137_at	4.49E-02	**2.45E-01**
	CRHR1	1394_at	2.32E-02	**5.67E-01**
	LRRC37A4P	55073_at	1.20E-01	**5.16E-01**
	PLEKHM1	9842_at	-5.67E-02	**2.53E-01**
	WNT3	7473_at	6.28E-02	**1.14E-01**
	ARL17A	23647_at	-4.63E-02	**2.43E-01**
	BRWD1	54014_at	1.47E-02	**8.01E-01**
	TXNRD1	7296_at	9.82E-02	**3.74E-01**
	SH3D20 (ARHGAP27)	201176_at	-2.72E-02	**4.37E-01**
	EPB41L5	57669_at	8.36E-02	**7.63E-02**
	NUDT1	4521_at	-3.43E-02	**4.11E-01**
TSEN54	*TSEN54*	283989_at	5.08E-03	**9.09E-01**
	GRB2	2885_at	3.00E-03	**9.50E-01**
	KIAA0195	9772_at	1.94E-02	**7.57E-01**
TET2	*TET2*	54790_at	-2.50E-02	**6.28E-01**
	PPA2	27068_at	-2.48E-02	**6.76E-01**

Supplementary Table 23: Gene-based analysis results using SKAT-O. [1]Gene-based tests were performed using SKAT-O for 2 subgroups of variants: 1) loss-of-function (LoF) and missense variants (N variants=92,858; N genes (>2 variants)=9,980), and 2) deleterious loss-of-function and missense variants (N variants=32,161; N genes (>2 variants)=3,393). [2]Variants predicted by SIFT to be 'deleterious'[52]; or by PolyPhen-2 to be 'probably damaging' or 'possibly damaging'[53]; or variants with CADD_PHRED score ≥ 20[54]. Column names: SKAT-O P: P value from SKAT-O test, SKAT P: P value from SKAT test; DropOne SKAT-O P: P value from SKAT-O test after dropping the variant that has the largest effect on the SKAT-O signal.

Smoking status	Comparison	Gene	Variant class [1]	SKAT-O P	SKAT P	# variants	Variant responsible for signal	DropOne SKAT-O P	Signal driven by single variant?
Never smokers	Low vs high FEV_1	***KIT***	**LoF & missense**	**2.3E-05**	**1.3E-02**	**61**	**rs72550820**	**0.0005**	**NO?**
		NT5DC2	LoF & missense	4.0E-05	3.6E-05	3	rs35920544	1	YES
		ABHD12	LoF & missense	5.0E-05	4.4E-05	3	rs41306784	0.344	YES
		PROX2	LoF & missense	7.1E-05	6.9E-05	6	rs117853159	0.806	YES
	Low vs average FEV_1	*TFB2M*	LoF & missense	8.3E-05	7.1E-05	5	rs143880306	0.053	YES
		CASP5	LoF & missense	8.4E-05	8.6E-05	5	rs141361242	0.584	YES
	Average vs high FEV_1	*HFE2*	LoF & missense	6.9E-05	1.6E-04	6	rs56025621	0.232	YES
Heavy smokers	Low vs high FEV_1	***VSIG10***	**deleterious [2] LoF & missense**	**1.3E-05**	**1.2E-03**	**6**	**rs76814182**	**0.002**	**NO?**
		ATP5SL	LoF & missense	3.1E-05	1.8E-05	4	rs2231943	0.843	YES
		FAM83D	LoF & missense	5.7E-05	1.5E-04	3	rs41276984	0.105	YES
		SEPP1	**LoF & missense**	**8.5E-05**	**5.2E-05**	**6**	**rs28919926**	**0.006**	**NO?**
	Heavy smokers vs never smokers	*NFATC2*	LoF & missense	2.9E-07	1.2E-06	6	rs140836558	0.160	YES
		GPR151	LoF & missense	2.3E-05	2.2E-05	7	rs114285050	0.413	YES
		EPB41L4A	LoF & missense	5.9E-05	3.7E-04	15	rs17266567	0.013	YES
		IFT57	LoF & missense	6.2E-05	5.8E-05	3	rs35713185	0.327	YES
		CARF	LoF & missense	6.9E-05	6.9E-05	5	rs115268453	0.221	YES
		GPR156	deleterious LoF & missense	9.4E-05	6.2E-05	3	rs147315768	0.045	YES

Supplementary Figures

Supplementary Figure 1: **a) Imputation quality (spline smoothed) against minor allele frequency (MAF) (log scale), and b) Percentages of usable variants on chromosome 2 passing imputation quality control (INFO > 0.5) and minor allele count (MAC) ≥ 3, in different minor allele frequency (MAF) ranges.**

Imputation against 1000G panel alone (grey) and 1000G+UK10K (the rest) reference panels (total number of imputed variants on chromosome 2 is 3,515,740 variants with MAC ≥ 3 for UK10K+1000G panel and 3,292,965 for 1000G panel) is shown. Colour reflects the component of the array content used for imputation (cyan: basic GWAS grid (18367 variants), green: as cyan, plus “booster 1” content (7,127 variants) to optimise imputation of common variation in European ancestry, blue: as green, plus “booster 2” content (18,838 variants) to optimise imputation of low frequency (MAF 1-5%) variation in European ancestry, black: all array content (additional 3,887 variants). Chromosome 2 was used as it is the largest representative autosomal chromosome.

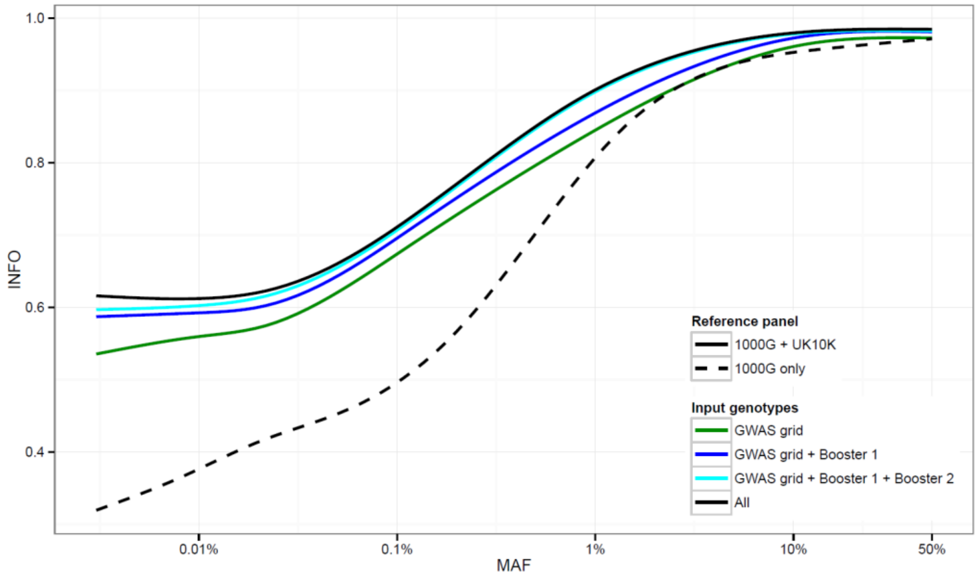

a)

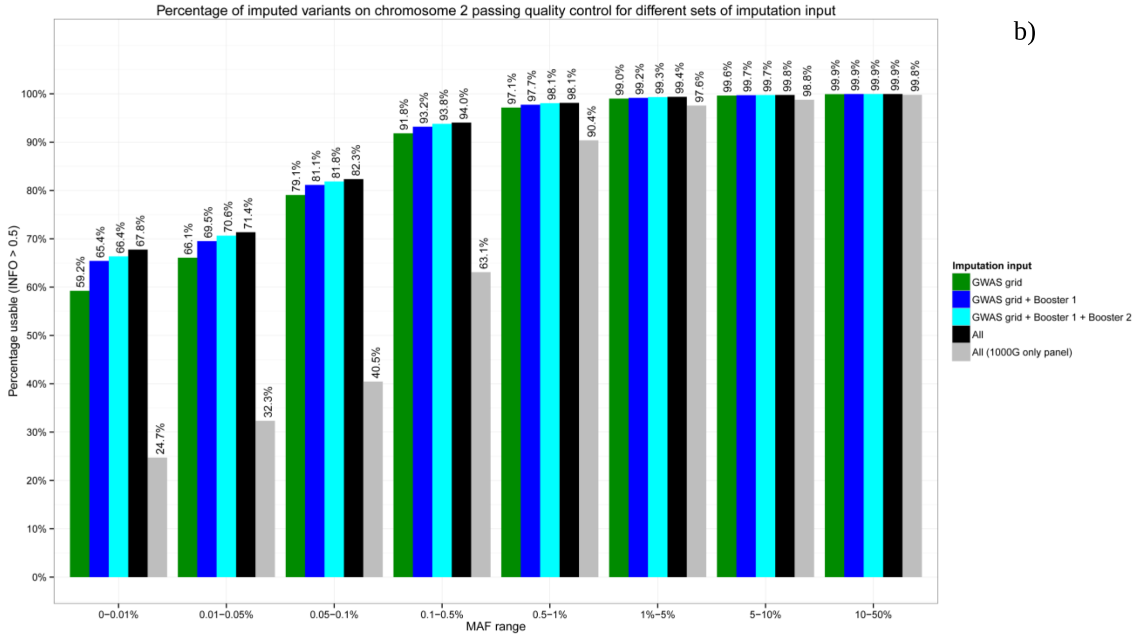

b)

Supplementary Figure 2: Region plots for novel signals of association with extremes of FEV_1.

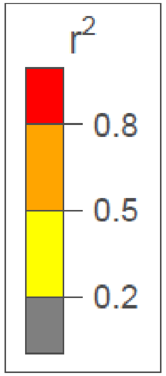

Colours used to show LD with sentinel variant (blue diamond) in region plots.

All region plots were produced with Locuszoom v.1.2 (http://locuszoom.sph.umich.edu/locuszoom/).

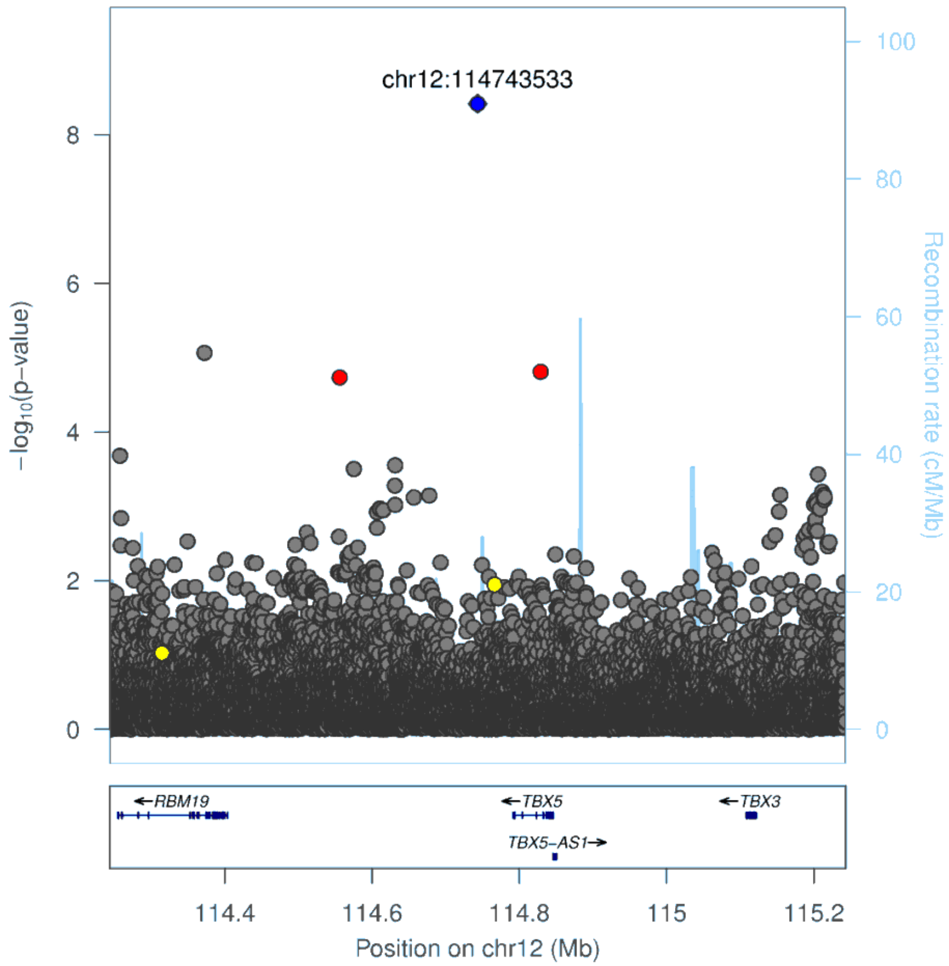

a) chr12:114743533: low FEV_1 vs high FEV_1 in heavy smokers.

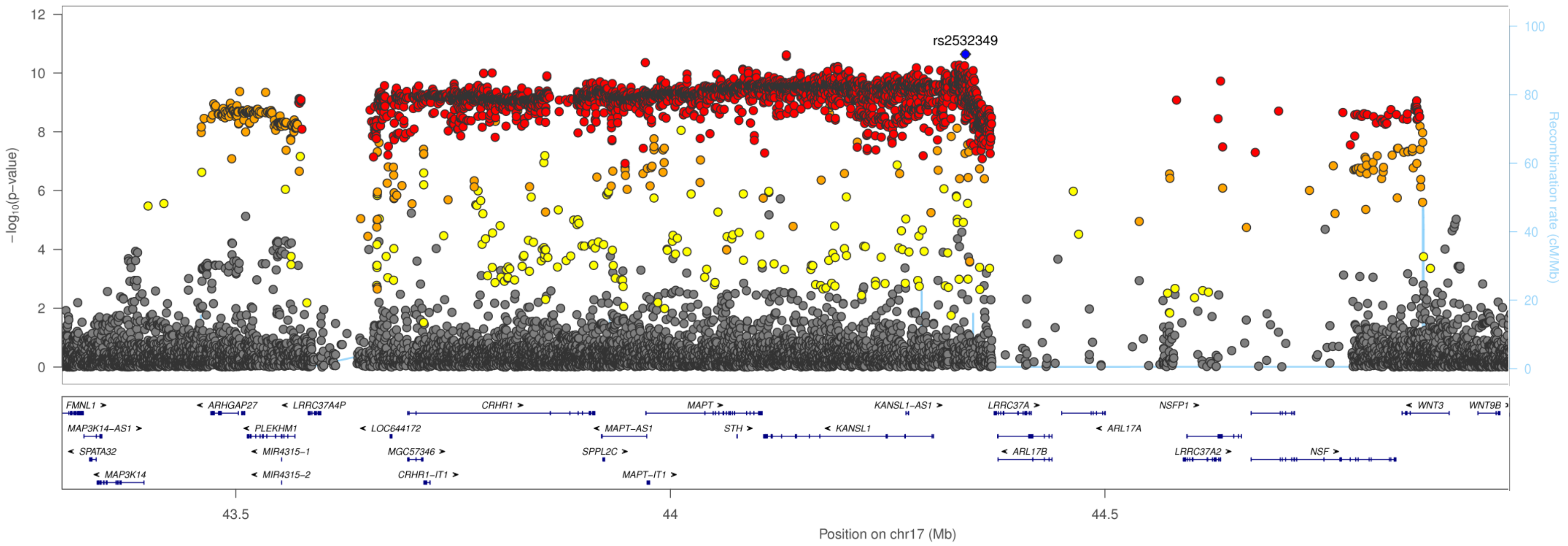

b) KANSL1 (17q21.31): low FEV_1 vs high FEV_1 in never smokers

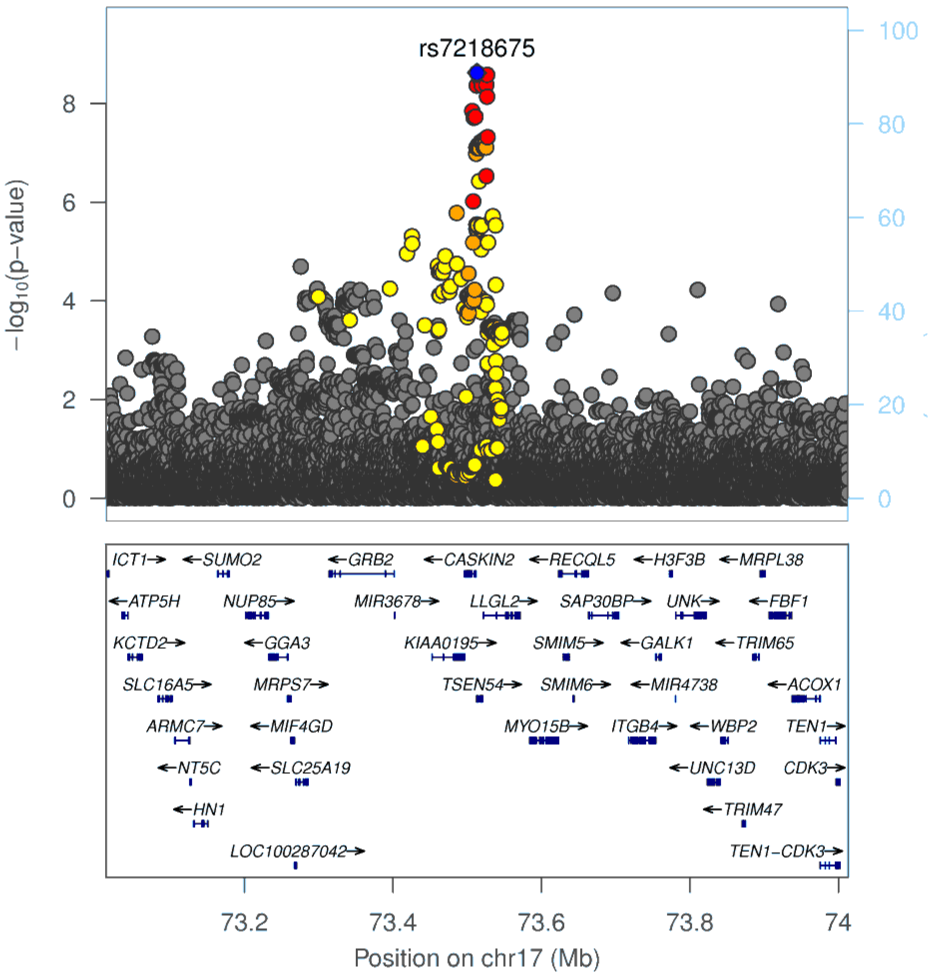

c) *TSEN54* low FEV_1 vs high FEV_1 in never smokers.

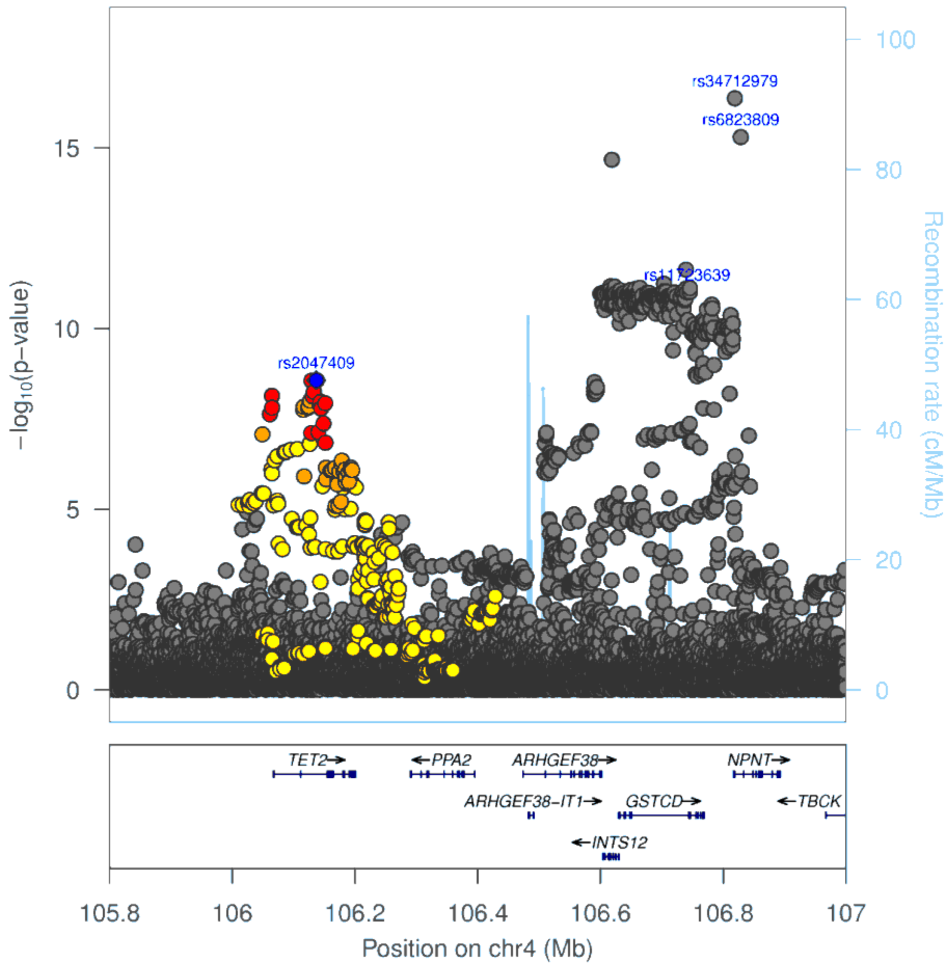

d) *TET2*: low FEV_1 vs high FEV_1 in never smokers. Broader region shown with top associations in previously reported *GSTCD* region.

Supplementary Figure 3: Region plots for independent signals at novel loci identified through joint conditional analysis with GCTA.

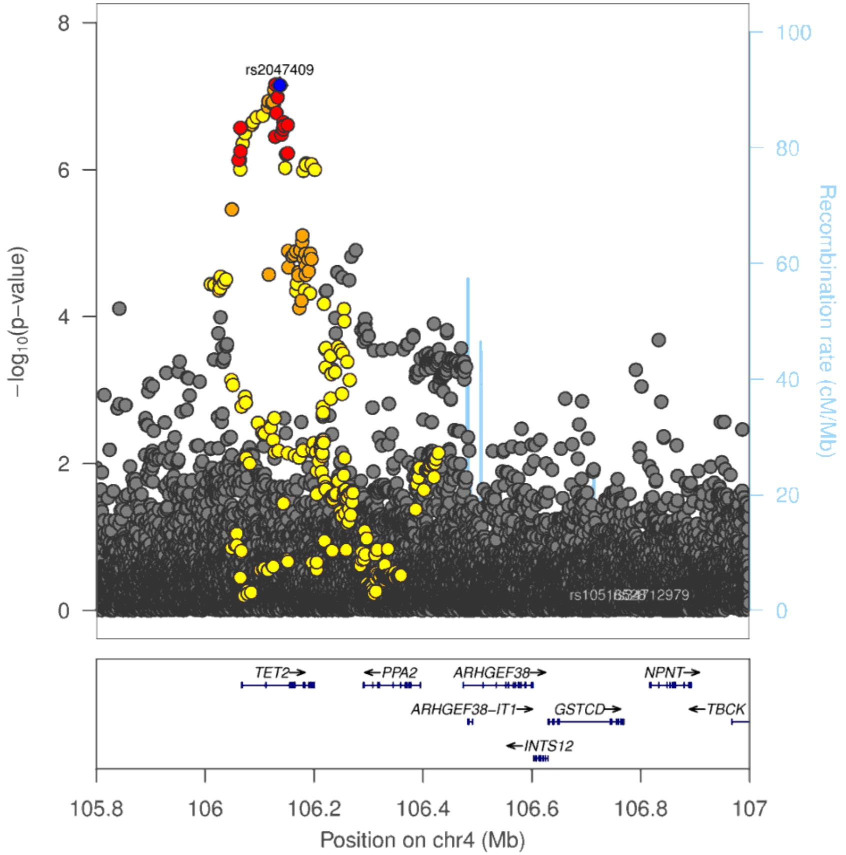

a) *TET2* novel independent signal rs2047409 conditioned on rs10516528 (*GSTCD*[2, 3]) & rs34712979 (*NPNT*, novel signal reported in this study).

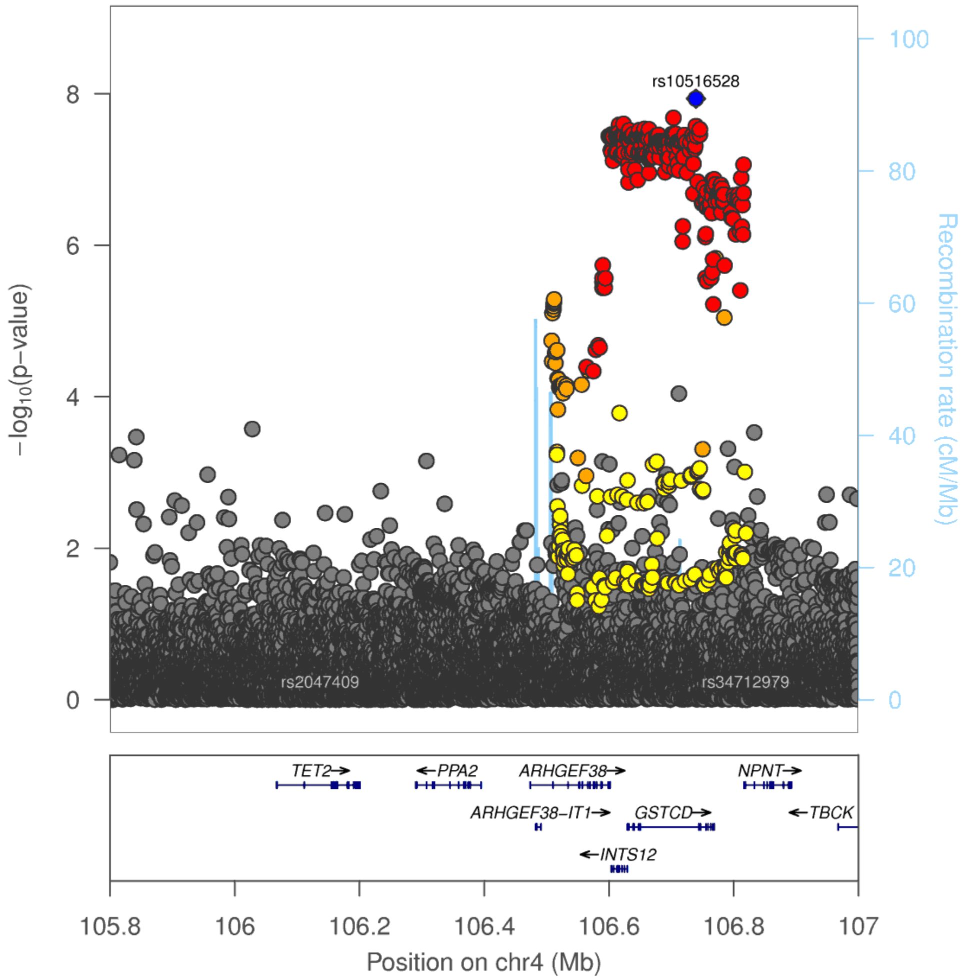

b) *GSTCD* independent signal rs10516528[2, 3] conditioned on rs2047409 (*TET2*, novel locus reported in this study) & rs34712979 (*NPNT*, novel signal reported in this study).

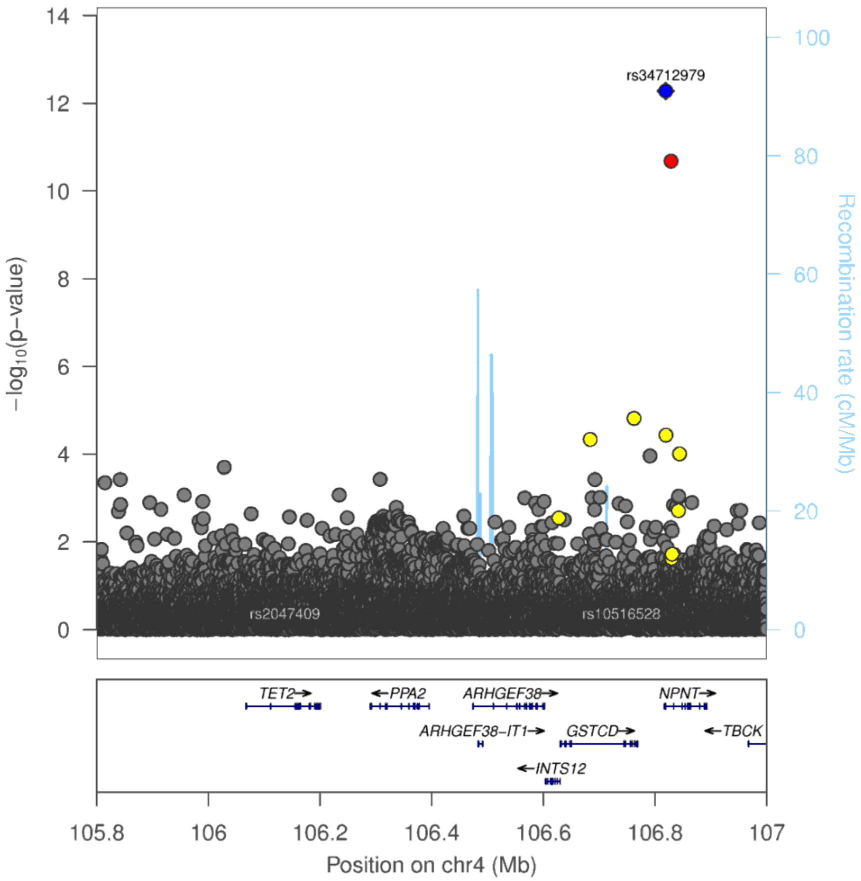

c) *NPNT* novel independent signal rs34712979 conditioned on rs2047409 (*TET2*, novel locus reported in this study) & rs10516528 (*GSTCD*[2, 3]).

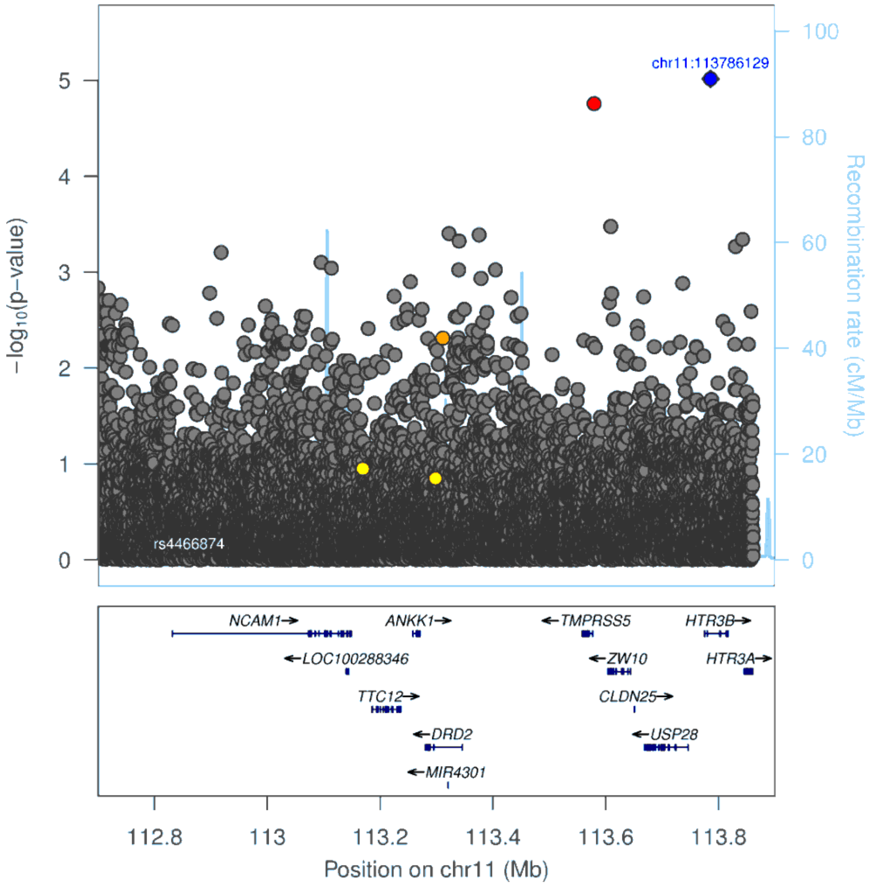

d) *NCAM1* independent signal chr11:113786129 conditioned on novel genome-wide significant signal rs4466874.

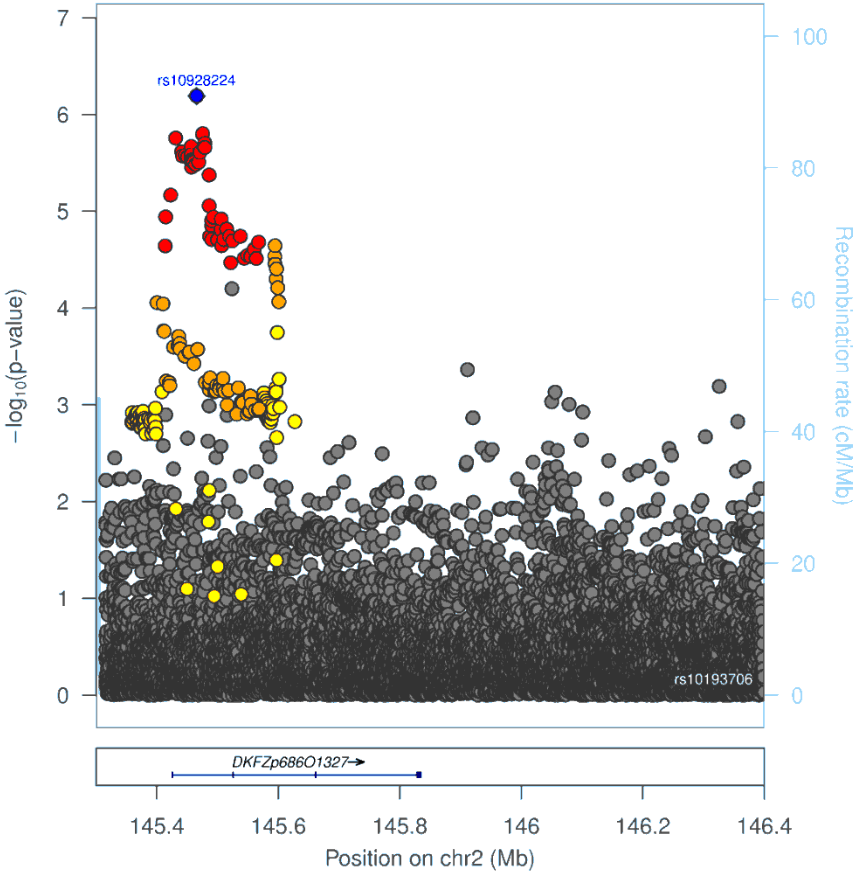

e) *TEX41/PABPC1P2* independent signal rs10928224
conditioned on novel genome-wide significant signal rs10193706.

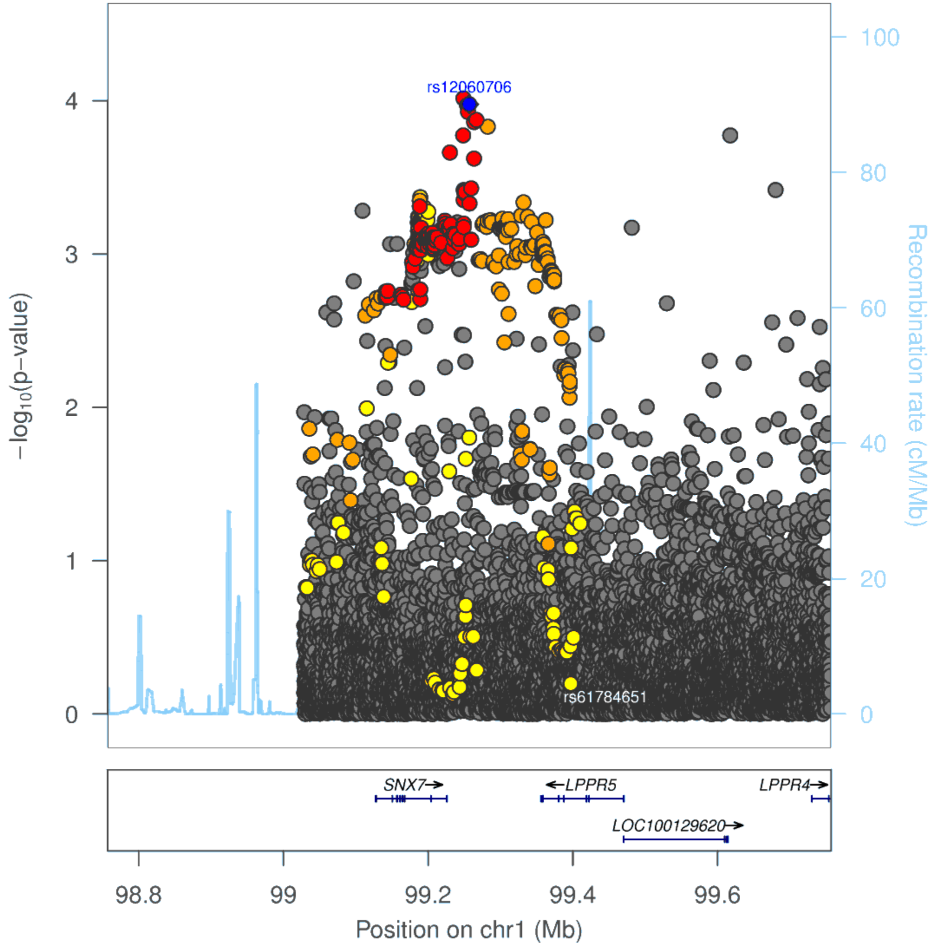

f) *LPPR5* independent signal rs12060706
conditioned on novel genome-wide significant signal rs61784651.

Supplementary Figure 4: **Region plots for novel signals of association at previously reported loci (*NPNT* and *HLA-DQB1*).**

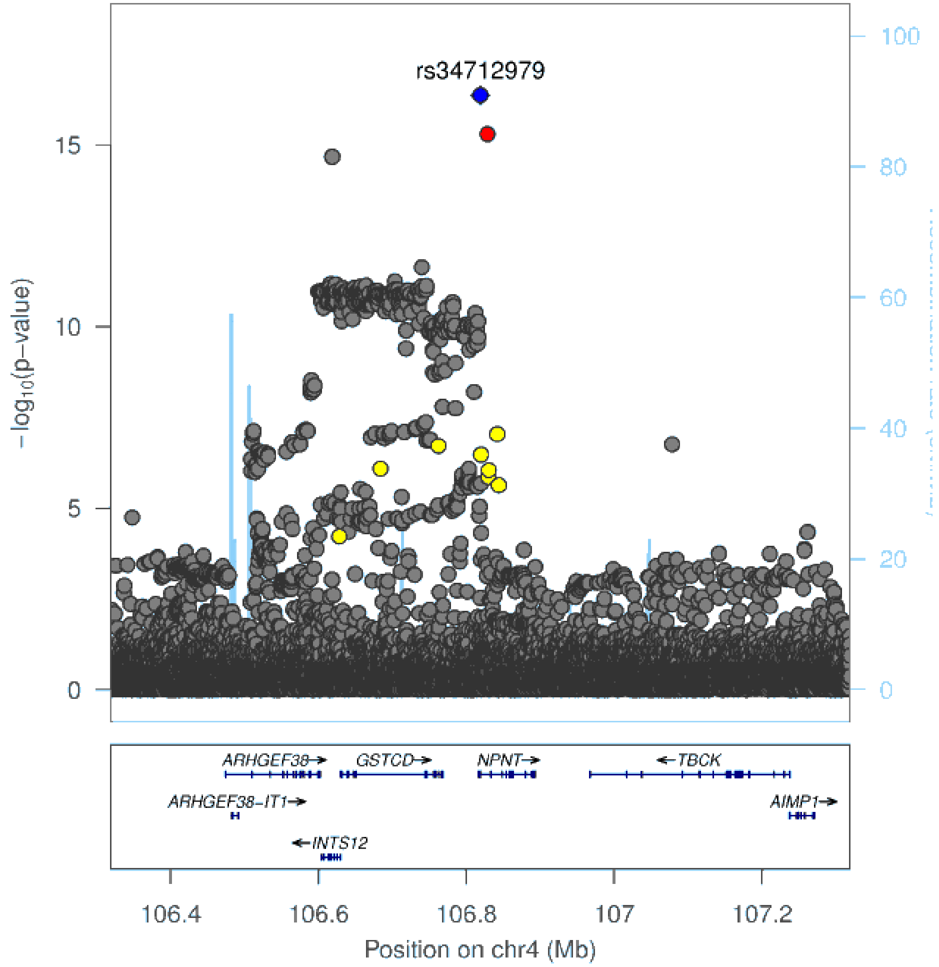

a) *NPNT* (low FEV_1 vs high FEV_1 in never smokers).

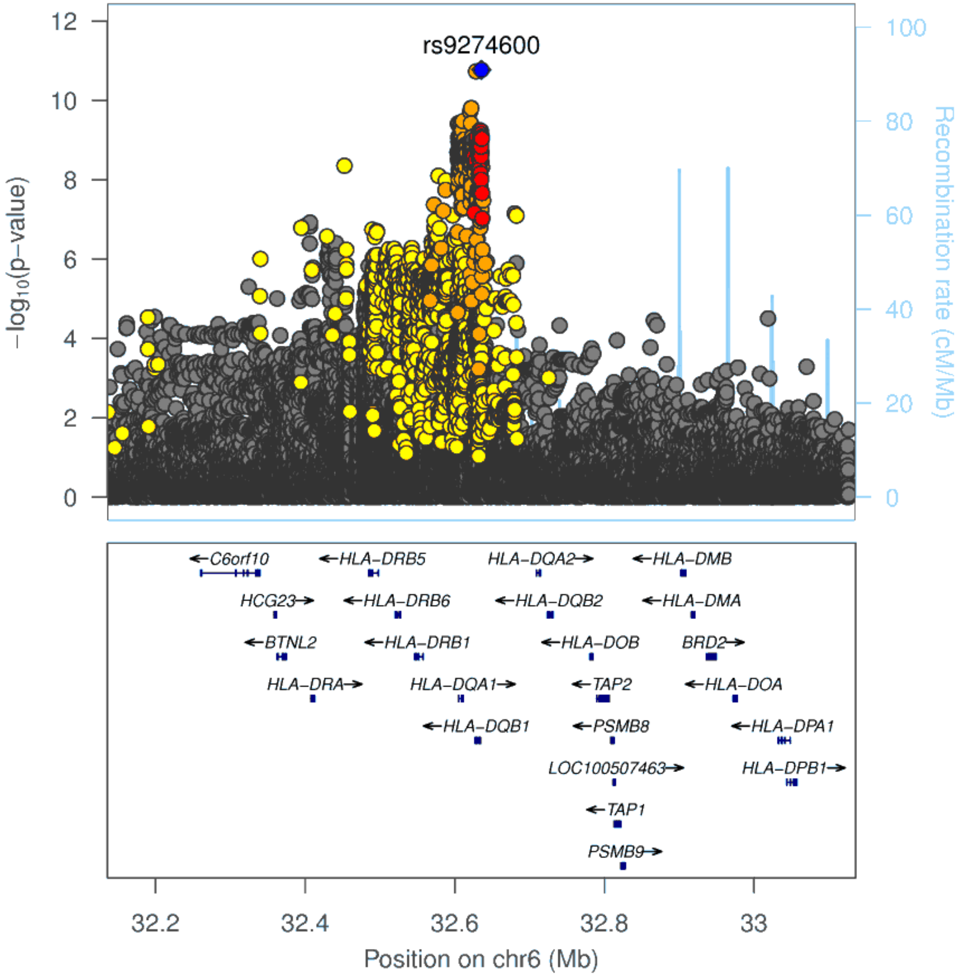

b) *HLA-DQB1*: low FEV_1 vs high FEV_1 in never smokers. Gabriel study asthma SNP is not in this study, but we have a proxy rs17843604 (r^2 0.917 with Gabriel SNP in HapMap 3; r^2 0.65 with rs9274600 in this study); rs17843604 association P = 4.86×10^{-9}.

Supplementary Figure 5: Effect of exclusion of individuals with asthma at novel loci associated with extremes of FEV_1. Odds ratios for the five novel genome-wide significant signals of association for low FEV_1 vs high FEV_1 in never smokers, before and after exclusion of individuals with doctor-diagnosed/self-reported asthma. A total of 2,828 individuals with low FEV_1 (never smokers) and 286 individuals with high FEV_1 (never smokers) with doctor-diagnosed/self-reported asthma were excluded.

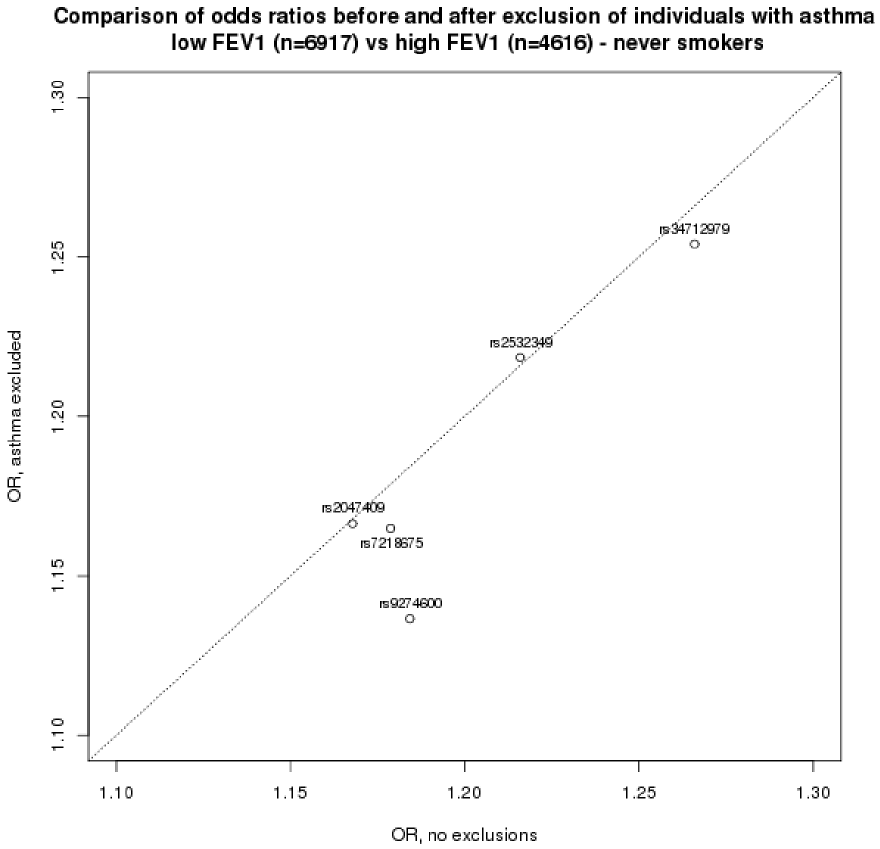

Supplementary Figure 6: Transcriptomic profiling of candidate lung function genes in primary human bronchial epithelial cells. Figures show novel and previously described (Ensembl) mRNA isoforms as well as their percent abundance. *A:* Individual predicted gene's isoforms with indicated splice variation identified using messenger RNA sequencing. Different splice events are described in the box beneath the main graph and novel transcripts are indicated by star. X-axis contains two outmost genomic coordinates. *B:* Percent abundance of individual transcripts in primary human bronchial epithelial cells (passage 3) grown under basal conditions.

Low vs high FEV_1 - never smokers
Locus *KANSL1*

A

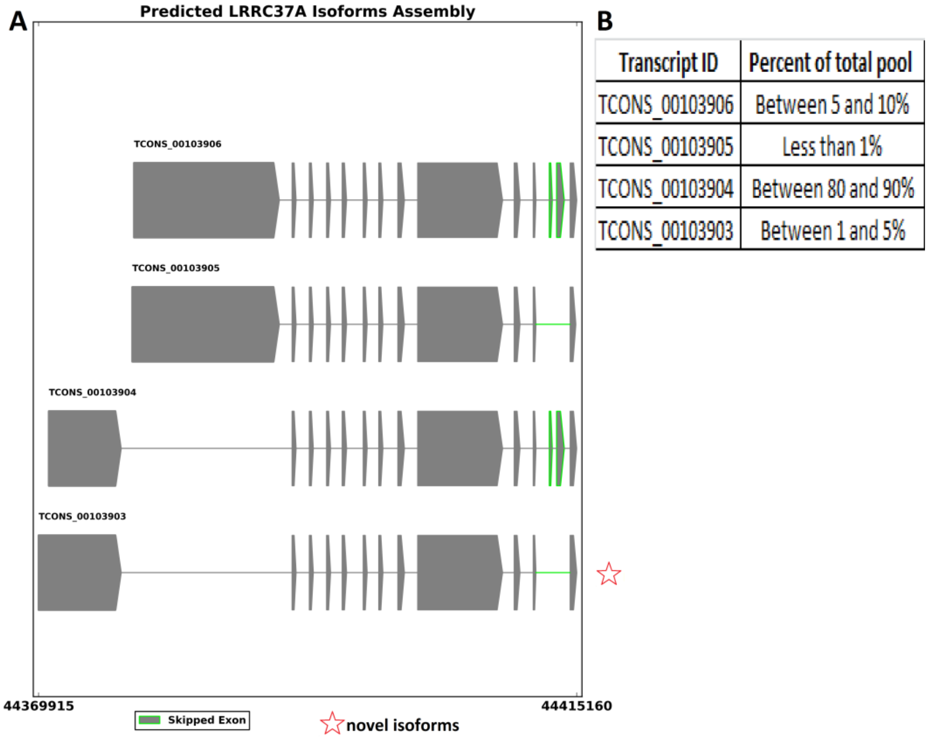

B

Transcript ID	Percent of total pool
TCONS_00103906	Between 5 and 10%
TCONS_00103905	Less than 1%
TCONS_00103904	Between 80 and 90%
TCONS_00103903	Between 1 and 5%

A

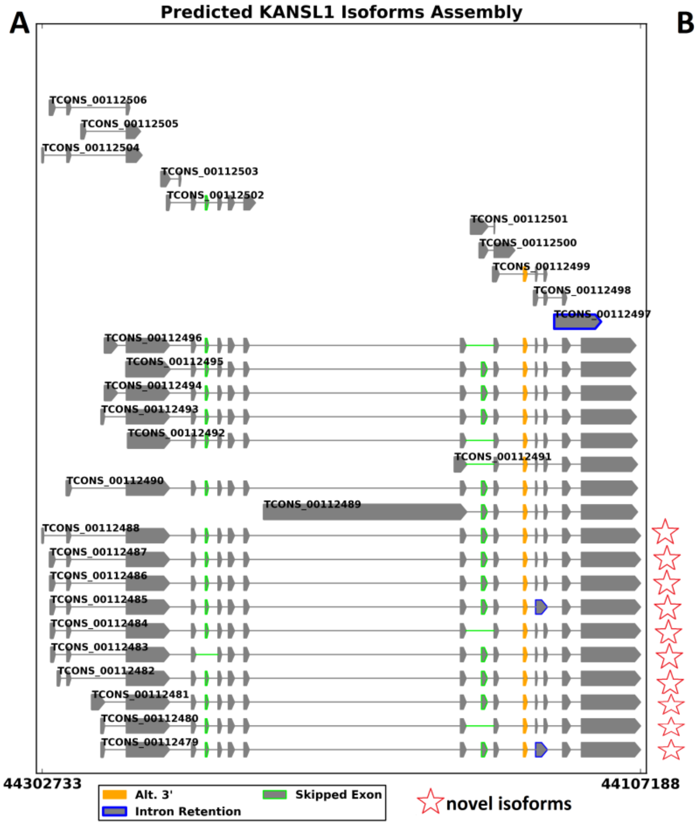

B

Transcript ID	Percent of total pool
TCONS_00112506	Between 1 and 5%
TCONS_00112505	Less than 1%
TCONS_00112504	Between 20 and 30%
TCONS_00112503	Between 10 and 20%
TCONS_00112502	Less than 1%
TCONS_00112501	Less than 1%
TCONS_00112500	Less than 1%
TCONS_00112499	Less than 1%
TCONS_00112498	Less than 1%
TCONS_00112497	Less than 1%
TCONS_00112496	Between 1 and 5%
TCONS_00112495	Between 20 and 30%
TCONS_00112494	Less than 1%
TCONS_00112493	Between 10 and 20%
TCONS_00112492	Between 1 and 5%
TCONS_00112491	Less than 1%
TCONS_00112490	Between 10 and 20%
TCONS_00112489	Less than 1%
TCONS_00112488	Between 1 and 5%
TCONS_00112487	Less than 1%
TCONS_00112486	Less than 1%
TCONS_00112485	Less than 1%
TCONS_00112484	Less than 1%
TCONS_00112483	Between 1 and 5%
TCONS_00112482	Less than 1%
TCONS_00112481	Between 1 and 5%
TCONS_00112480	Less than 1%
TCONS_00112479	Less than 1%

A

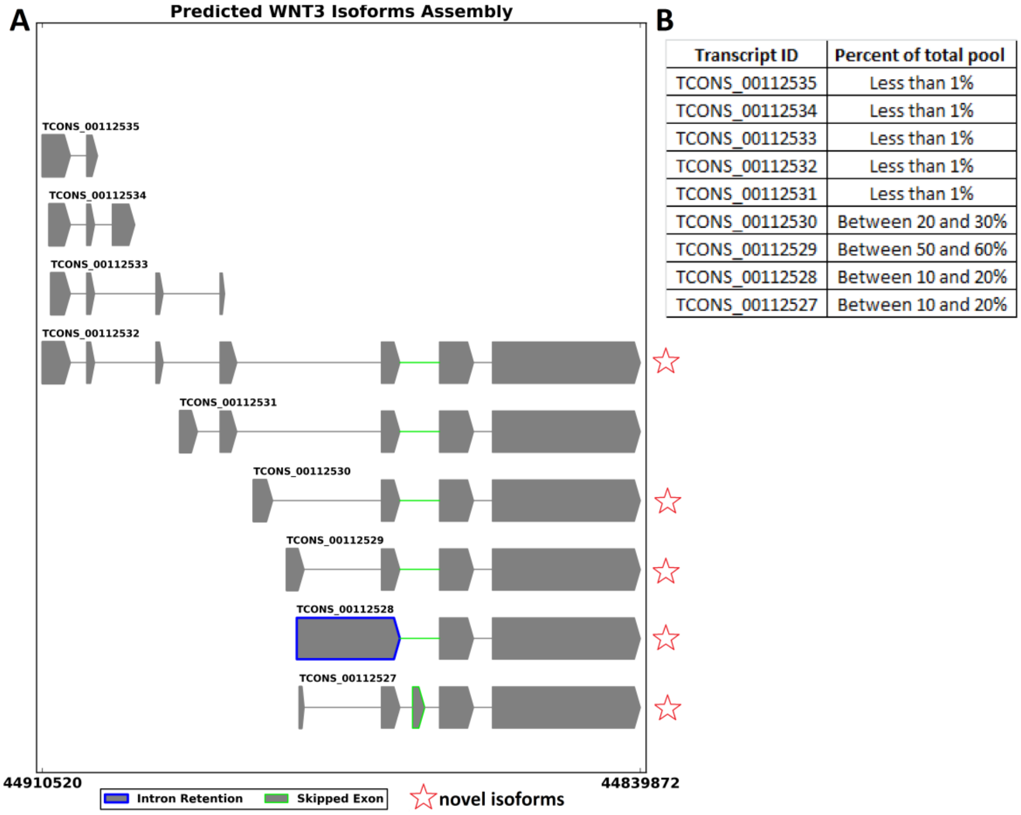

B

Transcript ID	Percent of total pool
TCONS_00112535	Less than 1%
TCONS_00112534	Less than 1%
TCONS_00112533	Less than 1%
TCONS_00112532	Less than 1%
TCONS_00112531	Less than 1%
TCONS_00112530	Between 20 and 30%
TCONS_00112529	Between 50 and 60%
TCONS_00112528	Between 10 and 20%
TCONS_00112527	Between 10 and 20%

A

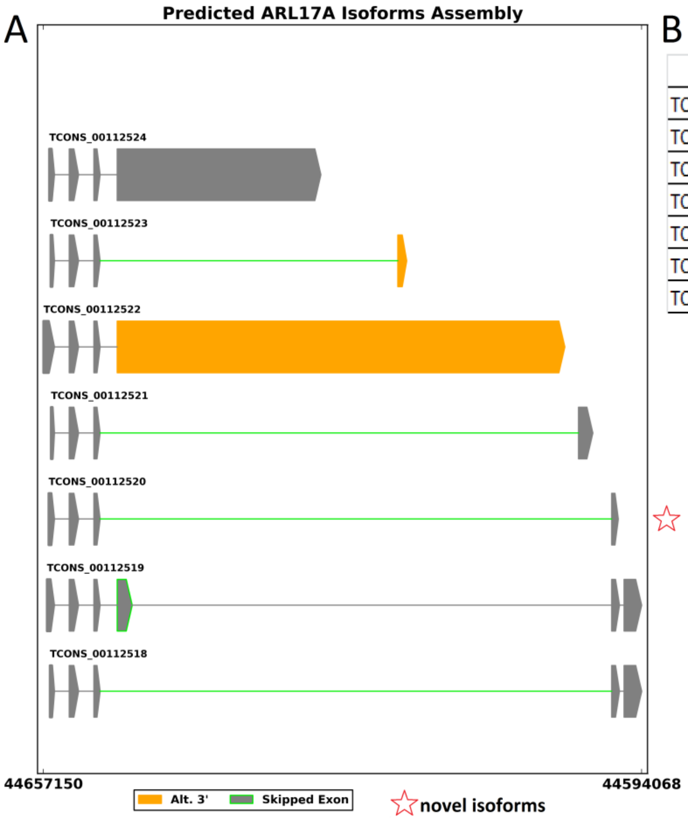

B

Transcript ID	Percent of total pool
TCONS_00112524	Between 70 and 80%
TCONS_00112523	Less than 1%
TCONS_00112522	Between 10 and 20%
TCONS_00112521	Less than 1%
TCONS_00112520	Between 1 and 5%
TCONS_00112519	Between 1 and 5%
TCONS_00112518	Between 1 and 5%

A

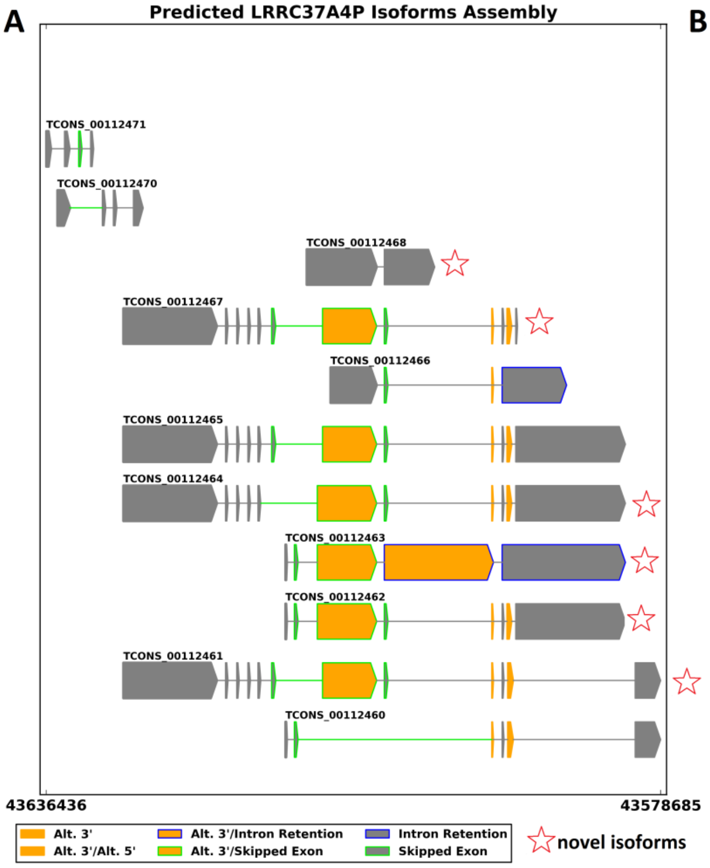

B

Transcript ID	Percent of total pool
TCONS_00112471	Less than 1%
TCONS_00112470	Between 5 and 10%
TCONS_00112468	Less than 1%
TCONS_00112467	Between 5 and 10%
TCONS_00112466	Between 40 and 50%
TCONS_00112465	Less than 1%
TCONS_00112464	Less than 1%
TCONS_00112463	Between 1 and 5%
TCONS_00112462	Between 40 and 50%
TCONS_00112461	Less than 1%
TCONS_00112460	Less than 1%

A

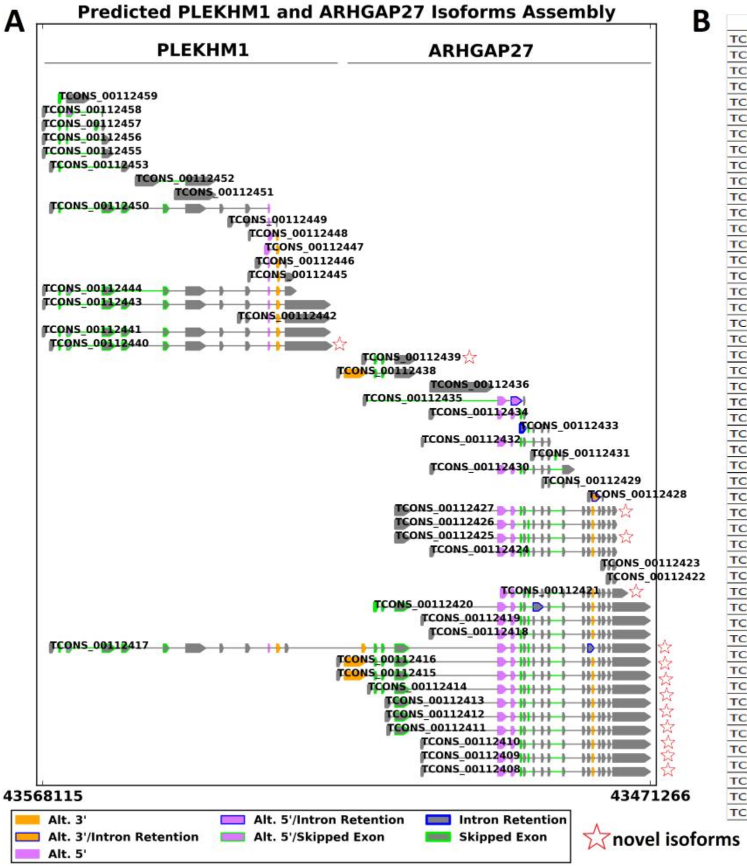

B

Transcript ID	Percent of total pool
TCONS_00112459	Between 1 and 5%
TCONS_00112458	Between 5 and 10%
TCONS_00112457	Between 1 and 5%
TCONS_00112456	Between 1 and 5%
TCONS_00112455	Less than 1%
TCONS_00112453	Less than 1%
TCONS_00112452	Less than 1%
TCONS_00112451	Between 1 and 5%
TCONS_00112450	Less than 1%
TCONS_00112449	Less than 1%
TCONS_00112448	Less than 1%
TCONS_00112447	Less than 1%
TCONS_00112446	Less than 1%
TCONS_00112445	Between 1 and 5%
TCONS_00112444	Between 1 and 5%
TCONS_00112443	Less than 1%
TCONS_00112442	Between 5 and 10%
TCONS_00112441	Between 20 and 30%
TCONS_00112440	Between 1 and 5%
TCONS_00112439	Between 5 and 10%
TCONS_00112438	Less than 1%
TCONS_00112436	Less than 1%
TCONS_00112435	Less than 1%
TCONS_00112434	Less than 1%
TCONS_00112433	Less than 1%
TCONS_00112432	Less than 1%
TCONS_00112431	Less than 1%
TCONS_00112430	Between 1 and 5%
TCONS_00112429	Less than 1%
TCONS_00112428	Between 1 and 5%
TCONS_00112427	Less than 1%
TCONS_00112426	Less than 1%
TCONS_00112425	Less than 1%
TCONS_00112424	Less than 1%
TCONS_00112423	Less than 1%
TCONS_00112422	Between 1 and 5%
TCONS_00112421	Less than 1%
TCONS_00112420	Less than 1%
TCONS_00112419	Between 5 and 10%
TCONS_00112418	Less than 1%
TCONS_00112417	Between 1 and 5%
TCONS_00112416	Less than 1%
TCONS_00112415	Between 1 and 5%
TCONS_00112414	Between 1 and 5%
TCONS_00112413	Less than 1%
TCONS_00112412	Less than 1%
TCONS_00112411	Less than 1%
TCONS_00112410	Between 5 and 10%
TCONS_00112409	Between 1 and 5%
TCONS_00112408	Less than 1%

A

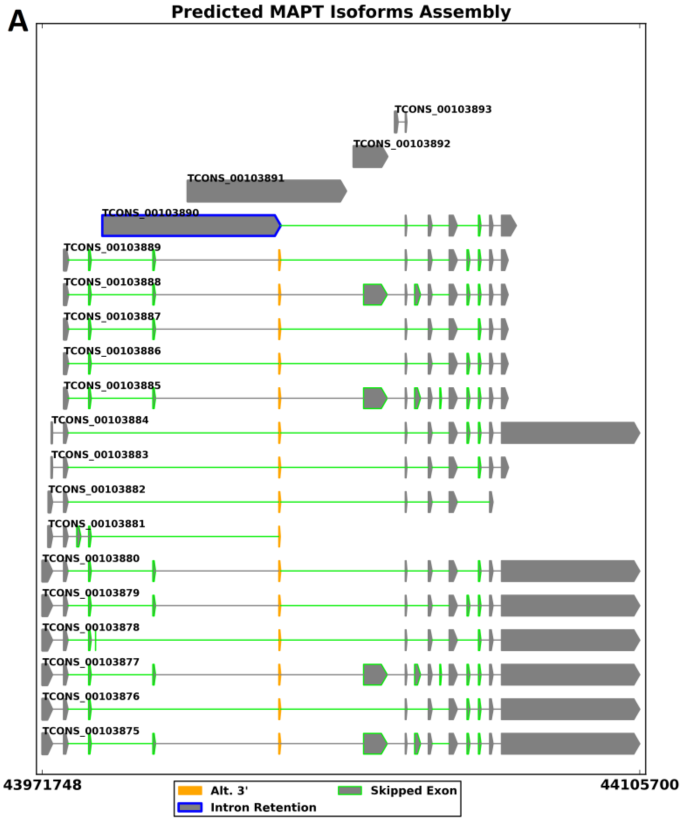

B

Transcript ID	Percent of total pool
TCONS_00103875	Between 5 and 10%
TCONS_00103876	Less than 1%
TCONS_00103877	Less than 1%
TCONS_00103878	Less than 1%
TCONS_00103879	Less than 1%
TCONS_00103880	Less than 1%
TCONS_00103881	Less than 1%
TCONS_00103882	Less than 1%
TCONS_00103883	Between 10 and 20%
TCONS_00103884	Between 30 and 40%
TCONS_00103885	Less than 1%
TCONS_00103886	Between 10 and 20%
TCONS_00103887	Between 10 and 20%
TCONS_00103888	Between 10 and 20%
TCONS_00103889	Less than 1%
TCONS_00103890	Less than 1%
TCONS_00103891	Less than 1%
TCONS_00103892	Less than 1%
TCONS_00103893	Less than 1%

Locus *TSEN54*

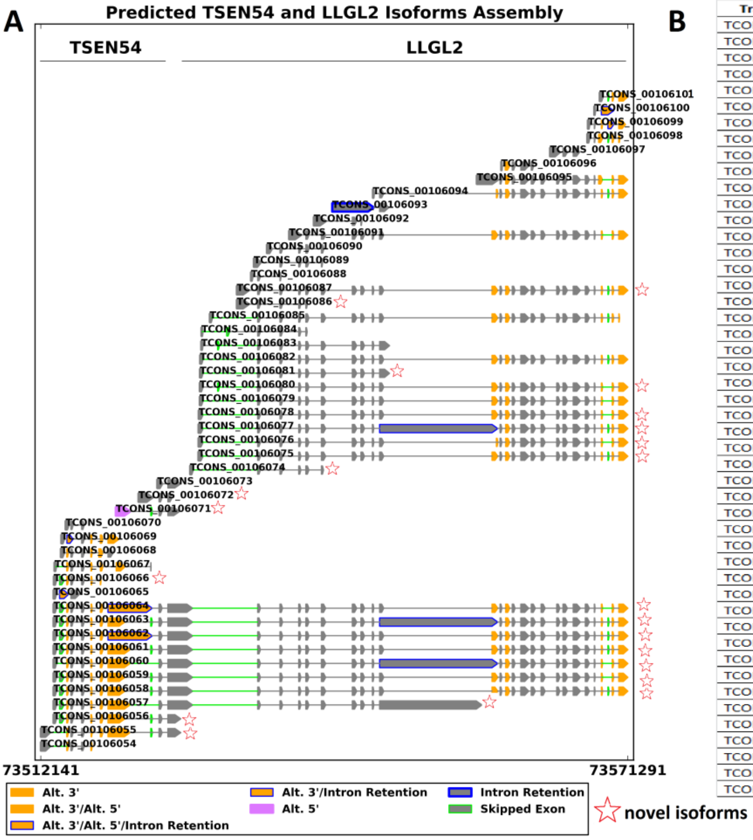

B

Transcript ID	Percent of total pool
TCONS_00106101	Between 1 and 5%
TCONS_00106100	Less than 1%
TCONS_00106099	Less than 1%
TCONS_00106098	Less than 1%
TCONS_00106097	Between 1 and 5%
TCONS_00106096	Less than 1%
TCONS_00106095	Between 1 and 5%
TCONS_00106094	Less than 1%
TCONS_00106093	Less than 1%
TCONS_00106092	Less than 1%
TCONS_00106091	Less than 1%
TCONS_00106090	Less than 1%
TCONS_00106089	Less than 1%
TCONS_00106088	Less than 1%
TCONS_00106087	Less than 1%
TCONS_00106086	Less than 1%
TCONS_00106085	Between 1 and 5%
TCONS_00106084	Less than 1%
TCONS_00106083	Less than 1%
TCONS_00106082	Between 10 and 20%
TCONS_00106081	Between 5 and 10%
TCONS_00106080	Less than 1%
TCONS_00106079	Between 5 and 10%
TCONS_00106078	Between 1 and 5%
TCONS_00106077	Between 1 and 5%
TCONS_00106076	Less than 1%
TCONS_00106075	Less than 1%
TCONS_00106074	Between 1 and 5%
TCONS_00106073	Between 5 and 10%
TCONS_00106072	Less than 1%
TCONS_00106071	Between 1 and 5%
TCONS_00106070	Less than 1%
TCONS_00106069	Less than 1%
TCONS_00106068	Less than 1%
TCONS_00106067	Less than 1%
TCONS_00106066	Less than 1%
TCONS_00106065	Less than 1%
TCONS_00106064	Less than 1%
TCONS_00106063	Less than 1%
TCONS_00106062	Between 5 and 10%
TCONS_00106061	Between 1 and 5%
TCONS_00106060	Between 1 and 5%
TCONS_00106059	Less than 1%
TCONS_00106058	Less than 1%
TCONS_00106057	Less than 1%
TCONS_00106056	Between 20 and 30%
TCONS_00106055	Less than 1%
TCONS_00106054	Less than 1%

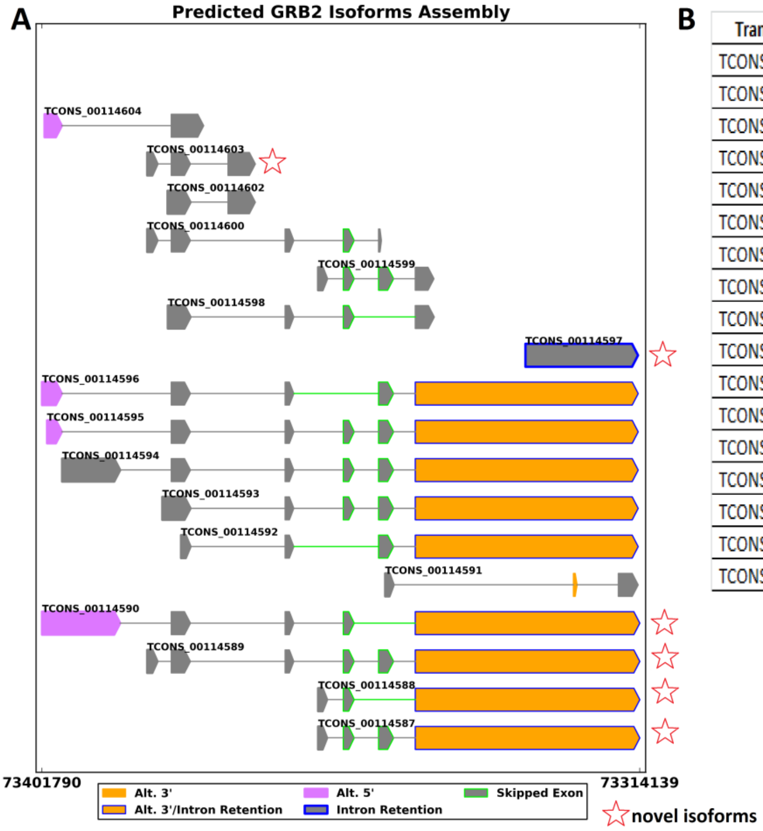

B

Transcript ID	Percent of total pool
TCONS_00114604	Between 5 and 10%
TCONS_00114603	Less than 1%
TCONS_00114602	Less than 1%
TCONS_00114600	Less than 1%
TCONS_00114599	Less than 1%
TCONS_00114598	Less than 1%
TCONS_00114597	Between 5 and 10%
TCONS_00114596	Between 1 and 5%
TCONS_00114595	Between 50 and 60%
TCONS_00114594	Less than 1%
TCONS_00114593	Between 20 and 30%
TCONS_00114592	Less than 1%
TCONS_00114591	Less than 1%
TCONS_00114590	Less than 1%
TCONS_00114589	Between 1 and 5%
TCONS_00114588	Less than 1%
TCONS_00114587	Less than 1%

Locus *TET2*

A

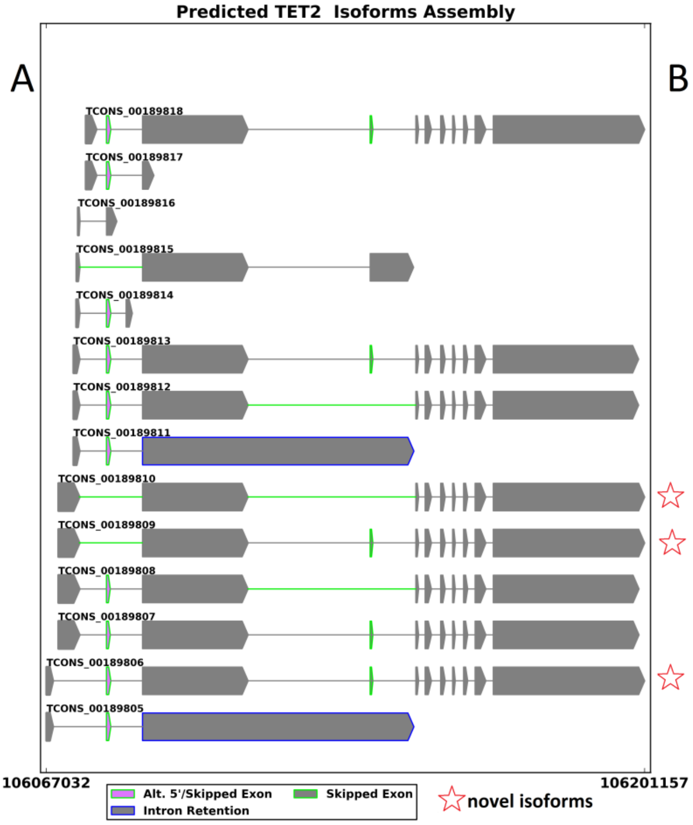

B

Transcript ID	Percent of total pool
TCONS_00189818	Less than 1%
TCONS_00189817	Less than 1%
TCONS_00189816	Less than 1%
TCONS_00189815	Between 1 and 5%
TCONS_00189814	Less than 1%
TCONS_00189813	Between 80 and 90%
TCONS_00189812	Between 1 and 5%
TCONS_00189811	Between 1 and 5%
TCONS_00189810	Less than 1%
TCONS_00189809	Between 5 and 10%
TCONS_00189808	Less than 1%
TCONS_00189807	Less than 1%
TCONS_00189806	Less than 1%
TCONS_00189805	Less than 1%

A

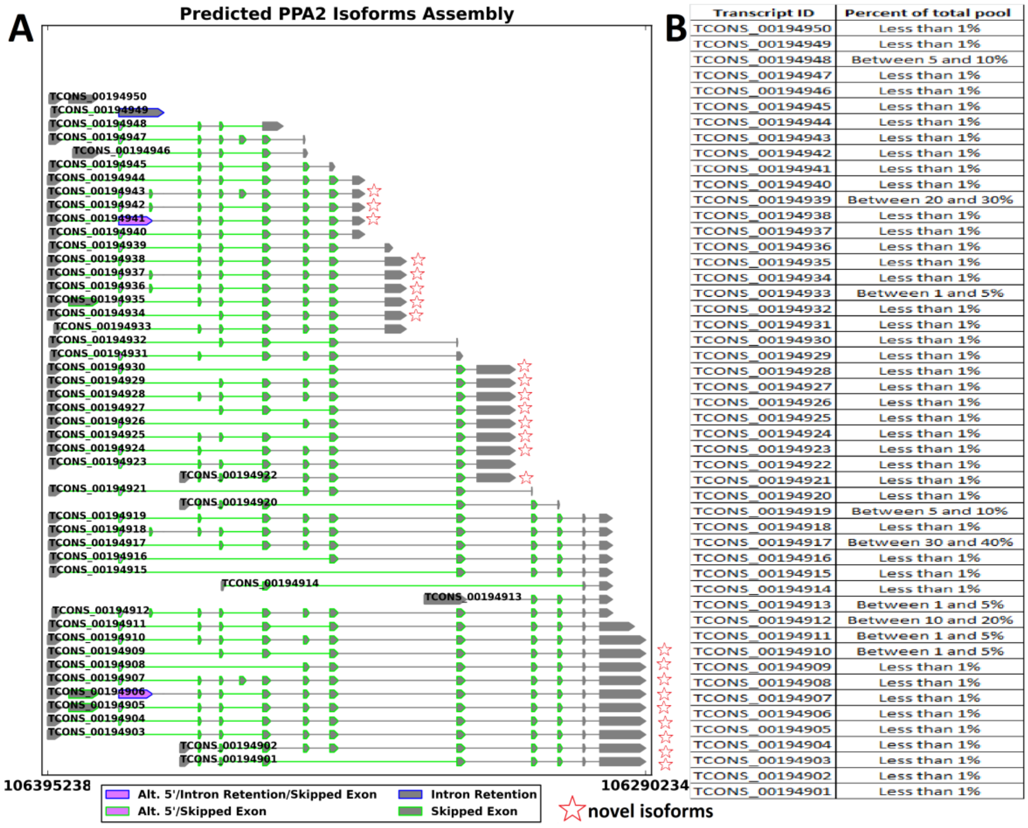

B

Transcript ID	Percent of total pool
TCONS_00194950	Less than 1%
TCONS_00194949	Less than 1%
TCONS_00194948	Between 5 and 10%
TCONS_00194947	Less than 1%
TCONS_00194946	Less than 1%
TCONS_00194945	Less than 1%
TCONS_00194944	Less than 1%
TCONS_00194943	Less than 1%
TCONS_00194942	Less than 1%
TCONS_00194941	Less than 1%
TCONS_00194940	Less than 1%
TCONS_00194939	Between 20 and 30%
TCONS_00194938	Less than 1%
TCONS_00194937	Less than 1%
TCONS_00194936	Less than 1%
TCONS_00194935	Less than 1%
TCONS_00194934	Less than 1%
TCONS_00194933	Between 1 and 5%
TCONS_00194932	Less than 1%
TCONS_00194931	Less than 1%
TCONS_00194930	Less than 1%
TCONS_00194929	Less than 1%
TCONS_00194928	Less than 1%
TCONS_00194927	Less than 1%
TCONS_00194926	Less than 1%
TCONS_00194925	Less than 1%
TCONS_00194924	Less than 1%
TCONS_00194923	Less than 1%
TCONS_00194922	Less than 1%
TCONS_00194921	Less than 1%
TCONS_00194920	Less than 1%
TCONS_00194919	Between 5 and 10%
TCONS_00194918	Less than 1%
TCONS_00194917	Between 30 and 40%
TCONS_00194916	Less than 1%
TCONS_00194915	Less than 1%
TCONS_00194914	Less than 1%
TCONS_00194913	Between 1 and 5%
TCONS_00194912	Between 10 and 20%
TCONS_00194911	Between 1 and 5%
TCONS_00194910	Between 1 and 5%
TCONS_00194909	Less than 1%
TCONS_00194908	Less than 1%
TCONS_00194907	Less than 1%
TCONS_00194906	Less than 1%
TCONS_00194905	Less than 1%
TCONS_00194904	Less than 1%
TCONS_00194903	Less than 1%
TCONS_00194902	Less than 1%
TCONS_00194901	Less than 1%

Supplementary Figure 7: Region plots for novel signals of association with smoking behaviour.

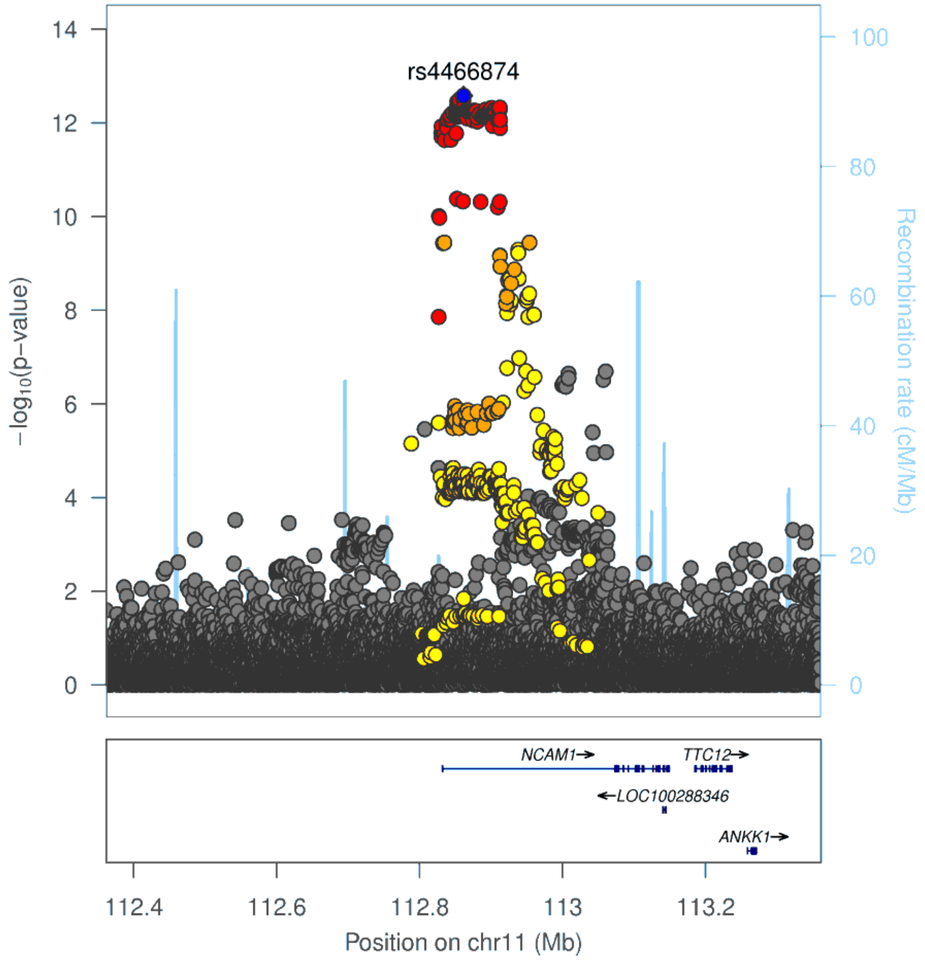

a) *NCAM1*

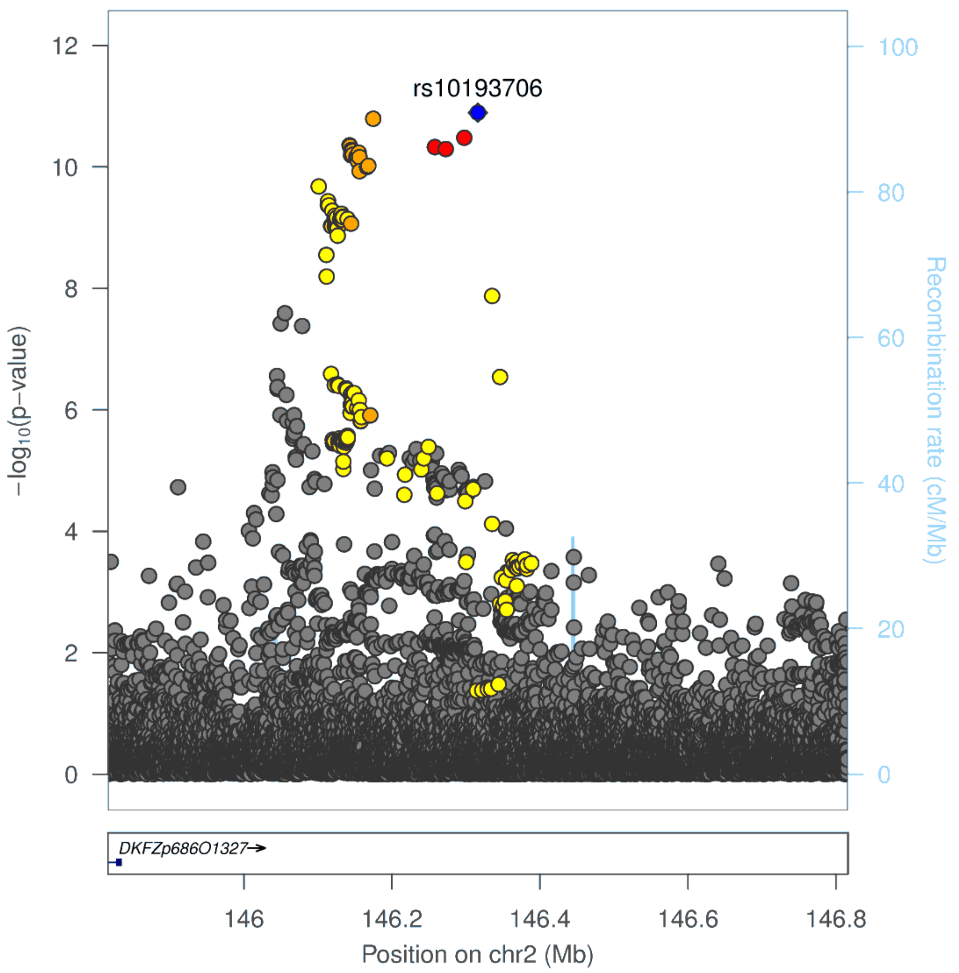

b) *TEX41/PABPC1P2*

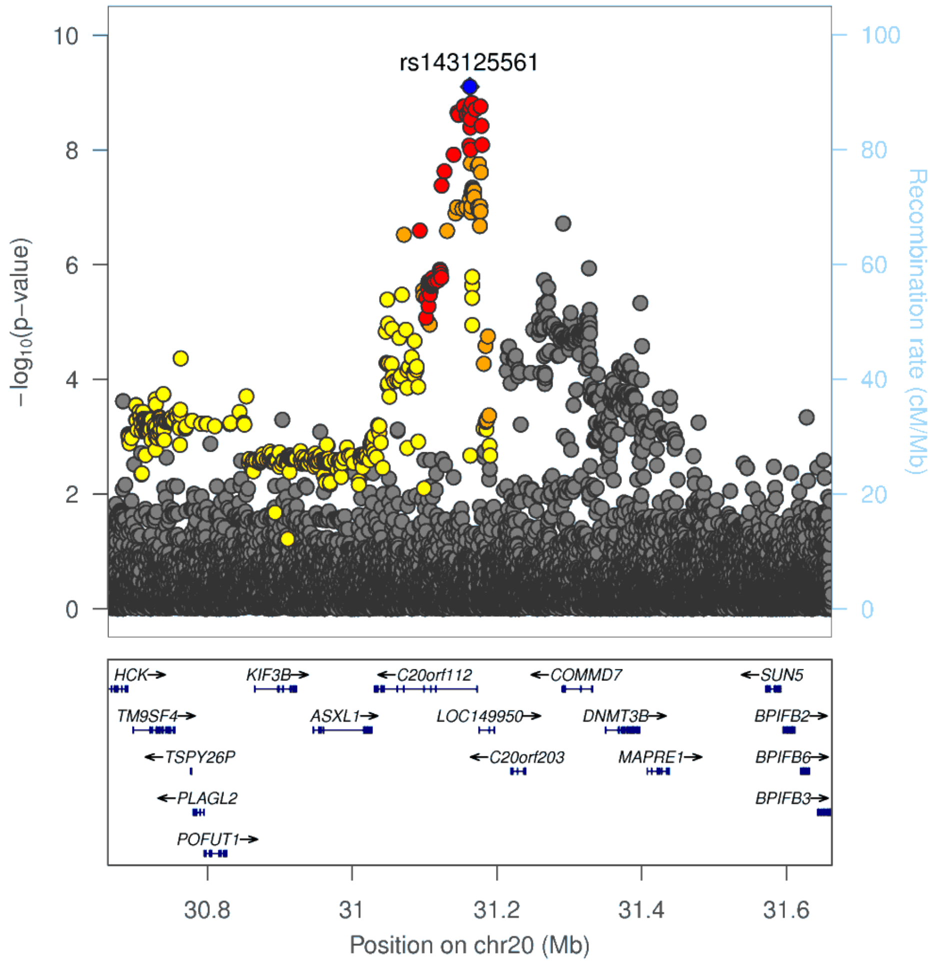

c) *NOL4L* (*C20orf112*)

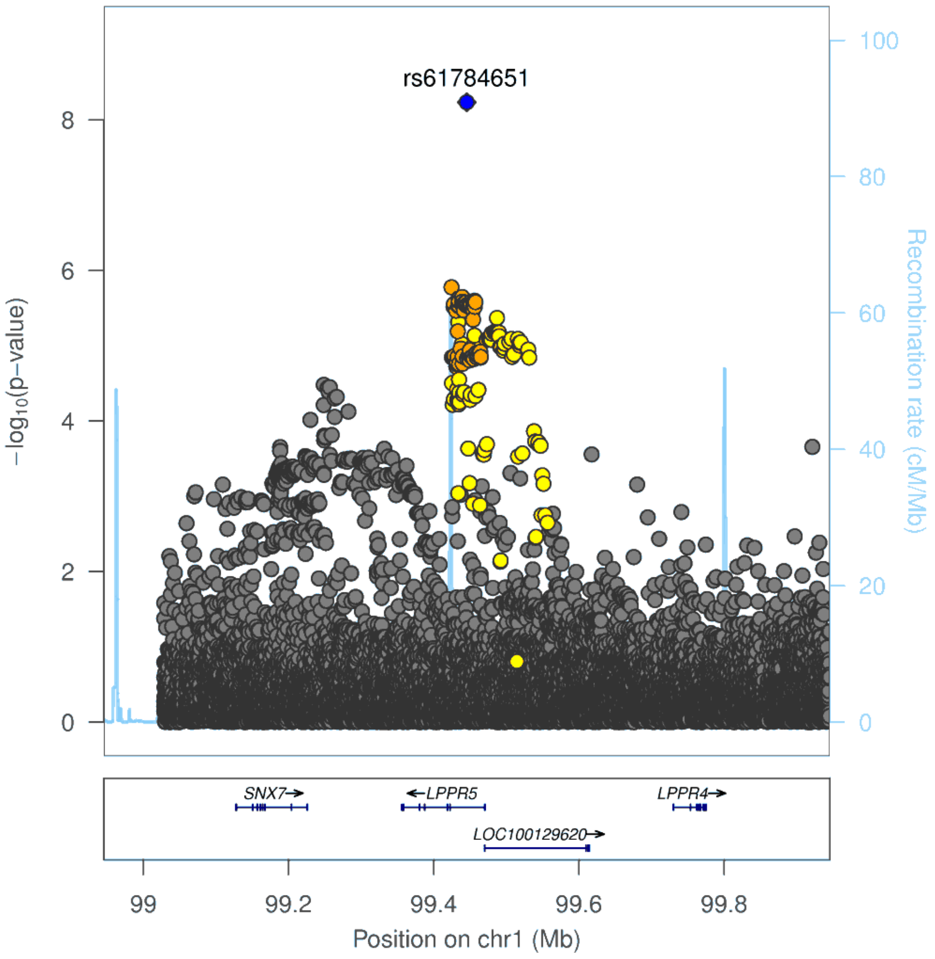

d) *LPPR5*

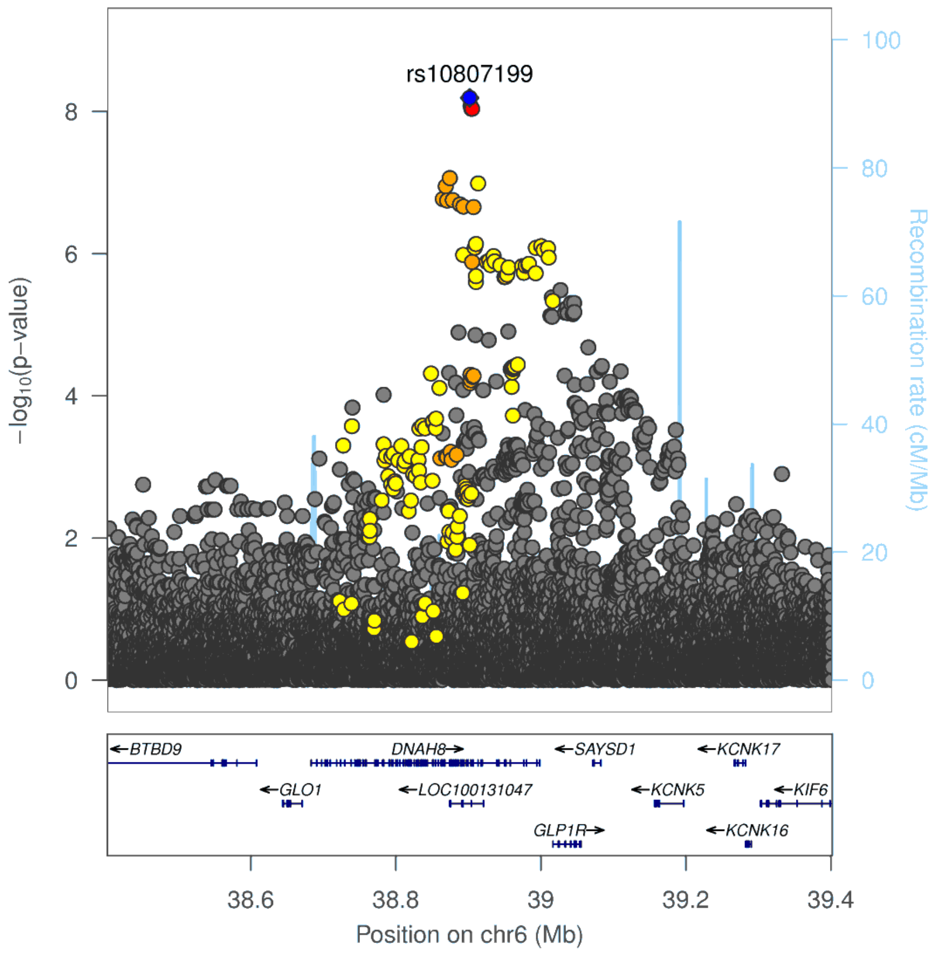
rs10807199
−log10(p−value)
Recombination rate (cM/Mb)
BTBD9
DNAH8
SAYSD1
KCNK17
GLO1
LOC100131047
KCNK5
KIF6
GLP1R
KCNK16
38.6
38.8
39
39.2
39.4
Position on chr6 (Mb)

e) *DNAH8*

Supplementary Figure 8: Quantile-Quantile (QQ) plots for all comparisons of extremes of FEV_1 and smoking behaviour.

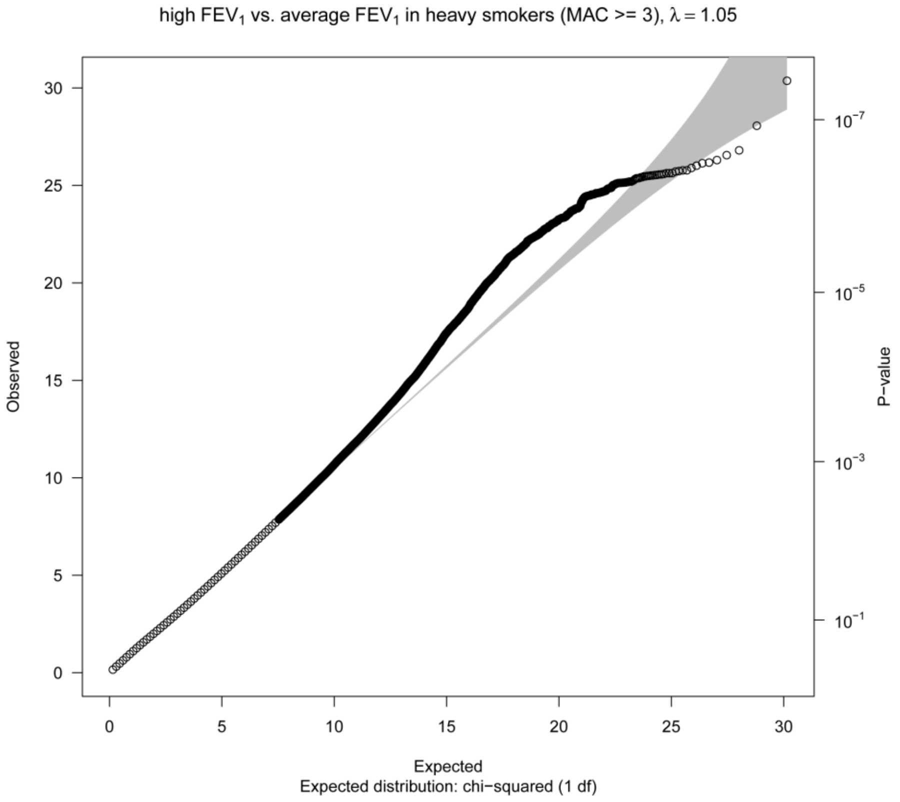

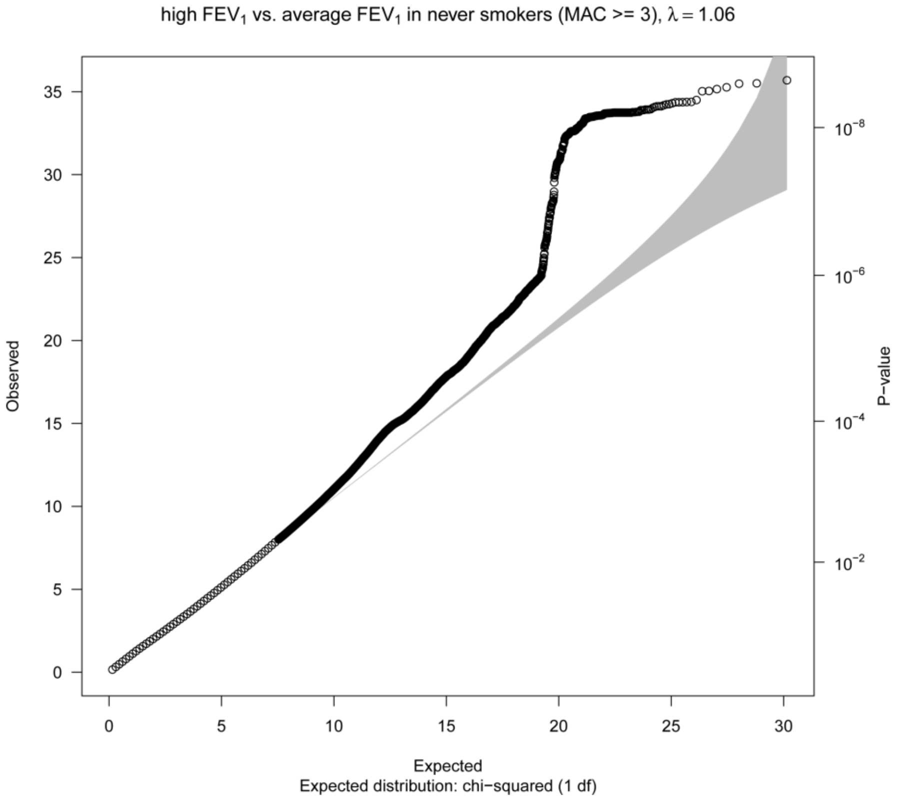

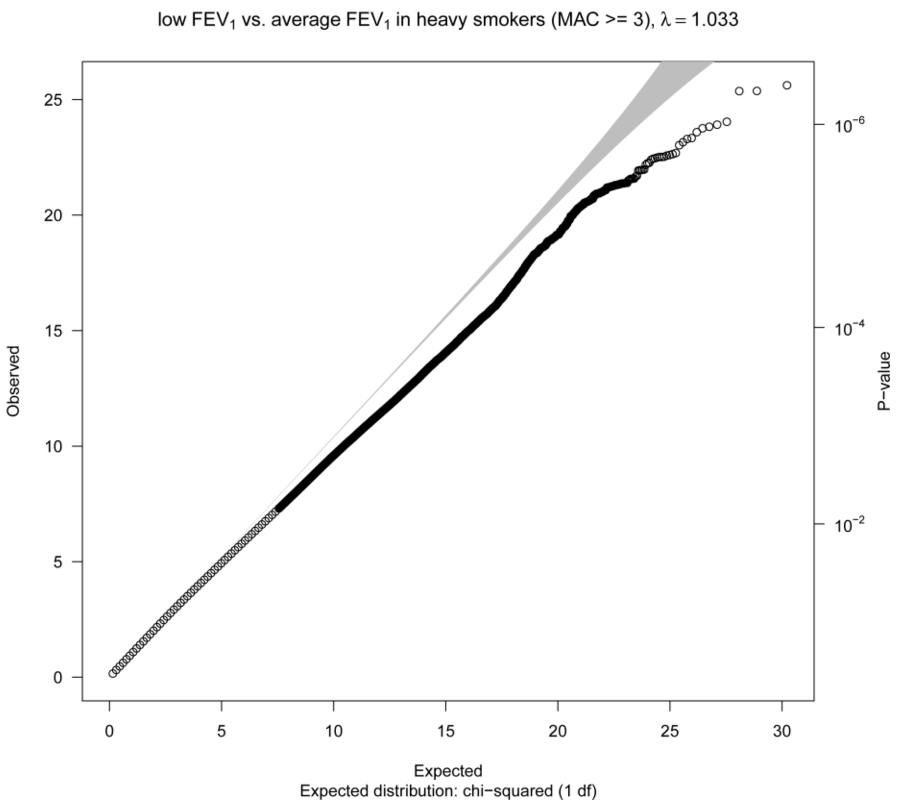
low FEV1 vs. average FEV1 in heavy smokers (MAC >= 3), λ = 1.033
Observed
Expected
Expected distribution: chi−squared (1 df)
P−value

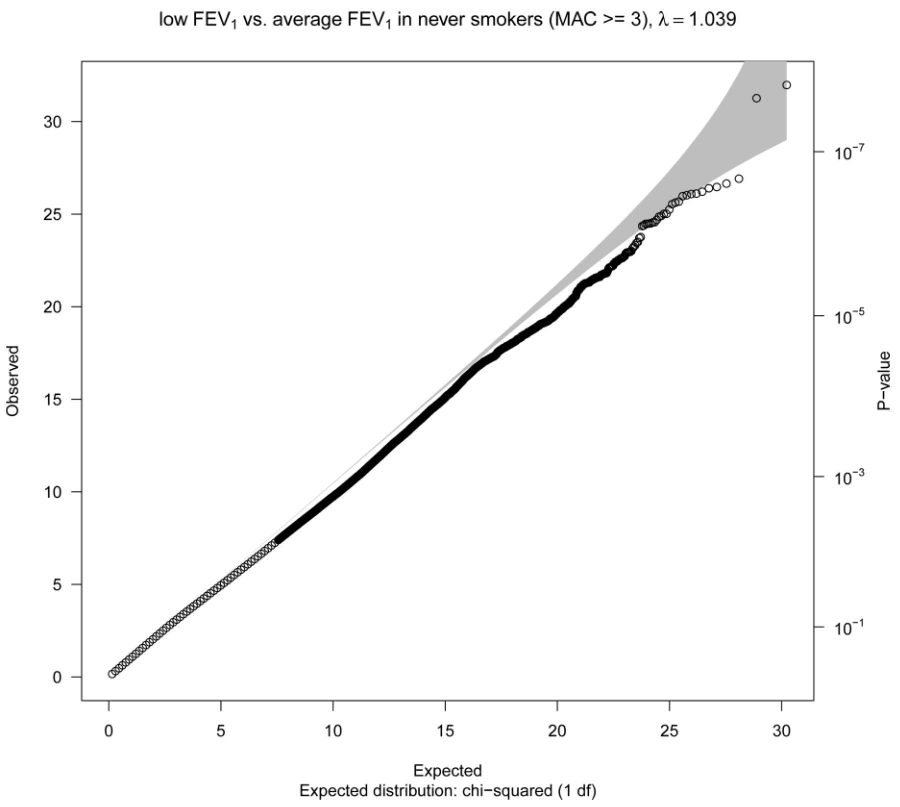
low FEV1 vs. average FEV1 in never smokers (MAC >= 3), λ = 1.039
Observed
Expected
Expected distribution: chi−squared (1 df)
P−value

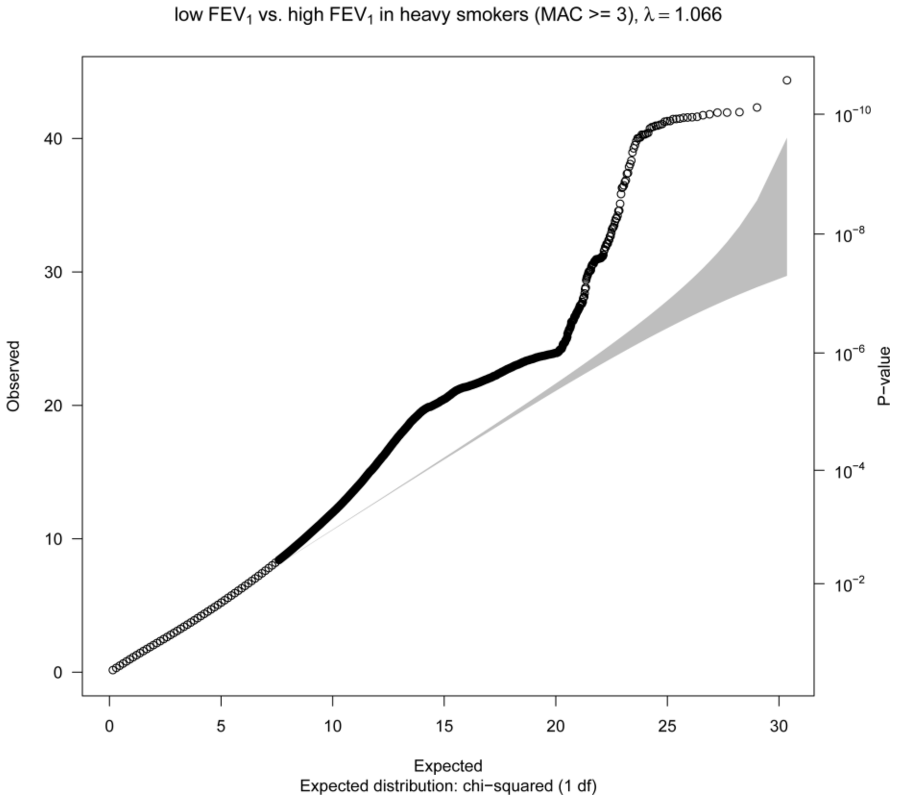

low FEV$_1$ vs. high FEV$_1$ in heavy smokers (MAC >= 3), $\lambda = 1.066$
Observed
Expected
Expected distribution: chi−squared (1 df)
P−value

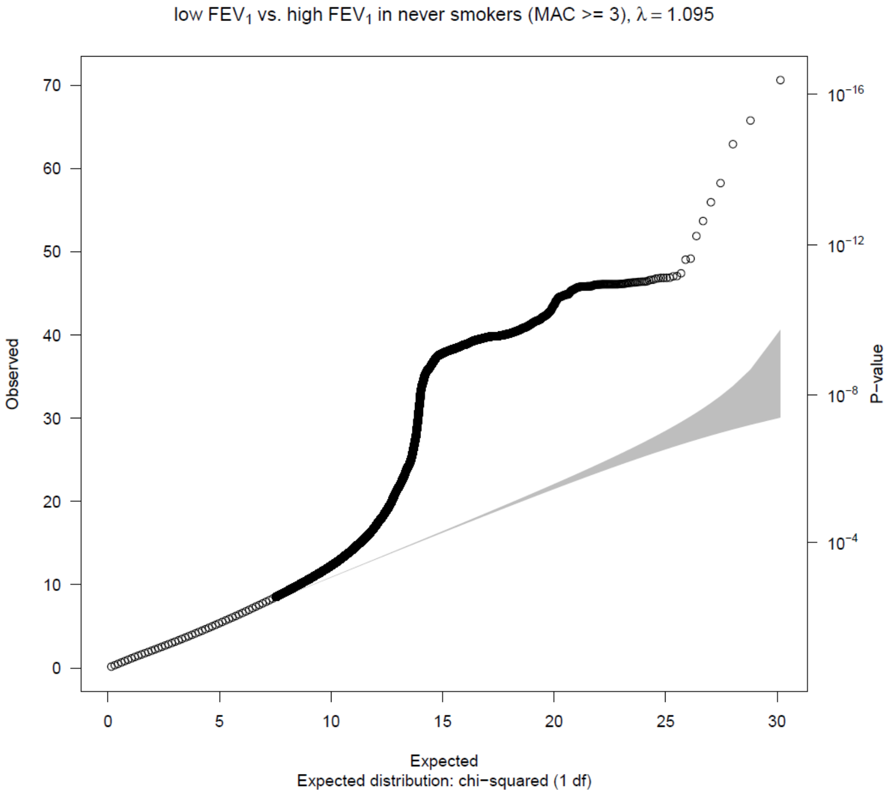

low FEV$_1$ vs. high FEV$_1$ in never smokers (MAC >= 3), $\lambda = 1.095$
Observed
Expected
Expected distribution: chi−squared (1 df)
P−value

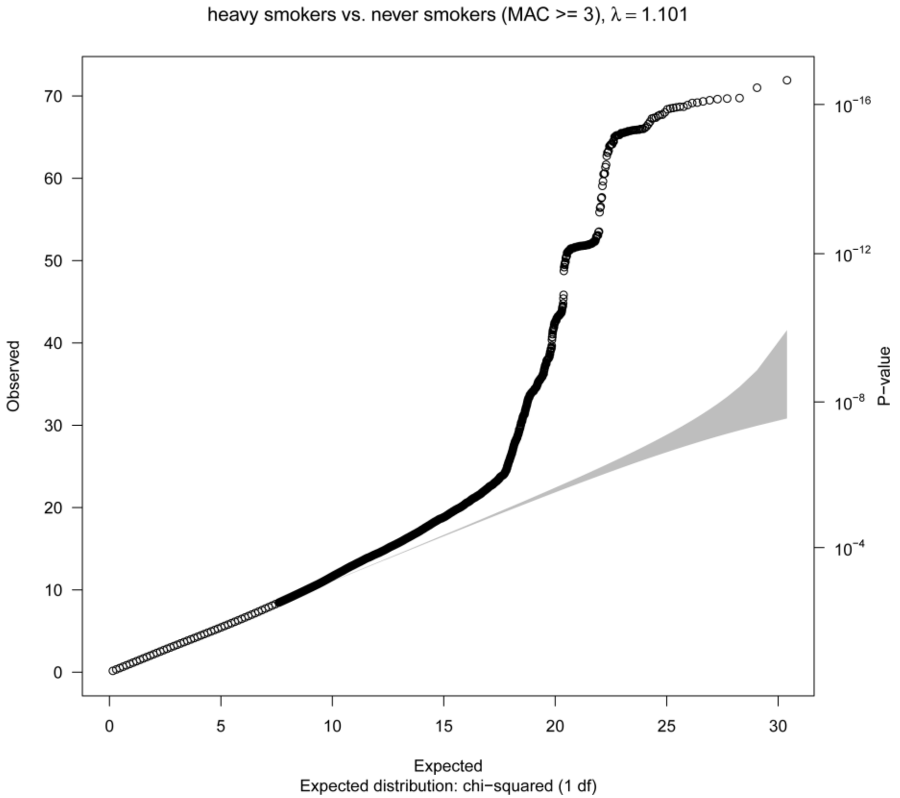
heavy smokers vs. never smokers (MAC >= 3), λ = 1.101
Observed
Expected
Expected distribution: chi−squared (1 df)
P−value
0
10
20
30
40
50
60
70
0
5
10
15
20
25
30
10^{-4}
10^{-8}
10^{-12}
10^{-16}

Supplementary Figure 9: **Single-point and drop-one plots for genes with SKAT-O P < 10-4.** Bars at single-point plots show the variant P value derived from score test. Bars at drop-one plots show the SKAT-O P value after excluding that variant. The vertical line refers to the SKAT-O P value. Minor allele frequency (MAF) bins were derived from the 48,943 samples. Labels above bars are showing the minor allele count for cases and controls (cases/controls). OR: odds ratio.

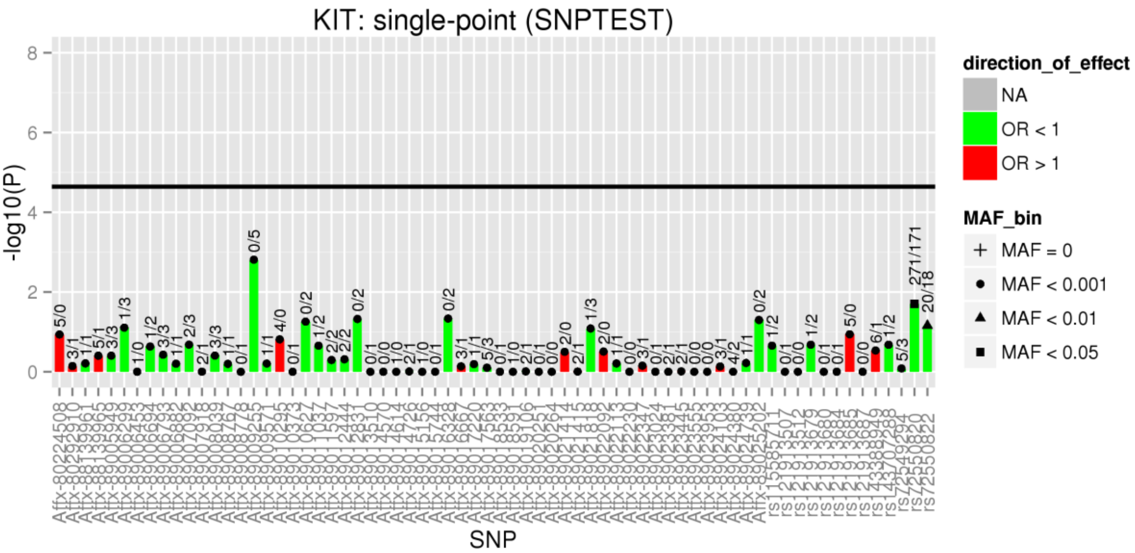

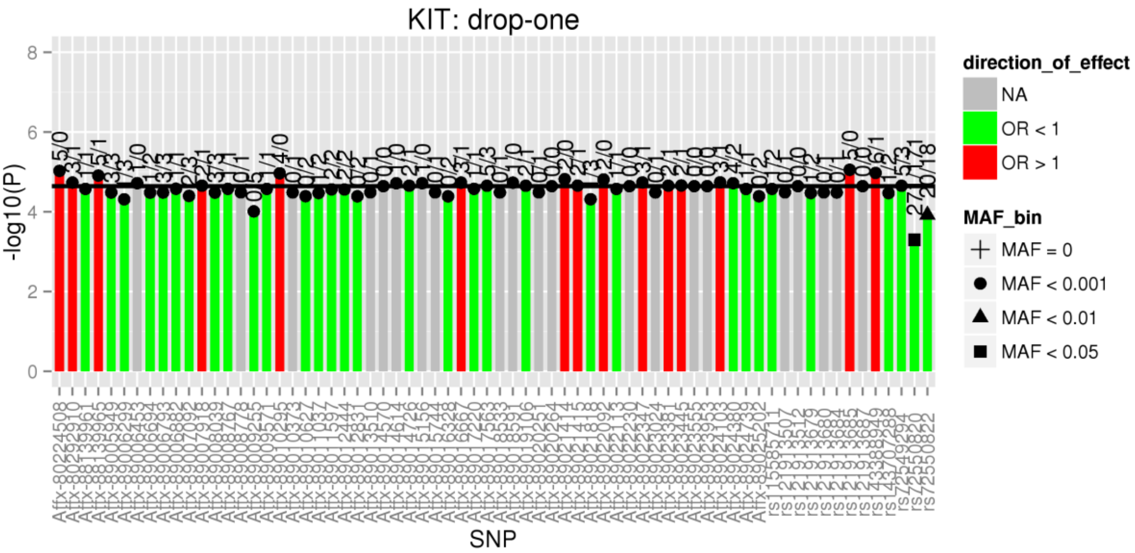

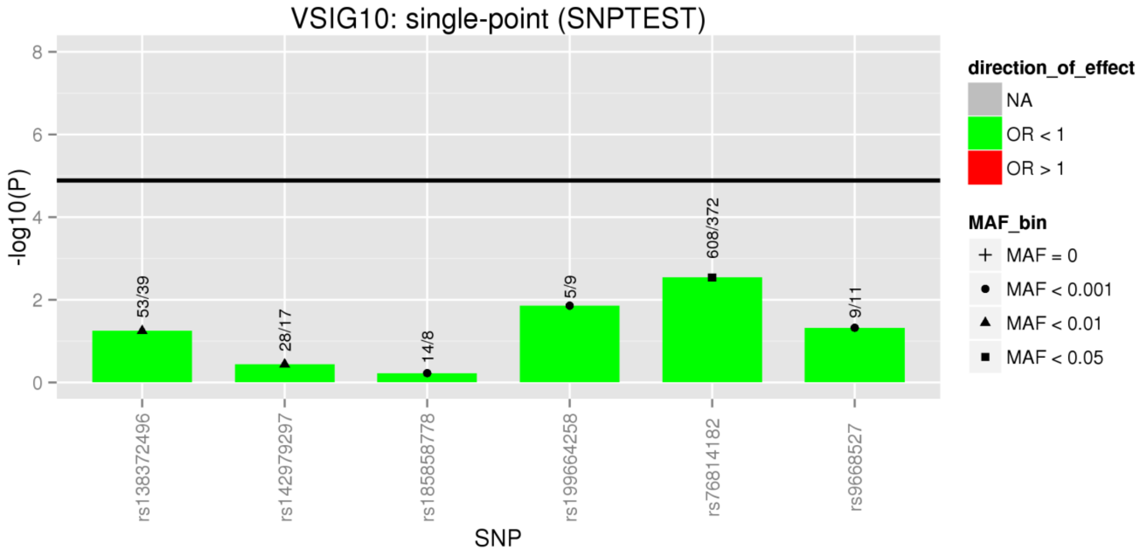
VSIG10: single-point (SNPTEST)
-log10(P)
0
2
4
6
8
53/39
28/17
14/8
5/9
608/372
9/11
rs138372496
rs142979297
rs185858778
rs199664258
rs76814182
rs9668527
SNP
direction_of_effect
NA
OR < 1
OR > 1
MAF_bin
MAF = 0
MAF < 0.001
MAF < 0.01
MAF < 0.05

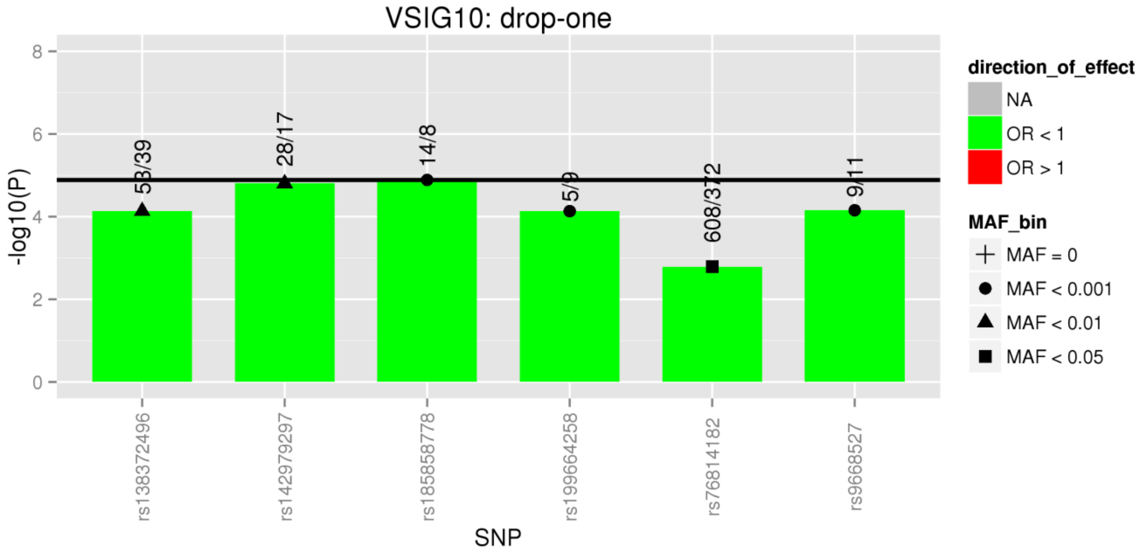
VSIG10: drop-one
-log10(P)
0
2
4
6
8
53/39
28/17
14/8
5/9
608/372
9/11
rs138372496
rs142979297
rs185858778
rs199664258
rs76814182
rs9668527
SNP
direction_of_effect
NA
OR < 1
OR > 1
MAF_bin
MAF = 0
MAF < 0.001
MAF < 0.01
MAF < 0.05

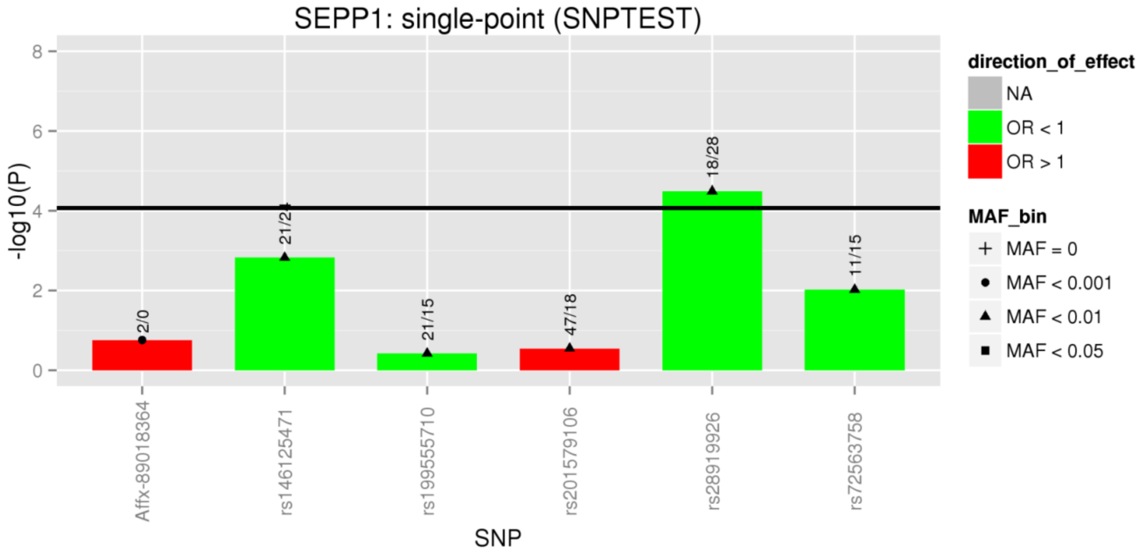
SEPP1: single-point (SNPTEST)
-log10(P)
SNP
Affx-89018364
rs146125471
rs199555710
rs201579106
rs28919926
rs72563758
2/0
21/24
21/15
47/18
18/28
11/15
direction_of_effect
NA
OR < 1
OR > 1
MAF_bin
MAF = 0
MAF < 0.001
MAF < 0.01
MAF < 0.05

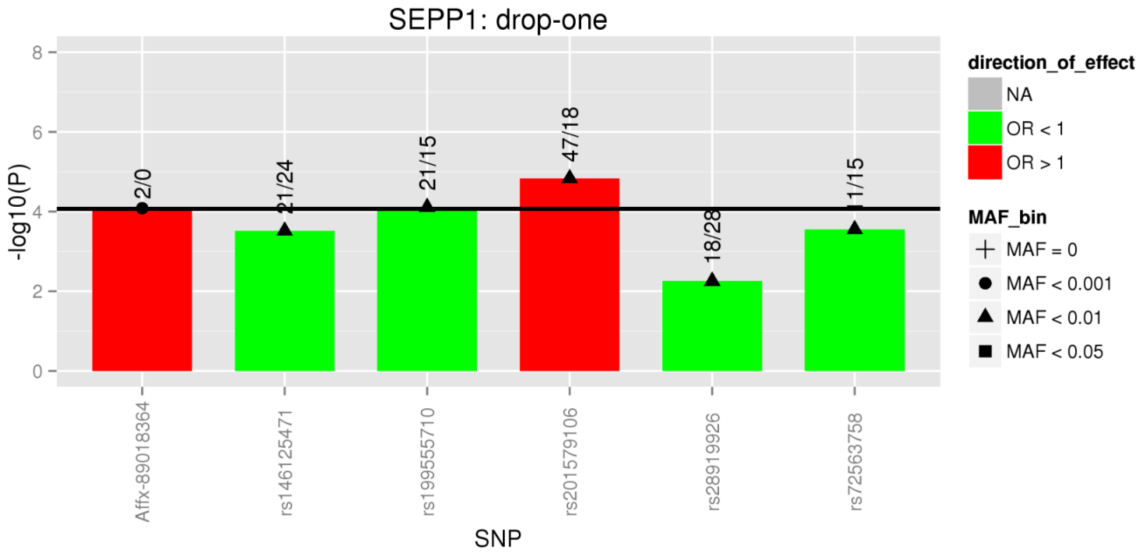
SEPP1: drop-one
-log10(P)
SNP
Affx-89018364
rs146125471
rs199555710
rs201579106
rs28919926
rs72563758
2/0
21/24
21/15
47/18
18/28
11/15
direction_of_effect
NA
OR < 1
OR > 1
MAF_bin
MAF = 0
MAF < 0.001
MAF < 0.01
MAF < 0.05

Supplementary Figure 10: **Manhattan plots for comparisons of high and low FEV_1 with average FEV_1.** Threshold for genome-wide significance ($P < 5\times10^{-8}$) is shown as the dotted red line. Variants with association $P < 5 \times 10^{-7}$ are coloured red. P values are from a Score test and have genomic control applied unless minor allele count (MAC) < 400 and Score $P < 10^{-6}$, in which case P values are from a Firth test with no genomic control. Novel loci are underlined.

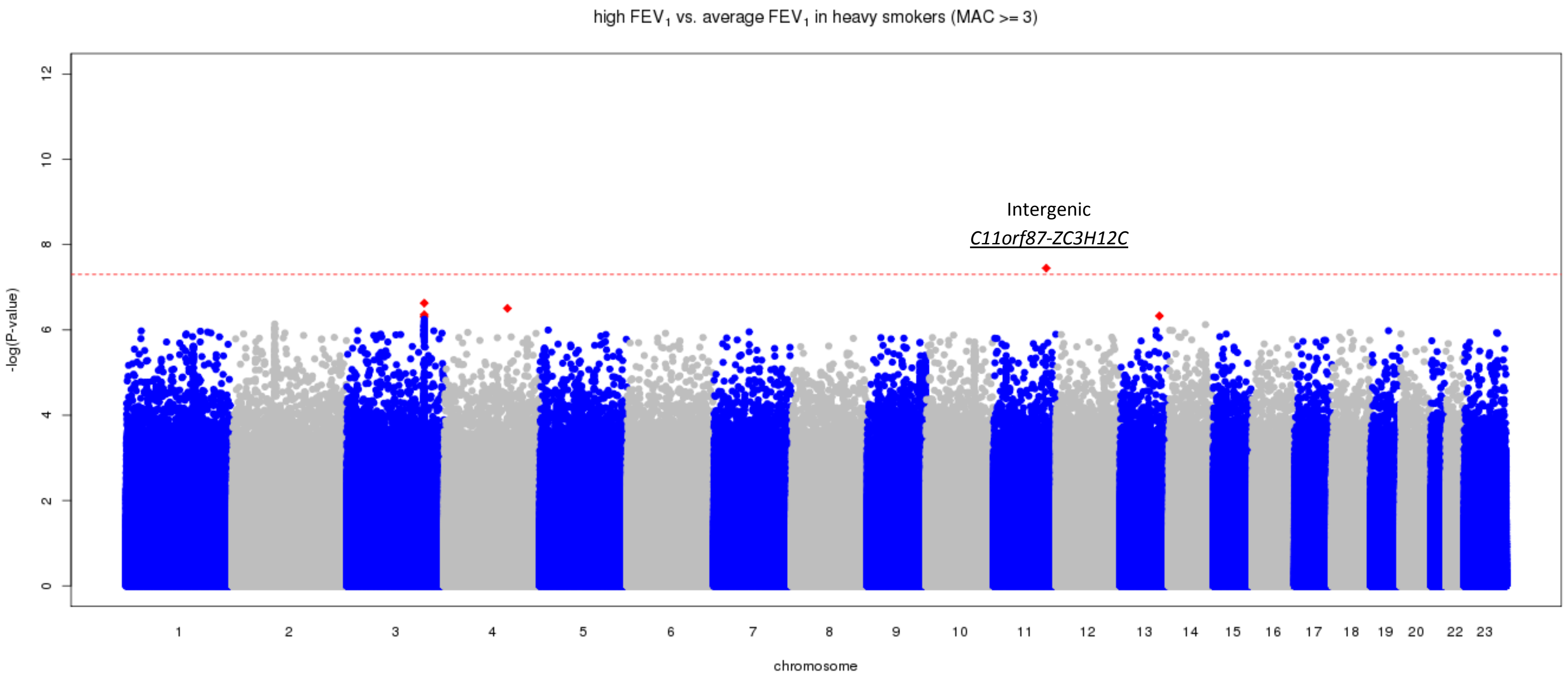

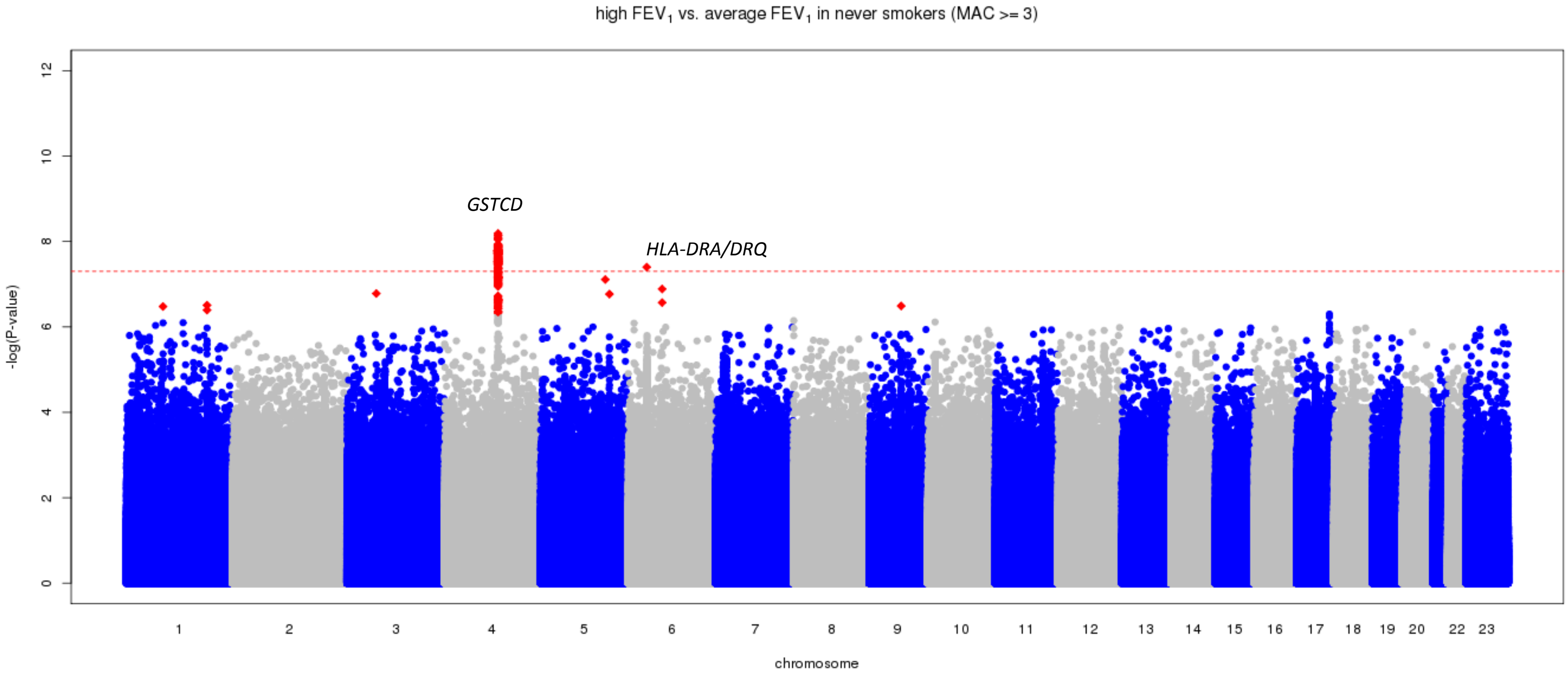
high FEV$_1$ vs. average FEV$_1$ in never smokers (MAC >= 3)
GSTCD
HLA-DRA/DRQ
-log(P-value)
0
2
4
6
8
10
12
1
2
3
4
5
6
7
8
9
10
11
12
13
14
15
16
17
18
19
20
22
23
chromosome

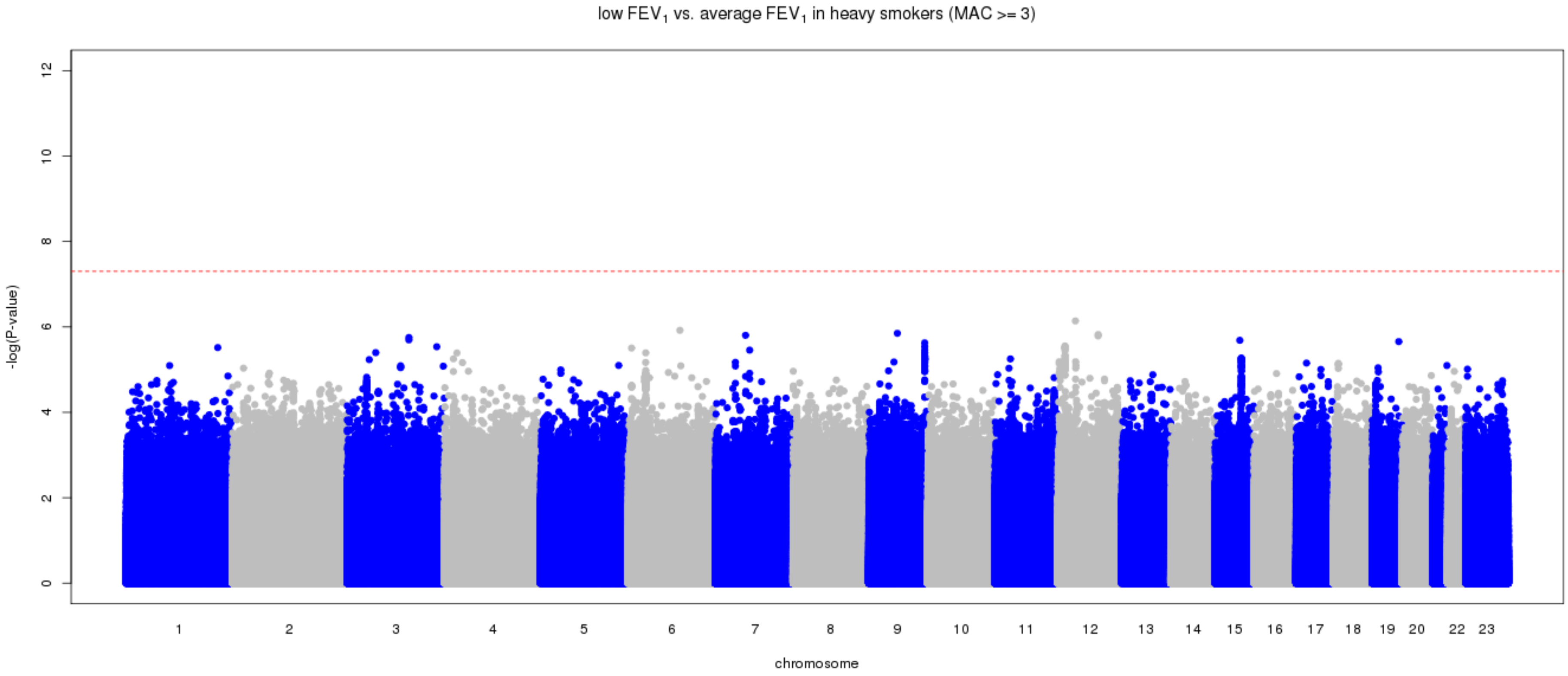
low FEV_1 vs. average FEV_1 in heavy smokers (MAC >= 3)
-log(P-value)
0
2
4
6
8
10
12
1
2
3
4
5
6
7
8
9
10
11
12
13
14
15
16
17
18
19
20
22
23
chromosome

low FEV_1 vs. average FEV_1 in never smokers (MAC >= 3)

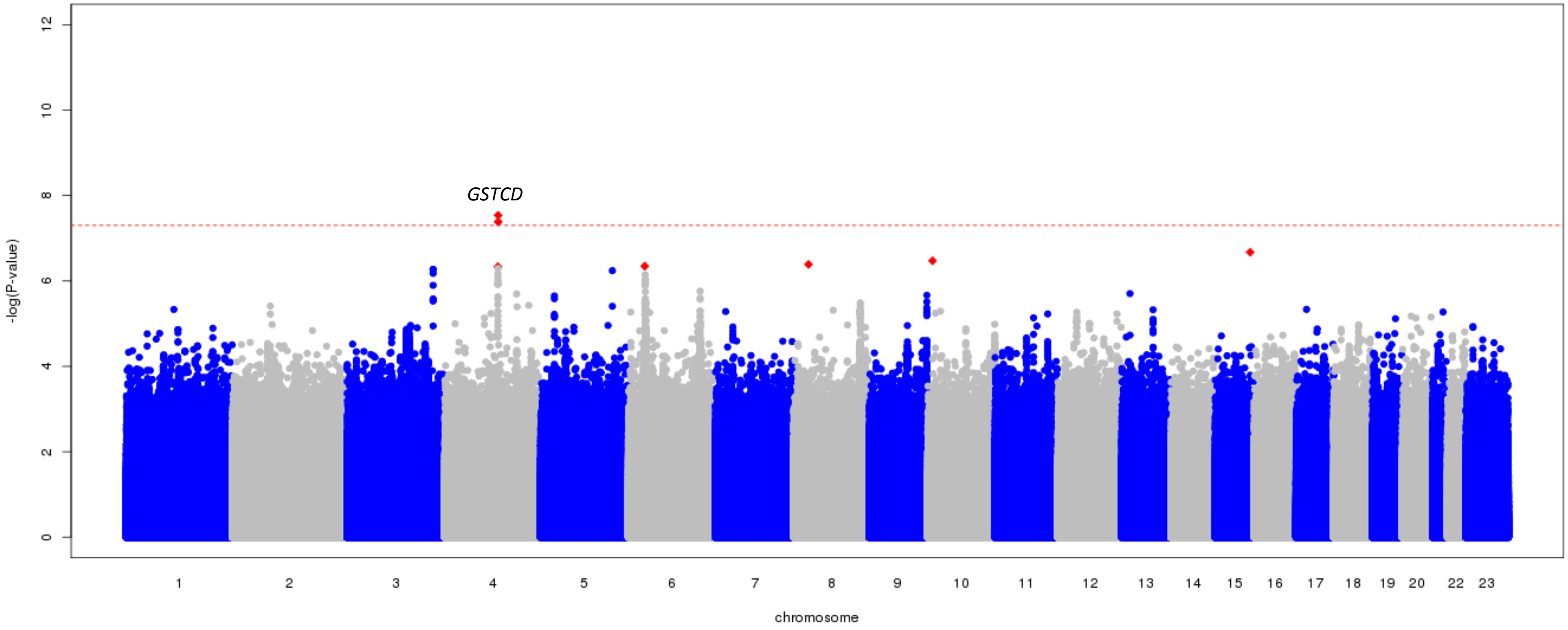

UK Brain Expression Consortium

John A Hardy[1], Michael E Weale[2], Mina Ryten[1,2], Colin Smith[3], Robert Walker[3], Juan Botía[1,2], Jana Vandrocova[1,2], Sebastian Guelfi[1,2], Karishma D'Sa[1,2], Mar Matarin[1], Vibin Varghese[2], Daniah Trabzuni[1], Adaikalavan Ramasamy[1,2,4] and Paola Forabosco[2,5].

[1]Department of Molecular Neuroscience, UCL Institute of Neurology, London WC1N 3BG, UK;

[2]Department of Medical & Molecular Genetics, King's College London SE1 9RT, UK;

[3]Department of Pathology, The University of Edinburgh, Wilkie Building, Teviot Place, Edinburgh EH8 9AG, UK;

[4]Jenner Institute, University of Oxford, Oxford OX3 7DQ, UK;

[5]Istituto di Ricerca Genetica e Biomedica, Cittadella Universitaria di Cagliari, 09042 Monserrato, Sardinia, Italy.

OxGSK Consortium

Jason Z Liu[1], Federica Tozzi[2], Dawn M Waterworth[3], Sreekumar G Pillai[3], Pierandrea Muglia[2], Lefkos Middleton[4], Wade Berrettini[5], Christopher W Knouff[6], Xin Yuan[3], Gérard Waeber[7,8], Peter Vollenweider[7, 8], Martin Preisig[7,9], Nicholas J Wareham[10], Jing Hua Zhao[10], Ruth J F Loos[10], Inês Barroso[11], Kay-Tee Khaw[12], Scott Grundy[13], Philip Barter[14], Robert Mahley[15,16], Antero Kesaniemi[17,18], Ruth McPherson[19], John B Vincent[20], John Strauss[20], James L Kennedy[20], Anne Farmer[21], Peter McGuffin[21], Richard Day[22], Keith Matthews[22], Per Bakke[23], Amund Gulsvik[23], Susanne Lucae[24], Marcus Ising[24], Tanja Brueckl[24], Sonja Horstmann[24], H-Erich Wichmann[25,26,27], Rajesh Rawal[25], Norbert Dahmen[28], Claudia Lamina[25,29], Ozren Polasek[30], Lina Zgaga[31], Jennifer Huffman[32], Susan Campbell[32], Jaspal Kooner[33], John C Chambers[34], Mary Susan Burnett[35], Joseph M Devaney[35], Augusto D Pichard[35], Kenneth M Kent[35], Lowell Satler[35], Joseph M Lindsay[35], Ron Waksman[35], Stephen Epstein[35], James F Wilson[31], Sarah H Wild[31], Harry Campbell[31], Veronique Vitart[32], Muredach P Reilly[36, 37], Mingyao Li[38], Liming Qu[38], Robert Wilensky[36], William Matthai[36], Hakon H Hakonarson[39], Daniel J Rader[36, 37], David Ellinghaus[40], Wolfgang Lieb[41], Andre Franke[40], Manuela Uda[42], Antonio Terracciano[43], Xiangjun Xiao[44], Fabio Busonero[42], Paul Scheet[44], David Schlessinger[43], David St Clair[45], Dan Rujescu[46], Gonçalo R Abecasis[47], Hans Jörgen Grabe[48], Alexander Teumer[49], Henry Völzke[50], Astrid Petersmann[51], Ulrich John[52], Igor Rudan[53,31], Caroline Hayward[32], Alan F Wright[32], Ivana Kolcic[30], Benjamin J Wright[54], John R Thompson[54], Anthony J Balmforth[55], Alistair S Hall[55], Nilesh J Samani[56], Carl A Anderson[11], Tariq Ahmad[57], Christopher G Mathew[58], Miles Parkes[59], Jack Satsangi[60], Mark Caulfield[61], Patricia B Munroe[61], Martin Farrall[62], Anna Dominiczak[63], Jane Worthington[64], Wendy Thomson[64], Steve Eyre[64], Anne Barton[64], The Wellcome Trust Case Control Consortium[65], Vincent Mooser[3], Clyde Francks[66, 67], Jonathan Marchini[1]

1. Department of Statistics, University of Oxford, Oxford, UK.
2. Genetics Division, GlaxoSmithKline, Verona, Italy.
3. Genetics Division, GlaxoSmithKline, Upper Merion, Pennsylvania, USA.
4. Division of Neurosciences and Mental Health, Imperial College London, UK.
5. Department of Psychiatry, University of Pennsylvania School of Medicine, Philadelphia, Pennsylvania, USA.
6. Genetics Division, GlaxoSmithKline, Research Triangle Park, North Carolina, USA.
7. Faculty of Biology and Medicine, University of Lausanne, 1011 Lausanne, Switzerland.
8. Department of Internal Medicine, University Hospital of Lausanne, 1011 Lausanne, Switzerland.
9. Department of Psychiatry, University Hospital of Lausanne, 1011 Lausanne, Switzerland.
10. Medical Research Council Epidemiology Unit, Institute of Metabolic Science, Cambridge, UK.
11. Wellcome Trust Sanger Institute, Hinxton, UK.
12. Department of Public Health and Primary Care, University of Cambridge, Cambridge, UK.
13. Center for Human Nutrition, University of Texas Southwestern Medical Center, Dallas, Texas, USA.
14. The Heart Research Institute, Sydney, New South Wales, Australia.
15. Gladstone Institute of Cardiovascular Disease, University of California, San Francisco, California, USA.
16. American Hospital, Istanbul, Turkey.
17. Department of Internal Medicine, University of Oulu, Oulu, Finland.
18. Biocenter Oulu, University of Oulu, Oulu, Finland.
19. Division of Cardiology, University of Ottawa Heart Institute, Ottawa, Ontario, Canada.
20. Centre for Addiction and Mental Health, University of Toronto, Toronto, Ontario, Canada.
21. Medical Research Council Social, Genetic and Developmental Psychiatry Centre, Institute of Psychiatry, King's College London, London, UK.
22. Center for Neuroscience, Division of Medical Sciences, University of Dundee, Dundee, UK.
23. Institute of Medicine, University of Bergen, Bergen, Norway.
24. Max Planck Institute of Psychiatry, Munich, Germany.
25. Institute of Epidemiology, Helmholtz Zentrum München, German Research Center for Environmental Health, Neuherberg, Germany.
26. Institute of Medical Informatics, Biometry and Epidemiology, Ludwig-MaximiliansUniversität, Munich, Germany.
27. Klinikum Grosshadern, Munich, Germany.
28. Psychiatrische Klinik und Poliklinik University of Mainz, Mainz, Germany.
29. Division of Genetic Epidemiology, Department of Medical Genetics, Molecular and Clinical Pharmacology, Innsbruck Medical University, Innsbruck, Austria.
30. School of Public Health, School of Medicine, University of Zagreb, Croatia.
31. Centre for Population Health Sciences, University of Edinburgh, Edinburgh, UK.
32. Institute of Genetics and Molecular Medicine, MRC Human Genetics Unit, Edinburgh, UK.
33. National Heart and Lung Institute, Imperial College London, London, UK.
34. Division of Epidemiology, Imperial College London, London, UK.

35. Cardiovascular Research Institute, MedStar Research Institute, Washington Hospital Center, Washington DC, USA.
36. The Cardiovascular Institute, University of Pennsylvania, Philadelphia, Pennsylvania, USA.
37. The Institute for Translational Medicine and Therapeutics, School of Medicine, University of Pennsylvania, Philadelphia, Pennsylvania, USA.
38. Biostatistics and Epidemiology, University of Pennsylvania, Philadelphia, Pennsylvania, USA.
39. The Center for Applied Genomics, Children's Hospital of Philadelphia, Philadelphia, Pennsylvania, USA.
40. Institute of Clinical Molecular Biology, ChristianAlbrechts-University of Kiel, Kiel, Germany.
41. Institute of Epidemiology and Biobank Popgen, Christian-Albrechts-University of Kiel, Kiel, Germany.
42. Istituto di Neurogenetica e Neurofarmacologia, Consiglio Nazionale delle Ricerche, Monserrato, Cagliari, Italy.
43. National Institute on Aging, Baltimore, Maryland, USA.
44. Department of Epidemiology, University of Texas M.D. Anderson Cancer Center, Houston, Texas, USA.
45. Department of Mental Health, University of Aberdeen, Aberdeen, UK.
46. Division of Molecular and Clinical Neurobiology, Department of Psychiatry, Ludwig-Maximilians-University, Munich, Germany.
47. Center for Statistical Genetics, Department of Biostatistics, University of Michigan, Ann Arbor, Michigan, USA.
48. Department of Psychiatry and Psychotherapy, University of Greifswald, Greifswald, Germany.
49. Interfacultary Institute for Genetics and Functional Genomics, University of Greifswald, Greifswald, Germany.
50. Institute for Community Medicine, University of Greifswald, Greifswald, Germany.
51. Institute of Clinical Chemistry and Laboratory Medicine, University of Greifswald, Greifswald, Germany.
52. Department of Social Medicine and Epidemiology, University of Greifswald, Greifswald, Germany.
53. Croatian Centre for Global Health, University of Split, Split, Croatia.
54. Department of Health Sciences, University of Leicester, Leicester, UK.
55. Mulitdisciplinary Cardiovascular Research Centre (MCRC), Leeds Institute of Genetics, Health and Therapeutics (LIGHT), University of Leeds, Leeds, UK.
56. Department of Cardiovascular Sciences, University of Leicester, Glenfield Hospital, Leicester, UK.
57. Peninsula College of Medicine and Dentistry, Exeter, UK.
58. Department of Medical and Molecular Genetics, King's College London School of Medicine, Guy's Hospital, London, UK.
59. Gastroenterology Research Unit, Addenbrooke's Hospital, Cambridge, UK.
60. Gastrointestinal Unit, Molecular Medicine Centre, University of Edinburgh, Western General Hospital, Edinburgh, UK.
61. Clinical Pharmacology and Barts and the London Genome Centre, William Harvey Research Institute, Barts and the London School of Medicine, Queen Mary University of London, London, UK.
62. Department of Cardiovascular Medicine, University of Oxford, Wellcome Trust Centre for Human Genetics, Oxford, UK.
63. British Heart Foundation Glasgow Cardiovascular Research Centre, Division of Cardiovascular and Medical Sciences, University of Glasgow, Western Infirmary, Glasgow, UK.
64. Arthritis Research UK Centre for Genetics and Genomics, University of Manchester, Manchester Academic Health Sciences Centre, Stopford Building, Oxford Rd, Manchester, M13 9PT, UK.
65. A full list of members is provided in the **Supplementary Material**.
66. Language & Genetics Department, Max Planck Institute for Psycholinguistics, Nijmegen, The Netherlands.
67. Donders Institute for Brain, Cognition and Behaviour, Radboud University Nijmegen, Nijmegen, The Netherlands.

Appendix 1:

UK Biobank Unique Identifiers (UDIs) used to select individuals for UK BiLEVE.

**Variable**	**UK Biobank Unique Data Identifier**	**Notes**
Ethnicity	21000	
FEV_1	3063	
FVC	3062	
Acceptability of each blow result	3061	Used for determining whether met ATS/ERS criteria.
Spirometry Method	23	
Age at recruitment	21003	
Sex	31	
Standing Height	50	
Current tobacco smoking	1239	Yes, on most or all days / Only Occasionally / No / Prefer not to answer.
Past tobacco smoking	1249	Smoked on most or all days / Smoked occasionally / Just tried once or twice / I have never smoked / Prefer not to answer.
Type of tobacco currently smoked (current smokers)	3446	Manufactured cigarettes / Hand-rolled cigarettes / Cigars or pipes / Prefer not to answer.
Type of tobacco previously smoked (former smokers)	2877	Manufactured cigarettes / Hand-rolled cigarettes / Cigars or pipes / Prefer not to answer.
Previously smoked cigarettes on most/all days (current pipe/cigar smokers)	5959	Asked if answered "Yes, on most or all days" to 1239 and "Cigars or pipes" to 3446.
Age started smoking (current smokers)	3436	
Age started smoking (former smokers)	2867	
Age stopped smoking (former smokers)	2897	
Age stopped smoking cigarettes (current pipe/cigar smokers)	6194	
Number of cigarettes smoked daily (current smokers)	3456	
Number of cigarettes previously smoked daily (former smokers)	2887	
Number of cigarettes previously smoked daily (current pipe/cigar smokers)	6183	
Ever stopped smoking for 6+ months	2907	
Blood clot, DVT, bronchitis, emphysema, asthma, rhinitis, eczema, allergy diagnosed by doctor	6152	Excluded from "healthy never smokers" if answered bronchitis, emphysema or asthma.
Wheeze or whistling in the chest in last year	2316	Excluded from "healthy never smokers" if answered yes/don't know or prefer not to answer.
Self-reported non-cancer illness	20002	Excluded from "healthy never smokers" if answered any of the following: asthma; copd; emphysema; chronic bronchitis; bronchiectasis; interstitial lung disease; asbestosis; pulmonary fibrosis; fibrosing /unspecified alveolitis; respiratory failure; pleurisy; spontaneous/recurrent pneumothorax; other respiratory problems

OxGSK Consortium information

OxGSK Consortium contributing cohort acknowledgements:
GSK Bipolar (Bipolar depression case-control): We thank the participants who donated their time and DNA to make this study possible; the staff at the recruiting sites in London, Toronto, and Dundee, and at GlaxoSmithKline for contributions to recruitment and study management.
The EPIC-Obesity case-control study: The EPIC Norfolk Study is funded by program grants from the Medical Research Council UK and Cancer Research UK; and by additional support from the European Union; Stroke Association; British Heart Foundation; Department of Health; Food Standards Agency; and the Wellcome Trust.
KORA: HEW is supported by the German Federal Ministry of Education and Research (BMBF) in the context of the German National Genome Research Network (NGFN-2 and NGFN-plus). The KORA research platform was initiated and financed by the Helmholtz Center Munich, German Research Center for Environmental Health, which is funded by the German Federal Ministry of Education and Research and by the State of Bavaria. Our research was supported within the Munich Center of Health Sciences (MC Health) as part of LMUinnovativ.
GSK UPD (Unipolar depression case-control): We would like to acknowledge all the participants in the study. We would like to thank numerous people at GSK and Max-Planck Institute, BKH Augsburg and Klinikum Ingolstadt in Germany who have contributed to this project.
Authors gratefully acknowledge the assistance for the **Bergen case-control study**: Trude Duelien Skorge, Borghild Hovland, Marianne Salomonsen, Tina Endresen, Rita Opeland, Gunn Nøstdal and Meera Gummaraja of the Institute of Medicine, University of Bergen and Mark Hall, Sandra Hammond, Rachel Taylor, Sara Alalouf and Santhi Subramanian of GlaxoSmithKline for data management support.
The **CoLaus|PsyCoLaus** study was and is supported by research grants from the Faculty of Biology and Medicine of Lausanne, the Swiss National Science Foundation (grants 3200B0–105993, 3200B0-118308, 33CSCO-122661, 33CS30-139468 and 33CS30-148401) and from GlaxoSmithKline (Psychiatry Center of Excellence for Drug Discovery and Genetics Division, Drug Discovery - Verona, R&D). The authors would like to express their gratitude to the Lausanne inhabitants who volunteered to participate in the CoLaus study.
POPGEN (The Popgen Biobank Study): This study was supported by the German Ministry of Education and Research (BMBF) through the e:Med consortium sysINFLAME and the project received infrastructure support through the DFG excellence cluster "Inflammation at Interfaces“.
The PopGen 2.0 network is supported by a grant from the German Federal Ministry of Education and Research (01EY1103).”
The PopGen project received infrastructure support through the German Research Foundation excellence cluster “Inflammation at Interfaces” (EXC306/2)

Recruitment of **PennCATH (Coronary artery disease case-control)** was supported by the Cardiovascular Institute of the University of Pennsylvania. Recruitment of the **MedStar** sample was supported in part by the MedStar Research Institute and the Washington Hospital Center and a research grant from GlaxoSmithKline. Genotyping of PennCATH and Medstar was performed at the Center for Applied Genomics at the Children’s Hospital of Philadelphia and supported by GlaxoSmithKline through an Alternate Drug Discovery Initiative research alliance award (M. P. R. and D. J. R.) with the University of Pennsylvania School of Medicine.
BRIGHT study (WTCCC-HT Hypertension cases): We are extremely grateful to all the patients who participated in the study and the nursing team. The BRIGHT study is supported by the Medical Research Council of Great Britain and the British Heart Foundation. Professor Dominiczak is a British Heart Foundation Chairholder. This work forms part of the research themes contributing to the translational research portfolio of Barts and the London Cardiovascular Biomedical Research Unit which is supported and funded by the National Institute of Health Research.
Collection of samples for the **WTCCC-RA (Rheumatoid Arthritis cases)** cohort was funded by The Arthritis Research Campaign. We are grateful to the patients, rheumatologists, nurses and laboratory staff who contributed to the ascertainment and preparation of these samples. This study makes use of data generated by the Wellcome Trust Case Control Consortium. A full list of the investigators who contributed to the generation of the data is available from www.wtccc.org.uk. Funding for the project was provided by the Wellcome Trust under award 076113.
Recruitment of the **WTCCC-CAD cases (WTCCC-CHD)** was funded by the British Heart Foundation and the UK Medical Research Council and the GWAS study by the Wellcome Trust. N.J.S. holds a chair funded by the British Heart Foundation.
The UK IBD Genetics Consortium (WTCCC-CD, Crohn’s disease cases): We thank all Crohn’s disease subjects who contributed samples, and consultants and nursing staff across the UK who helped with recruitment

of study subjects. Case collections were supported by the National Association for Colitis and Crohn's disease (NACC), the Wellcome Trust, the Medical Research Council UK. We also acknowledge support from the Department of Health via the National Institute for Health Research (NIHR) comprehensive Biomedical Research Centre awards to Guy's & St Thomas' NHS Foundation Trust in partnership with King's College London and to the Cambridge University Hospitals NHS Foundation Trust in partnership with the University of Cambridge School of Clinical Medicine.

Membership of the Wellcome Trust Case Control Consortium (WTCCC):

Management Committee: Paul R Burton[1], David G Clayton[2], Lon R Cardon[3], Nick Craddock[4], Panos Deloukas[5], Audrey Duncanson[6], Dominic P Kwiatkowski[3,5], Mark I McCarthy[3,7], Willem H Ouwehand[8,9], Nilesh J Samani[10], John A Todd[2], Peter Donnelly (Chair)[11]

Data and Analysis Committee: Jeffrey C Barrett[3], Paul R Burton[1], Dan Davison[11], Peter Donnelly[11], Doug Easton[12], David M. Evans[3], Hin-Tak Leung[2], Jonathan L Marchini[11], Andrew P Morris[3], Chris CA Spencer[11], Martin D Tobin[1], Lon R Cardon (Co-chair)[3], David G Clayton (Co-chair)[2]

UK Blood Services & University of Cambridge Controls: Antony P Attwood[5,8], James P Boorman[8,9], Barbara Cant[8], Ursula Everson[13], Judith M Hussey[14], Jennifer D Jolley[8], Alexandra S Knight[8], Kerstin Koch[8], Elizabeth Meech[15], Sarah Nutland[2], Christopher V Prowse[16], Helen E Stevens[2], Niall C Taylor[8], Graham R Walters[17], Neil M Walker[2], Nicholas A Watkins[8,9], Thilo Winzer[8], John A Todd[2], Willem H Ouwehand[8,9]

1958 Birth Cohort Controls: Richard W Jones[18], Wendy L McArdle[18], Susan M Ring[18], David P Strachan[19], Marcus Pembrey[18,20]

Bipolar Disorder (Aberdeen): Gerome Breen[21], David St Clair[21]; (**Birmingham):** Sian Caesar[22], Katherine Gordon-Smith[22,23], Lisa Jones[22]; **(Cardiff):** Christine Fraser[23], Elaine K Green[23], Detelina Grozeva[23], Marian L Hamshere[23], Peter A Holmans[23], Ian R Jones[23], George Kirov[23], Valentina Moskvina[23], Ivan Nikolov[23], Michael C O'Donovan[23], Michael J Owen[23], Nick Craddock[23]; **(London):** David A Collier[24], Amanda Elkin[24], Anne Farmer[24], Richard Williamson[24], Peter McGuffin[24]; **(Newcastle):** Allan H Young[25], I Nicol Ferrier[25]

Coronary Artery Disease (Leeds): Stephen G Ball[26], Anthony J Balmforth[26], Jennifer H Barrett[26], D Timothy Bishop[26], Mark M Iles[26], Azhar Maqbool[26], Nadira Yuldasheva[26], Alistair S Hall[26]; **(Leicester):** Peter S Braund[10], Paul R Burton[1], Richard J Dixon[10], Massimo Mangino[10], Suzanne Stevens[10], Martin D Tobin[1], John R Thompson[1], Nilesh J Samani[10]

Crohn's Disease (Cambridge): Francesca Bredin[27], Mark Tremelling[27], Miles Parkes[27]; **(Edinburgh):** Hazel Drummond[28], Charles W Lees[28], Elaine R Nimmo[28], Jack Satsangi[28];
(London): Sheila A Fisher[29], Alastair Forbes[30], Cathryn M Lewis[29], Clive M Onnie[29], Natalie J Prescott[29], Jeremy Sanderson[31], Christopher G Mathew[29]; **(Newcastle):** Jamie Barbour[32], M Khalid Mohiuddin[32], Catherine E Todhunter[32], John C Mansfield[32]; **(Oxford):** Tariq Ahmad[33], Fraser R Cummings[33], Derek P Jewell[33]

Hypertension (Aberdeen): John Webster[34]; **(Cambridge):** Morris J Brown[35], David G Clayton[2]; **(Evry, France):** G Mark Lathrop[36]; **(Glasgow):** John Connell[37], Anna Dominiczak[37]; **(Leicester):** Nilesh J Samani[10]; **(London):** Carolina A Braga Marcano[38], Beverley Burke[38], Richard Dobson[38], Johannie Gungadoo[38], Kate L Lee[38], Patricia B Munroe[38], Stephen J Newhouse[38], Abiodun Onipinla[38], Chris Wallace[38], Mingzhan Xue[38], Mark Caulfield[38]; **(Oxford):** Martin Farrall[39]

Rheumatoid Arthritis: Anne Barton[40], Ian N Bruce[40], Hannah Donovan[40], Steve Eyre[40], Paul D Gilbert[40], Samantha L Hider[40], Anne M Hinks[40], Sally L John[40], Catherine Potter[40], Alan J Silman[40], Deborah PM Symmons[40], Wendy Thomson[40], Jane Worthington[40]

Type 1 Diabetes: David G Clayton[2], David B Dunger[2,41], Sarah Nutland[2], Helen E Stevens[2], Neil M Walker[2], Barry Widmer[2,41], John A Todd[2]

Type 2 Diabetes (Exeter): Timothy M Frayling[42,43], Rachel M Freathy[42,43], Hana Lango[42,43], John R B Perry[42,43], Beverley M Shields[43], Michael N Weedon[42,43], Andrew T Hattersley[42,43]; **(London):** Graham A Hitman[44]; **(Newcastle):** Mark Walker[45]; **(Oxford):** Kate S Elliott[3,7], Christopher J Groves[7], Cecilia M Lindgren[3,7], Nigel W Rayner[3,7], Nicholas J Timpson[3,46], Eleftheria Zeggini[3,7], Mark I McCarthy[3,7]

Tuberculosis (Gambia): Melanie Newport[47], Giorgio Sirugo[47]; **(Oxford):** Emily Lyons[3], Fredrik Vannberg[3], Adrian VS Hill[3]

Ankylosing Spondylitis: Linda A Bradbury[48], Claire Farrar[49], Jennifer J Pointon[48], Paul Wordsworth[49], Matthew A Brown[48,49]

AutoImmune Thyroid Disease: Jayne A Franklyn[50], Joanne M Heward[50], Matthew J Simmonds[50], Stephen CL Gough[50]

Breast Cancer: Sheila Seal[51], Michael R Stratton[51,52], Nazneen Rahman[51]

Multiple Sclerosis: Maria Ban[53], An Goris[53], Stephen J Sawcer[53], Alastair Compston[53]

Gambian Controls (Gambia): David Conway[47], Muminatou Jallow[47], Melanie Newport[47], Giorgio Sirugo[47]; **(Oxford):** Kirk A Rockett[3], Dominic P Kwiatkowski[3,5]

DNA, Genotyping, Data QC and Informatics (Wellcome Trust Sanger Institute, Hinxton): Suzannah J Bumpstead[5], Amy Chaney[5], Kate Downes[2,5], Mohammed JR Ghori[5], Rhian Gwilliam[5], Sarah E Hunt[5], Michael Inouye[5], Andrew Keniry[5], Emma King[5], Ralph McGinnis[5], Simon Potter[5], Rathi Ravindrarajah[5], Pamela

Whittaker[5], Claire Widden[5], David Withers[5], Panos Deloukas[5]; **(Cambridge):** Hin- Tak Leung[2], Sarah Nutland[2], Helen E Stevens[2], Neil M Walker[2], John A Todd[2] **Statistics (Cambridge):** Doug Easton[12], David G Clayton[2]; **(Leicester):** Paul R Burton[1], Martin D Tobin[1]; **(Oxford):** Jeffrey C Barrett[3], David M Evans[3], Andrew P Morris[3], Lon R Cardon[3]; **(Oxford):** Niall J Cardin[11], Dan Davison[11], Teresa Ferreira[11], Joanne Pereira-Gale[11], Ingeleif B Hallgrimsdóttir[11], Bryan N Howie[11], Jonathan L Marchini[11], Chris CA Spencer[11], Zhan Su[11], Yik Ying Teo[3,11], Damjan Vukcevic[11], Peter Donnelly[11] **PIs:** David Bentley[5,54], Matthew A Brown[48,49], Lon R Cardon[3], Mark Caulfield[38], David G Clayton[2], Alistair Compston[53], Nick Craddock[23], Panos Deloukas[5], Peter Donnelly[11], Martin Farrall[39], Stephen CL Gough[50], Alistair S Hall[26], Andrew T Hattersley[42,43], Adrian VS Hill[3], Dominic P Kwiatkowski[3,5], Christopher G Mathew[29], Mark I McCarthy[3,7], Willem H Ouwehand[8,9], Miles Parkes[27], Marcus Pembrey[18,20], Nazneen Rahman[51], Nilesh J Samani[10], Michael R Stratton[51,52], John A Todd[2], Jane Worthington[40]

WTCCC Affiliations: 1 Genetic Epidemiology Group, Department of Health Sciences, University of Leicester, Adrian Building, University Road, Leicester, LE1 7RH, UK; 2 Juvenile Diabetes Research Foundation/WellcomeTrust Diabetes and Inflammation Laboratory, Department of Medical Genetics, Cambridge Institute for Medical Research, University of Cambridge, Wellcome Trust/MRC Building, Cambridge, CB2 0XY, UK; 3 Wellcome Trust Centre for Human Genetics, University of Oxford, Roosevelt Drive, Oxford OX3 7BN, UK; 4 Department of Psychological Medicine, Henry Wellcome Building, School of Medicine, Cardiff University, Heath Park, Cardiff CF14 4XN, UK; 5 The Wellcome Trust Sanger Institute, Wellcome Trust Genome Campus, Hinxton, Cambridge CB10 1SA, UK; 6 The Wellcome Trust, Gibbs Building, 215 Euston Road, London NW1 2BE, UK; 7 Oxford Centre for Diabetes, Endocrinology and Medicine, University of Oxford, Churchill Hospital, Oxford, OX3 7LJ, UK; 8 Department of Haematology, University of Cambridge, Long Road, Cambridge, CB2 2PT, UK; 9 National Health Service Blood and Transplant, Cambridge Centre, Long Road, Cambridge, CB2 2PT, UK; 10 Department of Cardiovascular Sciences, University of Leicester, Glenfield Hospital, Groby Road, Leicester, LE3 9QP, UK; 11 Department of Statistics, University of Oxford, 1 South Parks Road, Oxford OX1 3TG, UK; 12 Cancer Research UK Genetic Epidemiology Unit, Strangeways Research Laboratory, Worts Causeway, Cambridge CB1 8RN, UK; 13 National Health Service Blood and Transplant, Sheffield Centre, Longley Lane, Sheffield S5 7JN, UK; 14 National Health Service Blood and Transplant, Brentwood Centre, Crescent Drive, Brentwood, CM15 8DP, UK; 15 The Welsh Blood Service, Ely Valley Road, Talbot Green, Pontyclun, CF72 9WB, UK; 16 The Scottish National Blood Transfusion Service, Ellen’s Glen Road, Edinburgh, EH17 7QT, UK; 17 National Health Service Blood and Transplant, Southampton Centre, Coxford Road, Southampton, SO16 5AF, UK; 18 Avon Longitudinal Study of Parents and Children, University of Bristol, 24 Tyndall Avenue, Bristol, BS8 1TQ, UK; 19 Division of Community Health Services, St George’s University of London, Cranmer Terrace, London SW17 0RE, UK; 20 Institute of Child Health, University College London, 30 Guilford St, London WC1N 1EH, UK; 21 University of Aberdeen, Institute of Medical Sciences, Foresterhill, Aberdeen, AB25 2ZD, UK; 22 Department of Psychiatry, Division of Neuroscience, Birmingham University, Birmingham, B15 2QZ, UK; 23 Department of Psychological Medicine, Henry Wellcome Building, School of Medicine, Cardiff University, Heath Park, Cardiff CF14 4XN, UK; 24 SGDP, The Institute of Psychiatry, King's College London, De Crespigny Park Denmark Hill London SE5 8AF, UK; 25 School of Neurology, Neurobiology and Psychiatry, Royal Victoria Infirmary, Queen Victoria Road, Newcastle upon Tyne, NE1 4LP, UK; 26 LIGHT and LIMM Research Institutes, Faculty of Medicine and Health, University of Leeds, Leeds, LS1 3EX, UK; 27 IBD Research Group, Addenbrooke's Hospital, University of Cambridge, Cambridge, CB2 2QQ, UK; 28 Gastrointestinal Unit, School of Molecular and Clinical Medicine, University of Edinburgh, Western General Hospital, Edinburgh EH4 2XU UK; 29 Department of Medical & Molecular Genetics, King's College London School of Medicine, 8th Floor Guy's Tower, Guy's Hospital, London, SE1 9RT, UK; 30 Institute for Digestive Diseases, University College London Hospitals Trust, London, NW1 2BU, UK; 31 Department of Gastroenterology, Guy's and St Thomas' NHS Foundation Trust, London, SE1 7EH, UK; 32 Department of Gastroenterology & Hepatology, University of Newcastle upon Tyne, Royal Victoria Infirmary, Newcastle upon Tyne, NE1 4LP, UK; 33 Gastroenterology Unit, Radcliffe Infirmary, University of Oxford, Oxford, OX2 6HE, UK; 34 Medicine and Therapeutics, Aberdeen Royal Infirmary, Foresterhill, Aberdeen, Grampian AB9 2ZB, UK; 35 Clinical Pharmacology Unit and the Diabetes and Inflammation Laboratory, University of Cambridge, Addenbrookes Hospital, Hills Road, Cambridge CB2 2QQ, UK; 36 Centre National de Genotypage, 2, Rue Gaston Cremieux, Evry, Paris 91057.; 37 BHF Glasgow Cardiovascular Research Centre, University of Glasgow, 126 University Place, Glasgow, G12 8TA, UK; 38 Clinical Pharmacology and Barts and The London Genome Centre, William Harvey Research Institute, Barts and The London, Queen Mary’s School of Medicine, Charterhouse Square, London EC1M 6BQ, UK; 39 Cardiovascular Medicine, University of Oxford, Wellcome Trust Centre for Human Genetics, Roosevelt Drive, Oxford OX3 7BN, UK; 40arc Epidemiology Research Unit, University of Manchester, Stopford Building, Oxford Rd, Manchester, M13 9PT, UK; 41 Department of Paediatrics, University of Cambridge, Addenbrooke’s Hospital, Cambridge, CB2 2QQ, UK; 42 Genetics of

Complex Traits, Institute of Biomedical and Clinical Science, Peninsula Medical School, Magdalen Road, Exeter EX1 2LU UK; 43 Diabetes Genetics, Institute of Biomedical and Clinical Science, Peninsula Medical School, Barrack Road, Exeter EX2 5DU UK; 44 Centre for Diabetes and Metabolic Medicine, Barts and The London, Royal London Hospital, Whitechapel, London, E1 1BB UK; 45 Diabetes Research Group, School of Clinical Medical Sciences, Newcastle University, Framlington Place, Newcastle upon Tyne NE2 4HH, UK; 46 The MRC Centre for Causal Analyses in Translational Epidemiology, Bristol University, Canynge Hall, Whiteladies Rd, Bristol BS2 8PR, UK; 47 MRC Laboratories, Fajara, The Gambia; 48 Diamantina Institute for Cancer, Immunology and Metabolic Medicine, Princess Alexandra Hospital, University of Queensland, Woolloongabba, Qld 4102, Australia; 49 Botnar Research Centre, University of Oxford, Headington, Oxford OX3 7BN, UK; 50 Department of Medicine, Division of Medical Sciences, Institute of Biomedical Research, University of Birmingham, Edgbaston, Birmingham B15 2TT, UK; 51 Section of Cancer Genetics, Institute of Cancer Research, 15 Cotswold Road, Sutton, SM2 5NG, UK; 52 Cancer Genome Project, The Wellcome Trust Sanger Institute, Wellcome Trust Genome Campus, Hinxton, Cambridge CB10 1SA, UK; 53 Department of Clinical Neurosciences, University of Cambridge, Addenbrooke's Hospital, Hills Road, Cambridge CB2 2QQ, UK; 54 PRESENT ADDRESS: Illumina Cambridge, Chesterford Research Park, Little Chesterford, Nr Saffron Walden, Essex, CB10 1XL, UK.

References

1. Miller MR, Hankinson J, Brusasco V, Burgos F, Casaburi R, Coates A, et al. Standardisation of spirometry. European Respiratory Journal. 2005;26(2):319-38.
2. Hancock DB, Eijgelsheim M, Wilk JB, Gharib SA, Loehr LR, Marciante KD, et al. Meta-analyses of genome-wide association studies identify multiple loci associated with pulmonary function. Nature genetics. 2010;42(1):45-52. Epub 2009/12/17.
3. Repapi E, Sayers I, Wain LV, Burton PR, Johnson T, Obeidat M, et al. Genome-wide association study identifies five loci associated with lung function. Nature genetics. 2010;42(1):36-44. Epub 2009/12/17.
4. Soler Artigas M, Loth DW, Wain LV, Gharib SA, Obeidat M, Tang W, et al. Genome-wide association and large-scale follow up identifies 16 new loci influencing lung function. Nature genetics. 2011;43(11):1082-90. Epub 2011/09/29.
5. Wilk JB, Chen TH, Gottlieb DJ, Walter RE, Nagle MW, Brandler BJ, et al. A genome-wide association study of pulmonary function measures in the Framingham Heart Study. PLoS genetics. 2009;5(3):e1000429. Epub 2009/03/21.
6. Imboden M, Bouzigon E, Curjuric I, Ramasamy A, Kumar A, Hancock DB, et al. Genome-wide association study of lung function decline in adults with and without asthma. The Journal of allergy and clinical immunology. 2012;129(5):1218-28. Epub 2012/03/20.
7. Wain LV, Sayers I, Soler Artigas M, Portelli MA, Zeggini E, Obeidat M, et al. Whole exome re-sequencing implicates CCDC38 and cilia structure and function in resistance to smoking related airflow obstruction. PLoS genetics. 2014;10(5):e1004314. Epub 2014/05/03.
8. Wilk JB, Shrine NR, Loehr LR, Zhao JH, Manichaikul A, Lopez LM, et al. Genome-wide association studies identify CHRNA5/3 and HTR4 in the development of airflow obstruction. American journal of respiratory and critical care medicine. 2012;186(7):622-32. Epub 2012/07/28.
9. Hunninghake GM, Cho MH, Tesfaigzi Y, Soto-Quiros ME, Avila L, Lasky-Su J, et al. MMP12, lung function, and COPD in high-risk populations. The New England journal of medicine. 2009;361(27):2599-608. Epub 2009/12/19.
10. Obeidat M, Wain LV, Shrine N, Kalsheker N, Soler Artigas M, Repapi E, et al. A comprehensive evaluation of potential lung function associated genes in the SpiroMeta general population sample. PloS one. 2011;6(5):e19382. Epub 2011/06/01.
11. Wan YI, Shrine NR, Soler Artigas M, Wain LV, Blakey JD, Moffatt MF, et al. Genome-wide association study to identify genetic determinants of severe asthma. Thorax. 2012;67(9):762-8. Epub 2012/05/09.
12. Liu JZ, Tozzi F, Waterworth DM, Pillai SG, Muglia P, Middleton L, et al. Meta-analysis and imputation refines the association of 15q25 with smoking quantity. Nature genetics. 2010;42(5):436-40. Epub 2010/04/27.
13. Thorgeirsson TE, Gudbjartsson DF, Surakka I, Vink JM, Amin N, Geller F, et al. Sequence variants at CHRNB3-CHRNA6 and CYP2A6 affect smoking behavior. Nature genetics. 2010;42(5):448-53. Epub 2010/04/27.
14. Tobacco and Genetics Consortium. Genome-wide meta-analyses identify multiple loci associated with smoking behavior. Nature genetics. 2010;42(5):441-7. Epub 2010/04/27.
15. Mushiroda T, Wattanapokayakit S, Takahashi A, Nukiwa T, Kudoh S, Ogura T, et al. A genome-wide association study identifies an association of a common variant in TERT with susceptibility to idiopathic pulmonary fibrosis. Journal of medical genetics. 2008;45(10):654-6. Epub 2008/10/07.
16. Seibold MA, Wise AL, Speer MC, Steele MP, Brown KK, Loyd JE, et al. A common MUC5B promoter polymorphism and pulmonary fibrosis. The New England journal of medicine. 2011;364(16):1503-12. Epub 2011/04/22.
17. Lan Q, Hsiung CA, Matsuo K, Hong YC, Seow A, Wang Z, et al. Genome-wide association analysis identifies new lung cancer susceptibility loci in never-smoking women in Asia. Nature genetics. 2012;44(12):1330-5. Epub 2012/11/13.

18. Tsakiri KD, Cronkhite JT, Kuan PJ, Xing C, Raghu G, Weissler JC, et al. Adult-onset pulmonary fibrosis caused by mutations in telomerase. Proceedings of the National Academy of Sciences of the United States of America. 2007;104(18):7552-7. Epub 2007/04/27.
19. Purcell S, Neale B, Todd-Brown K, Thomas L, Ferreira MA, Bender D, et al. PLINK: a tool set for whole-genome association and population-based linkage analyses. American journal of human genetics. 2007;81(3):559-75. Epub 2007/08/19.
20. The 1000 Genomes Project Consortium, Abecasis GR, Altshuler D, Auton A, Brooks LD, Durbin RM, et al. A map of human genome variation from population-scale sequencing. Nature. 2010;467(7319):1061-73. Epub 2010/10/29.
21. U10K Consortium. The UK10K project identifies rare variants in health and disease. Nature. 2015. Epub 2015/09/15.
22. Huang J, Howie B, McCarthy S, Memari Y, Walter K, Min JL, et al. Improved imputation of low-frequency and rare variants using the UK10K haplotype reference panel. Nature communications. 2015;6:8111.
23. Marchini J, Howie B, Myers S, McVean G, Donnelly P. A new multipoint method for genome-wide association studies by imputation of genotypes. Nature genetics. 2007;39(7):906-13. Epub 2007/06/19.
24. Ma C, Blackwell T, Boehnke M, Scott LJ, Go TDi. Recommended joint and meta-analysis strategies for case-control association testing of single low-count variants. Genet Epidemiol. 2013;37(6):539-50. Epub 2013/06/22.
25. Wang K, Li M, Hakonarson H. ANNOVAR: functional annotation of genetic variants from high-throughput sequencing data. Nucleic Acids Res. 2010;38(16):e164. Epub 2010/07/06.
26. International Schizophrenia Consortium, Purcell SM, Wray NR, Stone JL, Visscher PM, O'Donovan MC, et al. Common polygenic variation contributes to risk of schizophrenia and bipolar disorder. Nature. 2009;460(7256):748-52. Epub 2009/07/03.
27. Yang J, Benyamin B, McEvoy BP, Gordon S, Henders AK, Nyholt DR, et al. Common SNPs explain a large proportion of the heritability for human height. Nature genetics. 2010;42(7):565-9. Epub 2010/06/22.
28. Yang J, Lee SH, Goddard ME, Visscher PM. GCTA: a tool for genome-wide complex trait analysis. American journal of human genetics. 2011;88(1):76-82. Epub 2010/12/21.
29. Fisher RA. The correlation between relatives on the supposition of Mendelian inheritance. Philos Trans R Soc Edinb. 1918;52:399-433.
30. Lee SH, Wray NR, Goddard ME, Visscher PM. Estimating missing heritability for disease from genome-wide association studies. American journal of human genetics. 2011;88(3):294-305. Epub 2011/03/08.
31. Ferreira MA, Matheson MC, Duffy DL, Marks GB, Hui J, Le Souef P, et al. Identification of IL6R and chromosome 11q13.5 as risk loci for asthma. Lancet. 2011;378(9795):1006-14. Epub 2011/09/13.
32. Moffatt MF, Gut IG, Demenais F, Strachan DP, Bouzigon E, Heath S, et al. A large-scale, consortium-based genomewide association study of asthma. The New England journal of medicine. 2010;363(13):1211-21. Epub 2010/09/24.
33. Ramasamy A, Kuokkanen M, Vedantam S, Gajdos ZK, Couto Alves A, Lyon HN, et al. Genome-wide association studies of asthma in population-based cohorts confirm known and suggested loci and identify an additional association near HLA. PloS one. 2012;7(9):e44008. Epub 2012/10/03.
34. Torgerson DG, Ampleford EJ, Chiu GY, Gauderman WJ, Gignoux CR, Graves PE, et al. Meta-analysis of genome-wide association studies of asthma in ethnically diverse North American populations. Nature genetics. 2011;43(9):887-92. Epub 2011/08/02.
35. Hao K, Bosse Y, Nickle DC, Pare PD, Postma DS, Laviolette M, et al. Lung eQTLs to help reveal the molecular underpinnings of asthma. PLoS genetics. 2012;8(11):e1003029. Epub 2012/12/05.

36. Lamontagne M, Couture C, Postma DS, Timens W, Sin DD, Pare PD, et al. Refining susceptibility loci of chronic obstructive pulmonary disease with lung eqtls. PloS one. 2013;8(7):e70220. Epub 2013/08/13.
37. Obeidat M, Miller S, Probert K, Billington CK, Henry AP, Hodge E, et al. GSTCD and INTS12 regulation and expression in the human lung. PloS one. 2013;8(9):e74630. Epub 2013/09/24.
38. Irizarry RA, Hobbs B, Collin F, Beazer-Barclay YD, Antonellis KJ, Scherf U, et al. Exploration, normalization, and summaries of high density oligonucleotide array probe level data. Biostatistics. 2003;4(2):249-64. Epub 2003/08/20.
39. Westra HJ, Peters MJ, Esko T, Yaghootkar H, Schurmann C, Kettunen J, et al. Systematic identification of trans eQTLs as putative drivers of known disease associations. Nature genetics. 2013;45(10):1238-43. Epub 2013/09/10.
40. Ramasamy A, Trabzuni D, Guelfi S, Varghese V, Smith C, Walker R, et al. Genetic variability in the regulation of gene expression in ten regions of the human brain. Nature neuroscience. 2014;17(10):1418-28. Epub 2014/09/01.
41. Steiling K, van den Berge M, Hijazi K, Florido R, Campbell J, Liu G, et al. A dynamic bronchial airway gene expression signature of chronic obstructive pulmonary disease and lung function impairment. American journal of respiratory and critical care medicine. 2013;187(9):933-42. Epub 2013/03/09.
42. Benjamini Y, Hochberg Y. Controlling the false discovery rate: a practical and powerful approach to multiple testing. Journal of the Royal Statistical Society Series B (Methodological). 1995:289-300.
43. Melen E, Kho AT, Sharma S, Gaedigk R, Leeder JS, Mariani TJ, et al. Expression analysis of asthma candidate genes during human and murine lung development. Respiratory research. 2011;12:86. Epub 2011/06/28.
44. Segre AV, Consortium D, investigators M, Groop L, Mootha VK, Daly MJ, et al. Common inherited variation in mitochondrial genes is not enriched for associations with type 2 diabetes or related glycemic traits. PLoS genetics. 2010;6(8). Epub 2010/08/18.
45. Loth DW, Artigas MS, Gharib SA, Wain LV, Franceschini N, Koch B, et al. Genome-wide association analysis identifies six new loci associated with forced vital capacity. Nature genetics. 2014;46(7):669-77. Epub 2014/06/16.
46. Yang J, Ferreira T, Morris AP, Medland SE, Genetic Investigation of ATC, Replication DIG, et al. Conditional and joint multiple-SNP analysis of GWAS summary statistics identifies additional variants influencing complex traits. Nature genetics. 2012;44(4):369-75, S1-3. Epub 2012/03/20.
47. Boettger LM, Handsaker RE, Zody MC, McCarroll SA. Structural haplotypes and recent evolution of the human 17q21.31 region. Nature genetics. 2012;44(8):881-5. Epub 2012/07/04.
48. Browning SR, Browning BL. Rapid and accurate haplotype phasing and missing-data inference for whole-genome association studies by use of localized haplotype clustering. American journal of human genetics. 2007;81(5):1084-97. Epub 2007/10/10.
49. Delaneau O, Zagury JF, Marchini J. Improved whole-chromosome phasing for disease and population genetic studies. Nature methods. 2013;10(1):5-6. Epub 2012/12/28.
50. Howie B, Fuchsberger C, Stephens M, Marchini J, Abecasis GR. Fast and accurate genotype imputation in genome-wide association studies through pre-phasing. Nature genetics. 2012;44(8):955-9. Epub 2012/07/24.
51. McLaren W, Pritchard B, Rios D, Chen Y, Flicek P, Cunningham F. Deriving the consequences of genomic variants with the Ensembl API and SNP Effect Predictor. Bioinformatics. 2010;26(16):2069-70.
52. Kumar P, Henikoff S, Ng PC. Predicting the effects of coding non-synonymous variants on protein function using the SIFT algorithm. Nature protocols. 2009;4(7):1073-81.
53. Adzhubei IA, Schmidt S, Peshkin L, Ramensky VE, Gerasimova A, Bork P, et al. A method and server for predicting damaging missense mutations. Nature methods. 2010;7(4):248-9.

54. Kircher M, Witten DM, Jain P, O'Roak BJ, Cooper GM, Shendure J. A general framework for estimating the relative pathogenicity of human genetic variants. Nature genetics. 2014;46(3):310-5.
55. Ritchie GR, Dunham I, Zeggini E, Flicek P. Functional annotation of noncoding sequence variants. Nature methods. 2014;11(3):294-6.
56. Lee S, Emond MJ, Bamshad MJ, Barnes KC, Rieder MJ, Nickerson DA, et al. Optimal unified approach for rare-variant association testing with application to small-sample case-control whole-exome sequencing studies. American journal of human genetics. 2012;91(2):224-37.
57. Wellcome Trust Case Control Consortium. Genome-wide association study of 14,000 cases of seven common diseases and 3,000 shared controls. Nature. 2007;447(7145):661-78. Epub 2007/06/08.
58. Basson CT, Bachinsky DR, Lin RC, Levi T, Elkins JA, Soults J, et al. Mutations in human TBX5 [corrected] cause limb and cardiac malformation in Holt-Oram syndrome. Nature genetics. 1997;15(1):30-5. Epub 1997/01/01.
59. Arora R, Metzger RJ, Papaioannou VE. Multiple roles and interactions of Tbx4 and Tbx5 in development of the respiratory system. PLoS genetics. 2012;8(8):e1002866. Epub 2012/08/10.
60. Tseng YR, Su YN, Lu FL, Jeng SF, Hsieh WS, Chen CY, et al. Holt-Oram syndrome with right lung agenesis caused by a de novo mutation in the TBX5 gene. American journal of medical genetics Part A. 2007;143A(9):1012-4. Epub 2007/03/17.
61. Cebra-Thomas JA, Bromer J, Gardner R, Lam GK, Sheipe H, Gilbert SF. T-box gene products are required for mesenchymal induction of epithelial branching in the embryonic mouse lung. Developmental dynamics : an official publication of the American Association of Anatomists. 2003;226(1):82-90. Epub 2003/01/01.
62. Holm H, Gudbjartsson DF, Arnar DO, Thorleifsson G, Thorgeirsson G, Stefansdottir H, et al. Several common variants modulate heart rate, PR interval and QRS duration. Nature genetics. 2010;42(2):117-22. Epub 2010/01/12.
63. Butler AM, Yin X, Evans DS, Nalls MA, Smith EN, Tanaka T, et al. Novel loci associated with PR interval in a genome-wide association study of 10 African American cohorts. Circulation Cardiovascular genetics. 2012;5(6):639-46. Epub 2012/11/10.
64. Lorenzen JA, Bonacci BB, Palmer RE, Wells C, Zhang J, Haber DA, et al. Rbm19 is a nucleolar protein expressed in crypt/progenitor cells of the intestinal epithelium. Gene expression patterns : GEP. 2005;6(1):45-56. Epub 2005/07/20.
65. Kallberg Y, Segerstolpe A, Lackmann F, Persson B, Wieslander L. Evolutionary conservation of the ribosomal biogenesis factor Rbm19/Mrd1: implications for function. PloS one. 2012;7(9):e43786. Epub 2012/09/18.
66. Zhang J, Tomasini AJ, Mayer AN. RBM19 is essential for preimplantation development in the mouse. BMC developmental biology. 2008;8:115. Epub 2008/12/18.
67. Borozdin W, Bravo-Ferrer Acosta AM, Seemanova E, Leipoldt M, Bamshad MJ, Unger S, et al. Contiguous hemizygous deletion of TBX5, TBX3, and RBM19 resulting in a combined phenotype of Holt-Oram and ulnar-mammary syndromes. American journal of medical genetics Part A. 2006;140A(17):1880-6. Epub 2006/08/08.
68. Latourelle JC, Dumitriu A, Hadzi TC, Beach TG, Myers RH. Evaluation of Parkinson disease risk variants as expression-QTLs. PloS one. 2012;7(10):e46199. Epub 2012/10/17.
69. Veerappa AM, Saldanha M, Padakannaya P, Ramachandra NB. Family based genome-wide copy number scan identifies complex rearrangements at 17q21.31 in dyslexics. American journal of medical genetics Part B, Neuropsychiatric genetics : the official publication of the International Society of Psychiatric Genetics. 2014;165B(7):572-80. Epub 2014/08/21.
70. Cai Y, Jin J, Swanson SK, Cole MD, Choi SH, Florens L, et al. Subunit composition and substrate specificity of a MOF-containing histone acetyltransferase distinct from the male-specific lethal (MSL) complex. The Journal of biological chemistry. 2010;285(7):4268-72. Epub 2009/12/19.
71. Dias J, Van Nguyen N, Georgiev P, Gaub A, Brettschneider J, Cusack S, et al. Structural analysis of the KANSL1/WDR5/KANSL2 complex reveals that WDR5 is required for efficient assembly

and chromatin targeting of the NSL complex. Genes & development. 2014;28(9):929-42. Epub 2014/05/03.
72. Zollino M, Orteschi D, Murdolo M, Lattante S, Battaglia D, Stefanini C, et al. Mutations in KANSL1 cause the 17q21.31 microdeletion syndrome phenotype. Nature genetics. 2012;44(6):636-8. Epub 2012/05/01.
73. Koolen DA, Kramer JM, Neveling K, Nillesen WM, Moore-Barton HL, Elmslie FV, et al. Mutations in the chromatin modifier gene KANSL1 cause the 17q21.31 microdeletion syndrome. Nature genetics. 2012;44(6):639-41. Epub 2012/05/01.
74. Ikram MA, Fornage M, Smith AV, Seshadri S, Schmidt R, Debette S, et al. Common variants at 6q22 and 17q21 are associated with intracranial volume. Nature genetics. 2012;44(5):539-44. Epub 2012/04/17.
75. Dawson HN, Ferreira A, Eyster MV, Ghoshal N, Binder LI, Vitek MP. Inhibition of neuronal maturation in primary hippocampal neurons from tau deficient mice. Journal of cell science. 2001;114(Pt 6):1179-87. Epub 2001/03/03.
76. Grundke-Iqbal I, Iqbal K, Quinlan M, Tung YC, Zaidi MS, Wisniewski HM. Microtubule-associated protein tau. A component of Alzheimer paired helical filaments. The Journal of biological chemistry. 1986;261(13):6084-9. Epub 1986/05/05.
77. Dumanchin C, Camuzat A, Campion D, Verpillat P, Hannequin D, Dubois B, et al. Segregation of a missense mutation in the microtubule-associated protein tau gene with familial frontotemporal dementia and parkinsonism. Human molecular genetics. 1998;7(11):1825-9. Epub 1998/09/16.
78. Buee L, Delacourte A. Comparative biochemistry of tau in progressive supranuclear palsy, corticobasal degeneration, FTDP-17 and Pick's disease. Brain pathology (Zurich, Switzerland). 1999;9(4):681-93. Epub 1999/10/12.
79. Do CB, Tung JY, Dorfman E, Kiefer AK, Drabant EM, Francke U, et al. Web-based genome-wide association study identifies two novel loci and a substantial genetic component for Parkinson's disease. PLoS genetics. 2011;7(6):e1002141. Epub 2011/07/09.
80. International Parkinson Disease Genomics Consortium, Nalls MA, Plagnol V, Hernandez DG, Sharma M, Sheerin UM, et al. Imputation of sequence variants for identification of genetic risks for Parkinson's disease: a meta-analysis of genome-wide association studies. Lancet. 2011;377(9766):641-9. Epub 2011/02/05.
81. Simon-Sanchez J, Schulte C, Bras JM, Sharma M, Gibbs JR, Berg D, et al. Genome-wide association study reveals genetic risk underlying Parkinson's disease. Nature genetics. 2009;41(12):1308-12. Epub 2009/11/17.
82. U. K. Parkinson's Disease Consortium, Wellcome Trust Case Control Consortium, Spencer CC, Plagnol V, Strange A, Gardner M, et al. Dissection of the genetics of Parkinson's disease identifies an additional association 5' of SNCA and multiple associated haplotypes at 17q21. Human molecular genetics. 2011;20(2):345-53. Epub 2010/11/04.
83. Fingerlin TE, Murphy E, Zhang W, Peljto AL, Brown KK, Steele MP, et al. Genome-wide association study identifies multiple susceptibility loci for pulmonary fibrosis. Nature genetics. 2013;45(6):613-20. Epub 2013/04/16.
84. Hoglinger GU, Melhem NM, Dickson DW, Sleiman PM, Wang LS, Klei L, et al. Identification of common variants influencing risk of the tauopathy progressive supranuclear palsy. Nature genetics. 2011;43(7):699-705. Epub 2011/06/21.
85. Noth I, Zhang Y, Ma SF, Flores C, Barber M, Huang Y, et al. Genetic variants associated with idiopathic pulmonary fibrosis susceptibility and mortality: a genome-wide association study. The Lancet Respiratory medicine. 2013;1(4):309-17. Epub 2014/01/17.
86. Gragnoli C. Hypothesis of the neuroendocrine cortisol pathway gene role in the comorbidity of depression, type 2 diabetes, and metabolic syndrome. The application of clinical genetics. 2014;7:43-53. Epub 2014/05/13.

87. Timpl P, Spanagel R, Sillaber I, Kresse A, Reul JM, Stalla GK, et al. Impaired stress response and reduced anxiety in mice lacking a functional corticotropin-releasing hormone receptor 1. Nature genetics. 1998;19(2):162-6. Epub 1998/06/10.
88. Labermaier C, Kohl C, Hartmann J, Devigny C, Altmann A, Weber P, et al. A polymorphism in the Crhr1 gene determines stress vulnerability in male mice. Endocrinology. 2014;155(7):2500-10. Epub 2014/04/30.
89. Byers HM, Dagle JM, Klein JM, Ryckman KK, McDonald EL, Murray JC, et al. Variations in CRHR1 are associated with persistent pulmonary hypertension of the newborn. Pediatric research. 2012;71(2):162-7. Epub 2012/01/20.
90. Kim WJ, Sheen SS, Kim TH, Huh JW, Lee JH, Kim EK, et al. Association between CRHR1 polymorphism and improved lung function in response to inhaled corticosteroid in patients with COPD. Respirology (Carlton, Vic). 2009;14(2):260-3. Epub 2009/02/13.
91. Rogers AJ, Tantisira KG, Fuhlbrigge AL, Litonjua AA, Lasky-Su JA, Szefler SJ, et al. Predictors of poor response during asthma therapy differ with definition of outcome. Pharmacogenomics. 2009;10(8):1231-42. Epub 2009/08/12.
92. Tantisira KG, Lazarus R, Litonjua AA, Klanderman B, Weiss ST. Chromosome 17: association of a large inversion polymorphism with corticosteroid response in asthma. Pharmacogenetics and genomics. 2008;18(8):733-7. Epub 2008/07/16.
93. Van Wesenbeeck L, Odgren PR, Coxon FP, Frattini A, Moens P, Perdu B, et al. Involvement of PLEKHM1 in osteoclastic vesicular transport and osteopetrosis in incisors absent rats and humans. The Journal of clinical investigation. 2007;117(4):919-30. Epub 2007/04/04.
94. Permuth-Wey J, Lawrenson K, Shen HC, Velkova A, Tyrer JP, Chen Z, et al. Identification and molecular characterization of a new ovarian cancer susceptibility locus at 17q21.31. Nature communications. 2013;4:1627. Epub 2013/03/29.
95. Couch FJ, Wang X, McGuffog L, Lee A, Olswold C, Kuchenbaecker KB, et al. Genome-wide association study in BRCA1 mutation carriers identifies novel loci associated with breast and ovarian cancer risk. PLoS genetics. 2013;9(3):e1003212. Epub 2013/04/02.
96. Katoh M. Molecular cloning and characterization of human WNT3. International journal of oncology. 2001;19(5):977-82. Epub 2001/10/18.
97. Katoh M. Regulation of WNT3 and WNT3A mRNAs in human cancer cell lines NT2, MCF-7, and MKN45. International journal of oncology. 2002;20(2):373-7. Epub 2002/01/15.
98. Nakashima N, Liu D, Huang CL, Ueno M, Zhang X, Yokomise H. Wnt3 gene expression promotes tumor progression in non-small cell lung cancer. Lung cancer (Amsterdam, Netherlands). 2012;76(2):228-34. Epub 2011/11/11.
99. Wu Y, Ginther C, Kim J, Mosher N, Chung S, Slamon D, et al. Expression of Wnt3 activates Wnt/beta-catenin pathway and promotes EMT-like phenotype in trastuzumab-resistant HER2-overexpressing breast cancer cells. Molecular cancer research : MCR. 2012;10(12):1597-606. Epub 2012/10/17.
100. Niemann S, Zhao C, Pascu F, Stahl U, Aulepp U, Niswander L, et al. Homozygous WNT3 mutation causes tetra-amelia in a large consanguineous family. American journal of human genetics. 2004;74(3):558-63. Epub 2004/02/12.
101. Pankratz N, Beecham GW, DeStefano AL, Dawson TM, Doheny KF, Factor SA, et al. Meta-analysis of Parkinson's disease: identification of a novel locus, RIT2. Annals of neurology. 2012;71(3):370-84. Epub 2012/03/28.
102. Philipps DL, Wigglesworth K, Hartford SA, Sun F, Pattabiraman S, Schimenti K, et al. The dual bromodomain and WD repeat-containing mouse protein BRWD1 is required for normal spermiogenesis and the oocyte-embryo transition. Developmental biology. 2008;317(1):72-82. Epub 2008/03/21.
103. Lechner S, Muller-Ladner U, Neumann E, Spottl T, Schlottmann K, Ruschoff J, et al. Thioredoxin reductase 1 expression in colon cancer: discrepancy between in vitro and in vivo

findings. Laboratory investigation; a journal of technical methods and pathology. 2003;83(9):1321-31. Epub 2003/09/19.
104. Berggren M, Gallegos A, Gasdaska JR, Gasdaska PY, Warneke J, Powis G. Thioredoxin and thioredoxin reductase gene expression in human tumors and cell lines, and the effects of serum stimulation and hypoxia. Anticancer research. 1996;16(6B):3459-66. Epub 1996/11/01.
105. Gosens I, Sessa A, den Hollander AI, Letteboer SJ, Belloni V, Arends ML, et al. FERM protein EPB41L5 is a novel member of the mammalian CRB-MPP5 polarity complex. Experimental cell research. 2007;313(19):3959-70. Epub 2007/10/09.
106. Garre P, Briceno V, Xicola RM, Doyle BJ, de la Hoya M, Sanz J, et al. Analysis of the oxidative damage repair genes NUDT1, OGG1, and MUTYH in patients from mismatch repair proficient HNPCC families (MSS-HNPCC). Clinical cancer research : an official journal of the American Association for Cancer Research. 2011;17(7):1701-12. Epub 2011/03/01.
107. Namavar Y, Chitayat D, Barth PG, van Ruissen F, de Wissel MB, Poll-The BT, et al. TSEN54 mutations cause pontocerebellar hypoplasia type 5. European journal of human genetics : EJHG. 2011;19(6):724-6. Epub 2011/03/04.
108. Rudaks LI, Moore L, Shand KL, Wilkinson C, Barnett CP. Novel TSEN54 mutation causing pontocerebellar hypoplasia type 4. Pediatric neurology. 2011;45(3):185-8. Epub 2011/08/10.
109. Simonati A, Cassandrini D, Bazan D, Santorelli FM. TSEN54 mutation in a child with pontocerebellar hypoplasia type 1. Acta neuropathologica. 2011;121(5):671-3. Epub 2011/04/07.
110. Lowenstein EJ, Daly RJ, Batzer AG, Li W, Margolis B, Lammers R, et al. The SH2 and SH3 domain-containing protein GRB2 links receptor tyrosine kinases to ras signaling. Cell. 1992;70(3):431-42. Epub 1992/08/07.
111. Tahiliani M, Koh KP, Shen Y, Pastor WA, Bandukwala H, Brudno Y, et al. Conversion of 5-methylcytosine to 5-hydroxymethylcytosine in mammalian DNA by MLL partner TET1. Science (New York, NY). 2009;324(5929):930-5. Epub 2009/04/18.
112. Jankowska AM, Szpurka H, Tiu RV, Makishima H, Afable M, Huh J, et al. Loss of heterozygosity 4q24 and TET2 mutations associated with myelodysplastic/myeloproliferative neoplasms. Blood. 2009;113(25):6403-10. Epub 2009/04/18.
113. Berndt SI, Gustafsson S, Magi R, Ganna A, Wheeler E, Feitosa MF, et al. Genome-wide meta-analysis identifies 11 new loci for anthropometric traits and provides insights into genetic architecture. Nature genetics. 2013;45(5):501-12. Epub 2013/04/09.
114. Eeles RA, Kote-Jarai Z, Al Olama AA, Giles GG, Guy M, Severi G, et al. Identification of seven new prostate cancer susceptibility loci through a genome-wide association study. Nature genetics. 2009;41(10):1116-21. Epub 2009/09/22.
115. Lango Allen H, Estrada K, Lettre G, Berndt SI, Weedon MN, Rivadeneira F, et al. Hundreds of variants clustered in genomic loci and biological pathways affect human height. Nature. 2010;467(7317):832-8. Epub 2010/10/01.
116. Michailidou K, Hall P, Gonzalez-Neira A, Ghoussaini M, Dennis J, Milne RL, et al. Large-scale genotyping identifies 41 new loci associated with breast cancer risk. Nature genetics. 2013;45(4):353-61, 61e1-2. Epub 2013/03/29.
117. Lundin M, Baltscheffsky H, Ronne H. Yeast PPA2 gene encodes a mitochondrial inorganic pyrophosphatase that is essential for mitochondrial function. The Journal of biological chemistry. 1991;266(19):12168-72. Epub 1991/07/05.
118. Clark SL, Souza RP, Adkins DE, Aberg K, Bukszar J, McClay JL, et al. Genome-wide association study of patient-rated and clinician-rated global impression of severity during antipsychotic treatment. Pharmacogenetics and genomics. 2013;23(2):69-77. Epub 2012/12/18.
119. Chuong CM, Edelman GM. Alterations in neural cell adhesion molecules during development of different regions of the nervous system. The Journal of neuroscience : the official journal of the Society for Neuroscience. 1984;4(9):2354-68. Epub 1984/09/01.
120. Aletsee-Ufrecht MC, Langley K, Rotsch M, Havemann K, Gratzl M. NCAM: a surface marker for human small cell lung cancer cells. FEBS letters. 1990;267(2):295-300. Epub 1990/07/16.

121. Arnett DK, Meyers KJ, Devereux RB, Tiwari HK, Gu CC, Vaughan LK, et al. Genetic variation in NCAM1 contributes to left ventricular wall thickness in hypertensive families. Circulation research. 2011;108(3):279-83. Epub 2011/01/08.
122. Huang CC, Tu SH, Lien HH, Jeng JY, Huang CS, Huang CJ, et al. Concurrent gene signatures for han chinese breast cancers. PloS one. 2013;8(10):e76421. Epub 2013/10/08.
123. Uyama E, Tsukahara T, Goto K, Kurano Y, Ogawa M, Kim YJ, et al. Nuclear accumulation of expanded PABP2 gene product in oculopharyngeal muscular dystrophy. Muscle & nerve. 2000;23(10):1549-54. Epub 2000/09/26.
124. Braun S, Berg C, Buck S, Gregor M, Klein R. Catalytic domain of PDC-E2 contains epitopes recognized by antimitochondrial antibodies in primary biliary cirrhosis. World journal of gastroenterology : WJG. 2010;16(8):973-81. Epub 2010/02/25.
125. Head RA, Brown RM, Zolkipli Z, Shahdadpuri R, King MD, Clayton PT, et al. Clinical and genetic spectrum of pyruvate dehydrogenase deficiency: dihydrolipoamide acetyltransferase (E2) deficiency. Annals of neurology. 2005;58(2):234-41. Epub 2005/07/29.
126. Bingle CD, Bingle L. Characterisation of the human plunc gene, a gene product with an upper airways and nasopharyngeal restricted expression pattern. Biochimica et biophysica acta. 2000;1493(3):363-7. Epub 2000/10/06.
127. Lukinskiene L, Liu Y, Reynolds SD, Steele C, Stripp BR, Leikauf GD, et al. Antimicrobial activity of PLUNC protects against Pseudomonas aeruginosa infection. Journal of immunology (Baltimore, Md : 1950). 2011;187(1):382-90. Epub 2011/06/03.
128. Liu Y, Bartlett JA, Di ME, Bomberger JM, Chan YR, Gakhar L, et al. SPLUNC1/BPIFA1 contributes to pulmonary host defense against Klebsiella pneumoniae respiratory infection. The American journal of pathology. 2013;182(5):1519-31. Epub 2013/03/19.
129. Benlloch S, Galbis-Caravajal JM, Alenda C, Peiro FM, Sanchez-Ronco M, Rodriguez-Paniagua JM, et al. Expression of molecular markers in mediastinal nodes from resected stage I non-small-cell lung cancer (NSCLC): prognostic impact and potential role as markers of occult micrometastases. Annals of oncology : official journal of the European Society for Medical Oncology / ESMO. 2009;20(1):91-7. Epub 2008/07/31.
130. Rathbone SR, Glossop JR, Gough JE, Cartmell SH. Cyclic tensile strain upon human mesenchymal stem cells in 2D and 3D culture differentially influences CCNL2, WDR61 and BAHCC1 gene expression levels. Journal of the mechanical behavior of biomedical materials. 2012;11:82-91. Epub 2012/06/05.
131. Zhu B, Mandal SS, Pham AD, Zheng Y, Erdjument-Bromage H, Batra SK, et al. The human PAF complex coordinates transcription with events downstream of RNA synthesis. Genes & development. 2005;19(14):1668-73. Epub 2005/07/19.
132. Surapureddi S, Yu S, Bu H, Hashimoto T, Yeldandi AV, Kashireddy P, et al. Identification of a transcriptionally active peroxisome proliferator-activated receptor alpha -interacting cofactor complex in rat liver and characterization of PRIC285 as a coactivator. Proceedings of the National Academy of Sciences of the United States of America. 2002;99(18):11836-41. Epub 2002/08/22.
133. Fairfax BP, Makino S, Radhakrishnan J, Plant K, Leslie S, Dilthey A, et al. Genetics of gene expression in primary immune cells identifies cell type-specific master regulators and roles of HLA alleles. Nature genetics. 2012;44(5):502-10. Epub 2012/03/27.
134. Williams BC, Karr TL, Montgomery JM, Goldberg ML. The Drosophila l(1)zw10 gene product, required for accurate mitotic chromosome segregation, is redistributed at anaphase onset. The Journal of cell biology. 1992;118(4):759-73. Epub 1992/08/01.
135. Arasaki K, Uemura T, Tani K, Tagaya M. Correlation of Golgi localization of ZW10 and centrosomal accumulation of dynactin. Biochemical and biophysical research communications. 2007;359(3):811-6. Epub 2007/06/15.
136. Musio A, Mariani T, Montagna C, Zambroni D, Ascoli C, Ried T, et al. Recapitulation of the Roberts syndrome cellular phenotype by inhibition of INCENP, ZWINT-1 and ZW10 genes. Gene. 2004;331:33-40. Epub 2004/04/20.

137. Guastadisegni MC, Lonoce A, Impera L, Di Terlizzi F, Fugazza G, Aliano S, et al. CBFA2T2 and C20orf112: two novel fusion partners of RUNX1 in acute myeloid leukemia. Leukemia. 2010;24(8):1516-9. Epub 2010/06/04.
138. Gelsi-Boyer V, Trouplin V, Adelaide J, Bonansea J, Cervera N, Carbuccia N, et al. Mutations of polycomb-associated gene ASXL1 in myelodysplastic syndromes and chronic myelomonocytic leukaemia. British journal of haematology. 2009;145(6):788-800. Epub 2009/04/25.
139. McGinley AL, Li Y, Deliu Z, Wang QT. Additional sex combs-like family genes are required for normal cardiovascular development. Genesis (New York, NY : 2000). 2014;52(7):671-86. Epub 2014/05/28.
140. Guo Y, Yang MC, Weissler JC, Yang YS. PLAGL2 translocation and SP-C promoter activity--a cellular response of lung cells to hypoxia. Biochemical and biophysical research communications. 2007;360(3):659-65. Epub 2007/07/10.
141. Sekiya R, Maeda M, Yuan H, Asano E, Hyodo T, Hasegawa H, et al. PLAGL2 regulates actin cytoskeletal architecture and cell migration. Carcinogenesis. 2014;35(9):1993-2001. Epub 2014/03/29.
142. Yang YS, Yang MC, Weissler JC. Pleiomorphic adenoma gene-like 2 expression is associated with the development of lung adenocarcinoma and emphysema. Lung cancer (Amsterdam, Netherlands). 2011;74(1):12-24. Epub 2011/03/15.
143. Yang YS, Yang MC, Guo Y, Williams OW, Weissler JC. PLAGL2 expression-induced lung epithelium damages at bronchiolar alveolar duct junction in emphysema: bNip3- and SP-C-associated cell death/injury activity. American journal of physiology Lung cellular and molecular physiology. 2009;297(3):L455-66. Epub 2009/07/04.
144. Liu B, Lu C, Song YX, Gao P, Sun JX, Chen XW, et al. The role of pleomorphic adenoma gene-like 2 in gastrointestinal cancer development, progression, and prognosis. International journal of clinical and experimental pathology. 2014;7(6):3089-100. Epub 2014/07/18.
145. Zheng H, Ying H, Wiedemeyer R, Yan H, Quayle SN, Ivanova EV, et al. PLAGL2 regulates Wnt signaling to impede differentiation in neural stem cells and gliomas. Cancer cell. 2010;17(5):497-509. Epub 2010/05/19.
146. Dillon DA, Chen X, Zeimetz GM, Wu WI, Waggoner DW, Dewald J, et al. Mammalian Mg2+-independent phosphatidate phosphatase (PAP2) displays diacylglycerol pyrophosphate phosphatase activity. The Journal of biological chemistry. 1997;272(16):10361-6. Epub 1997/04/18.
147. Viterbo D, Bluth MH, Lin YY, Mueller CM, Wadgaonkar R, Zenilman ME. Pancreatitis-associated protein 2 modulates inflammatory responses in macrophages. Journal of immunology (Baltimore, Md : 1950). 2008;181(3):1948-58. Epub 2008/07/22.
148. Samant SA, Ogunkua O, Hui L, Fossella J, Pilder SH. The T complex distorter 2 candidate gene, Dnahc8, encodes at least two testis-specific axonemal dynein heavy chains that differ extensively at their amino and carboxyl termini. Developmental biology. 2002;250(1):24-43. Epub 2002/09/26.
149. Soler Artigas M, Wain LV, Miller S, Kheirallah AK, Huffman JE, Ntalla I, et al. Sixteen new lung function signals identified through 1000 Genomes Project reference panel imputation. Nat Comms. 2015; doi:10.1038/ncomms9658.
